# High throughput functional profiling of genes at intraocular pressure loci reveals distinct networks for glaucoma

**DOI:** 10.1093/hmg/ddae003

**Published:** 2024-01-25

**Authors:** Connor J Greatbatch, Qinyi Lu, Sandy Hung, Alexander J Barnett, Kristof Wing, Helena Liang, Xikun Han, Tiger Zhou, Owen M Siggs, David A Mackey, Anthony L Cook, Anne Senabouth, Guei-Sheung Liu, Jamie E Craig, Stuart MacGregor, Joseph E Powell, Alex W Hewitt

**Affiliations:** Menzies Institute for Medical Research, University of Tasmania, 17 Liverpool Street, Hobart, Tasmania 7000, Australia; Menzies Institute for Medical Research, University of Tasmania, 17 Liverpool Street, Hobart, Tasmania 7000, Australia; Centre for Eye Research Australia, University of Melbourne, Royal Victorian Eye and Ear Hospital, 32 Gisborne St, East Melbourne 3002, Australia; Menzies Institute for Medical Research, University of Tasmania, 17 Liverpool Street, Hobart, Tasmania 7000, Australia; Menzies Institute for Medical Research, University of Tasmania, 17 Liverpool Street, Hobart, Tasmania 7000, Australia; Centre for Eye Research Australia, University of Melbourne, Royal Victorian Eye and Ear Hospital, 32 Gisborne St, East Melbourne 3002, Australia; QIMR Berghofer Medical Research Institute, 300 Herston Rd, Herston, Brisbane 4006, Australia; Department of Ophthalmology, Flinders University, Flinders Medical Centre, 1 Flinders Dr, Bedford Park, South Australia 5042, Australia; Garvan Institute of Medical Research, 384 Victoria St, Darlinghurst, Sydney, NSW 2010, Australia; School of Clinical Medicine, Faculty of Medicine and Health, Short Street, St George Hospital KOGARAH UNSW, Sydney 2217, Australia; Menzies Institute for Medical Research, University of Tasmania, 17 Liverpool Street, Hobart, Tasmania 7000, Australia; Lions Eye Institute, Centre for Vision Sciences, University of Western Australia, 2 Verdun Street Nedlands WA 6009, Australia; Wicking Dementia Research and Education Centre, University of Tasmania, 17 Liverpool Street, Hobart, TAS 7000, Australia; Garvan-Weizmann Centre for Cellular Genomics, Garvan Institute of Medical Research, 384 Victoria St, Darlinghurst, Sydney, NSW 2010, Australia; Menzies Institute for Medical Research, University of Tasmania, 17 Liverpool Street, Hobart, Tasmania 7000, Australia; Department of Ophthalmology, Flinders University, Flinders Medical Centre, 1 Flinders Dr, Bedford Park, South Australia 5042, Australia; QIMR Berghofer Medical Research Institute, 300 Herston Rd, Herston, Brisbane 4006, Australia; Garvan-Weizmann Centre for Cellular Genomics, Garvan Institute of Medical Research, 384 Victoria St, Darlinghurst, Sydney, NSW 2010, Australia; UNSW Cellular Genomics Futures Institute, University of New South Wales, 384 Victoria St, Darlinghurst, Sydney, NSW 2010, Australia; Menzies Institute for Medical Research, University of Tasmania, 17 Liverpool Street, Hobart, Tasmania 7000, Australia; Centre for Eye Research Australia, University of Melbourne, Royal Victorian Eye and Ear Hospital, 32 Gisborne St, East Melbourne 3002, Australia

**Keywords:** glaucoma, CRISPR, single cell sequencing, cell painting, GWAS

## Abstract

**Introduction:**

Primary open angle glaucoma (POAG) is a leading cause of blindness globally. Characterized by progressive retinal ganglion cell degeneration, the precise pathogenesis remains unknown. Genome-wide association studies (GWAS) have uncovered many genetic variants associated with elevated intraocular pressure (IOP), one of the key risk factors for POAG. We aimed to identify genetic and morphological variation that can be attributed to trabecular meshwork cell (TMC) dysfunction and raised IOP in POAG.

**Methods:**

62 genes across 55 loci were knocked-out in a primary human TMC line. Each knockout group, including five non-targeting control groups, underwent single-cell RNA-sequencing (scRNA-seq) for differentially-expressed gene (DEG) analysis. Multiplexed fluorescence coupled with CellProfiler image analysis allowed for single-cell morphological profiling.

**Results:**

Many gene knockouts invoked DEGs relating to matrix metalloproteinases and interferon-induced proteins. We have prioritized genes at four loci of interest to identify gene knockouts that may contribute to the pathogenesis of POAG, including *ANGPTL2, LMX1B, CAV1,* and *KREMEN1*. Three genetic networks of gene knockouts with similar transcriptomic profiles were identified, suggesting a synergistic function in trabecular meshwork cell physiology. *TEK* knockout caused significant upregulation of nuclear granularity on morphological analysis, while knockout of *TRIOBP, TMCO1* and *PLEKHA7* increased granularity and intensity of actin and the cell-membrane.

**Conclusion:**

High-throughput analysis of cellular structure and function through multiplex fluorescent single-cell analysis and scRNA-seq assays enabled the direct study of genetic perturbations at the single-cell resolution. This work provides a framework for investigating the role of genes in the pathogenesis of glaucoma and heterogenous diseases with a strong genetic basis.

## Introduction

Glaucoma is a heterogeneous group of diseases leading to irreversible blindness with characteristic optic nerve damage. The most common glaucoma subtype is primary open-angle glaucoma (POAG) [1, 2]. Elevated intraocular pressure (IOP) is the only known modifiable risk factor and plays a major role in the progression of POAG [3]. The circulatory system maintains IOP in the anterior segment of the eye [4, 5]. Aqueous humor is produced by the ciliary body and passes through the pupil before draining out to the episcleral blood vessels via conventional or unconventional pathways [1, 6]. The conventional outflow pathway through the trabecular meshwork accounts for approximately 80% of total aqueous humor outflow. Structural alterations observed in the trabecular meshwork are considered to increase outflow resistance in POAG [5, 7, 8].

Many POAG-associated loci have been identified through genome-wide association studies (GWAS) [9], with loci encompassing Caveolin 1 and 2 (*CAV1/CAV2*), Transmembrane and coiled-coil domain-containing protein 1 (*TMCO1*), cyclin-dependent kinase inhibitor 2B antisense RNA 1 (*CDKN2B-AS1*), ATB binding cassette subfamily A member 1 (*ABCA1*), actin filament associated protein 1 (*AFAP1*), GDP-mannose 4,6-dehydratase (*GMDS*), Forkhead Box C1 (*FOXC1*), thioredoxin reductase 2 (*TXNRD2*), and Ataxin 2 (*ATXN2*) [10–13]. Furthermore, protein altering variants in genes such as *MYOC, LTBP2, FOXC1, GMDS* and *CYP1B1* have been found to cause both congenital and juvenile onset glaucoma. These particular variants are generally associated with abnormal development of the aqueous circulatory system and Schlemm’s canal rather than maintenance, however some are also involved in maintenance such as *TEK* [14–18].

More recently, a GWAS meta-analysis identified 85 novel SNPs associated with IOP using data from the UK Biobank, the International Glaucoma Genetic Consortium, and the Australian & New Zealand Registry of Advanced Glaucoma Cohort [19]. Novel gene variants, including *ANGPT1, ANKH, MECOM* and *ETS1* were associated with POAG and IOP. However, this study also identified SNPs at *ADAMTS6*, *MYOF, ANAPC1, GLIS3*, and *FNDC3B* that are associated with phenotypes such as central corneal thickness and corneal hysteresis [19]. This highlights potential confounding factors in GWAS that make identification of genes implicated in the pathogenesis of POAG challenging. Furthermore, various SNPs identified in IOP associated GWAS are associated with more than one gene, making it difficult to precisely implicate the disease-causative gene.

**Figure 1:**
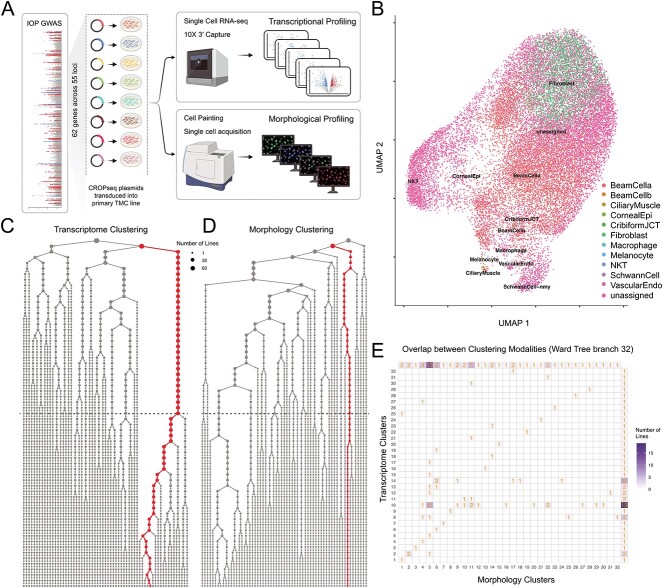
**CROP-seq interrogation of intraocular pressure associated loci.** (A) Schematic overview of the study, where primary human TMC lines were cultured, each with a single gene at an IOP-associated loci, knocked out. A further 5 cell lines were maintained as controls. All groups underwent transcriptional profiling and morphological profiling analysis. (B) Following single cell RNA sequencing CRISPR edited cells were compared to primary human TMCs. (C) Ward’s cluster tree displaying the hierarchical clustering of each cell line based on the single cell RNA expression profiles. The red dots display the cluster containing the non-targeting control lines. The dashed line represents cluster 32. (D) Ward’s cluster tree displaying the hierarchical clustering of each cell line based on the morphological profiles. The red dots display the cluster containing the non-targeting control lines. The dashed line represents cluster 32. (E) Agreement between clustering of cell lines based on morphology or expression profiles. The less distributed the knockout groups within each cluster are across clusters in the alternative approach, the higher the agreement between approaches. **Alt-text**: CROP-seq profiling of intraocular pressure associated genes using transcript and morphological features.

Recent advances in clustered regularly interspaced short palindromic repeats (CRISPR) and single-cell RNA sequencing (scRNA-seq) technology have allowed for high-throughput genetic screens at single-cell transcriptome resolution. In CRISPR droplet sequencing (CROP-seq), a guide-RNA (gRNA)-encoding vector makes gRNAs detectable in scRNA-seq, and as such, these gRNAs can be used to tag individual cells [20]. To investigate the role of POAG-associated loci in TMCs, we knocked out gene candidates in human TMC lines using CROP-seq. We then performed single-cell RNA sequencing as well as morphological profiling to identify the genotypic and phenotypic roles of each gene (Fig. 1A). The cell painting protocol involves cultured cells being stained with fluorescent dyes to reveal eight cellular substructures, thus allowing morphological features to be extracted from individual cells to display the effects of genetic perturbation [21, 22]. Morphological profiling can then be undertaken using CellProfiler, a high-throughput single-cell image analysis program designed to extract and analyze over one thousand phenotypic features. Taken together, this study screens gene candidates based on expression profiles and morphology profiles and helps understand the pathway in which these genes are involved in the causation of elevated IOP in TMCs.

## Results

### Data overview

A total of 134 gRNAs sequences were designed (124 gRNAs targeting 62 genes and 10 gRNAs for human non-targeting control; Supplementary Table 1), and after being cloned into a CROP-seq plasmid which also expressed SpCas9, were delivered to a primary TMC line in arrayed format. A total of 105 273 cells were captured with 25 879 (24.58%) cells passing our stringent quality control filtering to be included in transcriptional profiling.

To compare the similarity of cells to primary human TMCs we used the scPred package, an unbiased gene-marker free cell classification method [23]. As anticipated, our cultured cells were less heterogeneous than *in vivo* trabecular meshwork tissue (Supplementary Fig. 1). Beam A cells and non-myelinating Schwann cells were previously identified by van Zyl *et al*. as the most common cells (excluding Ciliary Muscle) in samples of human trabecular meshwork tissue [24]. The most commonly identified cell types in our data were Beam A cells and fibroblast-like cells, with non-myelinating Schwann cells contributing a smaller proportion (Fig. 1B and Supplementary Fig. 1). Our major cell types expressed key canonical genes (Supplementary Fig. 2). Importantly, Beam A cells expressed *BMP5*, fibroblast-like cells expressed *COL14A1* and *ANGPTL7*, while the non-myelinating Schwann cells expressed *SCN7A* (Supplementary Fig. 2).

From this analysis, we then sought to determine if the target gene knockout groups were unequally distributed throughout these predominant cell-types. Overall, cells from all gene knockout groups were assigned to each previously classified primary cell group (Supplementary Fig. 3).

Despite the fact that many CRISPR-edited gene transcripts would still be transcribed and thus captured using the 3′ RNAseq 10X technology, given presence of nonsense mediated decay, we sought to formally assess the knockout efficiency by comparing the targeted gene transcript in each knockout line to that of the non-targeting control cells. Overall, 25 of the target cell lines had lower on-target transcript counts compared to the controls at the Bonferroni corrected level (*P* < 0.0008; Supplementary Fig. 4, Supplementary Table 1). *ABO* and *TEX41* were found not to be expressed in our TMCs. We also investigated the concordance in transcriptome-wide profile of cells with different gRNAs targeting the same gene (Supplementary Table 2). Given that the lowest Pearson Correlation Coefficient was found to be 0.992, cells with each gRNA were combined for analysis (Supplementary Table 2). Canonical markers of the cell cycle were expressed in a similar pattern across all target gene knockout groups (Supplementary Fig. 5).

Differentially-expressed gene (DEG) analysis was then performed to investigate the effects of gene knockout at select IOP-associated loci. DEG analysis was performed for each gRNA individually against non-targeting control gRNAs (Supplementary Table 3). Potentially reflecting reduced power from the smaller number of cells profiled, or the fact that the cultures were transfected with the CRISPR construct at a relatively high multiplicity of infection (MOI), no distinct pattern in concordance of differentially expressed genes were observed between the individual gRNA and the combined gene-based knockout groupings (Supplementary Fig. 6). gRNA targeting the same target gene were combined for subsequent analyses.

The Euclidean distance of DEG expression between each knockout group and controls was computed to identify gene knockouts with similar expression patterns that may indicate novel gene networks involved in the pathogenesis of POAG. DEG analysis was also used to prioritize multi-gene loci to identify a pathological variant. Ward’s hierarchical clustering method was used to generate a cluster tree for further characterization of perturbed genes (Fig. 1C and Supplementary Fig. 7). This process of cell line-based clustering was then undertaken for the morphological features identified following Cell Painting and extracted using CellProfiler (Fig. 1D and Supplementary Fig. 8) [21, 25].

To investigate the similarity between clustering based on transcriptomic or morphology profiles we calculated the Rand index at branch 32 of the Ward tree. At this branch, where the non-targeting control cell line expression appeared to diverge most from the other gene knockout lines (Fig. 1C), the Rand index was 0.86, suggesting strong agreement between the two clustering approaches (Fig. 1E).

### scRNAseq clustering allows prioritization at multi-gene loci

**Figure 2:**
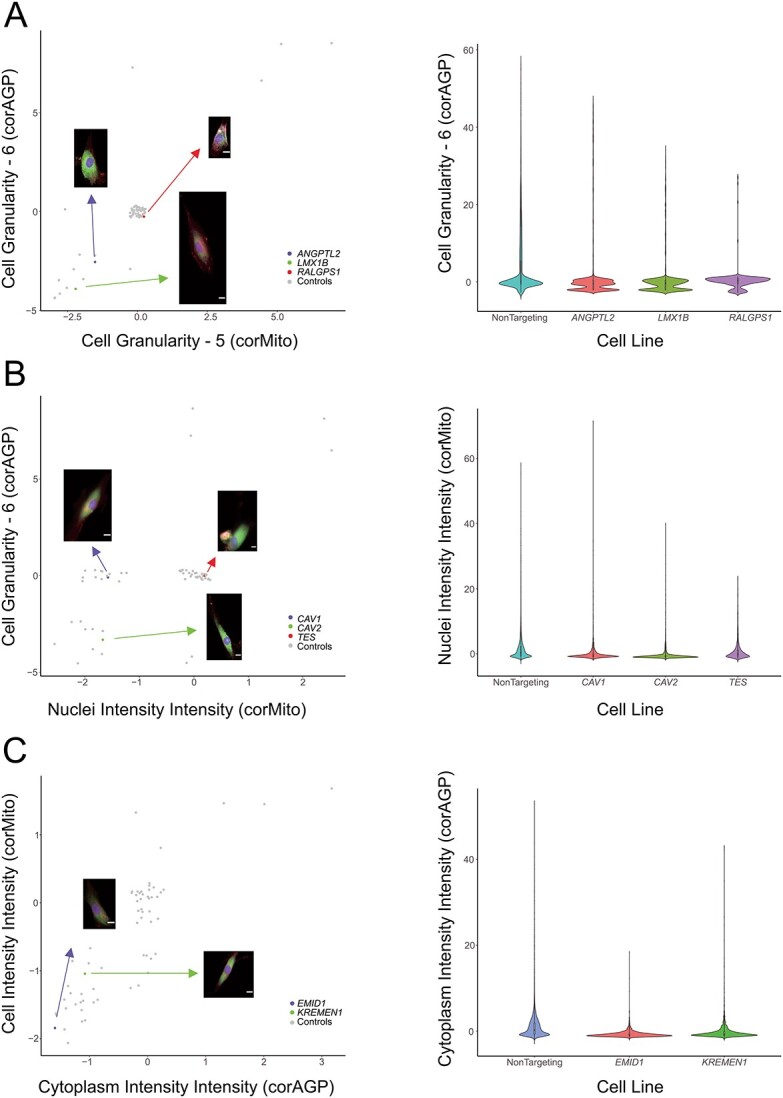
**Morphological features of selected genes at multi-gene loci. **(A) *ANGPTL2*, *LMX1B*, and *RALGPS1*; (B) *CAV1*, *CAV2*, and *TES*; (C) *EMID1*, and *KREMEN1*. Scatter plots of the mean value of each group of features with representative images (jitter was applied to avoid overplotting)(scale bar: 5 μm). **Alt-text**: Profiles for selected morphological features of genes from multi-gene loci.

DEG and morphological profiling analysis was used to prioritize the most likely pathological gene implicated at a locus containing many candidate genes (Table 1). We selected three genes chromosome 9q33 to knockout (*ANGPTL2, RALGPS1* and *LMX1B*). The *ANGPTL2* knockout cell line was shown to have the highest Euclidean distance (d = 18.79) as well as cluster independently from non-targeting control cells on the transcriptomic analysis (Supplementary Fig. 7). Furthermore, *ANGPTL2* also had the highest number of statistically significant DEGs (n = 23), which were primarily involved in interferon alpha/beta signaling. The *LMX1B* knockout group had the next highest Euclidean distance (14.06) and significant DEGs [11], which are primarily involved in regulating cell proliferation. *LMX1B* also clustered independently from the non-targeting control cell lines. Furthermore, the morphological profiling data revealed that *RALGPS1* had almost no significant changes in cellular morphology and was concordantly clustered with the non-targeting control cell line gene expression profiles. The *ANGPTL2* knockout, evoked a significant reduction of intensity and granularity in both the mitochondrial and actin/cell membrane channels, which was also observed in *LMX1B* knockout cells (Fig. 2A). Interestingly, the transcriptional start site for *ANGPTL2* is located further from the top IOP-associated SNP than *LMX1B* (Supplementary Fig. 9).

**Table 1:** Breakdown in number of differentially expressed genes from the individual knockout of genes at overlapping loci.

Multi-gene locus^#^	Overlapping genes	Dendrogram cluster at Branch 32	Transcriptional Euclidean distance from Control Lines	Significant DEGs compared to control (*P*-value < 10^−6^, log2 fold change > 2)^#^
chr1 bp:165714416	*ALDH9A1*	5	13.98	22
	*TMCO1*	12	12.96	7
chr9 bp:129367398	*ANGPTL2*	9	18.79	23
chr9 bp:129369971	*LMX1B*	22	14.06	11
chr9 bp:129373110	*RALGPS1*	10	7.1	4
chr9 bp:129863168				
chr7 bp:115810676	*CAV1*	2	8.93	4
chr7 bp:116151338	*CAV2*	10	6.19	3
	*TES*	10	5.57	2
chr11 bp:86406159	*ME3*	23	16.51	28
	*PRSS23*	28	18.03	28
chr22 bp:29620325	*EMID1*	10	7.29	4
	*KREMEN1*	13	11.51	5
chr22 bp:19860977	*GNB1L*	14	10.34	4
	*TXNRD2*	8	10.44	5

^#^base pair position on chromosome

At the chromosome 7q31 locus associated with variation in IOP, we selected three genes to knockout (*CAV1, CAV2,* and *TES*)*.* Cells with *CAV1* knocked out were found to transcriptionally cluster separately from the non-targeting control cells (Supplementary Fig. 7), and also had the highest number of statistically significant DEGs (Table 1). However, it is noteworthy that the *CAV2* knockout cells were found to significantly upregulate (6.23-fold increase, *P* = 2.06×10^−14^) the expression of *MYOC*, a gene whereby disease causing variants lead to elevated IOP and POAG [14, 26–29]. This could infer that the knockout of *CAV2* may induce cellular effects similar to *MYOC*, and this effect appeared to predominate in the Beam A cells (Supplementary Table 4). *CAV1* knockout resulted in a small reduction in mitochondrial intensity, while *CAV2* knockout produced a statistically significant reduction in the intensity and granularity of the mitochondrial and actin/cell membrane channels (Fig. 2B). CRISPR knockout of *TES* resulted in minimal morphological variation, with *TES* knockout cells clustering with the non-targeting control group (Fig. 2B and Supplementary Fig. 7).

We selected two genes (*EMID1* and *KREMEN1*) on chromosome 22q12 to knockout in our TMCs. Although the transcriptional start site for *EMID1* is closer to the top IOP-associated SNP (Supplementary Fig. 9), cells with *KREMEN1* knocked-out, but not *EM1D1* knockout cells, were found to transcriptionally cluster separately to the non-targeting controls (Supplementary Fig. 7). *KREMEN1* knockout cells were also found to have a greater number of DEGs, though these did not appear to act via a single pathway. Interestingly, knockout of *EMID1* resulted in intensity reduction in the mitochondrial and actin/cell membrane channels (Fig. 2C). The remaining multi-gene loci all clustered independently from the control group and had similar degrees of DEG expression, which makes it difficult to resolve the prioritized gene (Table 1).

### Key up- and downregulated DEGs of interest

DEG analysis revealed key genes with potential roles in the variation in IOP. Volcano plots of all gene knockouts across each cell type (*i.e.* similar to bulk RNA-seq) are displayed in Supplementary Fig. 10. Using the predicted cell-type from our scPred model, we also undertook DEG analysis on the subset of cells with the highest representation. Given the majority of cells were classified as Beam A cells or Fibroblast-like cells, differential gene expression was performed for these subsets. The results for this have been included in Supplementary Table 4, and the volcano plots from these analyses are displayed in Supplementary Figs 11 and 12.

We sought to identify direct patterns of expression across genes at different loci. In addition to a putative link between *CAV2* knockout and overexpression of *MYOC*, as outlined above, knockout of *ZNF280D* resulted in an over expression of *EMCN* in Beam A cells (fold change = 4.24, adjusted *P* = 5.22 × 10^−22^). In fibroblast-like TMCs, knockout of *ABCA1* led to an over expression of *ANGPT2* (fold change = 6.23, adjusted *P* = 1.25 × 10^−18^). Finally, knockout of *ADAMTS6*, *ALDH9A1*, *ARHGEF12*, *FBXO32*, *FER*, *FMNL2*, *GAS7*, *LMO7*, *ME3*, *MECOM*, *MYOF*, *PARD3B*, *PRSS23*, or *ZNF280D* result in reduced expression of *EFEMP1* in both Beam A cells or fibroblast like cells (*P* < 10^−6^). Murine expression of *EFEMP1* has been shown to interact with tissue inhibitor of metalloproteinase-3 (TIMP3), and may play a role in cell adhesion and migration [30].

**Figure 3:**
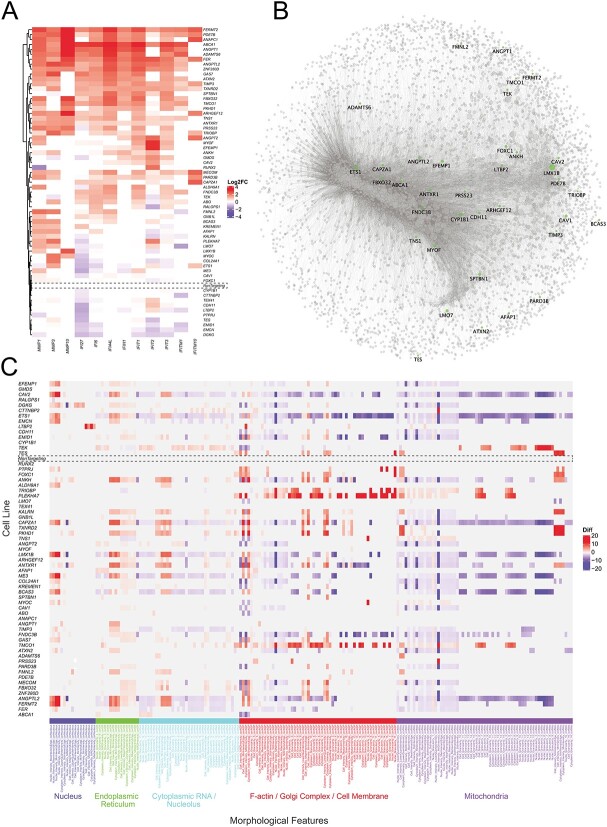
**Selected expression and morphological features in TMC knockout and control lines.** (A) Heatmap illustrating significant up-regulation of matrix metalloproteinases (*MMP1, MMP3, MMP10*) and interferon-related proteins (*IFI27, IFI6, IFI44L, IFIH1, IFIT1, IFIT2, IFIT3, IFITM1, IFITM10*). (B) Gene expression network in non-targeting control cells. A gene expression network for non-targeting control trabecular meshwork cells was generated to highlight target genes that are normally expressed in TMCs. Closer proximity between the genes indicate similar degrees of co-expression and the size of the node corresponds to the node centrality of PAGErank. Genes selected for knockout, found to have a Pearson correlation above 0.7 with another gene are displayed in green. (C) Heatmap displaying variation in TMC morphological features for knockout of genes at IOP-associated loci. Morphological features extracted by CellProfiler grouped by organelles of the same fluorescent channel are displayed. Features extracted are based on pixel intensities and calculations based on area and appearance. **Alt-text**: Selected transcript and morphological features in TMC control lines.

Matrix metalloproteinases previously associated with POAG were upregulated across several gene knockouts. MMP1 was statistically significantly (*P* < 10^−6^) upregulated in nine knockout cell lines (*ABCA1, ADAMTS6, ALDH9A1, ANAPC1, ANGPT1, FER, FERMT2, FMNL2, PDE7B*) with a fold-change ranging from 4.05–7.27. *MMP3* was similarly statistically significantly (*P* < 10^−6^) upregulated in seven knockout lines (*ADAMTS6, ANGPTL2, COL24A1, ETS1, MYOC, PDE7B, TRIOBP*) with a fold-change of 4.17–5.31 (Fig. 3A). Finally, MMP10 was upregulated in four knockout lines (*ADAMTS6, ANAPC1, FERMT2, PDE7B*) with a fold-change of 4.82–46.37 (*P* < 10^−6^) (Fig. 3A). The proteins encoded by these genes are part of a family of proteins involved in the breakdown of extracellular matrix in physiologic and pathologic processes. The matrix metalloproteinase family of proteins have also been previously implemented in TMC function and the pathogenesis of POAG, with upregulation of MMPs 1, 9, and 12 previously implicated in POAG [31–33].

Another group of highly upregulated DEGs were interferon-induced proteins, which are generally involved in antiviral immunity though appear to have a pleiotropic effect on IOP regulation in TMCs. *IFI44L* was statistically significantly (*P* < 10^−6^) upregulated in nine knockout lines (*ABCA1, ADAMTS6, ANAPC1, ANGPT1, FER, FERMT2, PDE7B, TMCO1, ZNF280D*) across a fold-change range of 4.39–10.39. Similarly, *IFIT1* was also upregulated in seven knockout cell lines (*ABCA1, ADAMTS6, ANAPC1, ANGPT1, FER, FERMT2, ZNF280D*) with a fold-change ranging from 4.31 to 7.30 (*P* < 10^−6^). *IL1RN* was also statistically significantly (*P* < 10^−6^) upregulated with a fold-change across 4.03–12.68 among 14 knockout lines (*ABCA1, ADAMTS6, ANGPT1, ANGPTL2 FBXO32, FER, FERMT2, FMNL2, MYOC, PARD3B, PDE7B, PRSS23, TIMP3, ZNF280D*) (Fig. 3A).

### Identification of putative genetic networks involved in the pathogenesis of POAG

To investigate gene-based networks we initially mapped the expression profile from the non-targeting controls cells, highlighting all genes selected for targeted knockout (Fig. 3B). This gene regulatory network was generated by retaining the most significant correlations, to generate as many edges as possible, with only Pearson correlations above 0.7 [34]. All but two genes were found to be expressed in the control cells, as outlined previously, yet 30 (58%) of the 62 target genes were found to be above this Pearson correlation threshold (Fig. 3B).

We next compared the DEG profile of knockout groups found to cluster together at the branch where the non-targeting control cell line expression appeared to diverge most from the other gene knockout lines (branch 32 of the Ward tree, Fig. 1C). One cluster contained three genes: *ABO, CAV1,* as well as *MYOC*, and targeted knockout led to upregulation of *TAC1* and *LCE1C*. *TAC1* is a potent vasodilator, and has previously been shown to be upregulated in specific *MYOC* mutations [35]. Conversely, *LCE1C*, which is predicted to be involved in keratinization and has not previously been implicated in TMC homeostasis. The *MYOC* knockout also induced upregulation of *SPP1*, which has been found to be highly expressed during trabecular meshwork differentiation [36].

Cells with *ANGPT2, PKHD1, TNS1,* or *TXNRD2* knocked-out were found to cluster together. There were five statistically significantly (*P* < 10^−6^) upregulated DEGs (*CXCL11, CST1, LCE1C, OASL, CD70*) and three downregulated DEGs (*STEAP4, CCN5, C1orf87)*; however, these genes have not previously been found to cause TMC dysfunction or POAG. Knockout of *CAPZA1, KALRN, LMO7, PLEKHA7, GNB1L,* or *TEX41* resulted in a similar DEG profile. There were two key DEGs identified on bulk analysis of this cluster, *MT1G* (upregulated) and *STEAP4* (downregulated); however, there was no previous association with POAG among these DEGs. Knockout of *ATXN2*, *FBXO32* or *PRSS23*, resulted in an over expression of *ANGPTL4* in fibroblast like cells (fold change range 4.12–4.83; adjusted *P* < 1.01E-24), which is noteworthy given that *ANGPT2,* which encodes for angiopoietin, is critically involved in the development of Schlemm’s canal, with monogenic variants known cause congenital glaucoma through elevated IOP [37–39].

### Key morphological features

**Figure 4:**
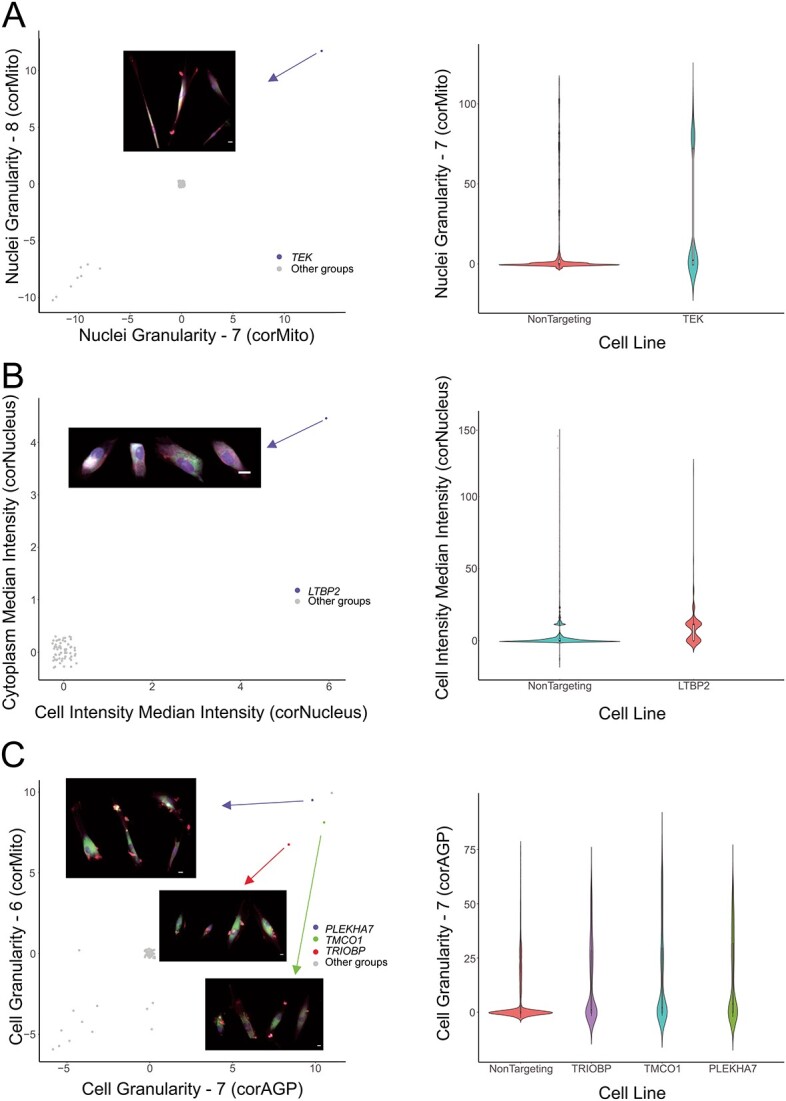
**Morphological features of key gene knockout groups.** (A) *TEK*; (B) *LTBP2*; (C) *PLEKHA7*, *TMCO1*, and *TRIOBP*. Panels display the scatter plots of the mean value of each group of features (jitter was applied to avoid overplotting) (scale bar: 5 μm). **Alt-text**: Profiles for selected morphological features of key gene knockout groups.

A heatmap was constructed showing cell lines with a difference of >1.5 or <−1.5 compared to non-targeting controls (*P*-value < 10^−40^) (Fig. 3C). This identified key genes of interest that had particular morphological changes. The *TEK* knockout group particularly showed an increase in the outputs referring to nuclear granularity identified with mitochondrial stain (Fig. 4A). The *LTBP2* knockout was the only group to show a significant increase in the mean nuclear intensity as well as the standard deviation nuclear intensity (Fig. 4B). Finally, *TRIOBP* and *TMCO1* both showed similar increases in actin/cell membrane granularity and intensity which was greater than any other groups; *TRIOBP* and *PLEKHA7* also demonstrated similar morphological profiles with similar degrees of feature increase across mitochondrial and actin/cell membrane channels (Fig. 4C).

## Discussion

GWAS have uncovered a large range of novel loci associated with many complex traits [40–43]. With such significant amounts of data generated from these studies, the challenge is posed as to how to efficiently identify the most relevant gene(s) at each implicated locus [44]. A thorough understanding of the disease is required to identify new pathological pathways and thus; new therapeutic interventions. This study sought to investigate the effects of IOP-associated gene knockouts on the morphological and transcriptome profiles of primary human TMCs. Applied in the context of POAG, this study aimed to identify genes associated with IOP to highlight potential TMC dysfunction with the goal of distinguishing new therapeutic pathways for drug discovery. We conservatively estimate that at least a fifth of the loci identified as influencing variation in IOP act through transcriptional perturbation of cells which constitute the majority of the trabecular meshwork (Beam A cells, Fibroblasts and non-myelinating Schwann cells). It is likely that genes at the remaining loci modulate cells in other tissue such as the ciliary body, Schlemm’s canal or the uveoscleral outflow. Future work exploring this in other cell types will hopefully also lead to new molecular insights into the direct pathogenesis of POAG.

In the gene knockout groups with genes related to congenital glaucoma, *GMDS, FOXC1, LTBP2, TEK,* and *CYP1B1,* no distinct patterns were observed in the transcriptome of these gene knockout groups. Similarly, the morphological profiles of these gene knockout groups demonstrated minimal change from non-targeting control groups. This supports the premise that these genes are more involved in trabecular meshwork embryogenous and development rather than ongoing cellular homeostasis. Previous work has shown that *CYP1B1* and *LTBP2* are also involved in modulating ocular development with disease causing variants resulting in abnormal development of the trabecular meshwork and anterior ocular circulation [45, 46]. Furthermore, *FOXC1* is known to be primarily involved in the development of the trabecular meshwork with disease causing variants leading to anterior segment dysgenesis and elevated IOP [47]. In addition to this, disease causing variants in these genes are often gain-of-function and as such may not exhibit a pathological response in knockout experiments. For example, *MYOC* mutations are typically gain-of-function resulting in protein misfolding inducing endoplasmic reticulum stress and extracellular matrix dysfunction [48].

This work highlights the power of using high throughput single cell profiling to resolve the likely causative gene at complex disease-associated loci identified via GWAS; a known challenge of GWAS-based drug discovery [41]. We were able to prioritize four genes at seven of the “multi-gene loci” studied. *ANGPTL2, LMX1B,* and *EMID1* were found to have a greater transcriptomic and morphological variation from non-targeting control cell lines than other genes at their corresponding loci, and appear most likely to contribute to the regulation of IOP and pathogenesis of POAG. Interestingly, although knockout of *CAV1* resulted in a greater number of DEGs than knockout of *CAV2*, *CAV2* knockout caused up-regulation of *MYOC*.

Hierarchical clustering was used to identify potential genetic networks of similar genes contributing to IOP physiology. Three clusters containing between three and five distinct gene knockouts produced similar DEG patterns indicating a potential interaction between these genes and thus; a genetic network contributing to IOP physiology and the pathogenesis of POAG. When analyzing individual DEG expression across gene knockouts, it was noted that genes related to matrix metalloproteinases and interferon-related proteins were significantly up- or down-regulated. Matrix metalloproteinases have a distinct footprint of evidence showing a role in the pathogenesis of POAG [31–33, 49, 50]. However, interferon-alpha and interferon-induced proteins have minimal previous associations with POAG potentially highlighting this as a novel pathological pathway in disease progression. Of note, *IFIH1* was the only interferon-related gene identified in DEG analysis which has been associated with glaucoma in literature. Mutations in *IFIH1* have been associated with Aicardi-Goutières syndrome and Singleton-Merten syndrome, both of which have similar overlapping features and are associated with glaucoma [51–56].

One of the limitations of this study is that only trabecular meshwork cells have been investigated *ex vivo*, and there are several other ocular cells implicated in regulation of IOP and POAG (such as the ciliary body, uveoscleral outflow, and Schlemm’s canal) [5]. Further work applying our novel CROP-seq constructs could be conducted to directly investigate these cells.

It is also important to note the variability in knockdown efficiency across gRNA as detected by 3′ scRNA-seq (Supplementary Table 1). This could reflect poor on-target efficacy *in vivo* of some gRNA, differing effects of gene domains being targeted, or even variability in lentiviral processing and nonsense mediated decay across TMCs. Interestingly, there was not a distinct pattern in the overlap in differentially expressed genes for each individual gRNA or combined gRNA, when the expression of the target gene was significantly reduced (Supplementary Fig. 6). We hypothesize that robust gene knockdown in a scRNA-seq screen would be better observed using CRISPR interference. A further limitation of this study is that the CRISPR gene knockout results in unpredictable effects on gene function and gene-network perturbations [57, 58]. CRISPR-mediated gene knockout may not actually recapitulate the molecular pathogenesis in POAG. For example, the loci found to influence variation in IOP may act through a variety of mechanisms from gain-of-function through to gene silencing. That said, the allelic effect size a specific locus confers on disease development, does not necessarily mirror the therapeutic potential of targeting that gene or pathway. For example, in the case of neovascular age-related macular degeneration, the genetic contribution of *vascular endothelial growth factor A* locus is relatively small, yet VEGF monoclonal antibodies have been remarkably successful at controlling disease [59–61]. Finally, although we knocked out a single gene to investigate its effect on the pathogenesis of POAG and IOP regulation, disease processes are often contributed to by a network of genes functioning in unison indicating that the knockout of a single gene may be insufficient to reproduce the complete disease phenotype [62]. This is further highlighted by the role of polygenic risk scores in POAG indicating that future studies may investigate knockout of arrays of genes as a method of quantifying the transcriptional profiles of polygenic genetic variations [63–65].

In summary, this work is the first time that high-throughput multiplex morphological profiling has been combined with scRNA-seq analysis. Together, these platforms have uncovered unifying pathways involved in the homeostasis of TMCs, variation in IOP, and the pathogenesis of glaucoma. Robust pipelines have been generated to create transcriptomic and cell morphology profiles. These results demonstrate that gene perturbation can be reflected in the cell morphology with corresponding regulatory pathways, and as a consequence, this resource further improves our understanding of gene function in disease. This comprehensive transcriptomic and morphological dataset of trabecular meshwork cells represent the largest functional follow-up of genes implicated through GWAS to date. In the gene expression comparison, different cell types may be grouped according to their transcriptome patterns [24], and the influence of the non-normal distributions and outliers may be minimized. For the cell morphology, using the median value of each feature, and adding features’ dispersion and covariances to the profiles may increase the hit rates and reliability in finding positive genes related to the disease.

## Materials and methods

### Cell culture

Primary human TMCs were isolated from donors through the Lions Eye Bank at the Royal Victorian Eye and Ear Hospital (ethics approval reference: 13-1151H) before being cryopreserved and delivered frozen to the Menzies Research Institute. TMCs were thawed and cultured in Dulbecco’s Modified Eagle Medium (DMEM, Gibco, 11965118) with 10% fetal bovine serum (FBS, Gibco, 16000044), and supplemented with 0.5% antibiotic-antimycotic (Gibco, 15240-062). The culture medium was changed as per local protocol after 72 h or when cells reached 80% confluence. All cell lines were cultured at 37°C with 5% CO_2_ in the incubator. Each fortnight cell lines were tested for mycoplasma using the PCR Mycoplasma Test Kit (PromoKine, PK-CA91-1096).

### Cloning and validation of the single-vector CROPseq system

To generate a single-vector system CROPseq plasmid expressing both SpCas9 and sgRNA(CROPseq-EFS-SpCas9-P2A-EGFP; Addgene #99248), the EF1a promoter in the CROPseq-Guide-Puro 124 (Addgene plasmid # 86708) was replaced with the EFS promoter to drive the expression of SpCas9 using the Gibson Assembly method (NEBuilder HiFi DNA Assembly master mix). The EFS-SpCas9-P2A fragment was amplified from lentiCRISPRv2 (Addgene plasmid # 52961) using Q5 high-fidelity DNA polymerase. The puromycin resistance gene was then subsequently replaced with EGFP using an amplified fragment from the pMLS-SV40-EGFP plasmid (Addgene plasmid # 46919). The expression and activity of the single-vector CROPseq plasmid was tested by cloning in a sgRNA targeting the *DNMT3B* (sgRNA sequence: CAGGATTGGGGGCGAGTCGG) or LacZ control gene (sgRNA sequence: TGCGAATACGCCCACGCGAT) using Gibson Assembly method and transformed into NEBStable bacteria (NEB) as outlined by Datlinger and colleagues [20], and tested in HEK293A cells (Life Technologies). EGFP expression was visualized using the Eclipse Ti-E inverted fluorescence microscope (Nikon). The cleavage activity of the SpCas9 was measured through the indel formation using SURVEYOR assay (Integrated DNA Technologies). Briefly, genomic DNA was extracted (QIAamp DNA mini kit; Qiagen) from HEK293A cells transfected with CROPseq-EFS-SpCas9-P2A-EGFP DNMT3B sgRNA plasmid using Fugene HD (Promega). PCR fragment for SURVEYOR assay was amplified using Q5 high-fidelity polymerase using the primers F: 5′-CAAGAGCATCACCCTAAGAATGC-3′ and R: 5′-GTTGTCAGAGACTCTCCCCAAAG-3′ from Datlinger *et al*. [20] Q5 PCR conditions were as per the manufacturer’s protocol with the following thermocycling conditions: 98°C 30 s; 35 cycles of 98°C 10 s, 71°C 30 s, 72°C 15 s; 72°C 2 min. PCR products were gel purified using the QIAquick gel extraction kit (Qiagen). 200 ng of purified PCR product was used in the SURVEYOR assay as outlined in the manufacturer’s protocol.

### Confirmation of sgRNA sequence via sanger sequencing

In total, 134 sgRNAs sequences were designed to generate the 67 trabecular meshwork cell lines (124 sgRNAs for 62 genes and 10 sgRNAs for human non-targeting control, 2 sgRNAs for each cell line) (Supplementary Table 1). Each of the sgRNAs was cloned into CROPseq-Guide-pEFS-SpCas9-p2a-puro backbone (Addgene: #99248). The sequences of all sgRNAs templates were confirmed by in-house Sanger sequencing. Firstly, each template was amplified by the BigDye Terminator Cycle v3.1 Sequencing kit (Applied Biosystems, 4337454). The 10 μl reaction system contained 1 μl template, 1 μl 10 μM primer, 0.25 μl Reaction Mix, 1.75 μl 5X Sequencing Buffer, and 6 μl nuclease-free water. Cycling was performed using the following program: initial polymerase activation for 1 min at 96°C and 25 cycles of amplification (denaturation for 10 s at 96°C, annealing for 5 s at 50°C, and extension for 4 min at 60°C), then held at 15°C. Samples were purified with the CleanSEQ kit (Beckman Coulter, A29151) following the Agencourt CleanSEQ Dye-Terminator Removal protocol. Briefly, 10 μl of vortexed CleanSEQ reagent and 42 μl of 85% ethanol was added to each 10 μl sample and gently mixed. The sample was placed on the 96-well magnetic plate for 3–5 min until the magnetic beads formed a ring and the solution was clear. The supernatant was removed and samples were washed twice with 100 μl 85% ethanol with 30 s of incubation and then air dried for five minutes. Lastly, 30 μl nuclease-free water was added to each sample and incubated for 3–5 min on the magnetic tray to elute the purified DNA. Next, 15 μl of purified cycle sequencing product was added to the sequencing plate, then denatured by incubating at 95°C for 5 min. Sequencing Genetic Analyzer 3500, Applied Biosystems) was undertaken using the default program for 850 bp DNA length. Finally, the online alignment tool MAFFT (version 7) was used to confirm whether the sequences of all the 134 sgRNAs were matched with reference sequences.

### Cell transfection

The primary TMCs were transfected with 50 μl of lentiviral plasmid and 450 μl of TMCs culture medium mixed with 1:100 lentiblast (OZ Bioscience, LB01500). Each well was seeded with approximately 3.0×10^4^ cells. Cell cultures were incubated for 72 h before 1 μg/ml puromycin selection occurred over four days. Then the primary TMCs were growing in regular TMC culture medium for one week of recovery.

### Single-cell RNA sequencing

The cells were recovered in culture medium and single-cell capture was performed at the Garvan-Weizmann Centre for Cellular Genomics. Single-cell suspensions from different wells were pooled, centrifuged and resuspended in DPBS containing 1% BSA (Sigma-Aldrich, A8806-5G), and filtered by 37 μm strainer (STEMCELL, 27215). The estimated number of cells in each well in the Chromium chip was optimized to capture about 16 000 cells. The Chromium library was then generated following the protocol of the Chromium Single Cell 3′ v2 Library (10× Genomics). Briefly, individual cells were allocated into nanoliter-scale Gel Bead-in-EMulsions, in which the bead carries the primers containing a read 1 primer sequence, a 16 nt 10× barcode, a 10 nt Unique Molecular Identifier, and a poly-dT primer sequence. A barcoded, full-length cDNA was produced from each poly-adenylated mRNA after incubation with the Gel Bead-in-EMulsions. llcDNAs were pooled and amplified by PCR. In the library construction, P5, P7, a sample index, and read 2 primer sequences were added to each of the cDNA by End Repair, A-tailing, Adaptor Ligation, and PCR. The region of P5 and P7 allowed the library fragment to attach to the flow cell surface during the sequencing. Read 1 and read 2 sequences are standard Illumina sequencing primer sites used in paired-end sequencing. Then part of the library samples were sequenced on an Illumina NovaSeq 6000 system using the S4 flowcell with a read depth of 16 785 reads per cell resulting in a mean number of RNA features of 4195 per cell. Following this, the cell UMI-sgRNA sequence in the NGS library was also amplified and sequenced on an Illumina MiSeq-based sequencing.

### Cell painting immunohistochemistry

For each group, 4.0 × 10^3^ puromycin-selected TMCs were seeded to 96-well plates by fluorescence-activated cell sorting (FACS) via a Beckman Coulter MoFlo Astrios EQ with three replicates of each knockout group allocated at random. The whole experiment was performed in three batches of TMCs, thus, nine wells of cells were captured for each gene knockout group. The plate layout can be assessed on GitHub. TMCs were stained and fixed 48 h after FACS following the CellPainting protocol [21, 22]. TMCs were washed three times with HBSS without final aspiration and then sealed with parafilm. All 96-well plates were kept at 4°C in the dark before imaging.

### Automated image acquisition

Images were captured at 20× magnification in Phase Gradient Contrast (PGC), and five fluorescent channels, DAPI (385/465 nm), AF488(470/517 nm), AF514 (511/543 nm), AF594 (590/618 nm), AF647 (625/668 nm) on ZEISS Celldiscoverer 7 system. In each well, 25 sites were imaged, with autofocus in the DAPI channel as the reference.

### Morphological image feature extraction

CellProfiler (Version 3.1.9) was used to locate and segment the cells for single-cell feature extraction [25]. The pipelines in CellProfiler were set up to correct uneven illumination, flag aberrant images and identify the nuclei from DAPI channel and the entire cell from AF594 channel, then measure the features of the size, shape, texture, intensity, and the local density of the nuclei, cell and cytoplasm.

### Establishing the CellProfiler pipeline

The CellProfiler image processing pipeline consists of three parts; illumination correction, quality control and image analysis. The illumination correction pipeline begins by improving fluorescence intensity measurement followed by the quality control pipeline to identify and exclude aberrant images such as unfocussed images and debris. To identify cell components, the nucleus was defined as the primary object with the cell body defined as the secondary object, and the cytoplasm as the tertiary object. Subsequently, the features of size, shape, granularity, colocalization, local density, and textures were measured, and the data was saved in an SQLite database. Image analysis was carried out on a Nectar (The National eResearch Collaboration Tools and Resources project) Cloud workstation instance.

### Data curation and analysis

Data preparation was performed using R (Version 3.6.3) as described by Caicedo *et al*. [66], which included feature transformation, normalization and batch-effect correction. Firstly, all the negative controls were selected to explore the distribution of the features and the batch effects. Two transformation methods were applied, generalized logarithmic function [67] and Box-Cox transformation [68]. To avoid nonpositive values, generalized logarithmic function used a shrinkage strategy while Box-Cox transformation used a shift strategy [66]. The Anderson-Darling test was performed to evaluate the normality of each feature [69]. Next, the value of each feature was normalized by subtracting the median value of each feature from the control group and dividing by the corresponding median absolute deviation (MAD) *1.4826 in each plate, respectively. The single-cell data was aggregated by the median value of each well to create profiles of each replicate. The Spearman’s correlation was calculated for all replicates within a plate and across different plates. The replicates are selected with Spearman’s correlation score >0.2.

### Computational analysis of single cell sequencing data

We used the scPred package to train a cell-type classification model on single cell RNA-seq data from human trabecular meshwork tissue [23, 24], and applied it to our dataset to investigate the projected cell-type distribution of our cells against primary TMC. All gene knockout groups underwent hierarchical clustering and were plotted as a cluster tree. The optimal number of clusters was determined by the silhouette method. To annotate each of the clusters, the top features and tail features were extracted. The library was mapped to the GRCH38 *Homo sapiens* genome, and the resulting mapped counts between all samples were depth-equalized via the cellranger aggr pipeline. Each gRNAs was assigned to a respective cell, and cells were dropped from further analysis if no gRNA was identified, or if more than 1 gRNA was mapped. Then the scRNA-seq data was loaded into R via the Seurat package (Version 3.0), and SCTransform function was used to normalize the data. All cells passing QC and targeted by a single sgRNAs were visualized in a uniform manifold approximation and projection (UMAP) plot and were clustered with the Louvain method. The differentially expressed genes (DEGs) of each gene knockout group were selected with log2 fold change >2 compared to the human non-targeting controls. Then a hierarchical clustering was performed on the subset of all DEGs of all gene knockout groups. The optimal number of clusters was determined by the silhouette method. DEGs to the human non-targeting controls were selected to present each cluster.

To investigate the similarity between clustering based on transcriptomic or morphological profiles we calculated the Rand index, which is a measure of similarity between two data clusterings and represents the probability that the two clusterings will agree on a randomly selected pair of data points. Branch 32 of the Ward tree was selected as this is the branch where the non-targeting control cell lines diverged most from the gene knockout lines in the transcriptome analysis.

## Supplementary Material

HMG-2023-CE-00657_R1_Supplementary_Figures_ddae003

HMG-2023-CE-00657_SupplementaryTable1_ddae003

HMG-2023-CE-00657_SupplementaryTable2_ddae003

SupplementaryTable3_ddae003

SupplementaryTable4_ddae003

## Data Availability

Single-cell RNA sequencing and single-cell imaging data is available at the European Bioimage Archive (Accession Numbers: S-BSST840 & S-BSST841 respectively). GitHub: https://github.com/PeterLu0403/CROP_seq_Cellpainting and https://github.com/powellgenomicslab/CROP-seq
