# Supplementary material for: High throughput functional profiling of genes at intraocular pressure loci reveals distinct networks for glaucoma
Source: Hum Mol Genet. 2024 Jan 25;33(9):739–51. doi: 10.1093/hmg/ddae003 (PMC11031357; doi:10.1093/hmg/ddae003)
Supplement: HMG-2023-CE-00657_R1_Supplementary_Figures_ddae003 [file hmg-2023-ce-00657_r1_supplementary_figures_ddae003.pdf]

High throughput functional profiling of genes at intraocular pressure loci reveals distinct networks for glaucoma

SUPPLEMENTARY CONTENTS:

Supplementary Figure 1: **3**
Individual cell subtypes identified using scPred following comparison of our captured TMCs to a previously reported single cell RNAseq dataset generated from adult human trabecular meshwork. Related to Figure 1.

Supplementary Figure 2: **4**
Expression of canonical genes in the predominant cell subtypes identified using scPred. Related to Figure 1.

Supplementary Figure 3: **5**
Distribution of each knockout cell line to the projected TMC types captured. Cell classification projection was based on a previously reported single cell RNAseq dataset generated from adult human trabecular meshwork. Related to Figure 1.

Supplementary Figure 4: **6**
Comparison of target gene expression levels between each target knockout cell line and non-targeting control cells. ABO and TEX41 were found not to be expressed in our TMCs. P-values are displayed from the Student t-test.

Supplementary Figure 5: **7**
Expression of cell cycle genes across cells where target gene or non-targeting control gRNAs have been transfected.

Supplementary Figure 6: **8**
Overlap in differentially expressed genes for each individual gRNA and combined gene-based gRNA analysis across all cell types. Each Venn diagram depicts the number of statistically significant differentially expressed genes, with the component with the highest number in each set highlighted in red. The gRNA or combined gRNA analysis (as depicted by the target gene name) are underlined when the target gene expression levels was significantly reduced (P<0.05) compared to the non-targeting control cells.

Supplementary Figure 7: **10**
Cluster tree displaying the hierarchical clustering of each cell line based on the single cell RNAseq expression profiles. Here, the distance between each group within one branch is closer than those located in different branches. The bottom grid shows the genes within the same locus at the chromosome (black), genes associated with congenital or juvenile glaucoma are (blue), or the non-targeting control cells (red). The number of circles in each row represents the number of clusters found with the respective resolution parameter, with the circle's label and area denoting the number of groups in the cluster. The arrow widths and labels denote what proportion of the cells in each cluster came from the clusters at the previous resolution parameter. Related to Figure 1C.

Supplementary Figure 8: **12**
Cluster tree displaying the hierarchical clustering of each cell line based on the single cell morphological profiles. Here, the distance between each group within one branch is closer than those located in different branches. The bottom grid shows the genes within the same locus at the chromosome (black), genes associated with congenital or juvenile glaucoma are (blue), or the non-targeting control cells (red). The number of circles in each row represents the number of clusters found with the respective resolution parameter, with the circle's label and area denoting the number of groups in the cluster. The arrow widths and labels denote what proportion of the cells in each cluster came from the clusters at the previous resolution parameter. Related to Figure 1D.

Supplementary Figure 9: **14**
Scatter plots displaying the SNPs identified in the GWAS conducted by MacGregor et al., 2018 at each multi-gene-loci of interest along with human TMC line sets of transcription start site (TSS) peaks identified by the FANTOM5 project.

Supplementary Figure 10: **18**
Bulk analysis of differential expressed genes between gene knockout groups compared to non-targeting control groups. Cigar and volcano plots comparing the Log2 expression, and the Log2 fold change versus -log10(P value), respectively, for the gene-targeting cells and control non-targeting cells.

Supplementary Figure 11: **49**
Differential expressed genes between gene knockout and non-targeting control Beam A Cells. The volcano plots display the Log2 fold change versus -log10(P value) for the gene-targeted cells and control non-targeting cells.

Supplementary Figure 12: **77**
Differential expressed genes between gene knockout and non-targeting control Fibroblast-like Cells. The volcano plots display the Log2 fold change versus -log10(P value) for the gene-targeting cells and control non-targeting cells.

Supplementary Table 1:
CRISPR/Cas9 sgRNAs designed to target genes at loci previously associated with variation in intraocular pressure.

Supplementary Table 2:
Transcriptome-wide correlation between different CRISPR/Cas9 sgRNAs with identical target genes.

Supplementary Table 3:
Statistically significantly differentially expressed genes for each gRNA compared to non-targeting controls. Differential gene expression was performed in bulk, as well as on the subset of Beam A or Fibroblast-like cells (the subset of cells found to have the highest representation).

Supplementary Table 4:
Statistically significantly differentially expressed genes for each target gene compared to non-targeting controls. Differential gene expression was performed in bulk, as well as on the subset of Beam A or Fibroblast-like cells (the subset of cells found to have the highest representation).

Supplementary Figure 1:

Individual cell subtypes identified using scPred following comparison of our captured TMCs to a previously reported single cell RNAseq dataset generated from adult human trabecular meshwork. Related to Figure 1.

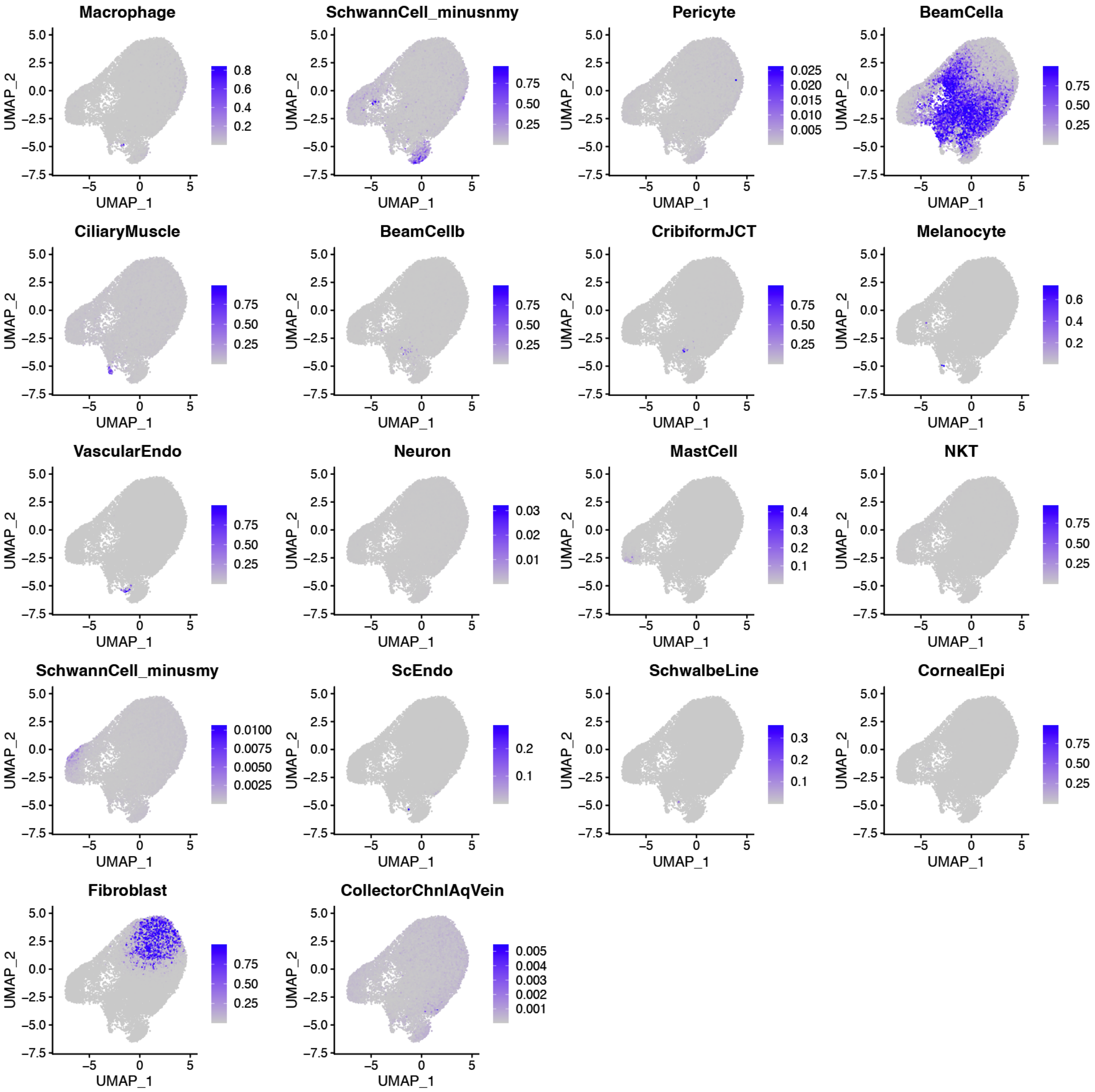

Supplementary Figure 2:

Expression of canonical genes in the predominant cell subtypes identified using scPred. Related to Figure 1.

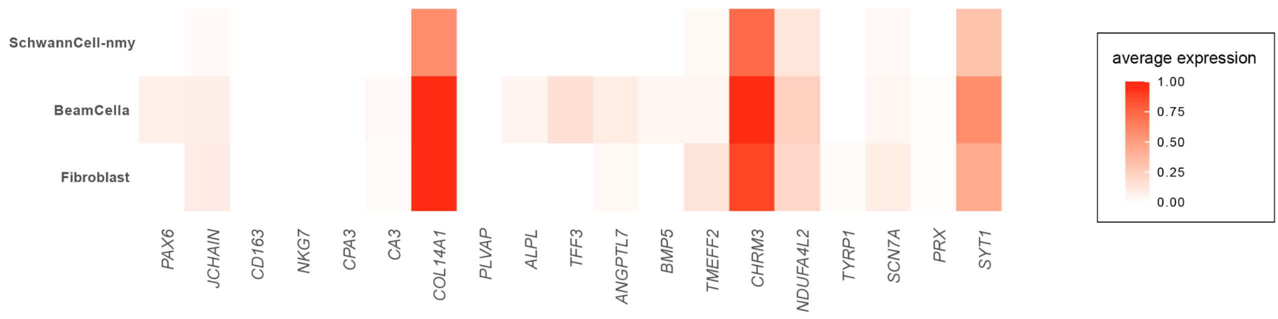

Supplementary Figure 3:
Distribution of each knockout cell line to the projected TMC types captured. Cell classification projection was based on a previously reported single cell RNAseq dataset generated from adult human trabecular meshwork. Related to Figure 1.

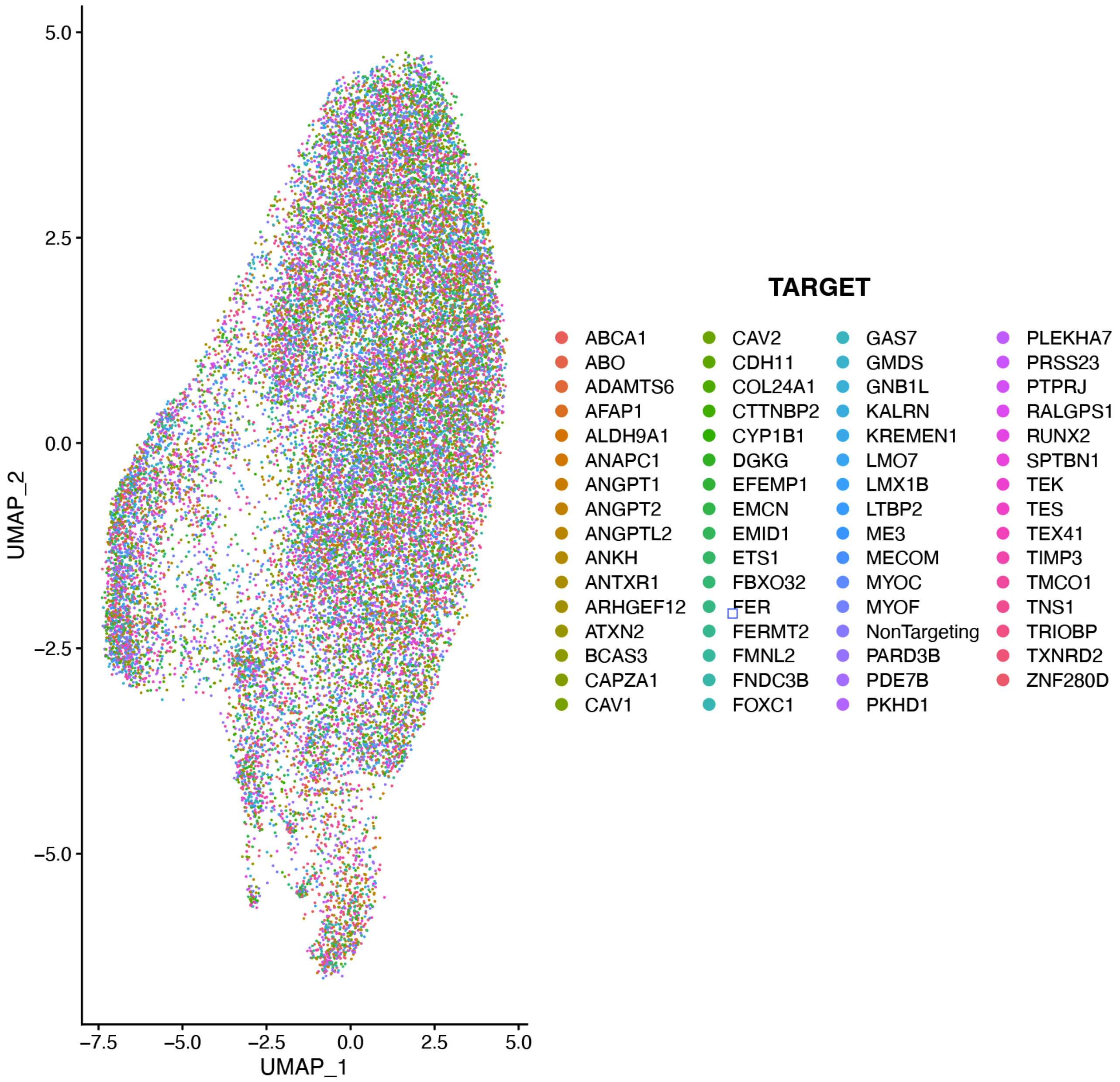

Supplementary Figure 4:

Comparison of target gene expression levels between each target knockout cell line and non-targeting control cells. ABO and TEX41 were found not to be expressed in our TMCs. P-values are displayed from the Student t-test.

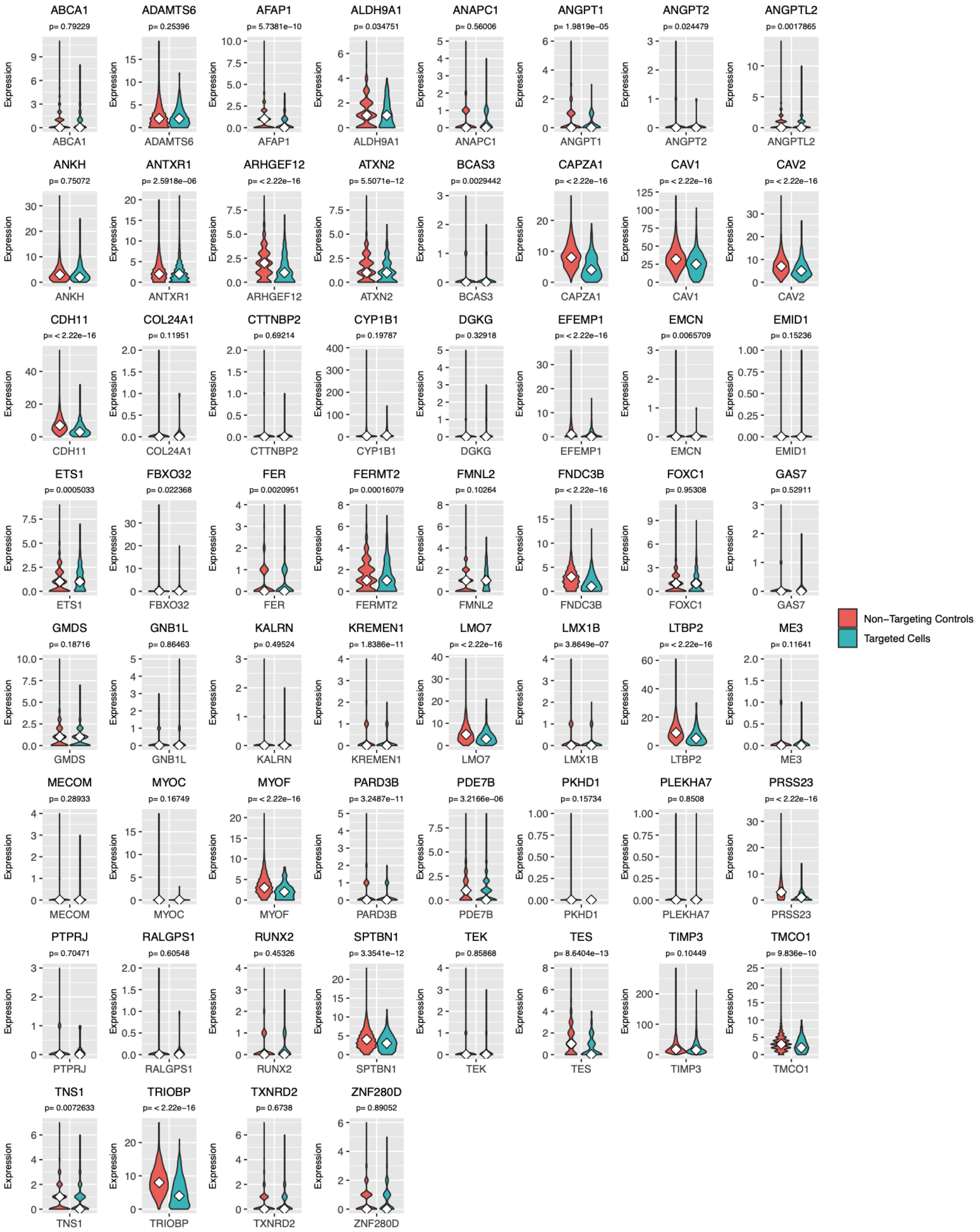

Supplementary Figure 5:

Expression of cell cycle genes across cells where target gene or non-targeting control gRNAs have been transfected.

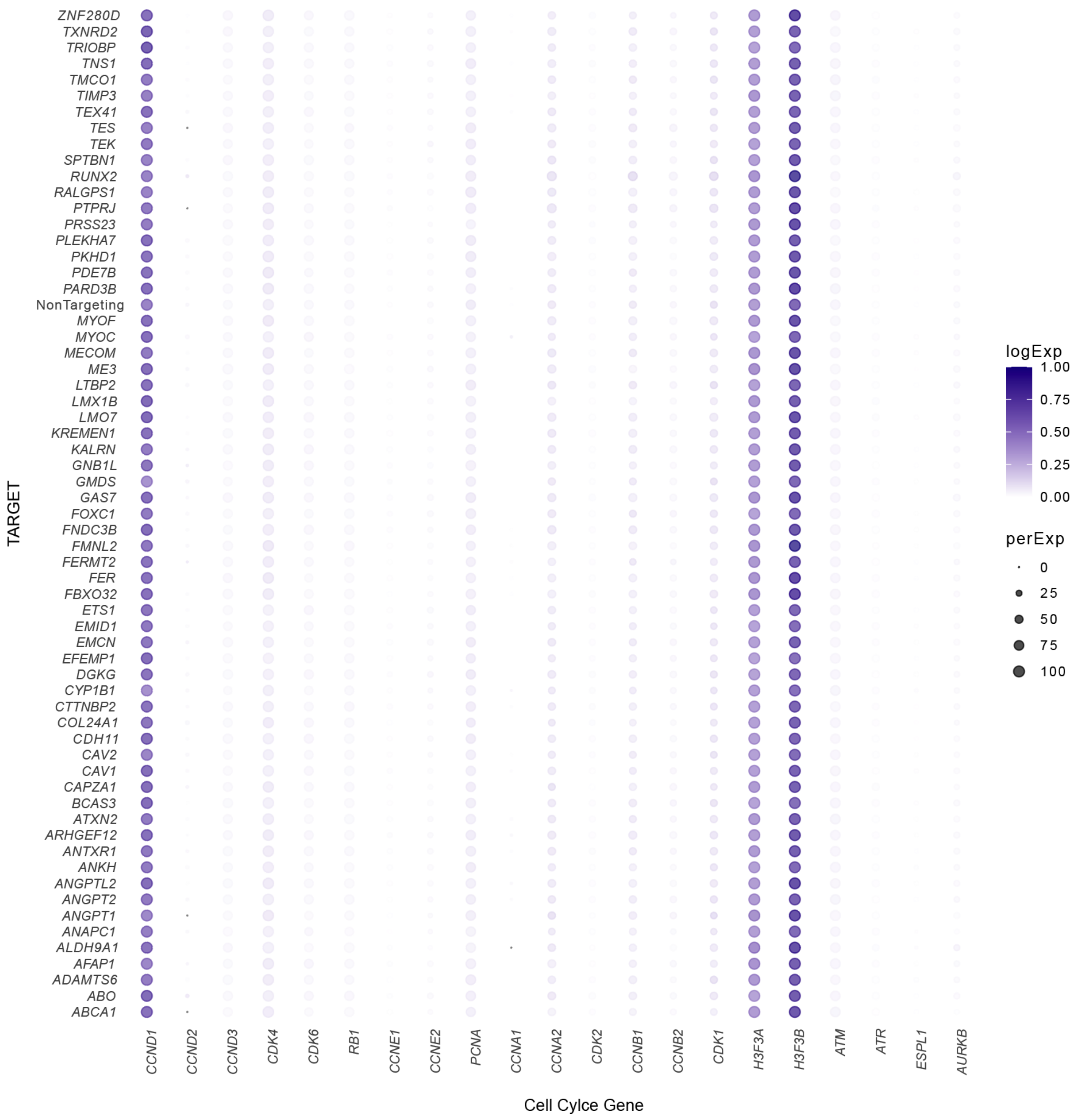

Supplementary Figure 6:
Overlap in differentially expressed genes for each individual gRNA and combined gene-based gRNA analysis across all cell types. Each Venn diagram depicts the number of statistically significant differentially expressed genes, with the component with the highest number in each set highlighted in red. The gRNA or combined gRNA analysis (as depicted by the target gene name) are underlined when the target gene expression levels was significantly reduced (P<0.05) compared to the non-targeting control cells.

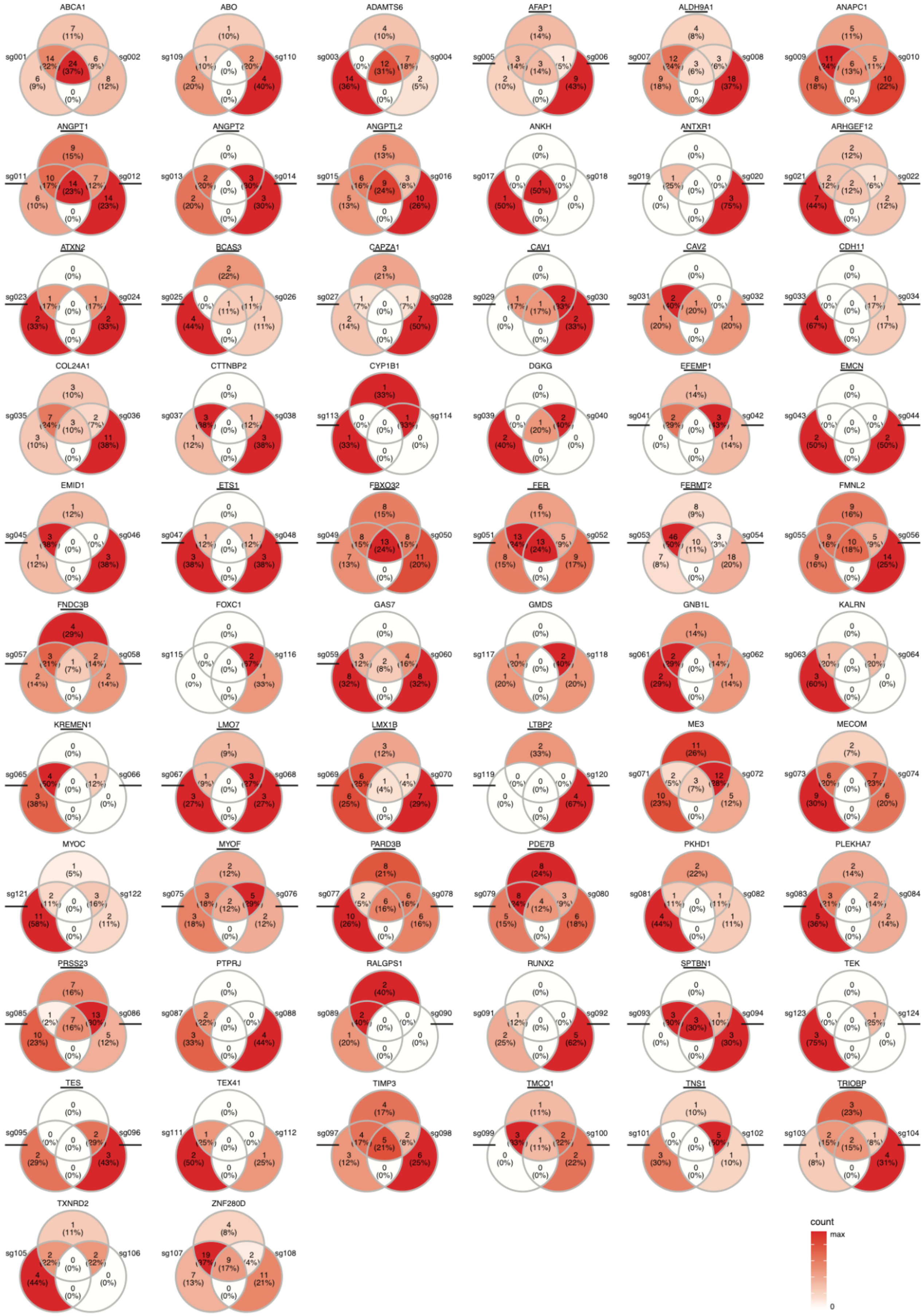
ABCA1 sg001 sg002
ABO sg109 sg110
ADAMTS6 sg003 sg004
AFAP1 sg005 sg006
ALDH9A1 sg007 sg008
ANAPC1 sg009 sg010
ANGPT1 sg011 sg012
ANGPT2 sg013 sg014
ANGPTL2 sg015 sg016
ANKH sg017 sg018
ANTXR1 sg019 sg020
ARHGEF12 sg021 sg022
ATXN2 sg023 sg024
BCAS3 sg025 sg026
CAPZA1 sg027 sg028
CAV1 sg029 sg030
CAV2 sg031 sg032
CDH11 sg033 sg034
COL24A1 sg035 sg036
CTTNBP2 sg037 sg038
CYP1B1 sg113 sg114
DGKG sg039 sg040
EFEMP1 sg041 sg042
EMCN sg043 sg044
EMID1 sg045 sg046
ETS1 sg047 sg048
FBXO32 sg049 sg050
FER sg051 sg052
FERMT2 sg053 sg054
FMNL2 sg055 sg056
FNDC3B sg057 sg058
FOXC1 sg115 sg116
GAS7 sg059 sg060
GMDS sg117 sg118
GNB1L sg061 sg062
KALRN sg063 sg064
KREMEN1 sg065 sg066
LMO7 sg067 sg068
LMX1B sg069 sg070
LTBP2 sg119 sg120
ME3 sg071 sg072
MECOM sg073 sg074
MYOC sg121 sg122
MYOF sg075 sg076
PARD3B sg077 sg078
PDE7B sg079 sg080
PKHD1 sg081 sg082
PLEKHA7 sg083 sg084
PRSS23 sg085 sg086
PTPRJ sg087 sg088
RALGPS1 sg089 sg090
RUNX2 sg091 sg092
SPTBN1 sg093 sg094
TEK sg123 sg124
TES sg095 sg096
TEX41 sg111 sg112
TIMP3 sg097 sg098
TMCO1 sg099 sg100
TNS1 sg101 sg102
TRIOBP sg103 sg104
TXNRD2 sg105 sg106
ZNF280D sg107 sg108
count
max
0

Supplementary Figure 7:
Cluster tree displaying the hierarchical clustering of each cell line based on the single cell RNAseq expression profiles. Here, the distance between each group within one branch is closer than those located in different branches. The bottom grid shows the genes within the same locus at the chromosome (black), genes associated with congenital or juvenile glaucoma are (blue), or the non-targeting control cells (red). The number of circles in each row represents the number of clusters found with the respective resolution parameter, with the circle's label and area denoting the number of groups in the cluster. The arrow widths and labels denote what proportion of the cells in each cluster came from the clusters at the previous resolution parameter. Related to ***Figure 1C****.*

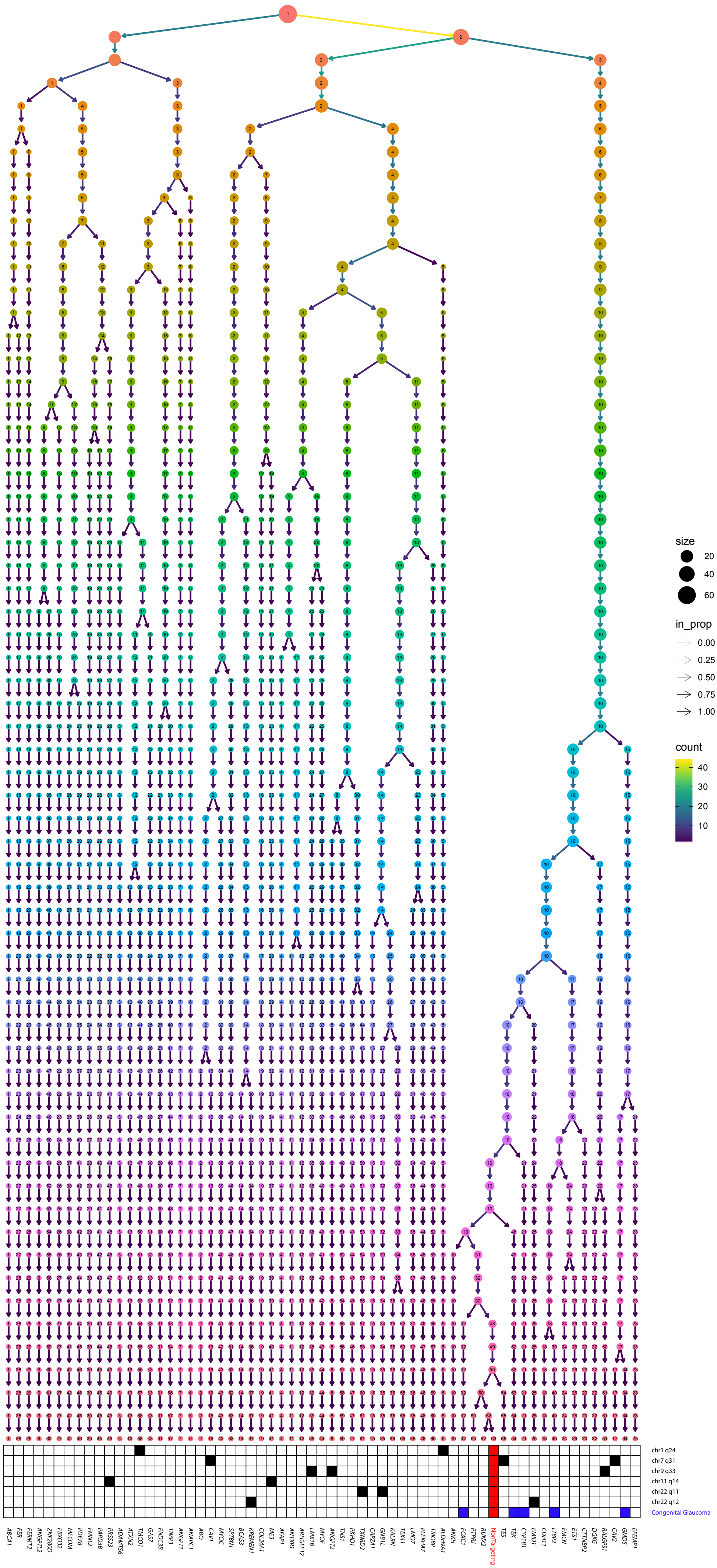
size
20
40
60
in_prop
0.00
0.25
0.50
0.75
1.00
count
40
30
20
10
chr1 q24
chr7 q31
chr9 q33
chr11 q14
chr22 q11
chr22 q12
Congenital Glaucoma
ABCA1
FER
FERMT2
ANGPTL2
ZNF280D
FBXO32
MECOM
PDE7B
FMNL2
PARD3B
PRSS23
ADAMTS6
ATXN2
TMCO1
GAS7
FNDC3B
TIMP3
ANGPT1
ANAPC1
ABO
CAV1
MYOC
SPTBN1
BCAS3
KREMEN1
COL24A1
ME3
AFAP1
ANTXR1
ARHGEF12
LMX1B
MYOF
ANGPT2
TNS1
PKHD1
TXNRD2
CAPZA1
GNB1L
KALRN
TEX41
LMO7
PLEKHA7
TRIOBP
ALDH9A1
ANKH
FOXC1
PTPRJ
RUNX2
NonTargeting
TES
TEK
CYP1B1
EMID1
CDH11
LTBP2
EMCN
ETS1
CTTNBP2
DGKG
RALGPS1
CAV2
GMDS
EFEMP1

Supplementary Figure 8:
Cluster tree displaying the hierarchical clustering of each cell line based on the single cell morphological profiles. Here, the distance between each group within one branch is closer than those located in different branches. The bottom grid shows the genes within the same locus at the chromosome (black), genes associated with congenital or juvenile glaucoma are (blue), or the non-targeting control cells (red). The number of circles in each row represents the number of clusters found with the respective resolution parameter, with the circle's label and area denoting the number of groups in the cluster. The arrow widths and labels denote what proportion of the cells in each cluster came from the clusters at the previous resolution parameter. Related to ***Figure 1D****.*

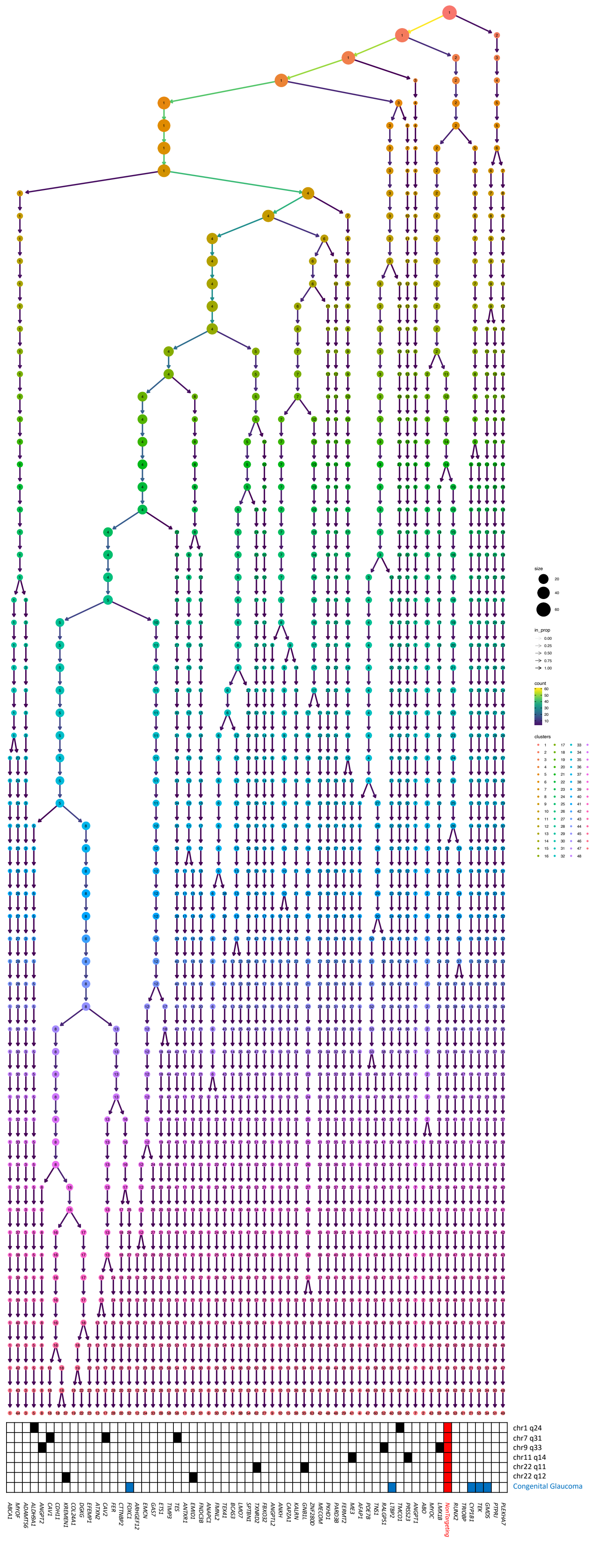
size
20
40
60
in_prop
0.00
0.25
0.50
0.75
1.00
count
60
50
40
30
20
10
clusters
1 2 3 4 5 6 7 8 9 10 11 12 13 14 15 16 17 18 19 20 21 22 23 24 25 26 27 28 29 30 31 32 33 34 35 36 37 38 39 40 41 42 43 44 45 46 47 48 49 50 51 52 53 54 55 56 57 58 59 60 61 62 63
chr1 q24
chr7 q31
chr9 q33
chr11 q14
chr22 q11
chr22 q12
Congenital Glaucoma
ABCA1
MYOF
ADAMTS6
ALDH9A1
ANGPT2
CAV1
CDH11
KREMEN1
COL24A1
DGKG
EFEMP1
ATXN2
CAV2
FER
CTTNBP2
FOXC1
ARHGEF12
EMCN
GAS7
ETS1
TIMP3
TES
ANTXR1
EMID1
FNDC3B
ANAPC1
FMNL2
TEX41
BCAS3
LMO7
SPTBN1
TXNRD2
FBXO32
ANGPTL2
ANKH
CAPZA1
KALRN
GNB1L
ZNF280D
MECOM
PKHD1
PARD3B
FERMT2
ME3
AFAP1
PDE7B
TNS1
RALGPS1
LTBP2
TMCO1
PRSS23
ANGPT1
ABO
MYOC
LMX1B
NonTargeting
RUNX2
TRIOBP
CYP1B1
TEK
GMDS
PTPRJ
PLEKHA7

Supplementary Figure 9:

Scatter plots displaying the SNPs identified in the GWAS conducted by MacGregor et al., 2018 at each multi-gene-loci of interest along with human TMC line sets of transcription start site (TSS) peaks identified by the FANTOM5 project.

<u>chr1:165,514,416-165,914,416:</u>

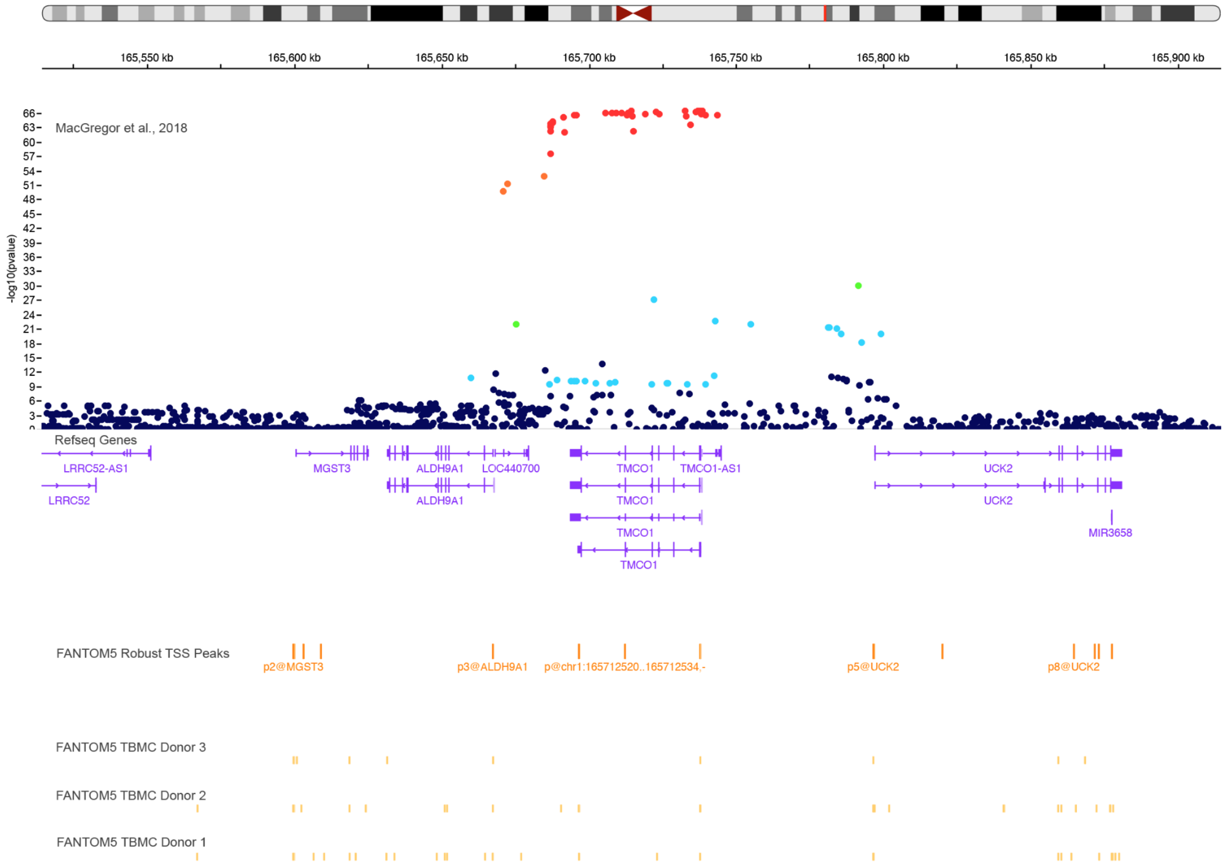

chr7:115,811,771-116,211,771:

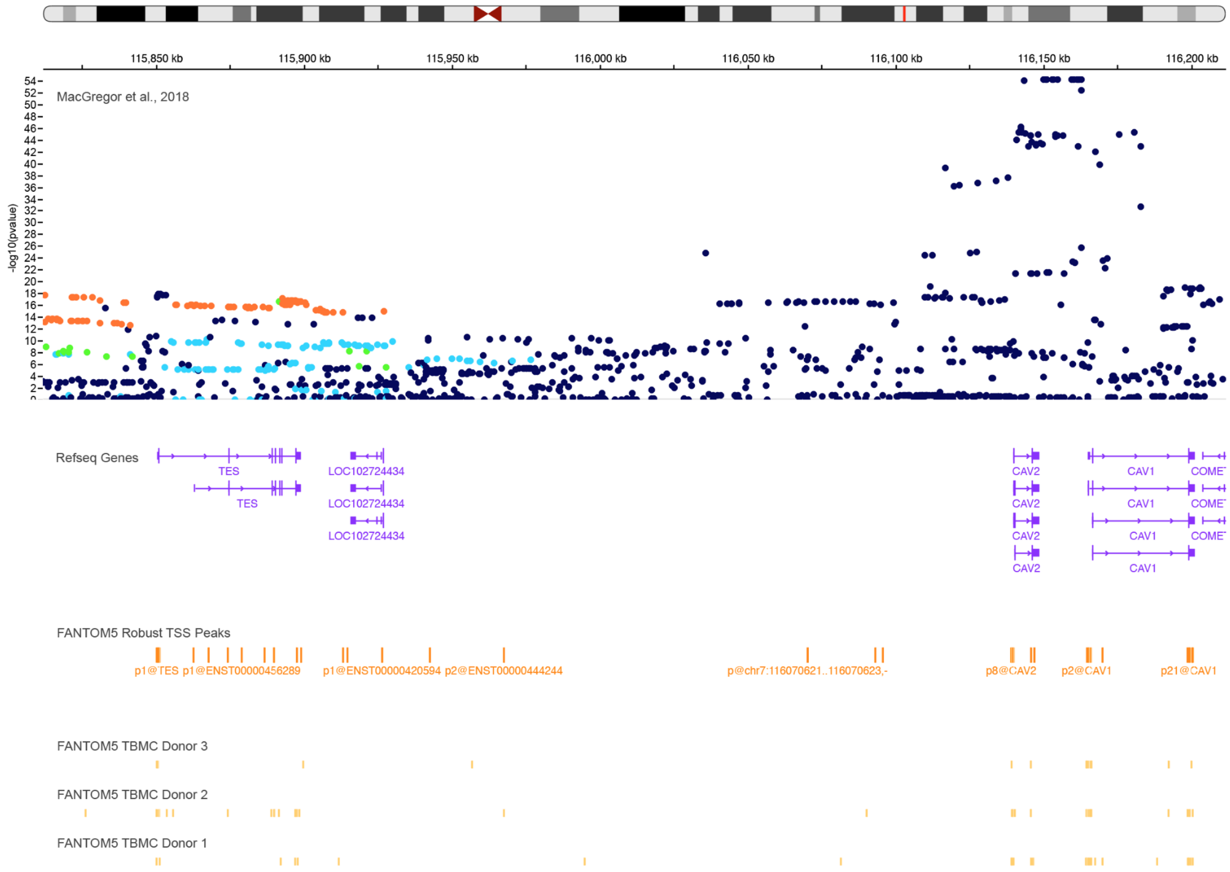

chr9:129,232,361-130,032,363:

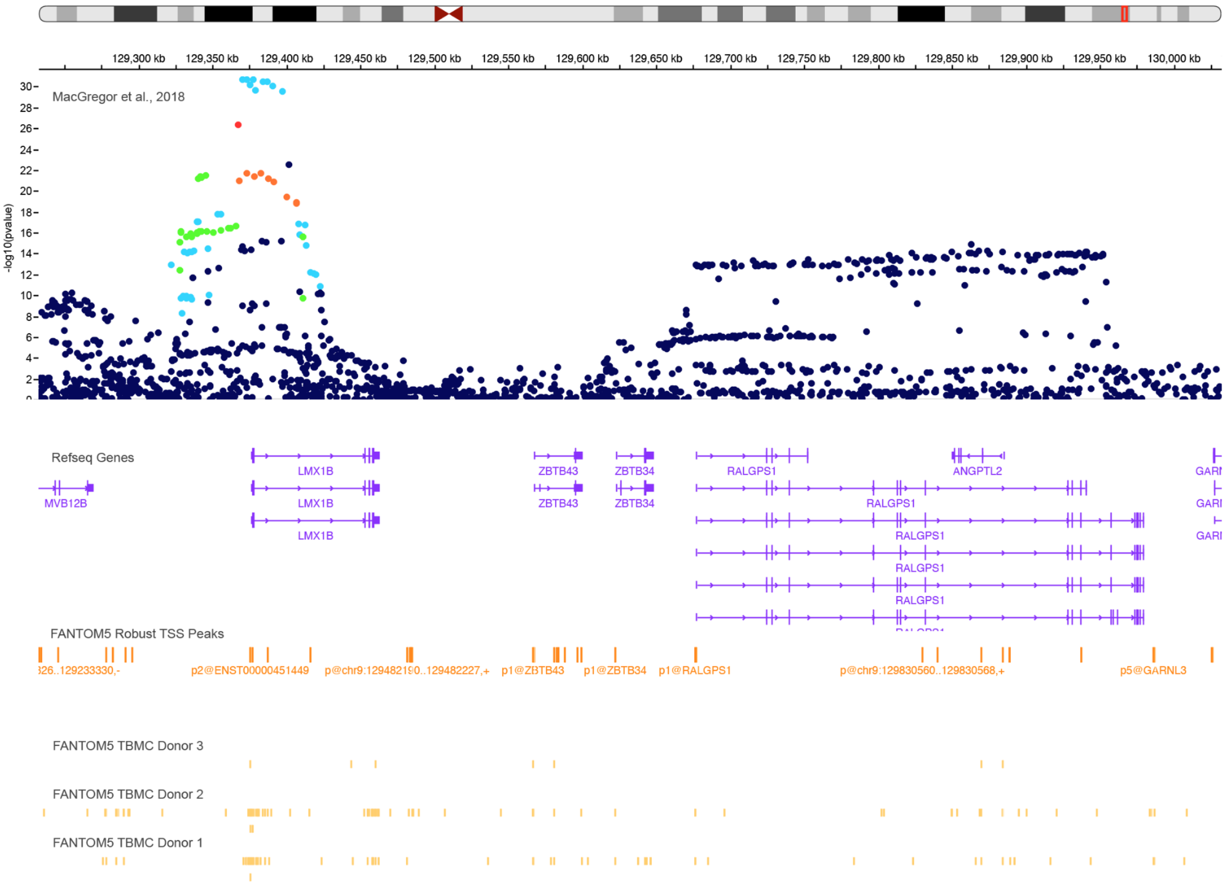

chr11:85,976,231-86,776,232:

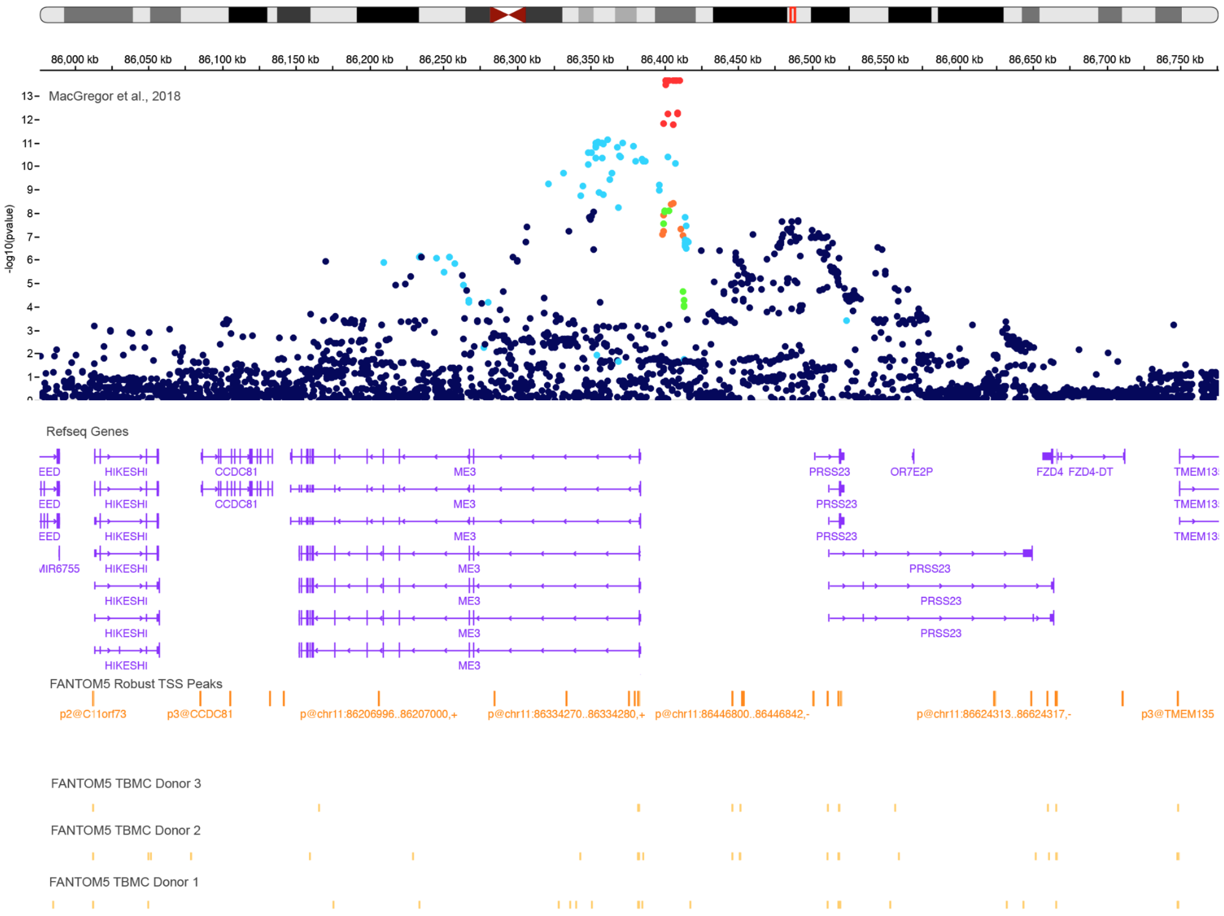

chr22:19,656,962-20,056,962:

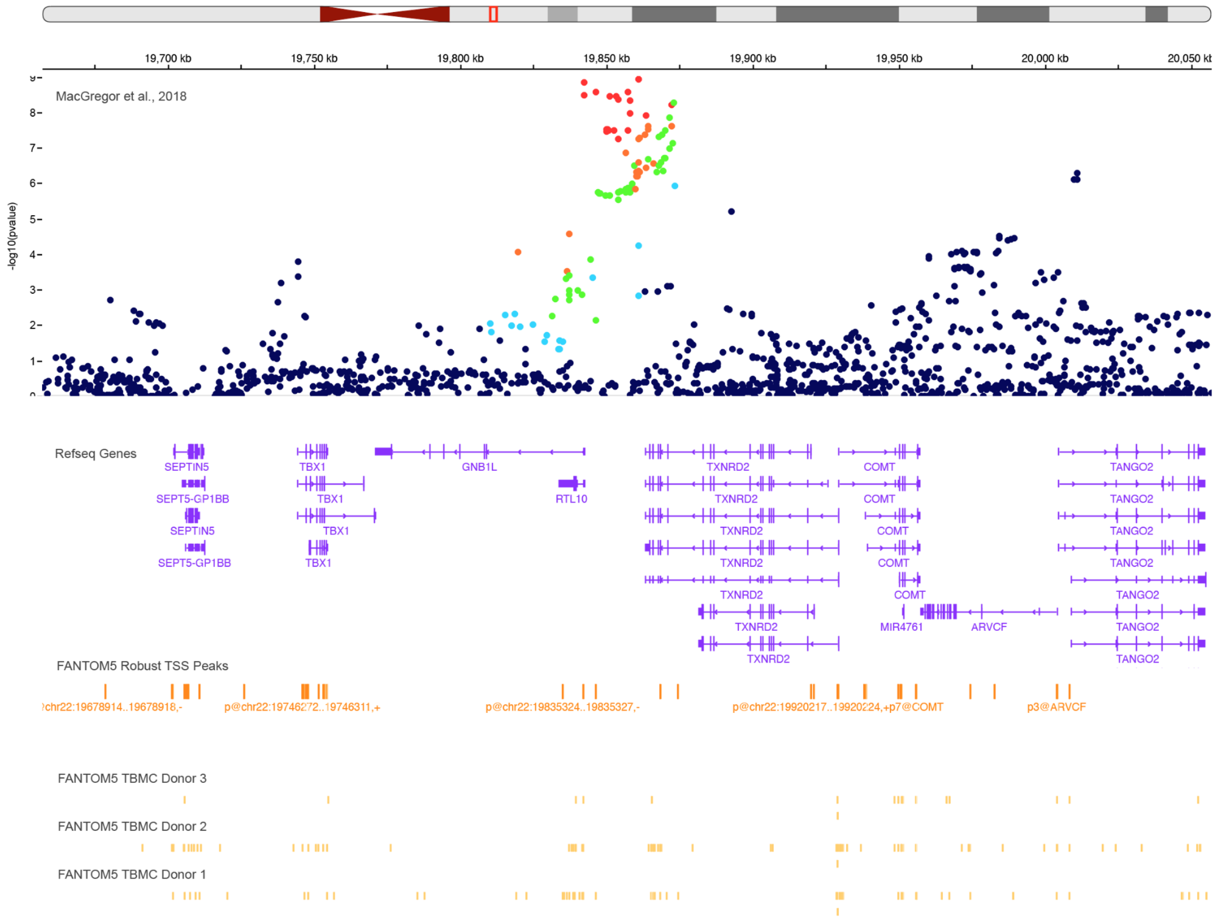

chr22:29,381,091-29,781,091:

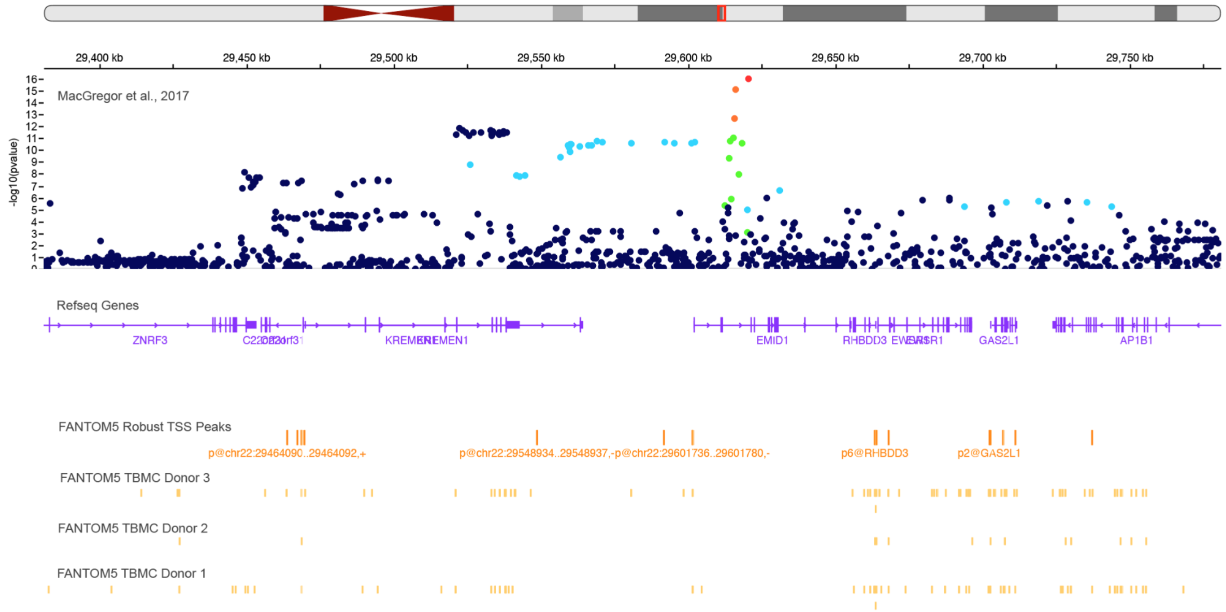

Supplementary Figure 10:

Bulk analysis of differential expressed genes between gene knockout groups compared to non-targeting control groups. Cigar and volcano plots comparing the Log2 expression, and the Log2 fold change versus -log$_{10}$(P value), respectively, for the gene-targeting cells and control non-targeting cells.

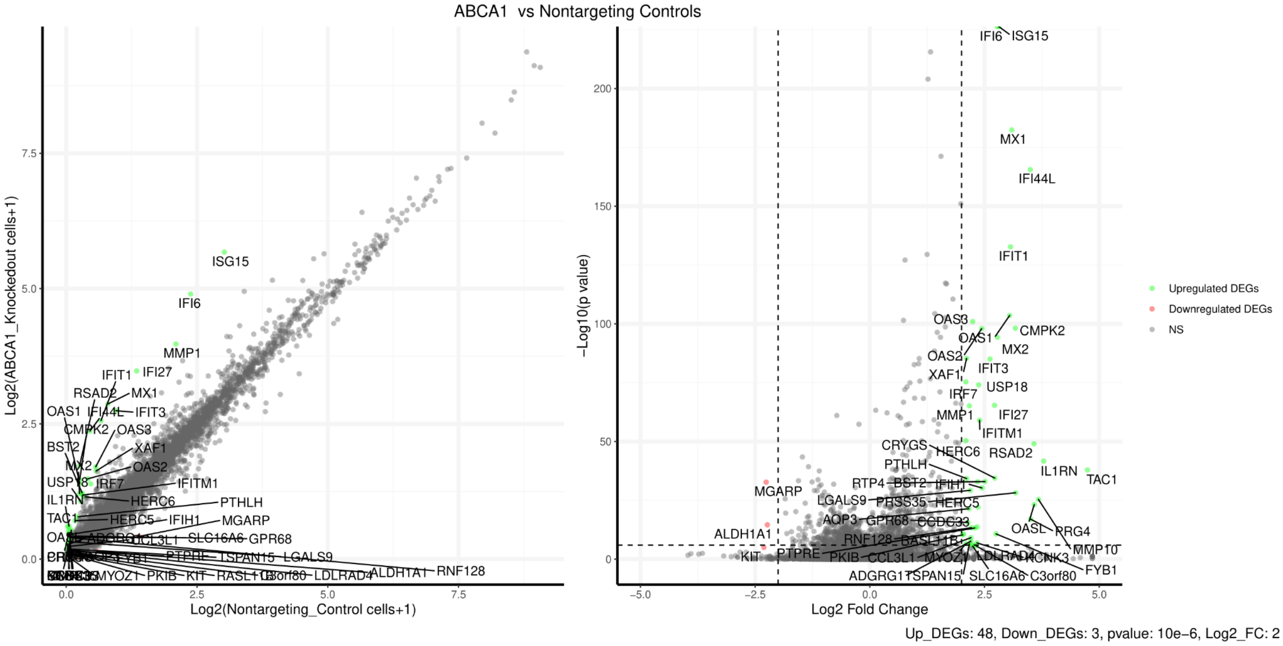

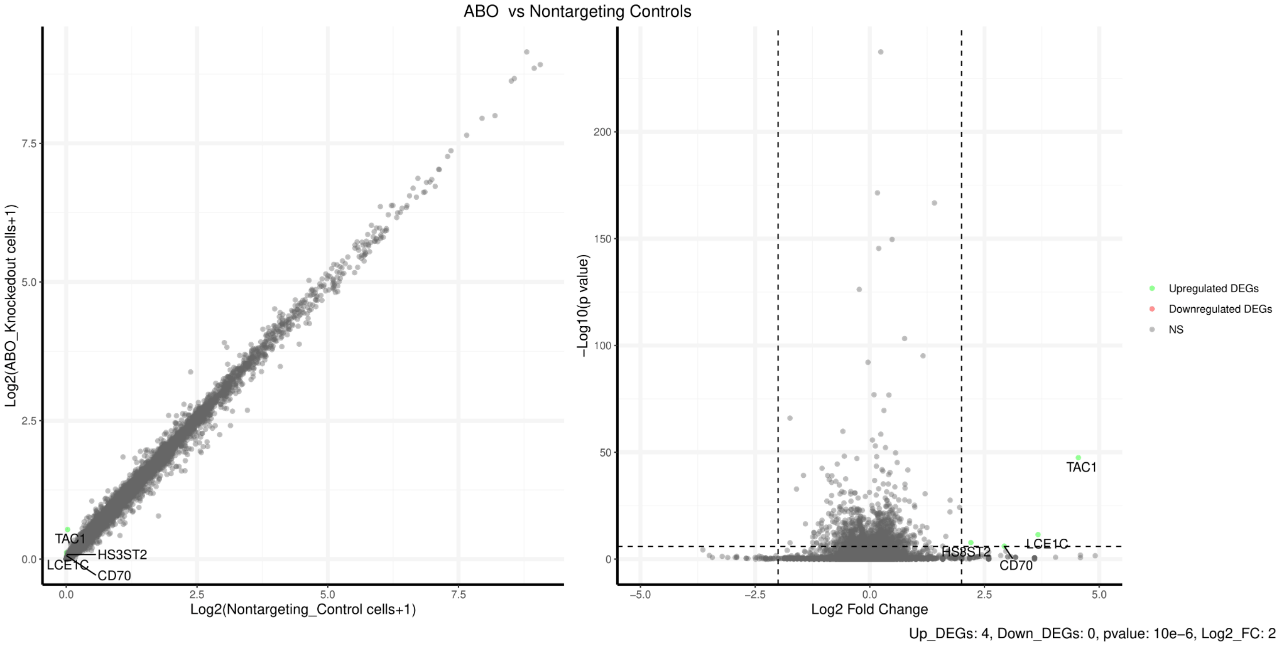

ADAMTS6 vs Nontargeting Controls

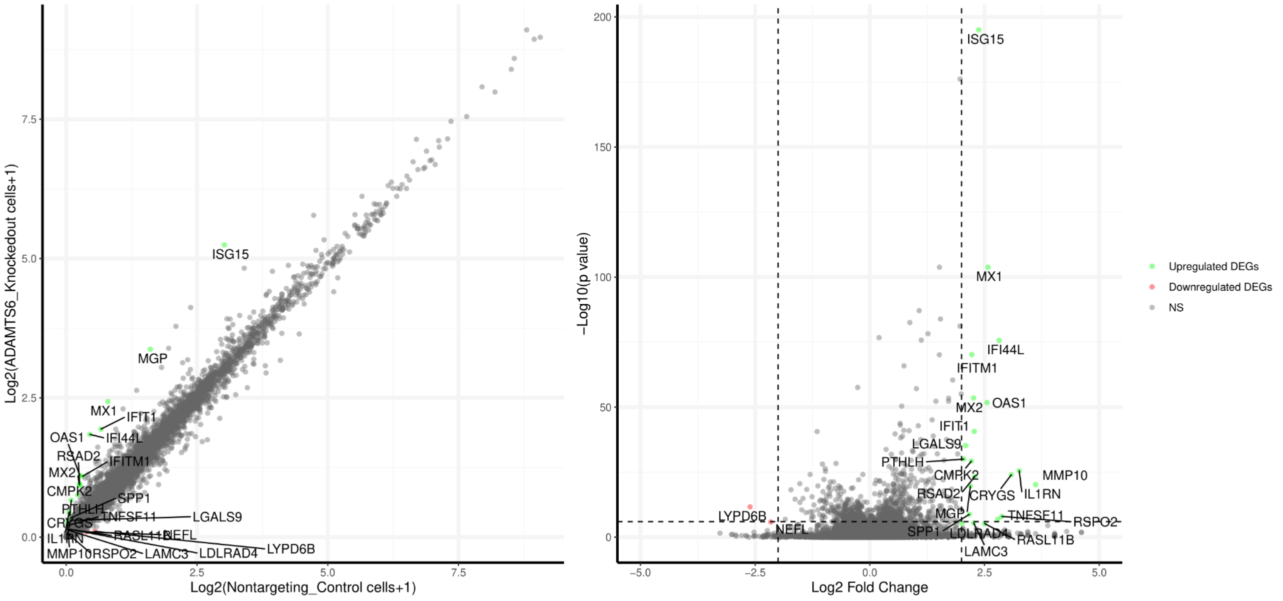

Up_DEGs: 21, Down_DEGs: 2, pvalue: 10e−6, Log2_FC: 2

AFAP1 vs Nontargeting Controls

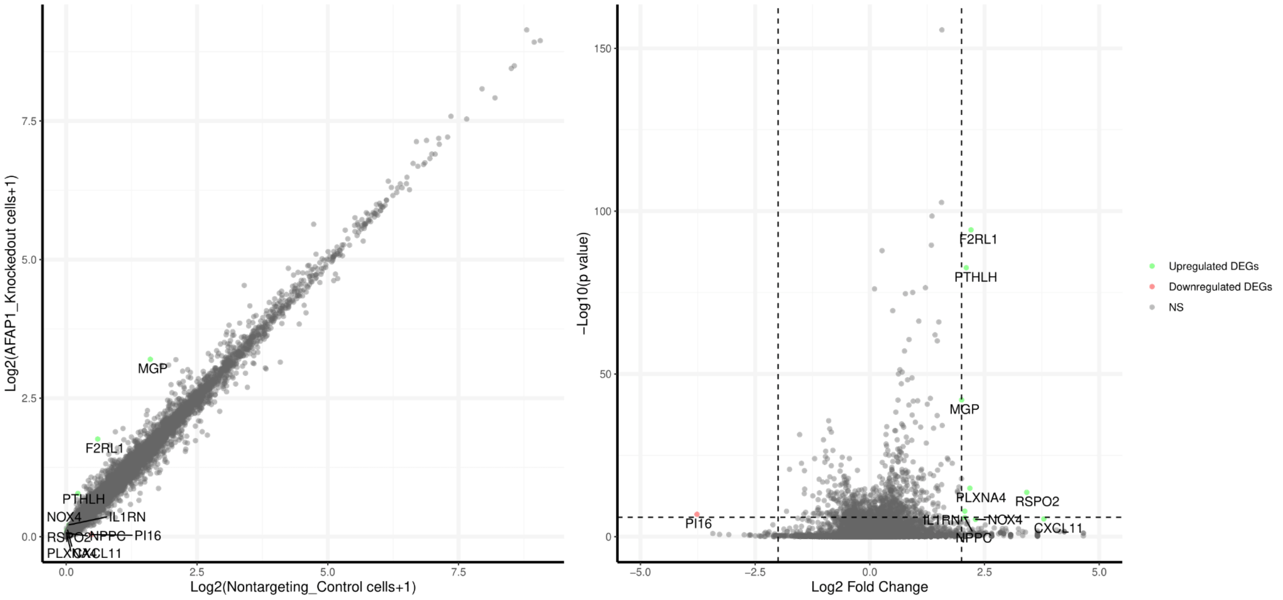

Up_DEGs: 9, Down_DEGs: 1, pvalue: 10e−6, Log2_FC: 2

ALDH9A1 vs Nontargeting Controls

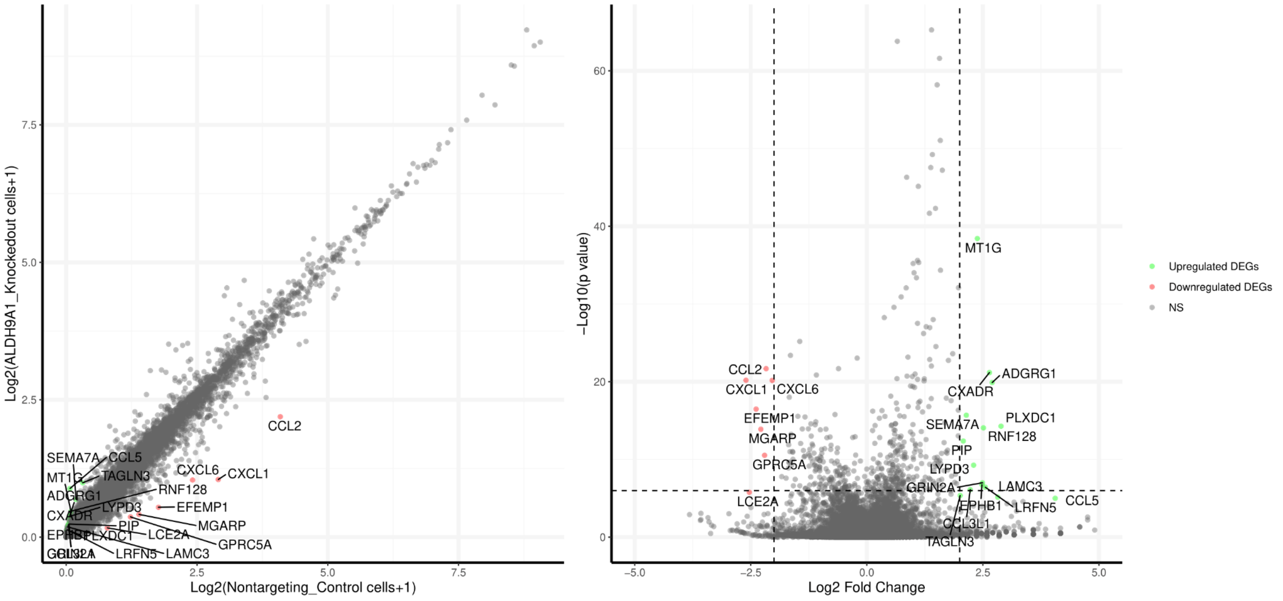

Up_DEGs: 15, Down_DEGs: 7, pvalue: 10e−6, Log2_FC: 2

ANAPC1 vs Nontargeting Controls

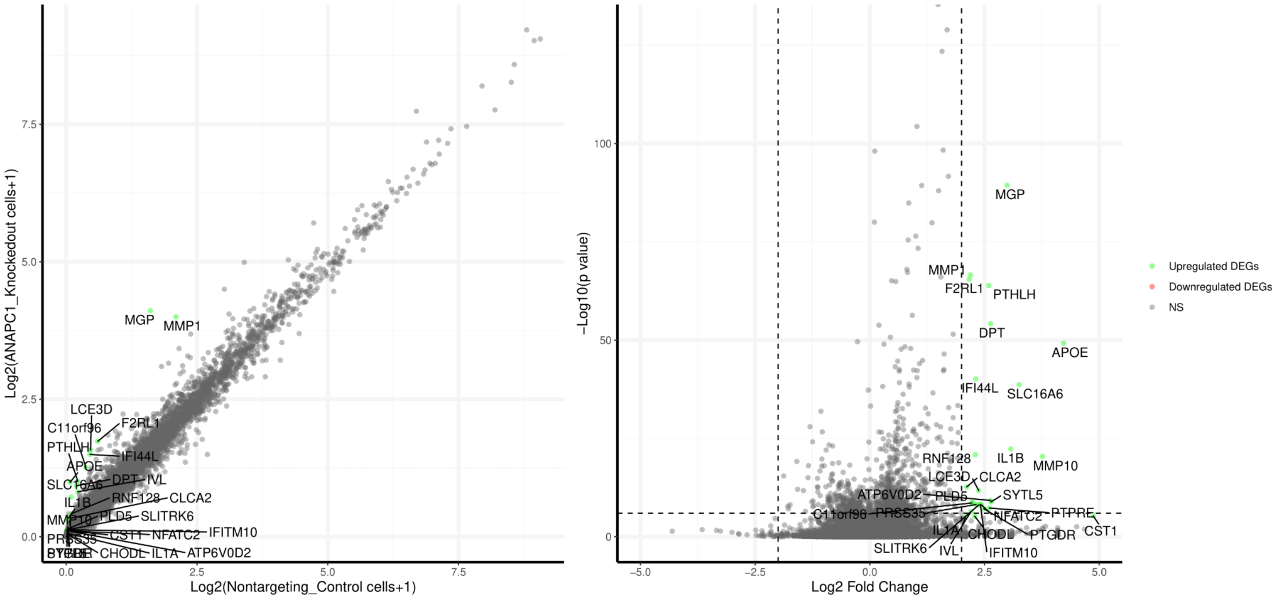

Up_DEGs: 27, Down_DEGs: 0, pvalue: 10e−6, Log2_FC: 2

ANGPT1 vs Nontargeting Controls

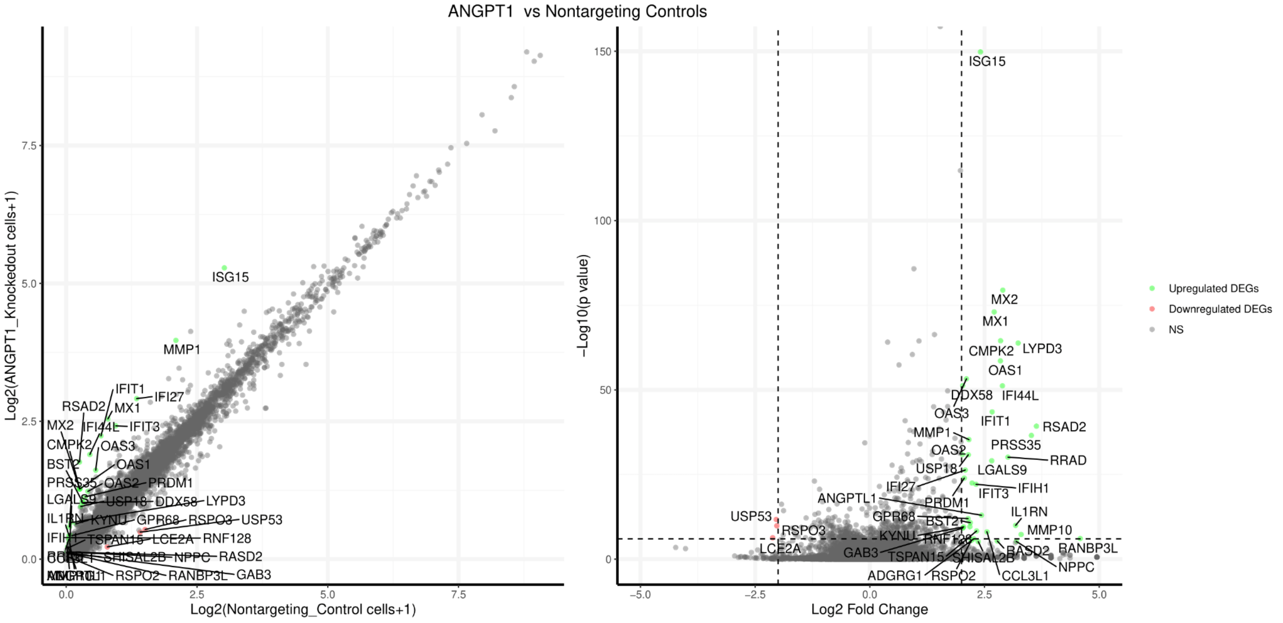

Up_DEGs: 37, Down_DEGs: 3, pvalue: 10e−6, Log2_FC: 2

ANGPT2 vs Nontargeting Controls

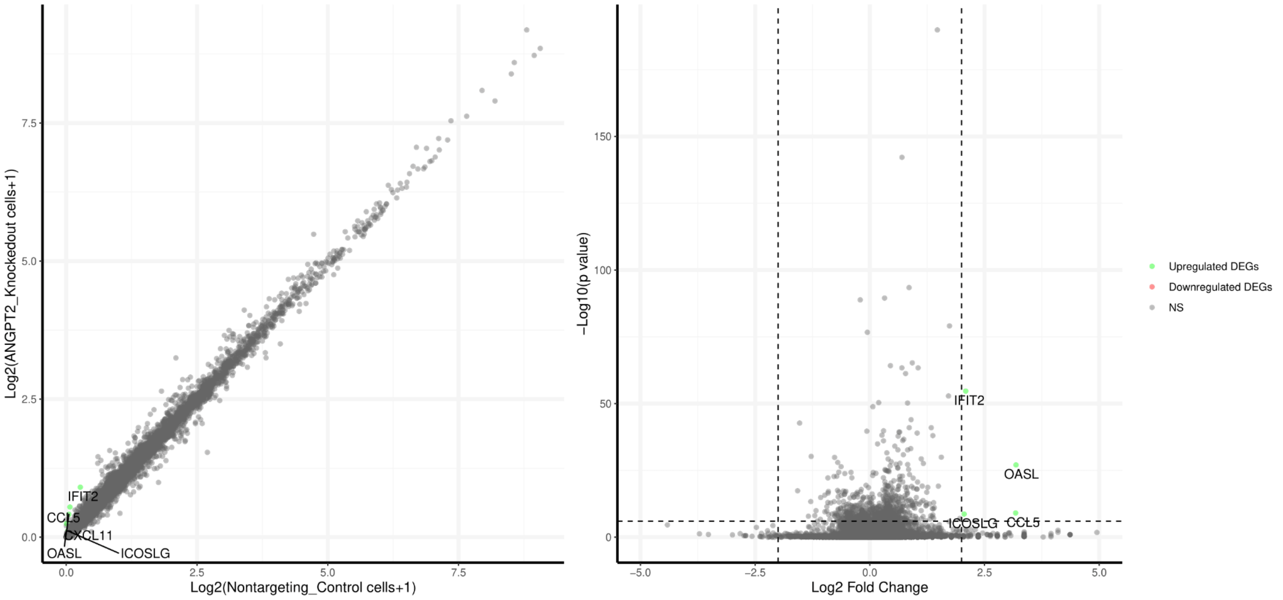

Up_DEGs: 5, Down_DEGs: 0, pvalue: 10e−6, Log2_FC: 2

ANGPTL2 vs Nontargeting Controls

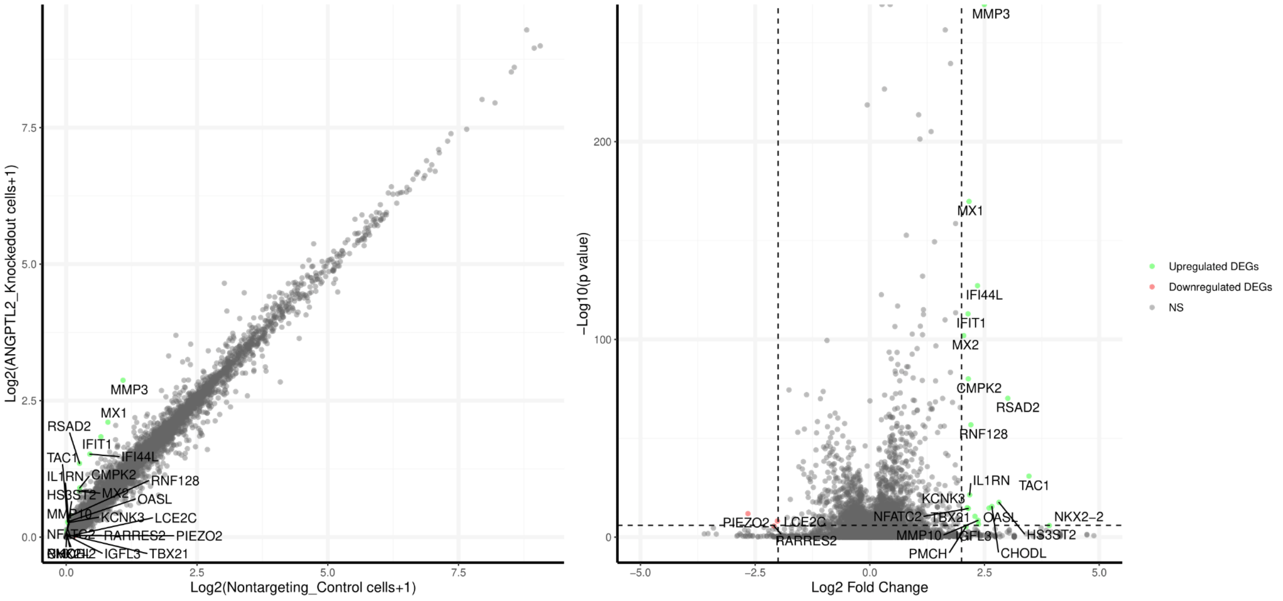

Up_DEGs: 20, Down_DEGs: 3, pvalue: 10e−6, Log2_FC: 2

ANKH vs Nontargeting Controls

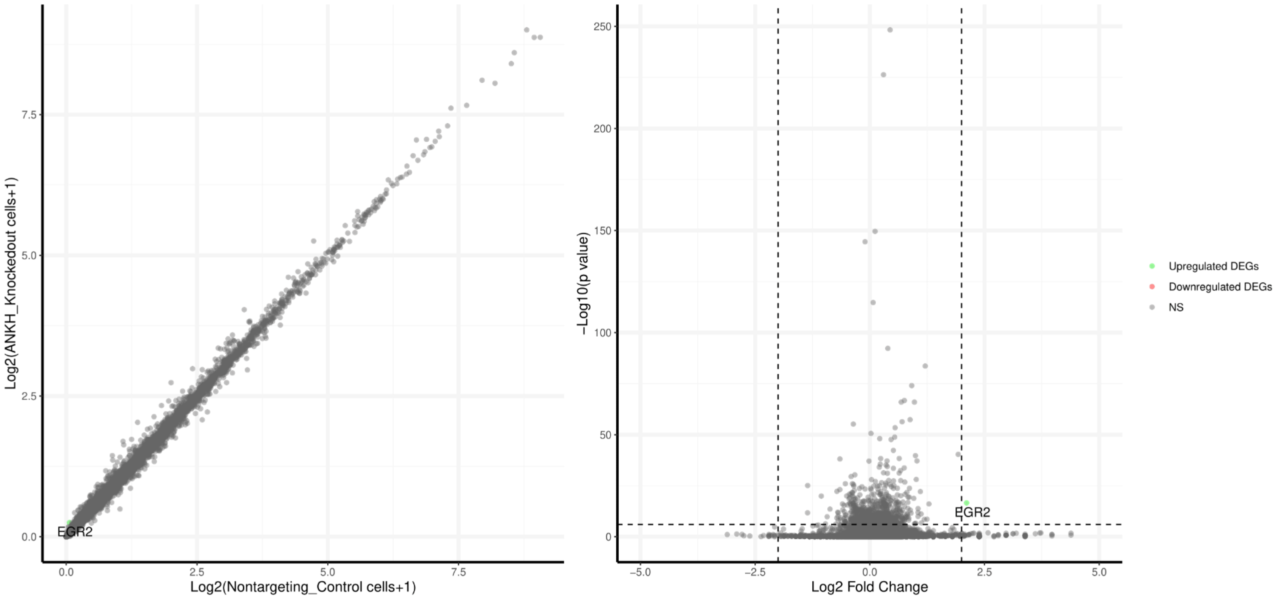

Up_DEGs: 1, Down_DEGs: 0, pvalue: 10e−6, Log2_FC: 2

ANTXR1 vs Nontargeting Controls

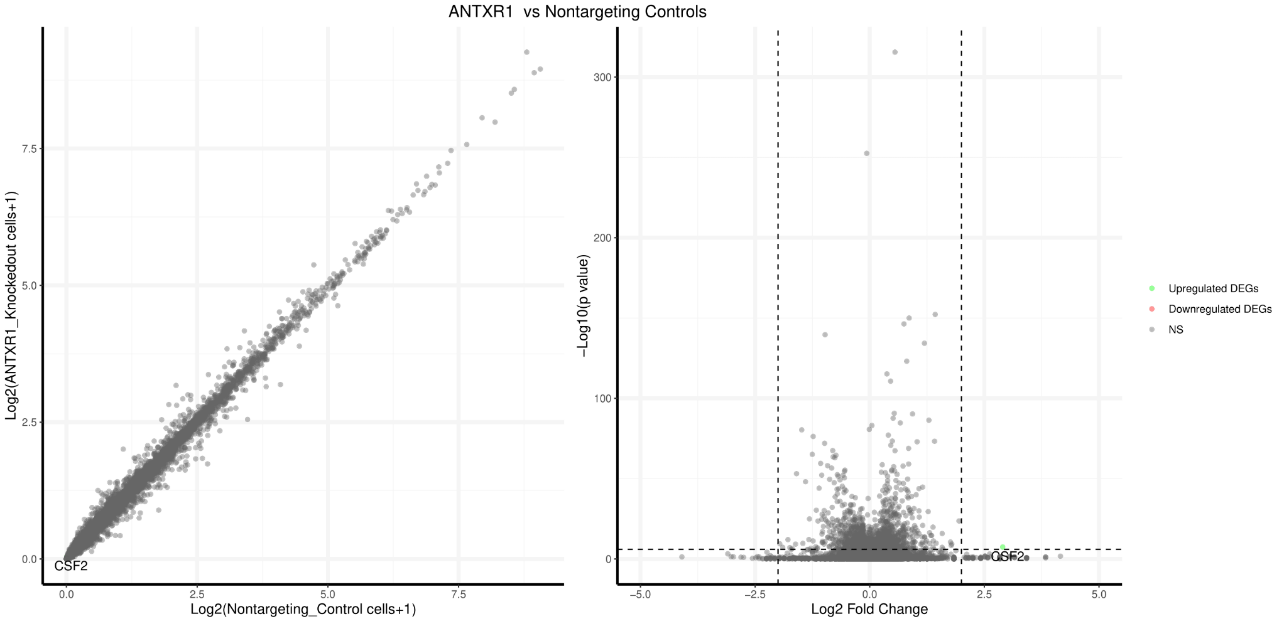

Up_DEGs: 1, Down_DEGs: 0, pvalue: 10e−6, Log2_FC: 2

ARHGEF12 vs Nontargeting Controls

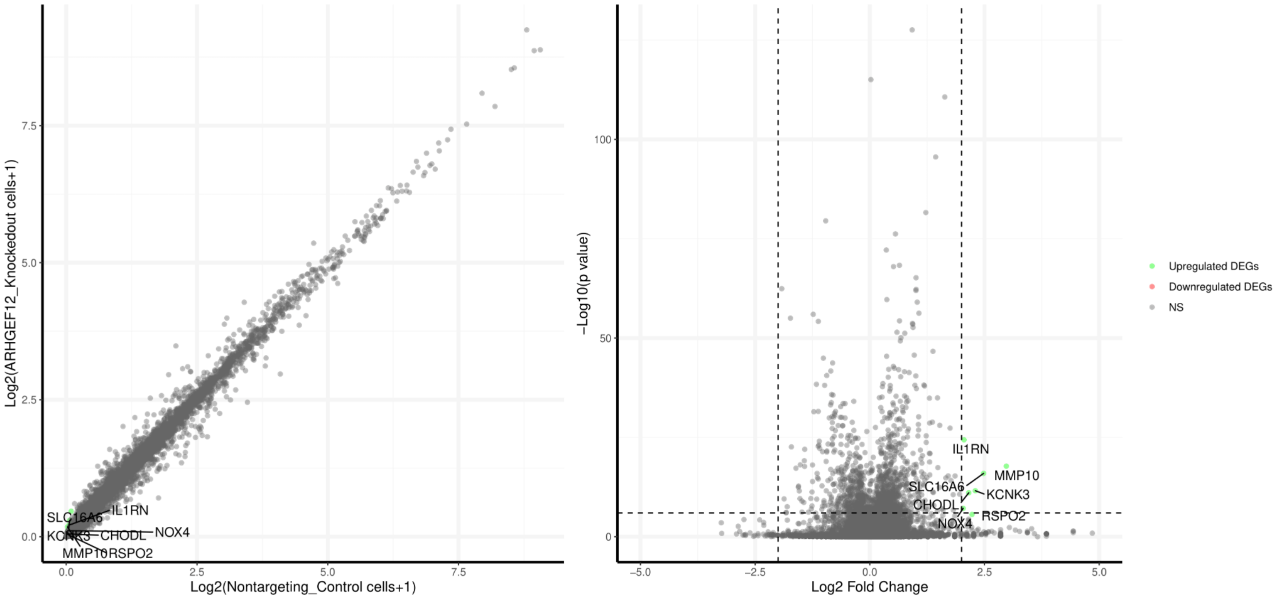

Up_DEGs: 7, Down_DEGs: 0, pvalue: 10e−6, Log2_FC: 2

ATXN2 vs Nontargeting Controls

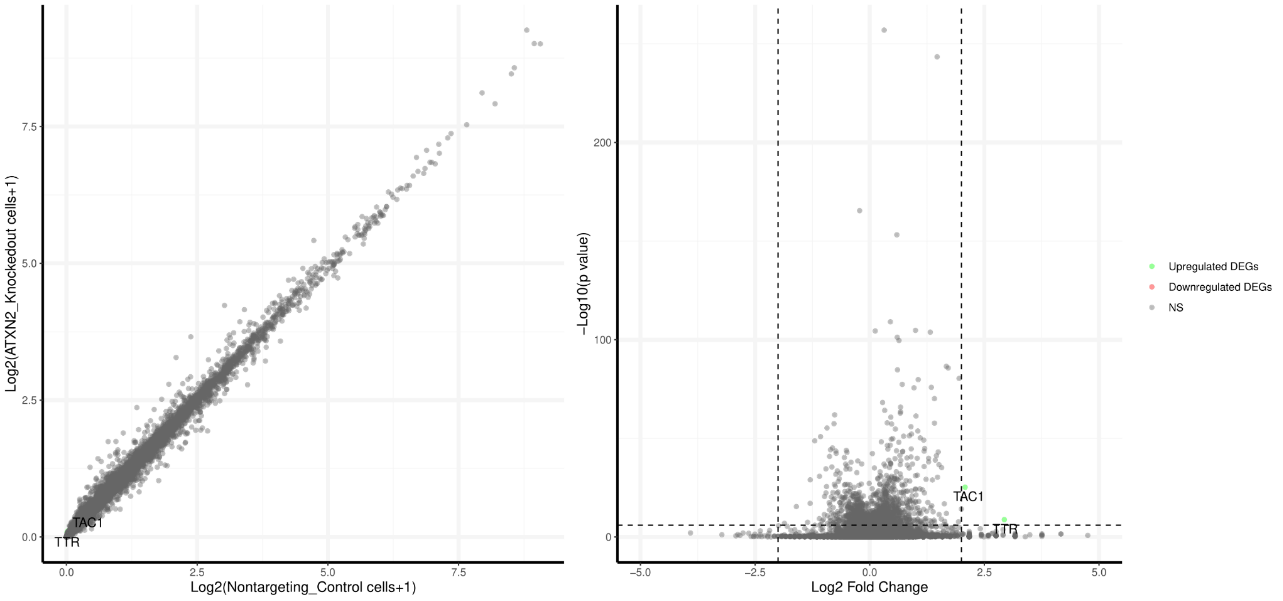

Up_DEGs: 2, Down_DEGs: 0, pvalue: 10e−6, Log2_FC: 2

BCAS3 vs Nontargeting Controls

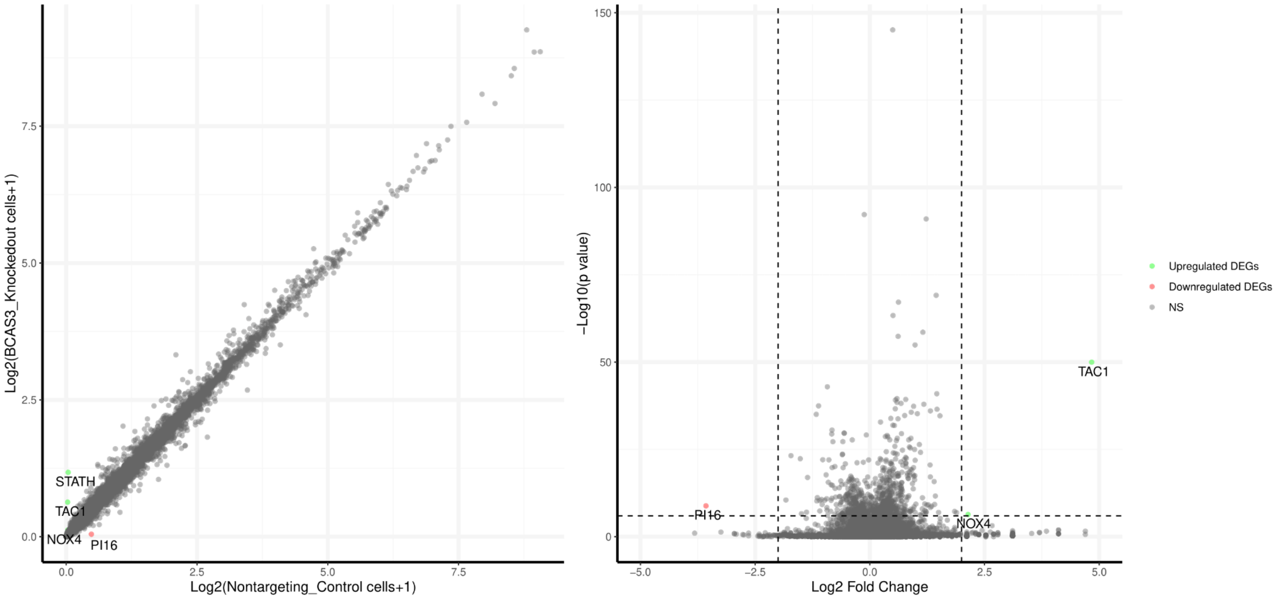

Up_DEGs: 3, Down_DEGs: 1, pvalue: 10e−6, Log2_FC: 2

CAPZA1 vs Nontargeting Controls

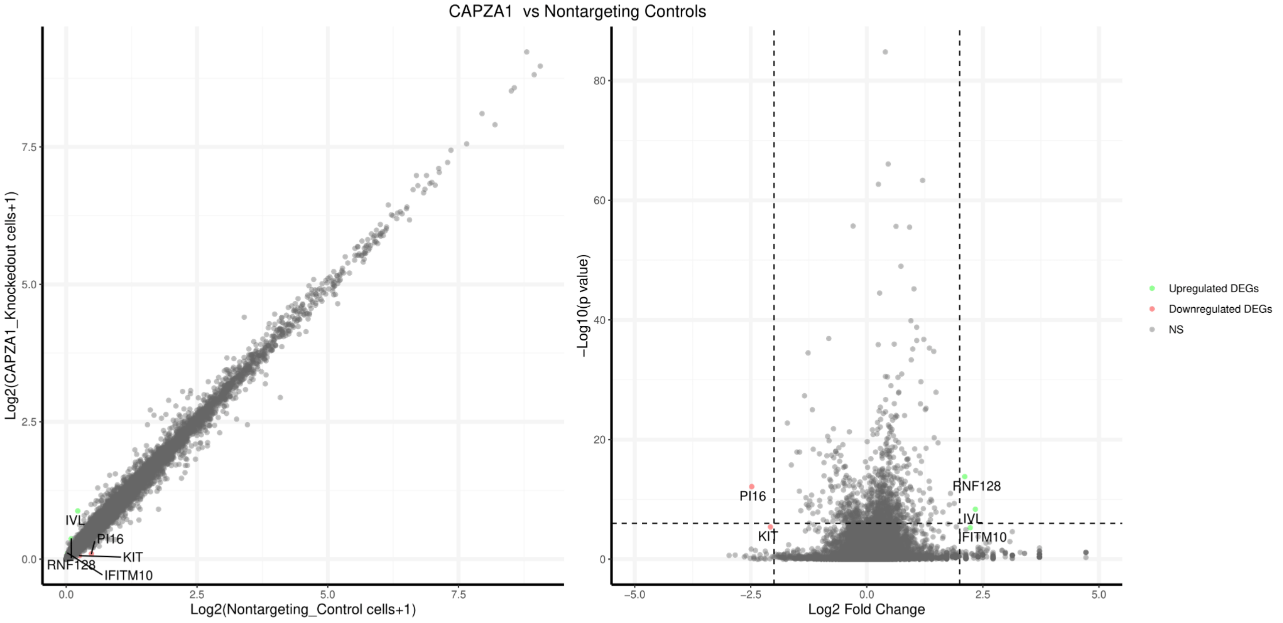

Up_DEGs: 3, Down_DEGs: 2, pvalue: 10e−6, Log2_FC: 2

CAV1 vs Nontargeting Controls

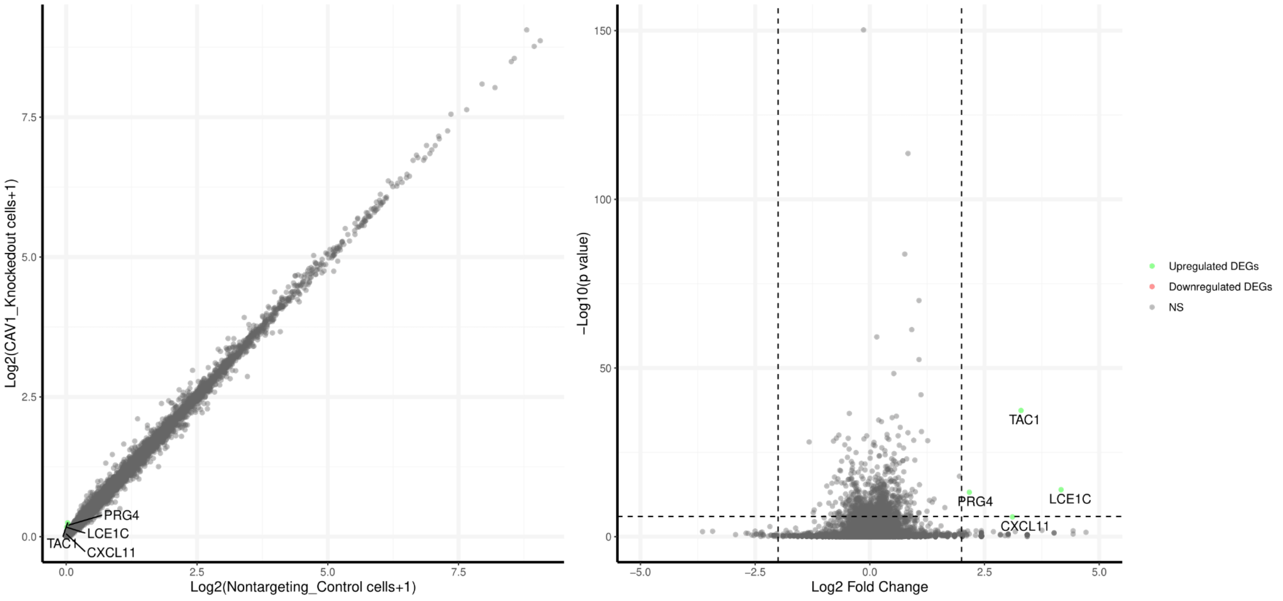

Up_DEGs: 4, Down_DEGs: 0, pvalue: 10e−6, Log2_FC: 2

CAV2 vs Nontargeting Controls

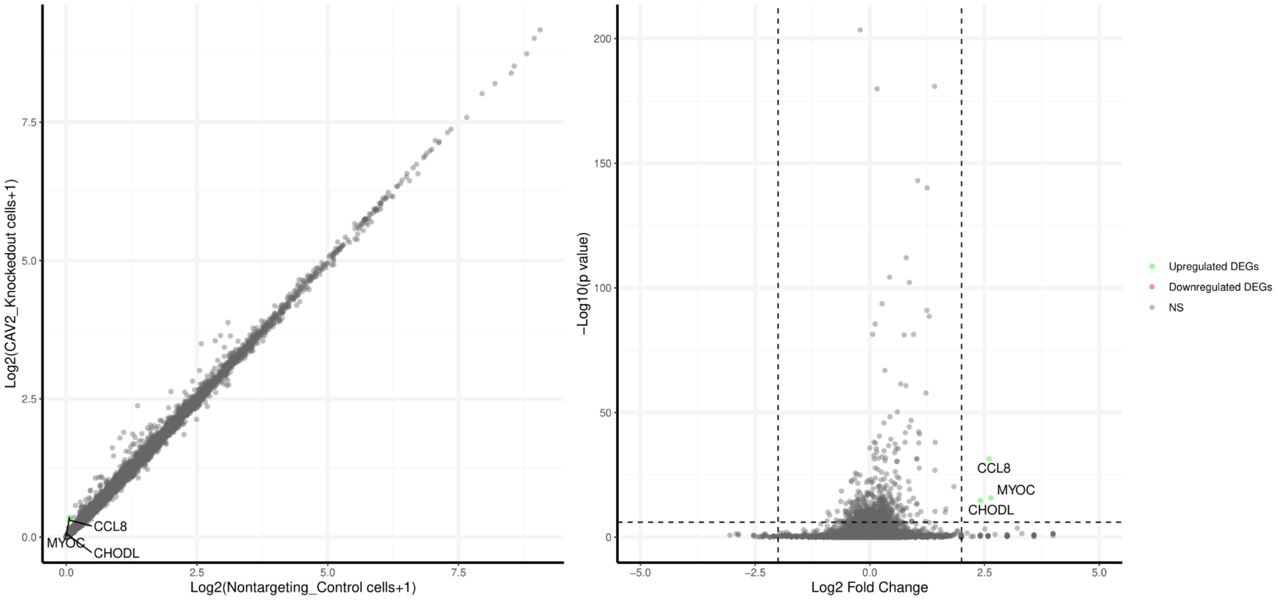

Up_DEGs: 3, Down_DEGs: 0, pvalue: 10e−6, Log2_FC: 2

CDH11 vs Nontargeting Controls

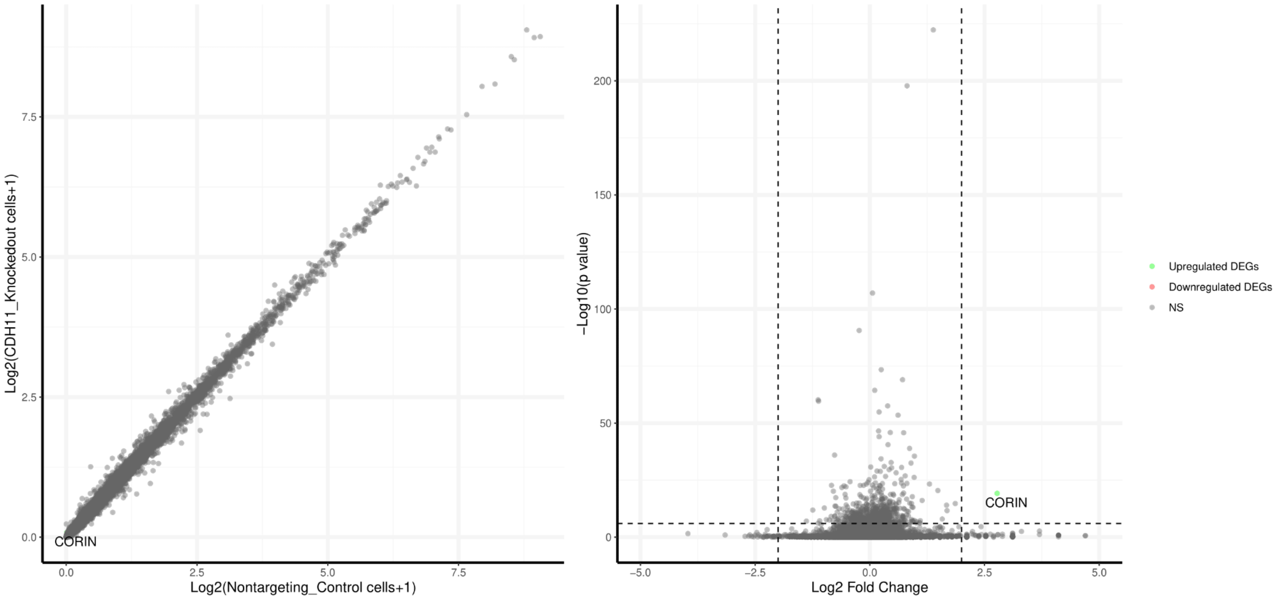

Up_DEGs: 1, Down_DEGs: 0, pvalue: 10e−6, Log2_FC: 2

COL24A1 vs Nontargeting Controls

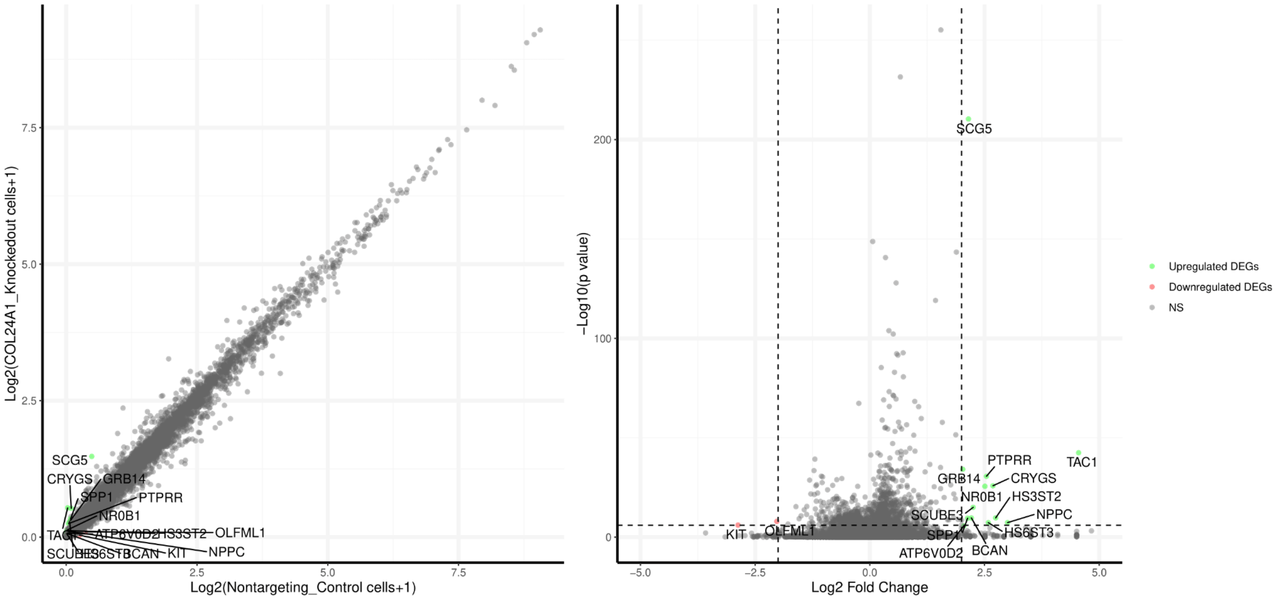

Up_DEGs: 13, Down_DEGs: 2, pvalue: 10e−6, Log2_FC: 2

CTTNBP2 vs Nontargeting Controls

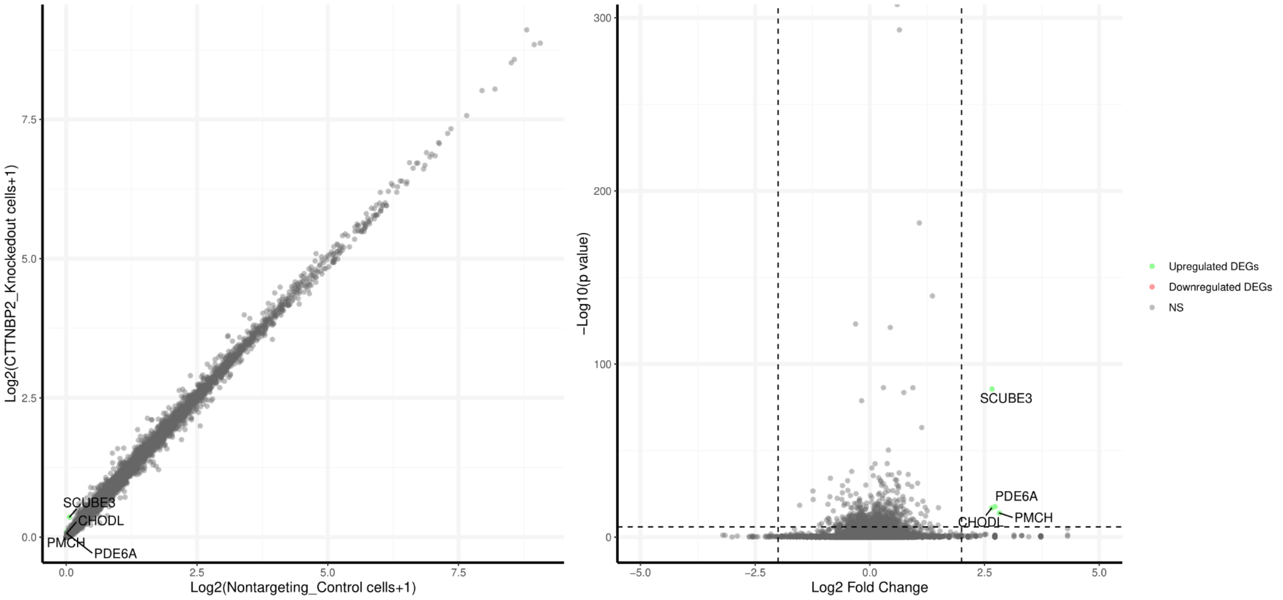

Up_DEGs: 4, Down_DEGs: 0, pvalue: 10e−6, Log2_FC: 2

CYP1B1 vs Nontargeting Controls

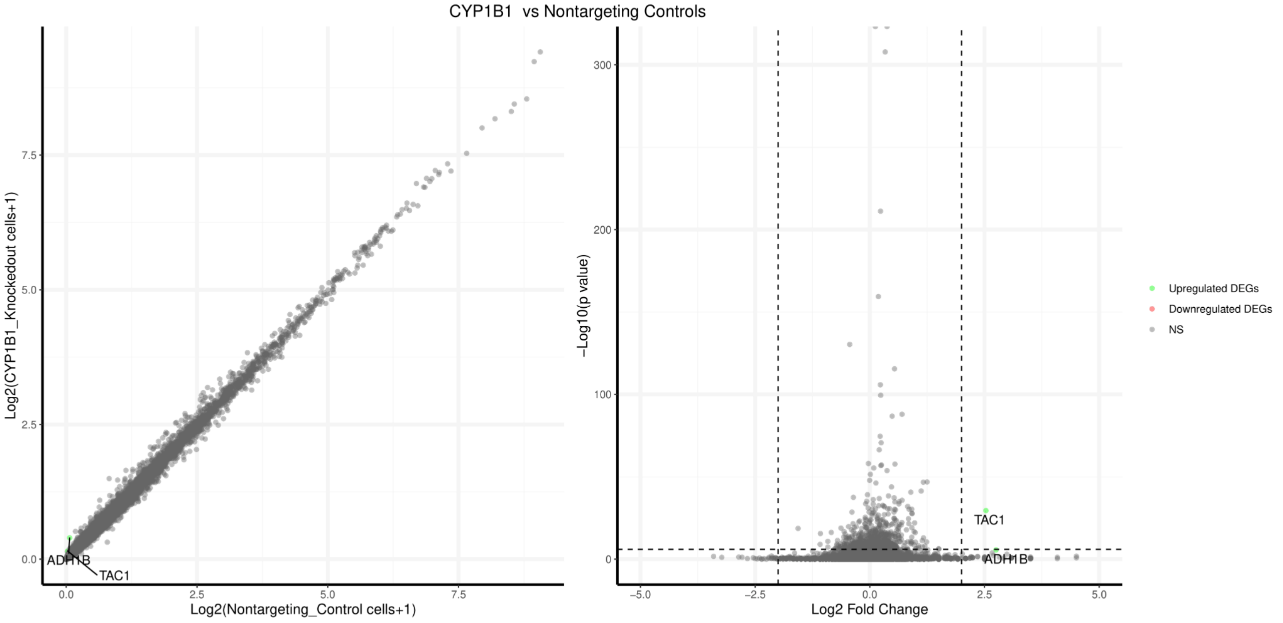

Up_DEGs: 2, Down_DEGs: 0, pvalue: 10e−6, Log2_FC: 2

DGKG vs Nontargeting Controls

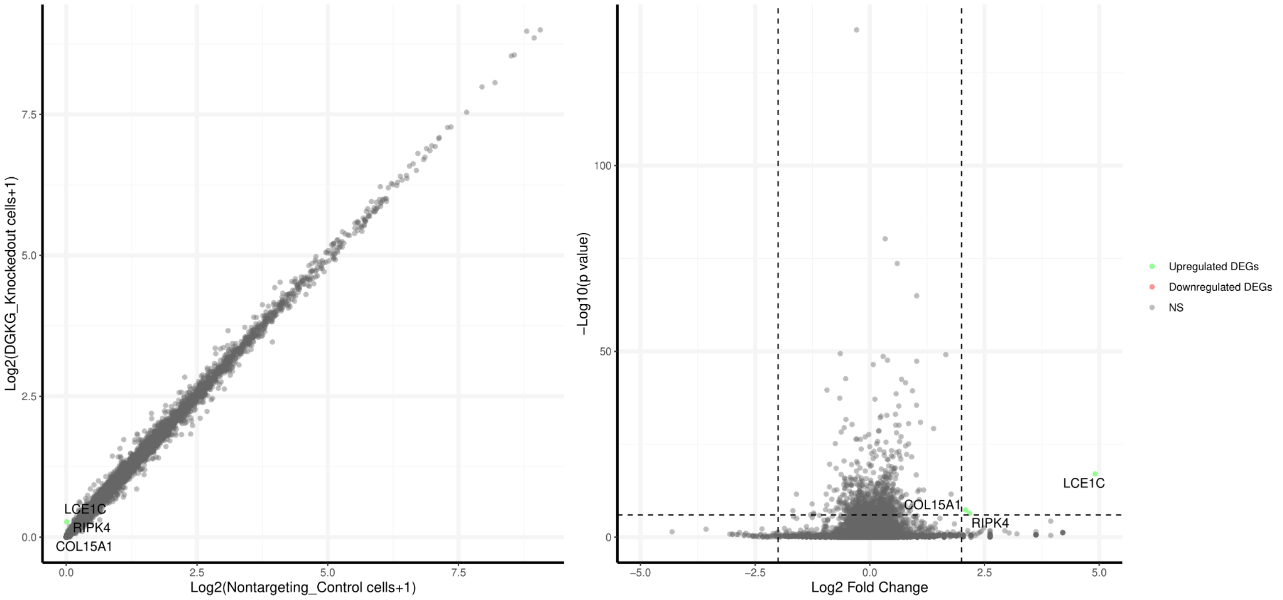

Up_DEGs: 3, Down_DEGs: 0, pvalue: 10e−6, Log2_FC: 2

EFEMP1 vs Nontargeting Controls

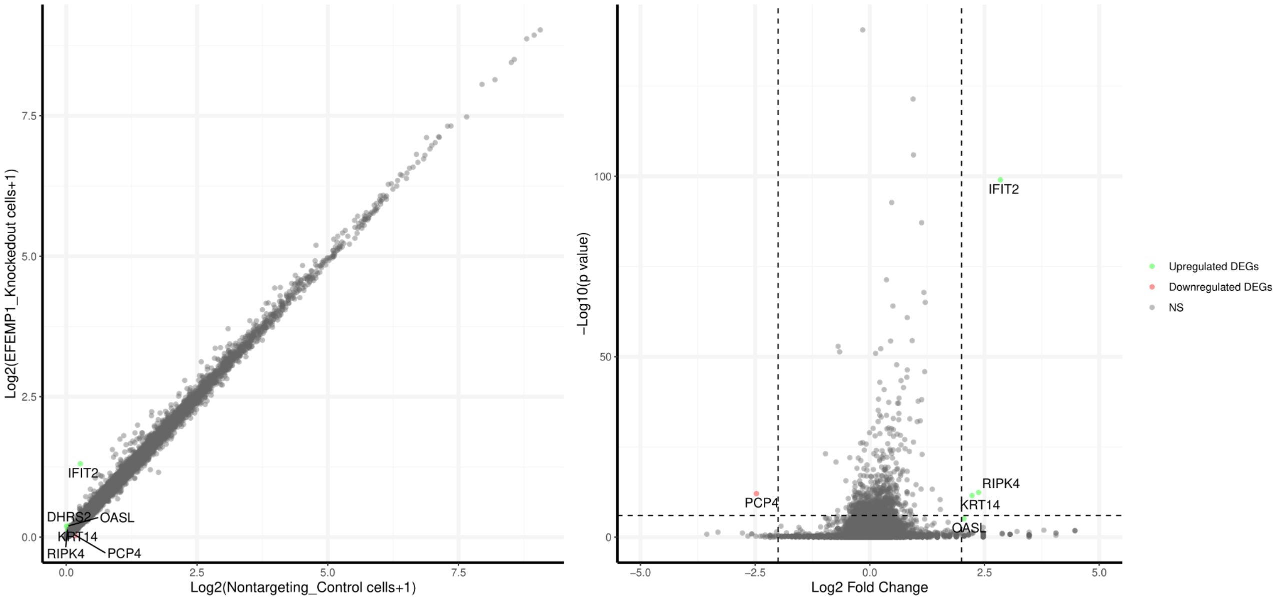

Up_DEGs: 5, Down_DEGs: 1, pvalue: 10e−6, Log2_FC: 2

EMCN vs Nontargeting Controls

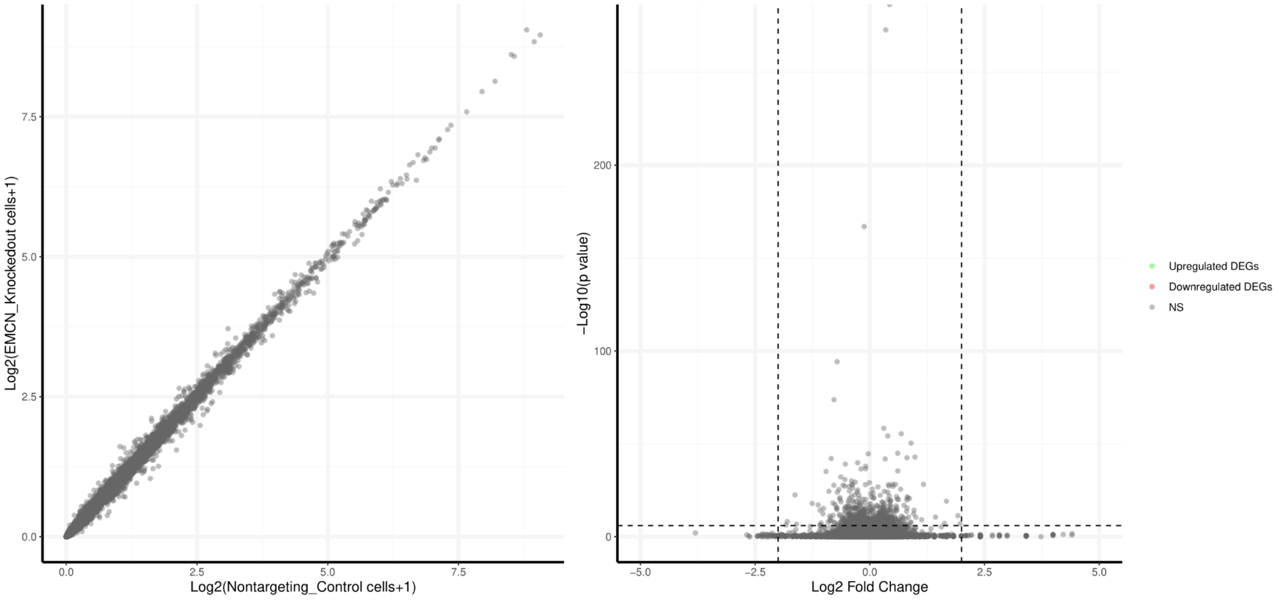

Up_DEGs: 0, Down_DEGs: 0, pvalue: 10e−6, Log2_FC: 2

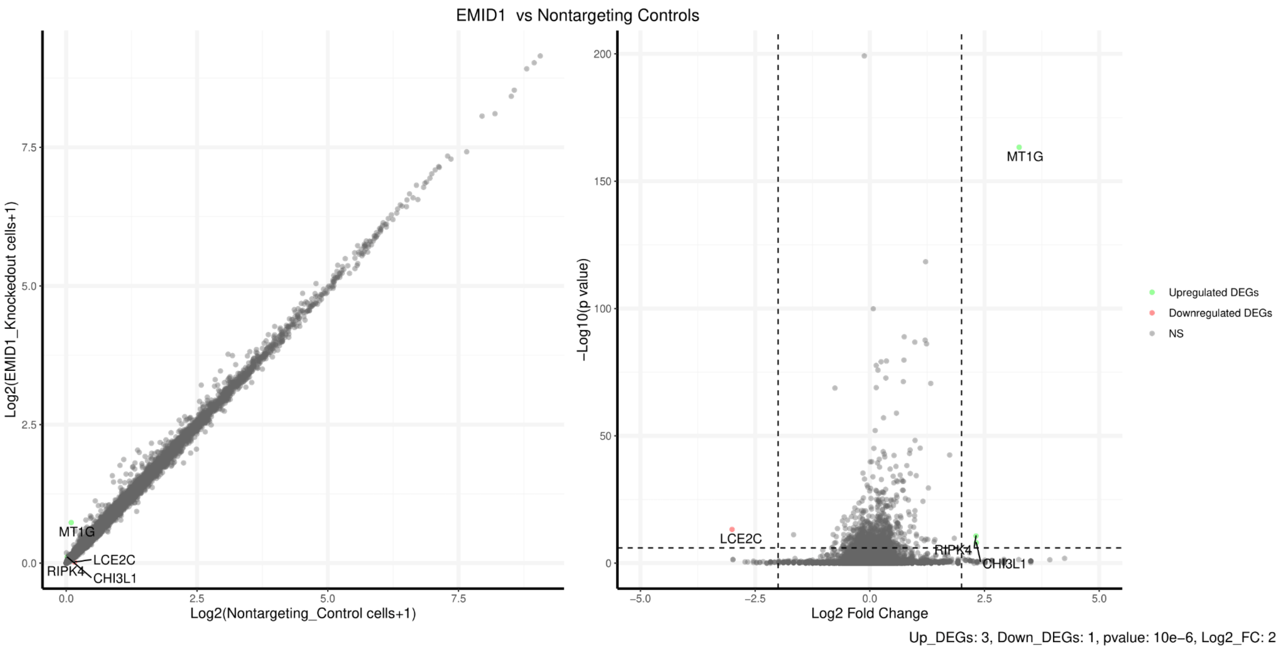
EMID1 vs Nontargeting Controls
Log2(EMID1_Knockedout cells+1)
Log2(Nontargeting_Control cells+1)
MT1G
LCE2C
RIPK4
CHI3L1
−Log10(p value)
Log2 Fold Change
Upregulated DEGs
Downregulated DEGs
NS
Up_DEGs: 3, Down_DEGs: 1, pvalue: 10e−6, Log2_FC: 2

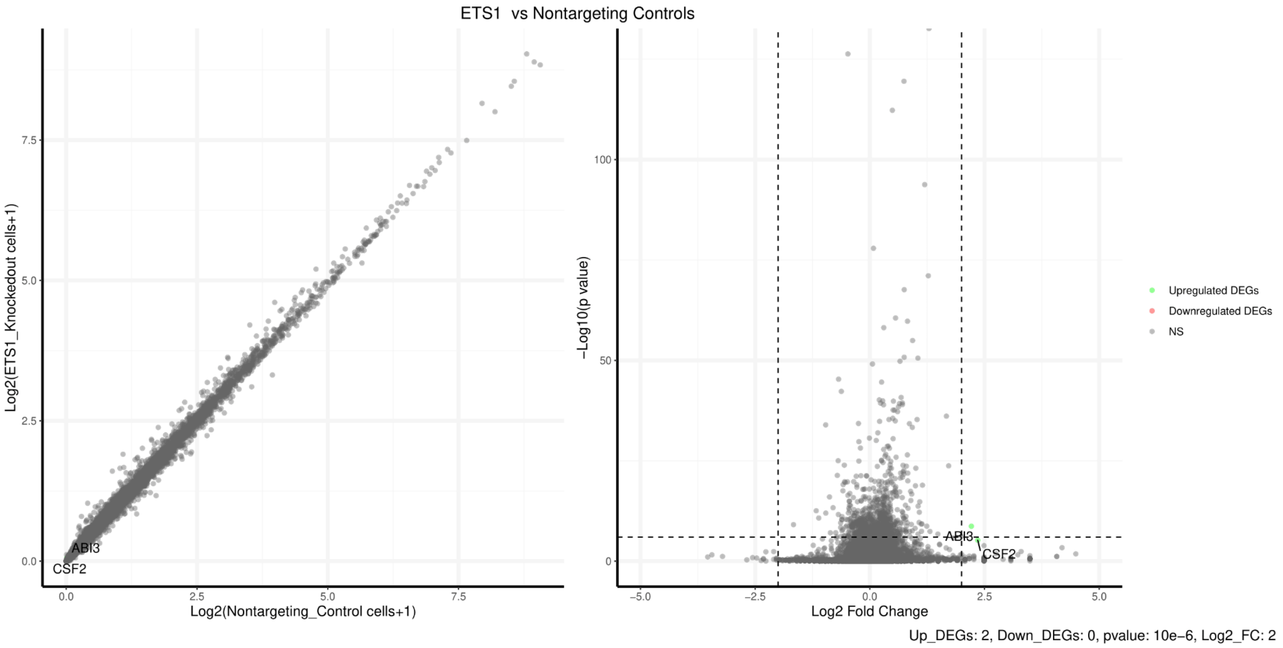
ETS1 vs Nontargeting Controls
Log2(ETS1_Knockedout cells+1)
Log2(Nontargeting_Control cells+1)
ABI3
CSF2
−Log10(p value)
Log2 Fold Change
Upregulated DEGs
Downregulated DEGs
NS
Up_DEGs: 2, Down_DEGs: 0, pvalue: 10e−6, Log2_FC: 2

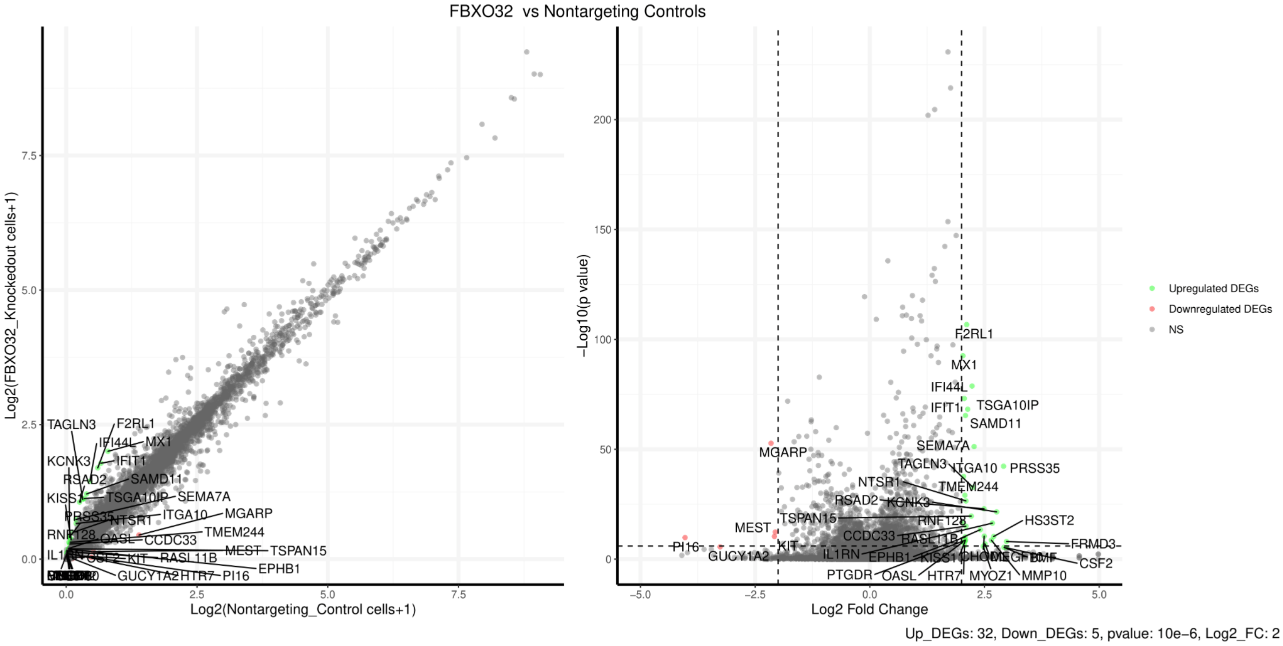
FBXO32 vs Nontargeting Controls
Log2(FBXO32_Knockedout cells+1)
Log2(Nontargeting_Control cells+1)
−Log10(p value)
Log2 Fold Change
Upregulated DEGs
Downregulated DEGs
NS
Up_DEGs: 32, Down_DEGs: 5, pvalue: 10e−6, Log2_FC: 2

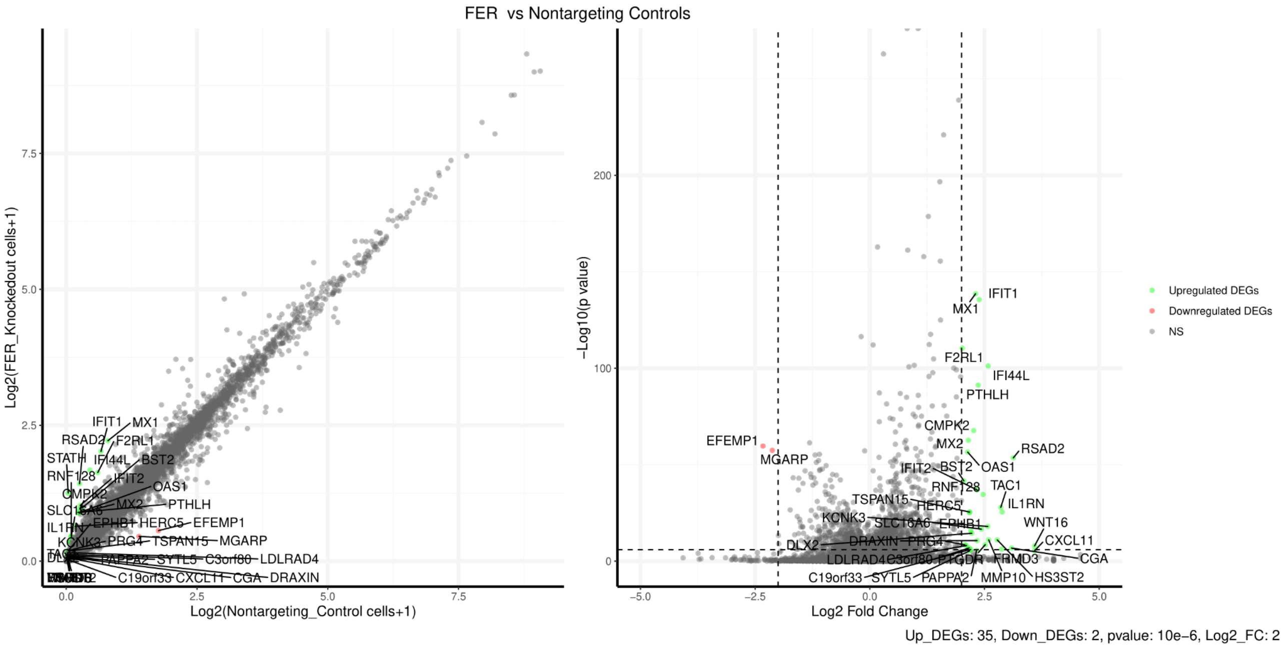
FER vs Nontargeting Controls
Log2(FER_Knockedout cells+1)
Log2(Nontargeting_Control cells+1)
−Log10(p value)
Log2 Fold Change
Upregulated DEGs
Downregulated DEGs
NS
Up_DEGs: 35, Down_DEGs: 2, pvalue: 10e−6, Log2_FC: 2

FERMT2 vs Nontargeting Controls

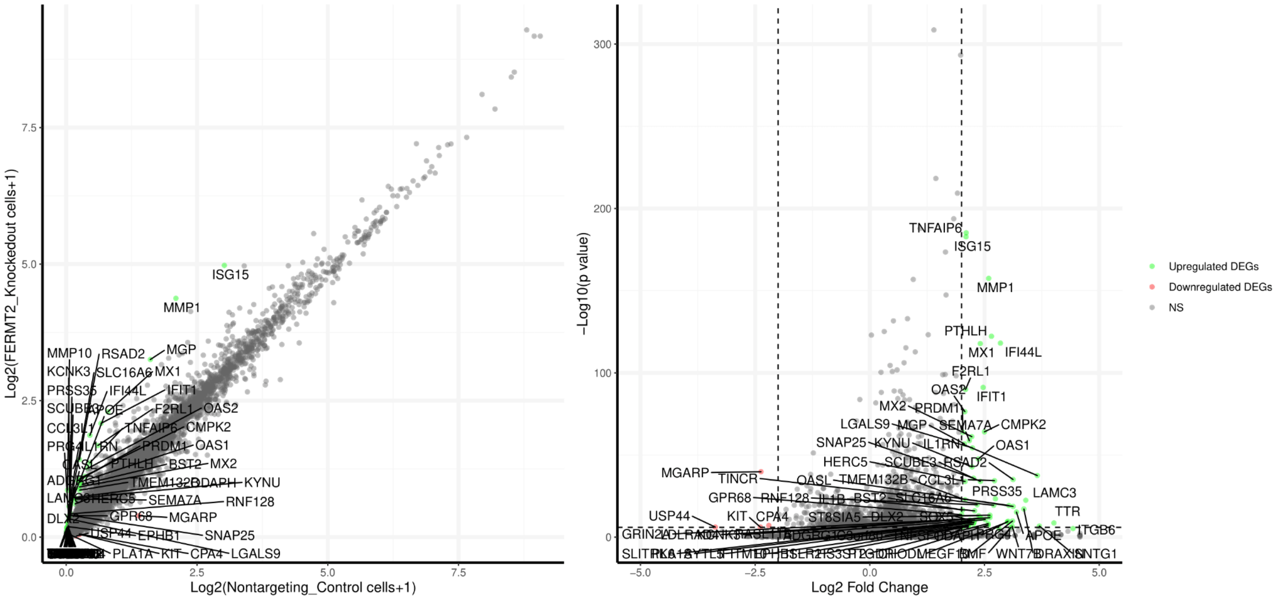

Up_DEGs: 63, Down_DEGs: 4, pvalue: 10e−6, Log2_FC: 2

FMNL2 vs Nontargeting Controls

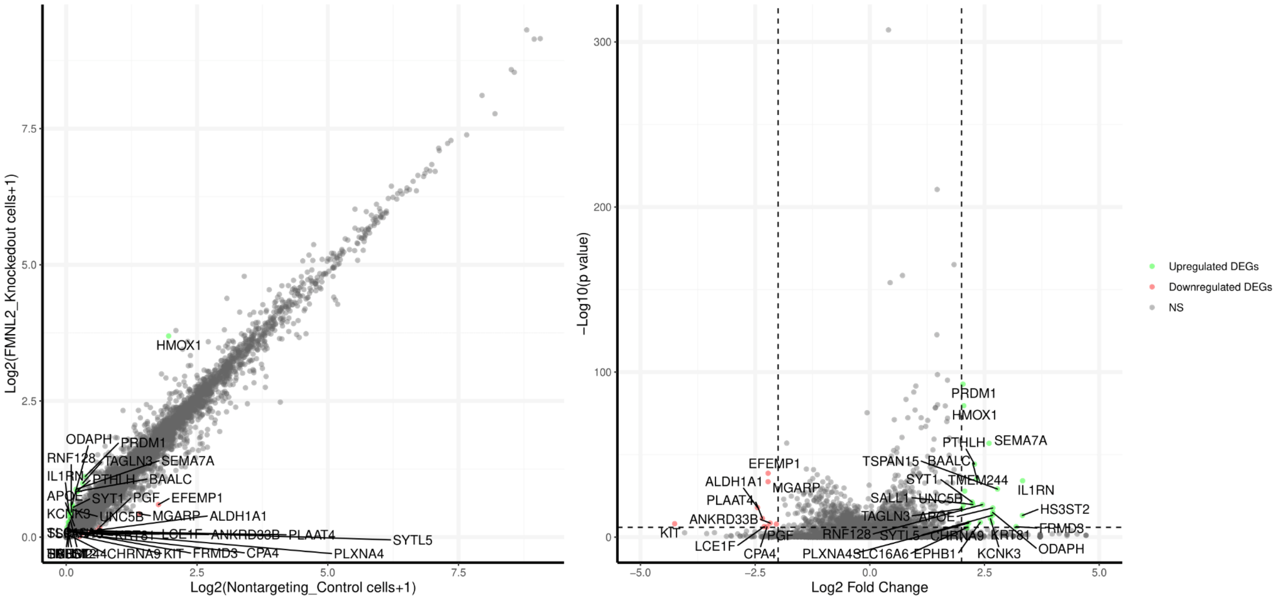

Up_DEGs: 24, Down_DEGs: 9, pvalue: 10e−6, Log2_FC: 2

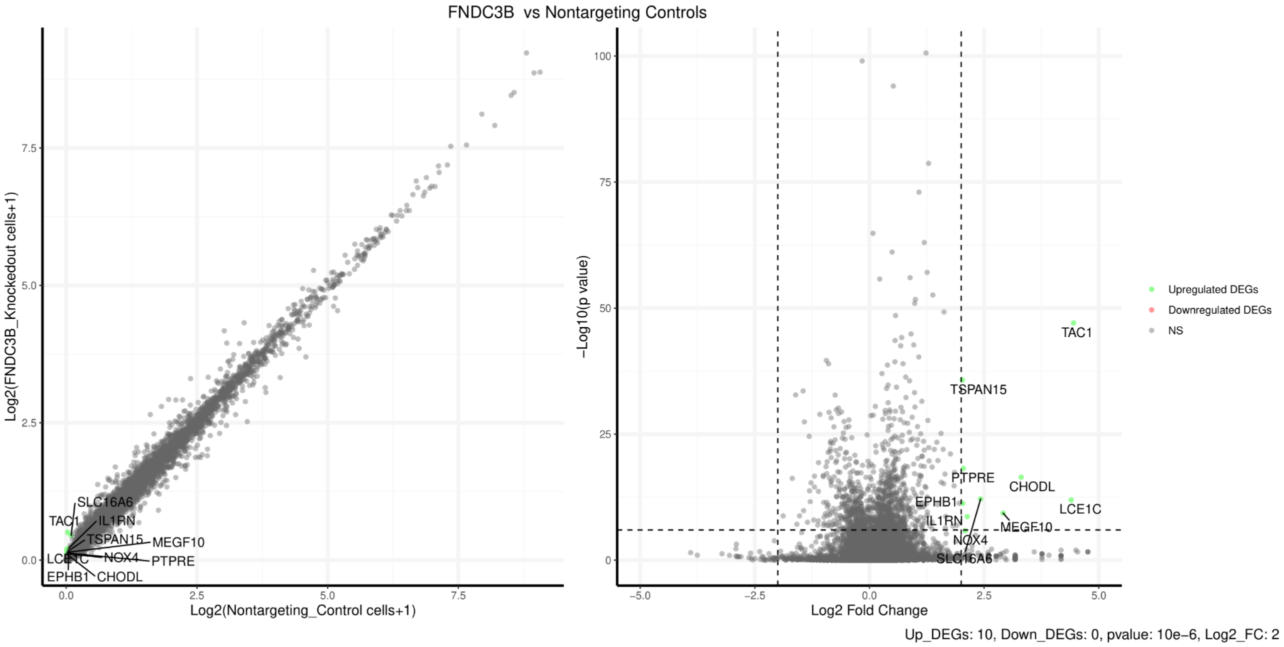
FNDC3B vs Nontargeting Controls
Log2(FNDC3B_Knockedout cells+1)
Log2(Nontargeting_Control cells+1)
SLC16A6
TAC1
IL1RN
TSPAN15
MEGF10
LCE1C
NOX4
PTPRE
EPHB1
CHODL
−Log10(p value)
Log2 Fold Change
Upregulated DEGs
Downregulated DEGs
NS
Up_DEGs: 10, Down_DEGs: 0, pvalue: 10e−6, Log2_FC: 2

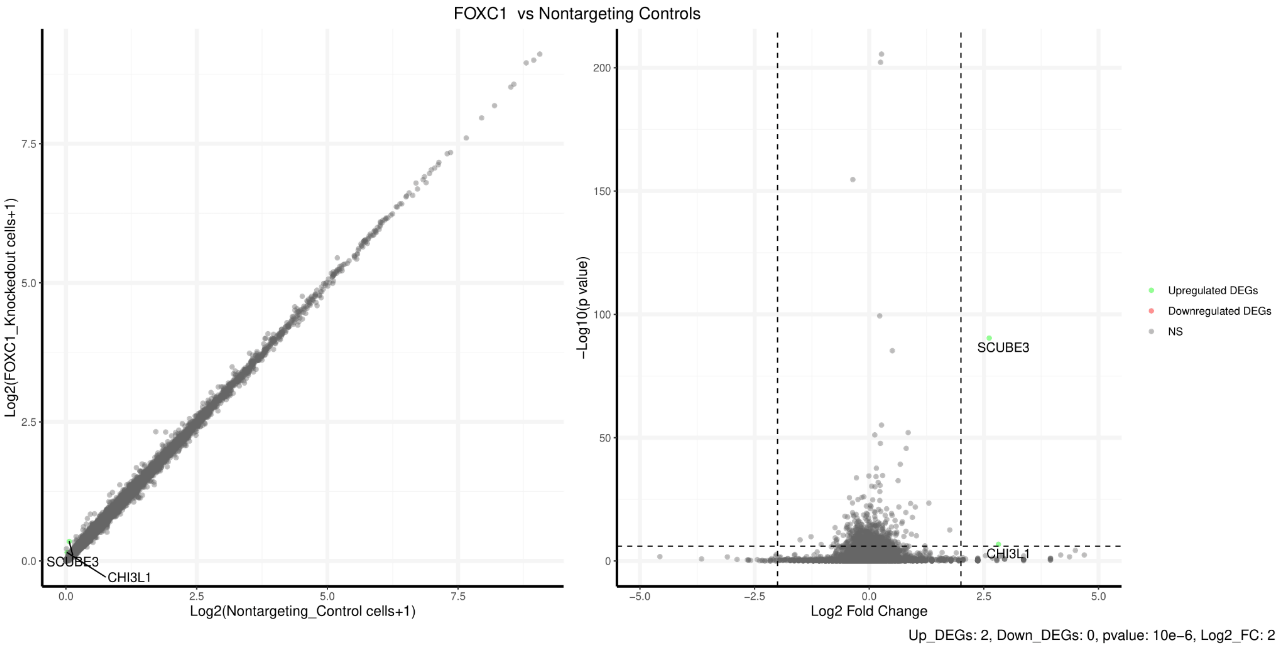
FOXC1 vs Nontargeting Controls
Log2(FOXC1_Knockedout cells+1)
Log2(Nontargeting_Control cells+1)
SCUBE3
CHI3L1
−Log10(p value)
Log2 Fold Change
Upregulated DEGs
Downregulated DEGs
NS
Up_DEGs: 2, Down_DEGs: 0, pvalue: 10e−6, Log2_FC: 2

GAS7 vs Nontargeting Controls

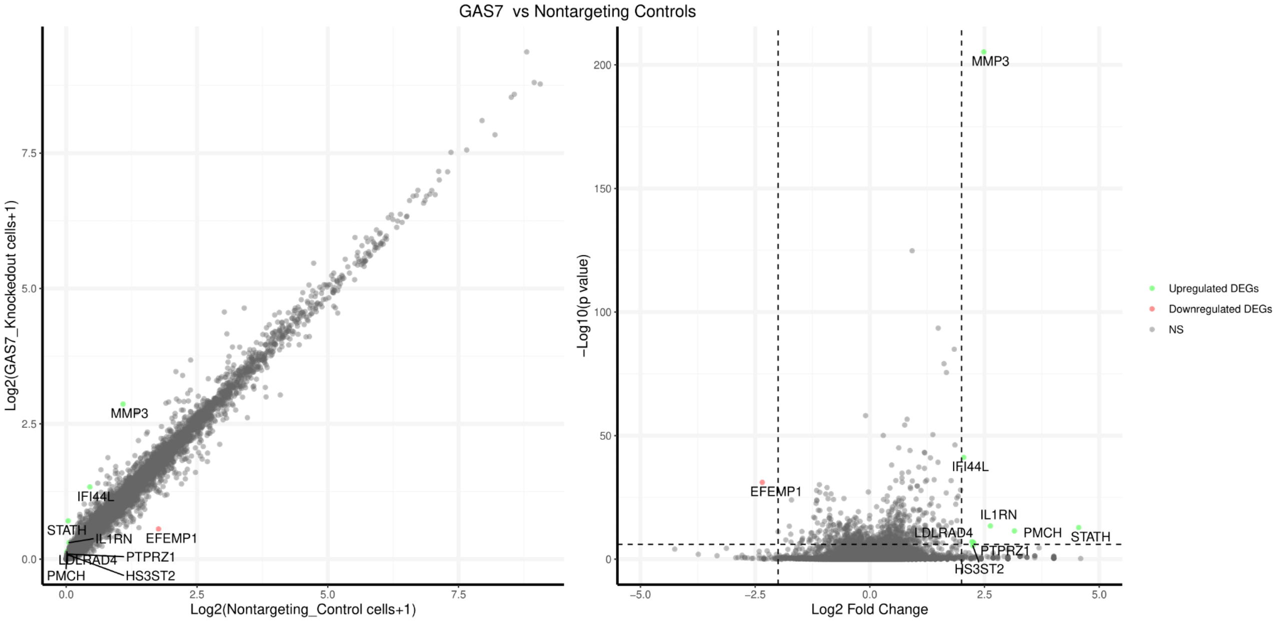

Up_DEGs: 8, Down_DEGs: 1, pvalue: 10e−6, Log2_FC: 2

GMDS vs Nontargeting Controls

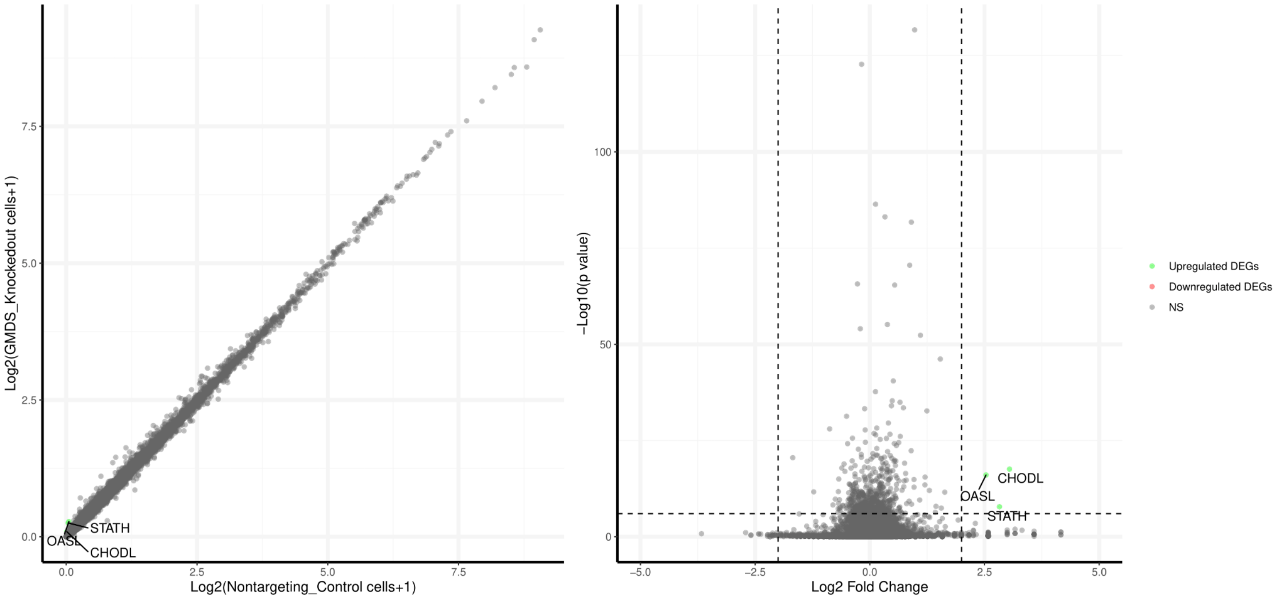

Up_DEGs: 3, Down_DEGs: 0, pvalue: 10e−6, Log2_FC: 2

GNB1L vs Nontargeting Controls

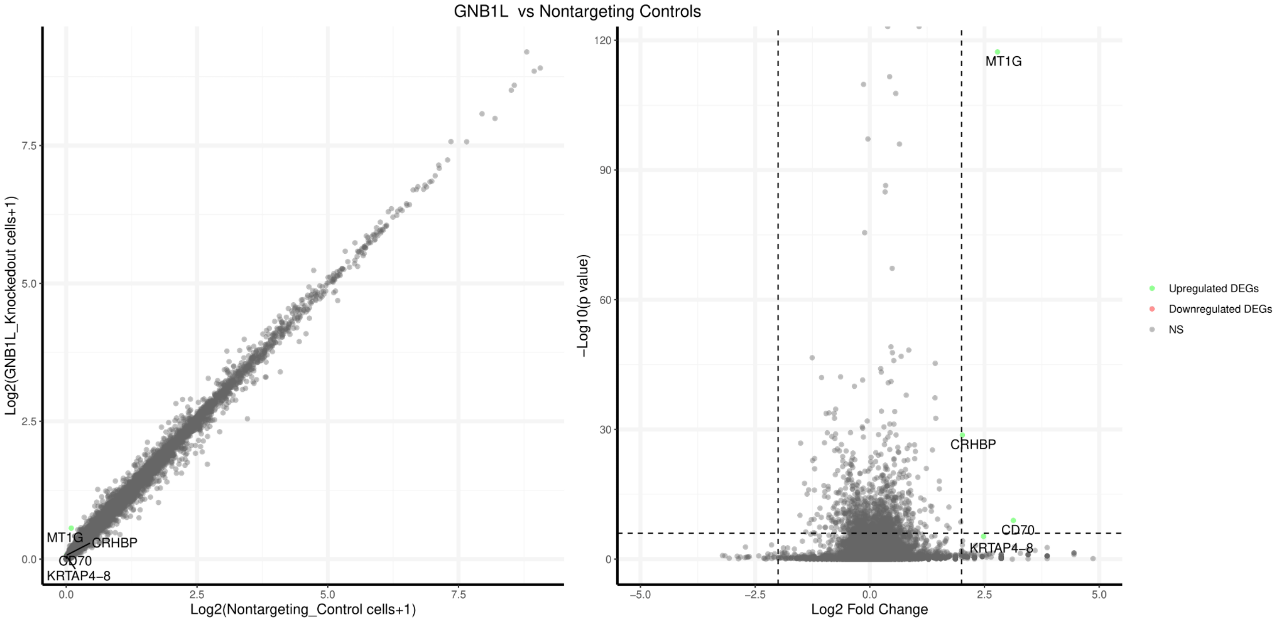

Up_DEGs: 4, Down_DEGs: 0, pvalue: 10e−6, Log2_FC: 2

KALRN vs Nontargeting Controls

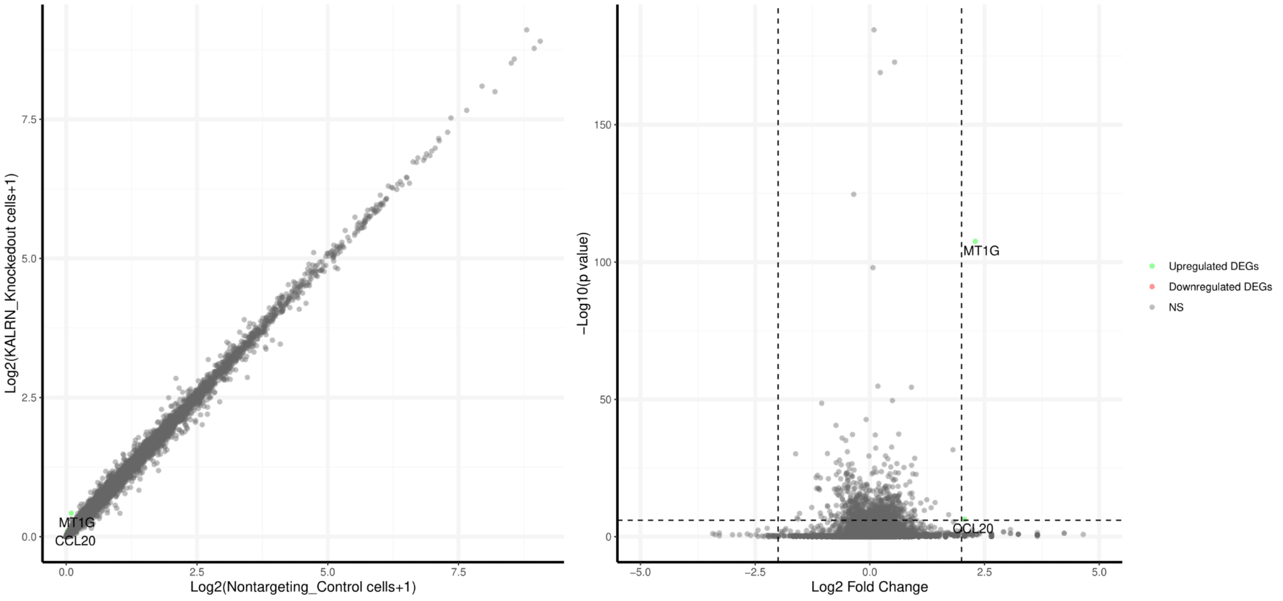

Up_DEGs: 2, Down_DEGs: 0, pvalue: 10e−6, Log2_FC: 2

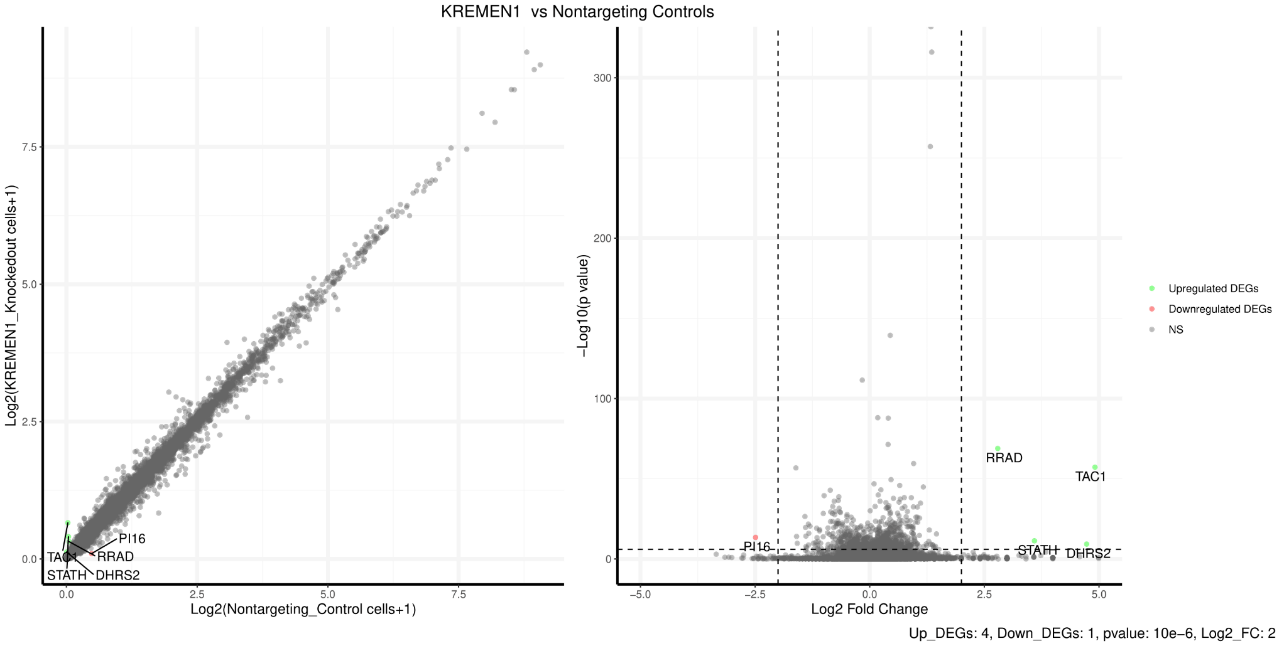
KREMEN1 vs Nontargeting Controls
Log2(KREMEN1_Knockedout cells+1)
Log2(Nontargeting_Control cells+1)
PI16
TAC1
RRAD
STATH
DHRS2
−Log10(p value)
Log2 Fold Change
Upregulated DEGs
Downregulated DEGs
NS
Up_DEGs: 4, Down_DEGs: 1, pvalue: 10e−6, Log2_FC: 2

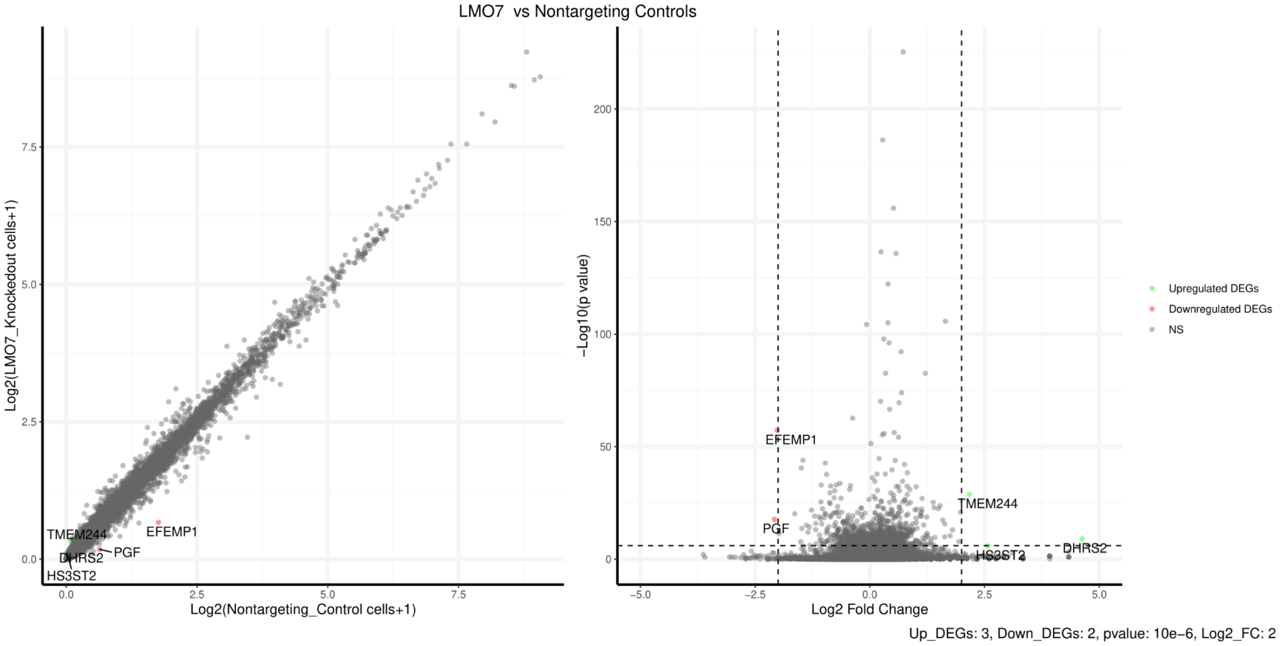
LMO7 vs Nontargeting Controls
Log2(LMO7_Knockedout cells+1)
Log2(Nontargeting_Control cells+1)
TMEM244
EFEMP1
PGF
DHRS2
HS3ST2
−Log10(p value)
Log2 Fold Change
Upregulated DEGs
Downregulated DEGs
NS
Up_DEGs: 3, Down_DEGs: 2, pvalue: 10e−6, Log2_FC: 2

LMX1B vs Nontargeting Controls

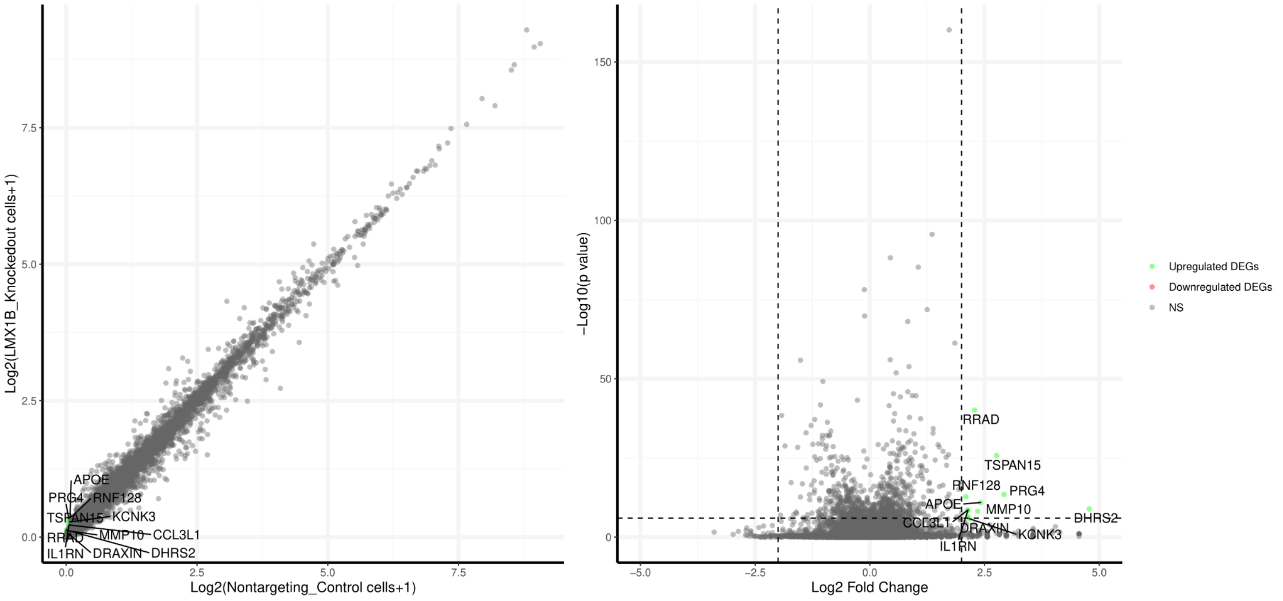

Up_DEGs: 11, Down_DEGs: 0, pvalue: 10e−6, Log2_FC: 2

LTBP2 vs Nontargeting Controls

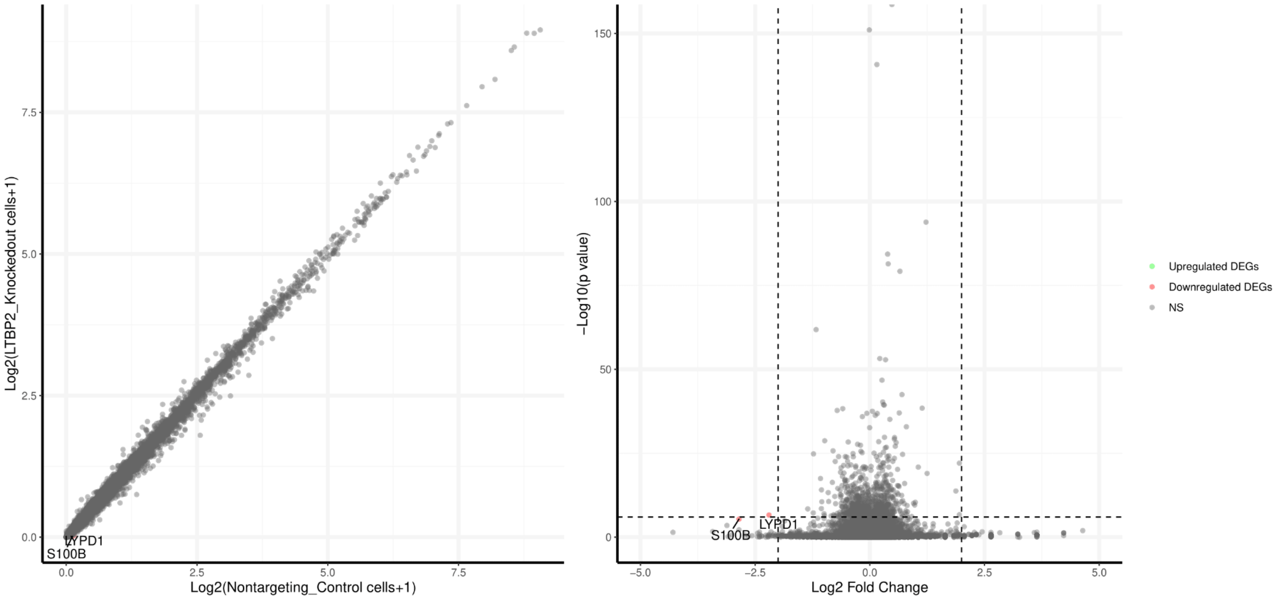

Up_DEGs: 0, Down_DEGs: 2, pvalue: 10e−6, Log2_FC: 2

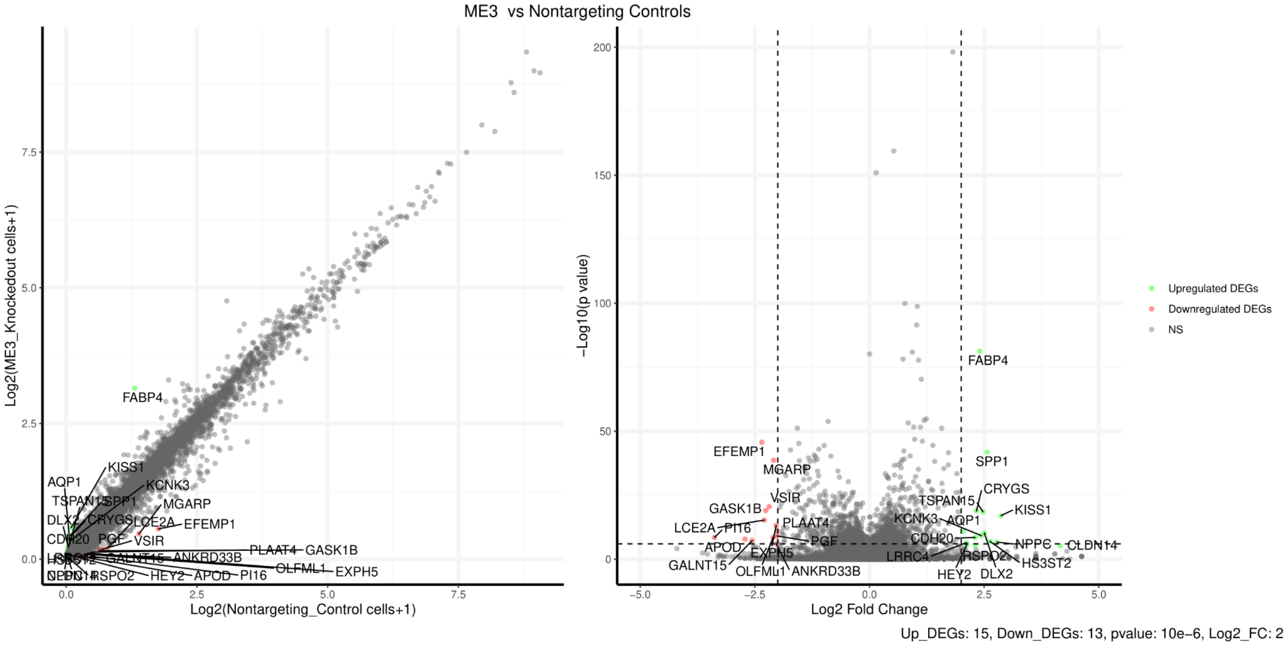
ME3 vs Nontargeting Controls
Log2(ME3_Knockedout cells+1)
Log2(Nontargeting_Control cells+1)
FABP4
KISS1
AQP1
KCNK3
MGARP
EFEMP1
VSIR
PGF
PLAAT4
GASK1B
ANKRD33B
OLFML1
EXPH5
HEY2
APOD
PI16
RSPO2
GALNT15
−Log10(p value)
Log2 Fold Change
SPP1
CRYGS
TSPAN15
LCE2A
CDH20
NPPC
CLDN14
HS3ST2
DLX2
LRRC4
Upregulated DEGs
Downregulated DEGs
NS
Up_DEGs: 15, Down_DEGs: 13, pvalue: 10e−6, Log2_FC: 2

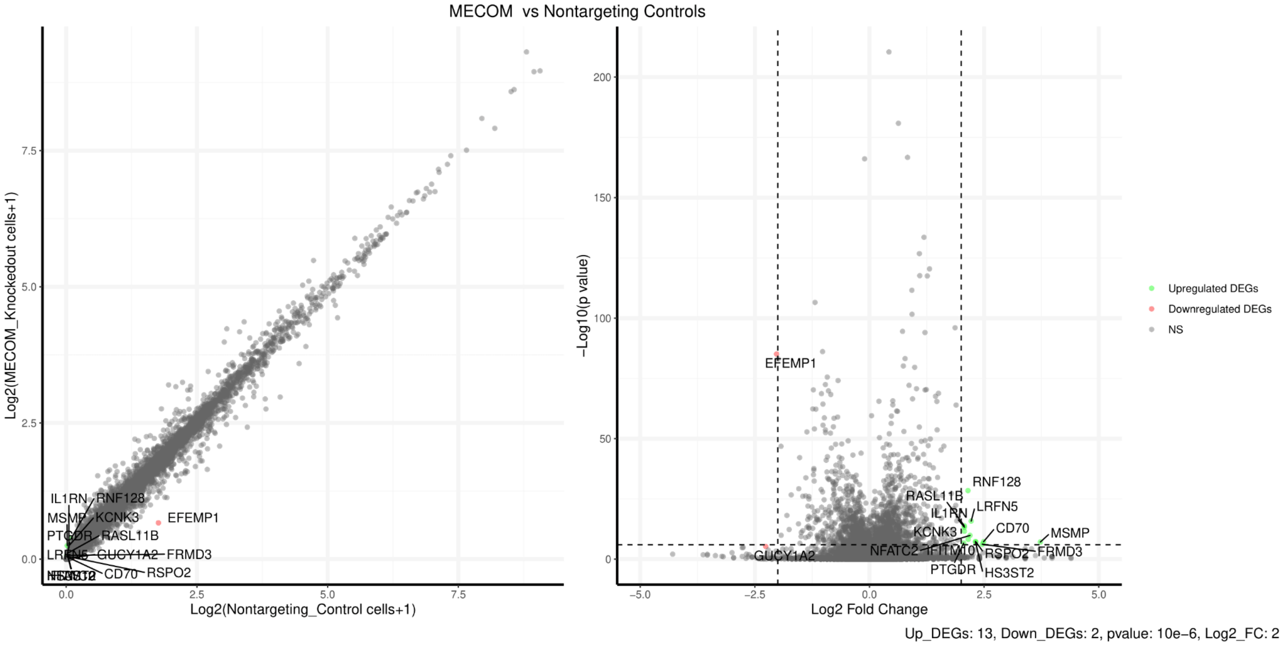
MECOM vs Nontargeting Controls
Log2(MECOM_Knockedout cells+1)
Log2(Nontargeting_Control cells+1)
IL1RN
RNF128
MSMP
KCNK3
EFEMP1
PTGDR
RASL11B
LRFN5
GUCY1A2
FRMD3
CD70
RSPO2
−Log10(p value)
Log2 Fold Change
NFATC2
IFITM10
HS3ST2
Upregulated DEGs
Downregulated DEGs
NS
Up_DEGs: 13, Down_DEGs: 2, pvalue: 10e−6, Log2_FC: 2

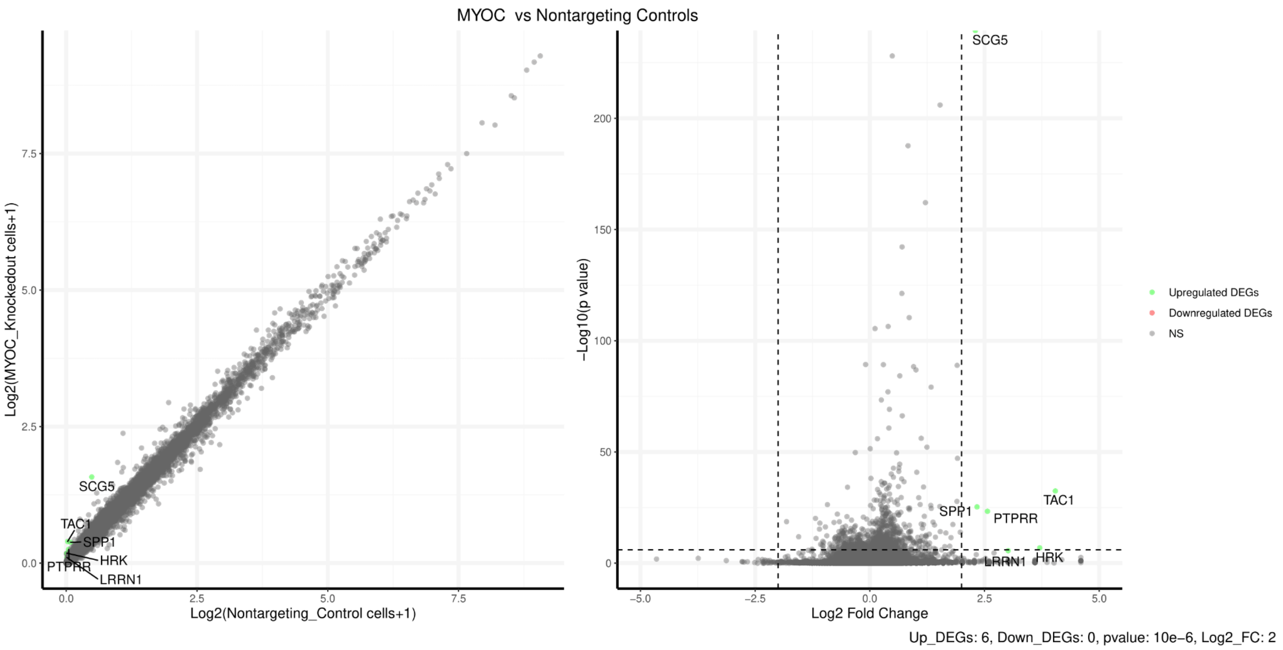
MYOC vs Nontargeting Controls
Log2(MYOC_Knockedout cells+1)
Log2(Nontargeting_Control cells+1)
SCG5
TAC1
SPP1
HRK
PTPRR
LRRN1
−Log10(p value)
Log2 Fold Change
PTPRR
Upregulated DEGs
Downregulated DEGs
NS
Up_DEGs: 6, Down_DEGs: 0, pvalue: 10e−6, Log2_FC: 2

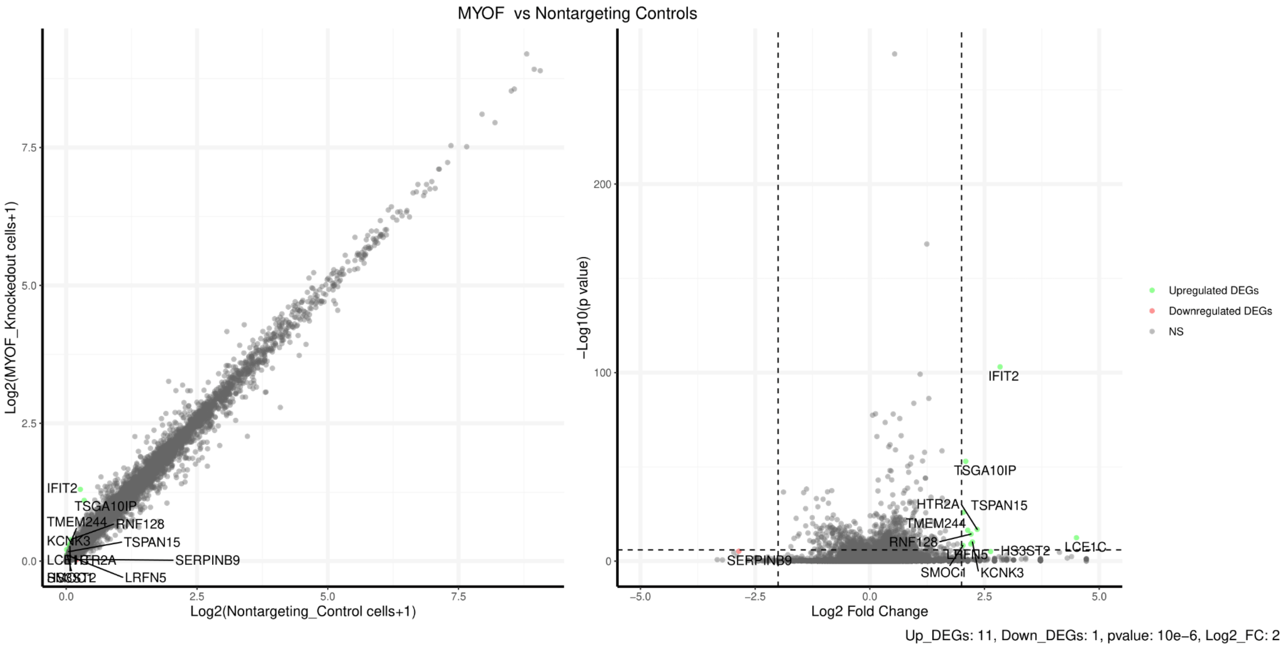
MYOF vs Nontargeting Controls
Log2(MYOF_Knockedout cells+1)
Log2(Nontargeting_Control cells+1)
IFIT2
TSGA10IP
TMEM244
RNF128
KCNK3
TSPAN15
SERPINB9
LRFN5
−Log10(p value)
Log2 Fold Change
HTR2A
TSPAN15
SMOC1
KCNK3
HS3ST2
LCE1C
Upregulated DEGs
Downregulated DEGs
NS
Up_DEGs: 11, Down_DEGs: 1, pvalue: 10e−6, Log2_FC: 2

PARD3B vs Nontargeting Controls

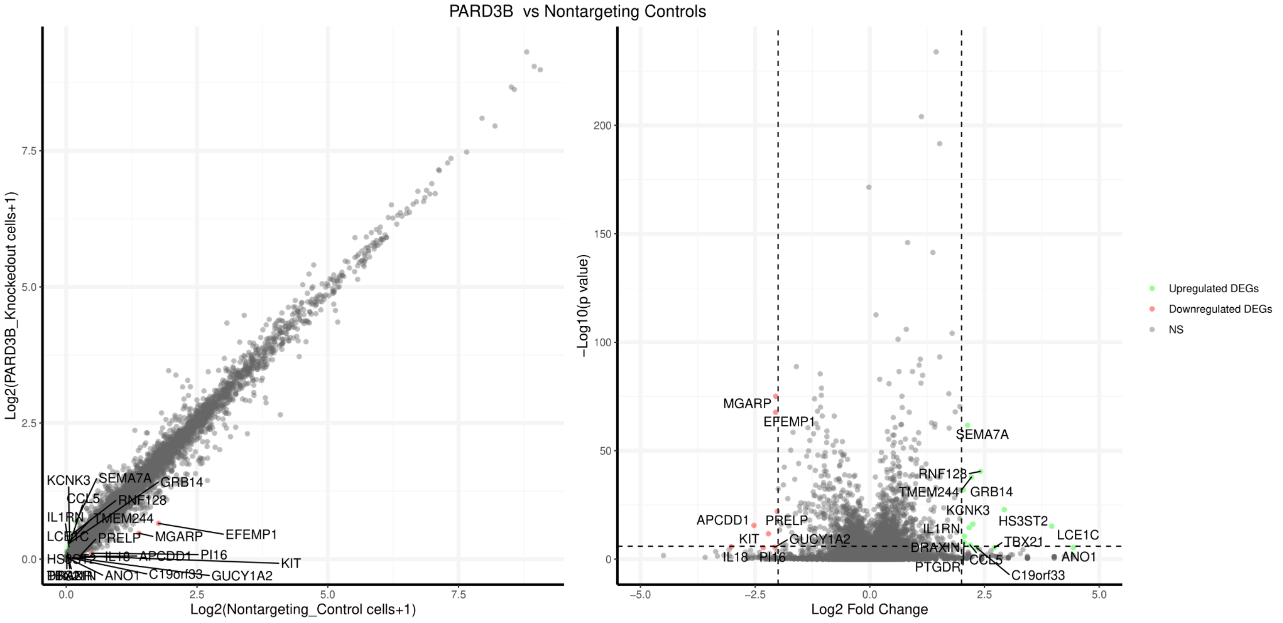

Up_DEGs: 14, Down_DEGs: 8, pvalue: 10e−6, Log2_FC: 2

PDE7B vs Nontargeting Controls

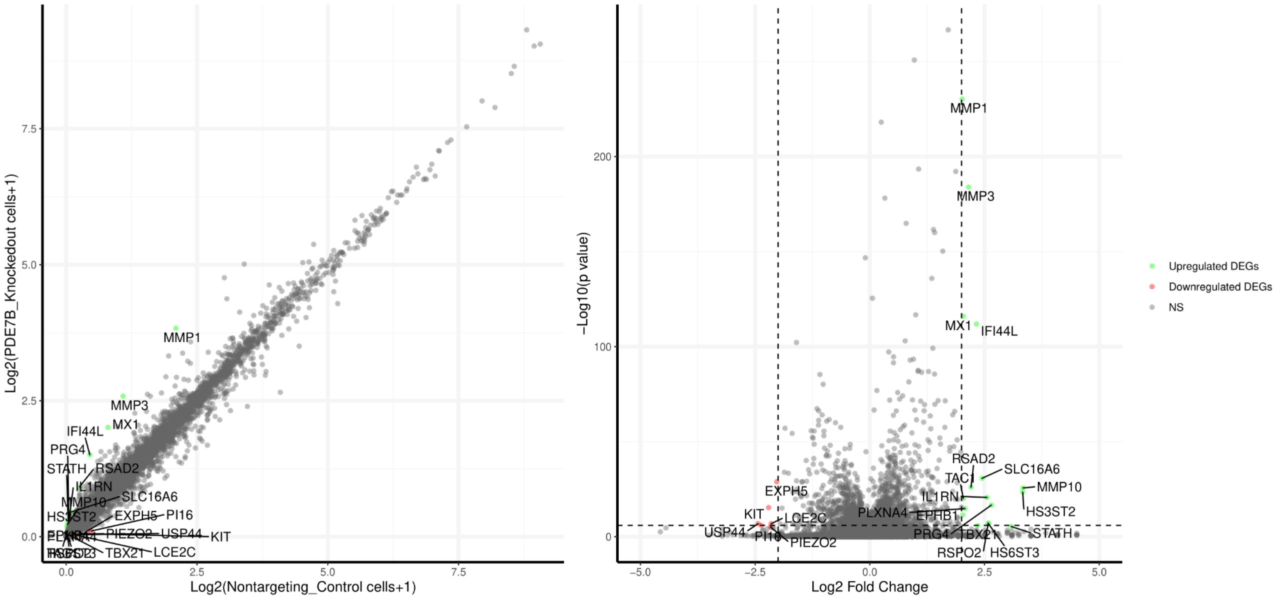

Up_DEGs: 17, Down_DEGs: 6, pvalue: 10e−6, Log2_FC: 2

PKHD1 vs Nontargeting Controls

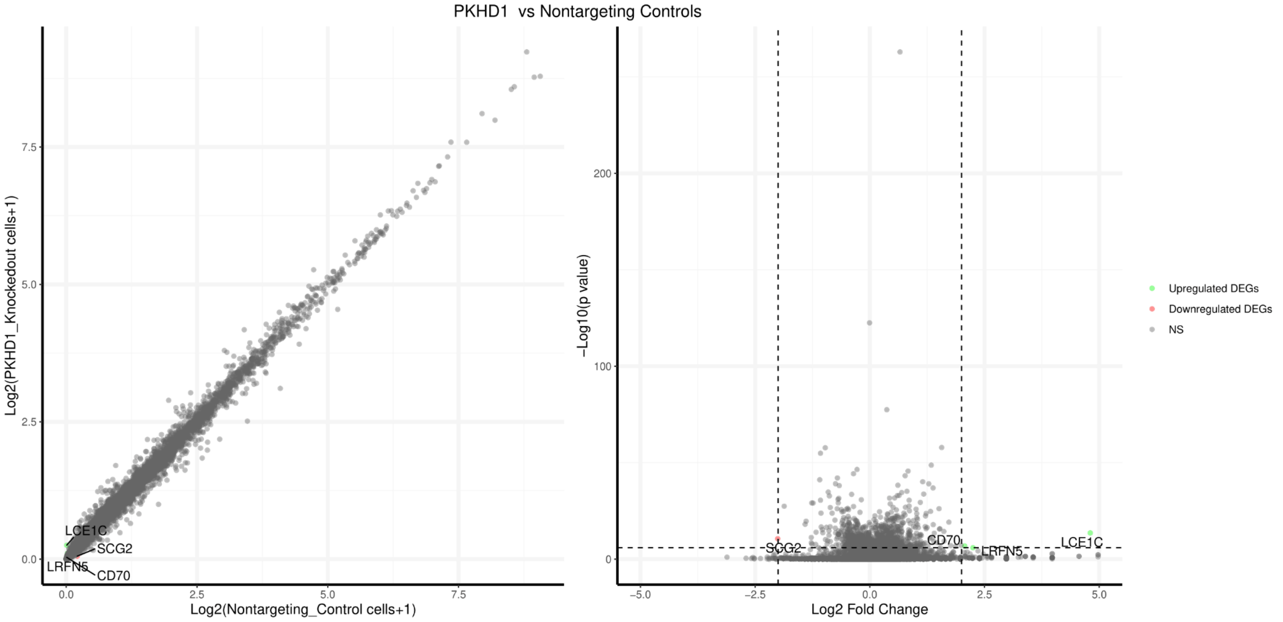

Up_DEGs: 3, Down_DEGs: 1, pvalue: 10e−6, Log2_FC: 2

PLEKHA7 vs Nontargeting Controls

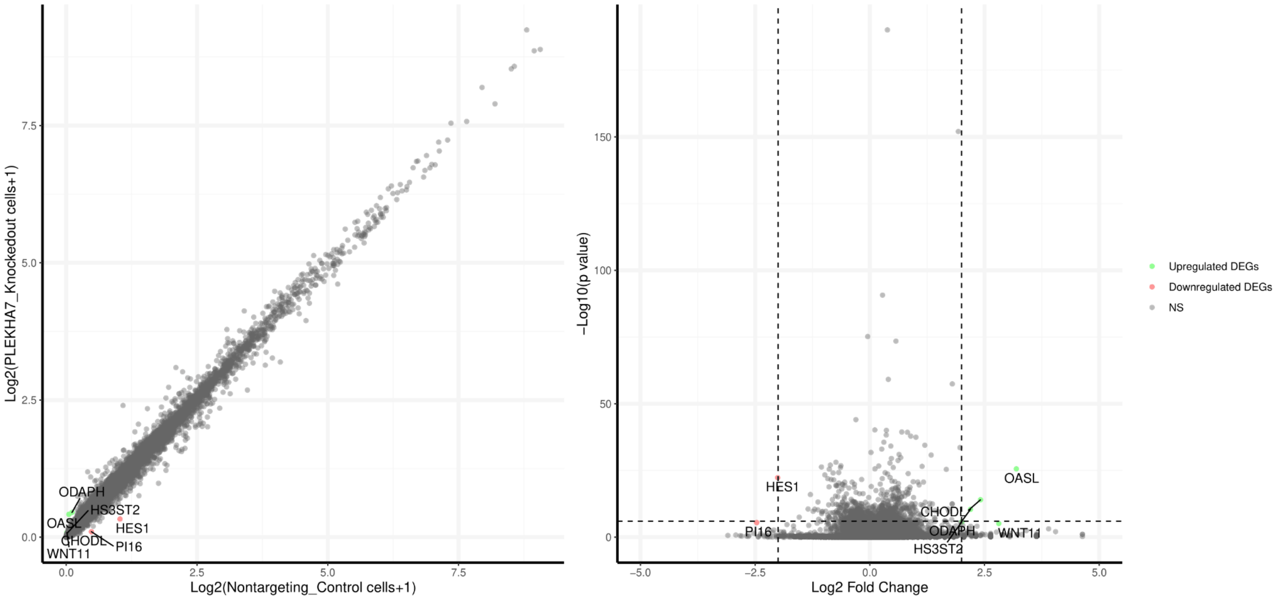

Up_DEGs: 5, Down_DEGs: 2, pvalue: 10e−6, Log2_FC: 2

PRSS23 vs Nontargeting Controls

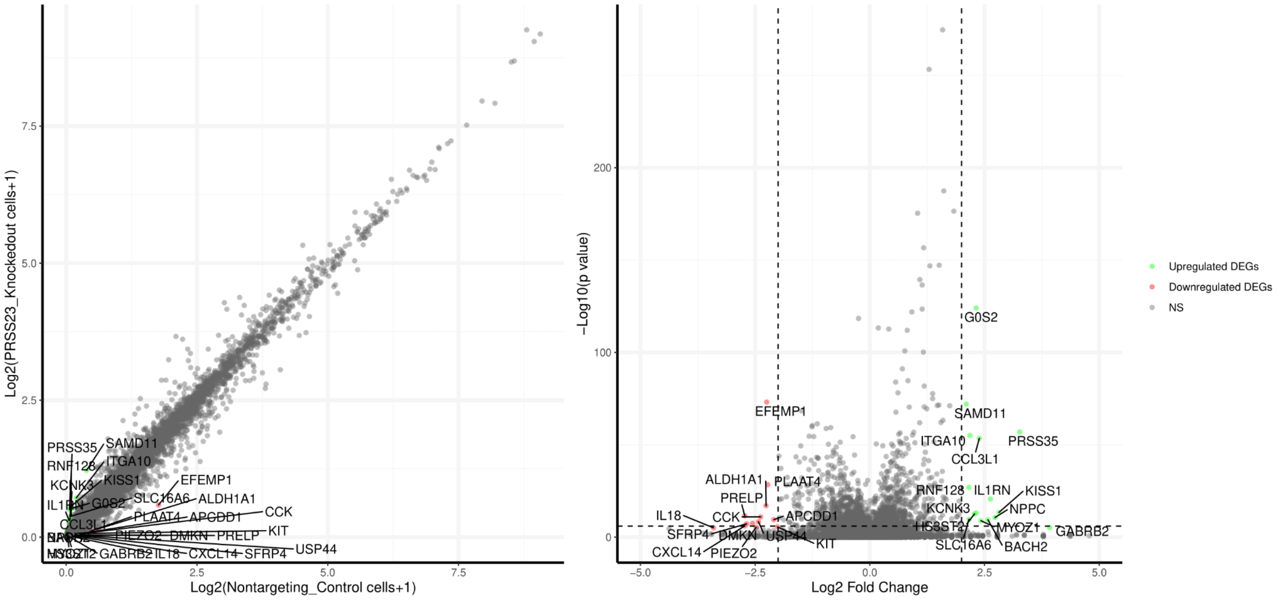

Up_DEGs: 15, Down_DEGs: 13, pvalue: 10e−6, Log2_FC: 2

PTPRJ vs Nontargeting Controls

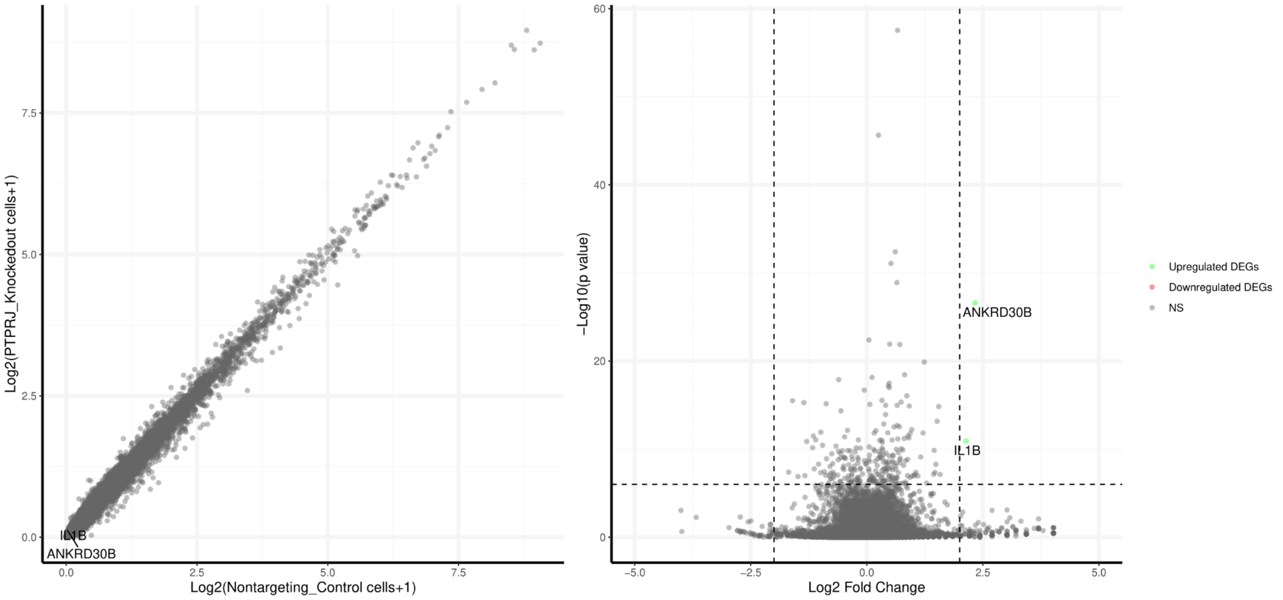

Up_DEGs: 2, Down_DEGs: 0, pvalue: 10e−6, Log2_FC: 2

RALGPS1 vs Nontargeting Controls

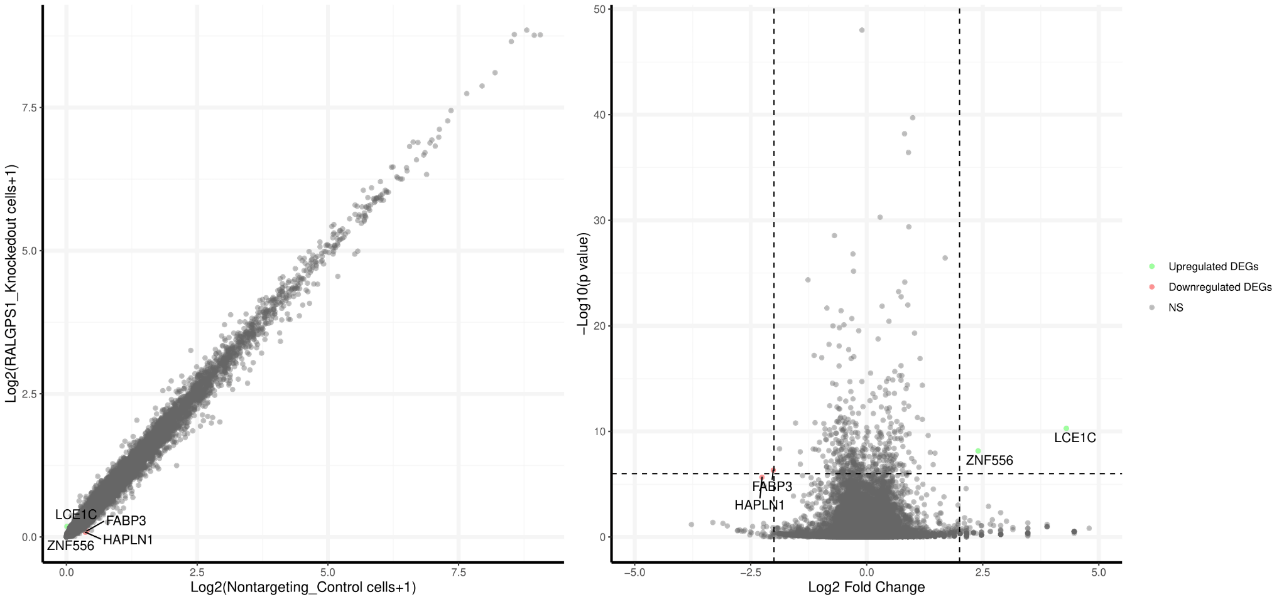

Up_DEGs: 2, Down_DEGs: 2, pvalue: 10e−6, Log2_FC: 2

RUNX2 vs Nontargeting Controls

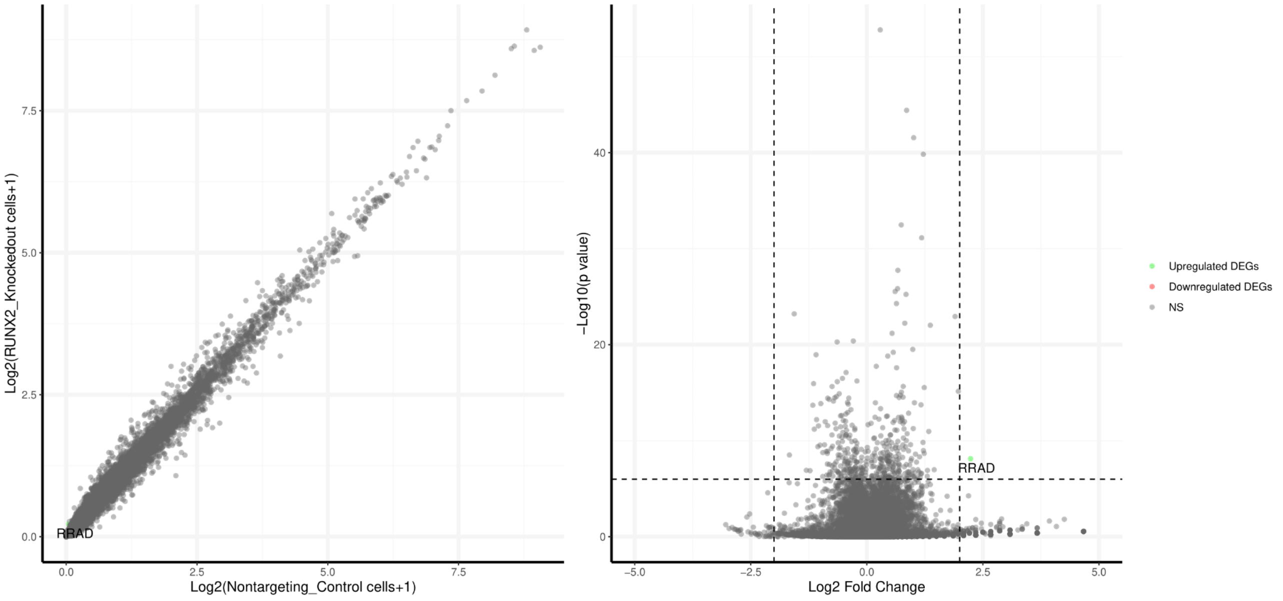

Up_DEGs: 1, Down_DEGs: 0, pvalue: 10e−6, Log2_FC: 2

SPTBN1 vs Nontargeting Controls

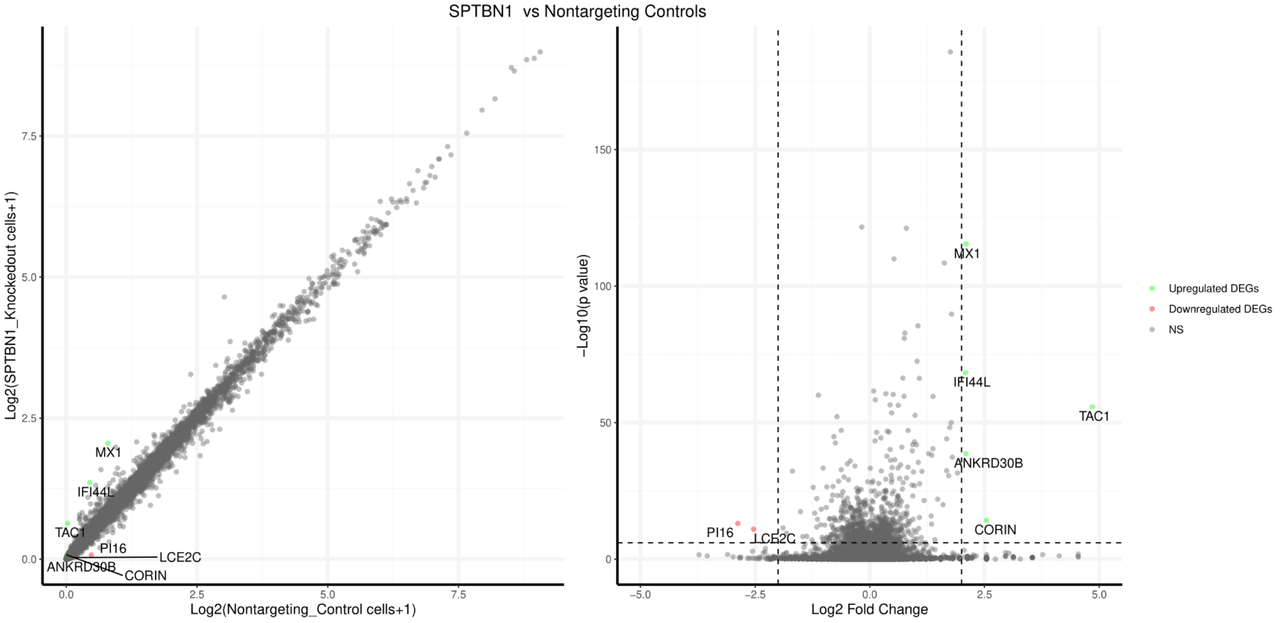

Up_DEGs: 5, Down_DEGs: 2, pvalue: 10e−6, Log2_FC: 2

TEK vs Nontargeting Controls

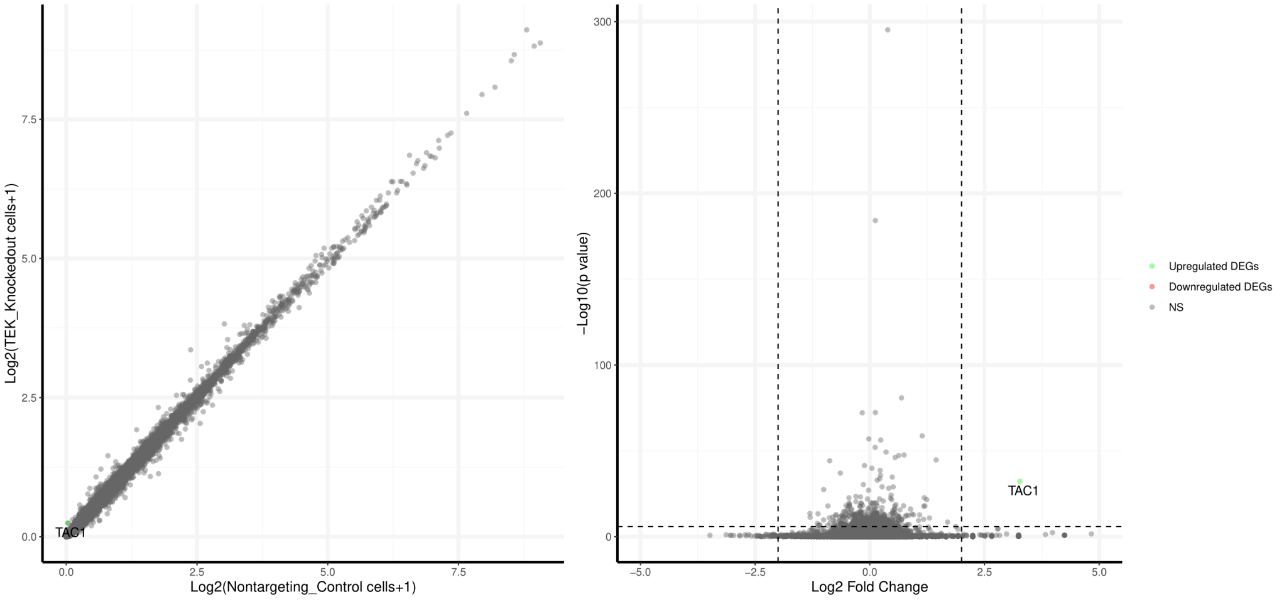

Up_DEGs: 1, Down_DEGs: 0, pvalue: 10e−6, Log2_FC: 2

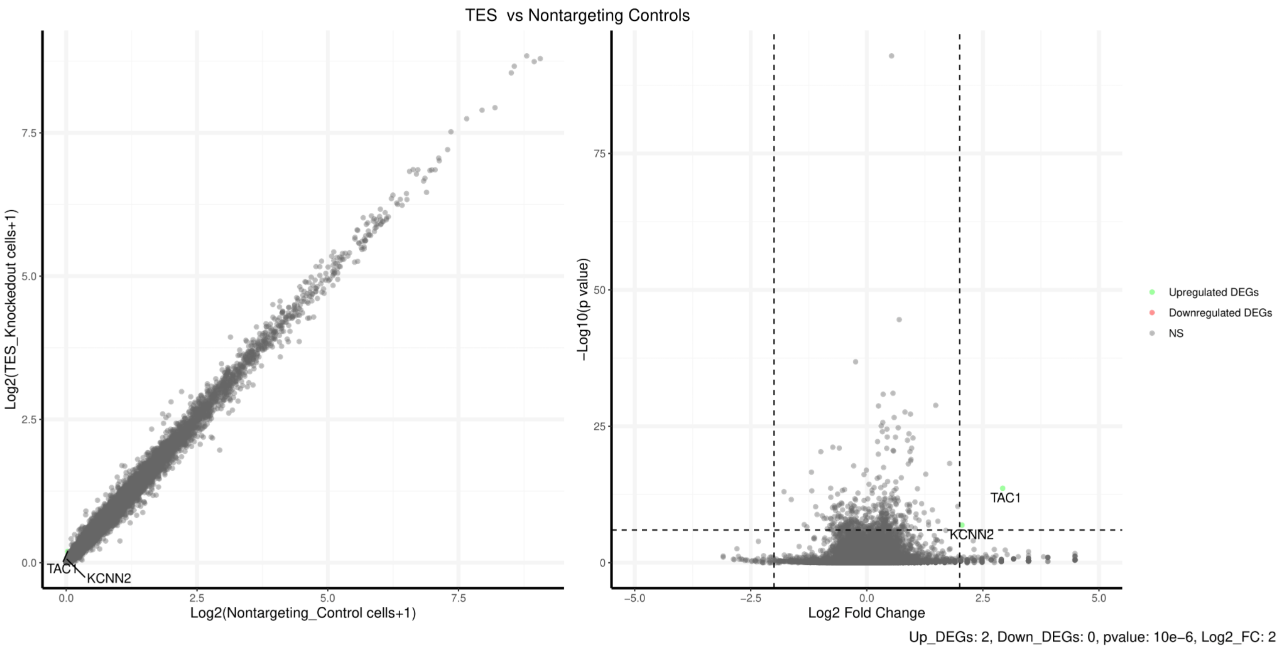

TES vs Nontargeting Controls
Log2(TES_Knockedout cells+1)
Log2(Nontargeting_Control cells+1)
TAC1
KCNN2
−Log10(p value)
Log2 Fold Change
Upregulated DEGs
Downregulated DEGs
NS
Up_DEGs: 2, Down_DEGs: 0, pvalue: 10e−6, Log2_FC: 2

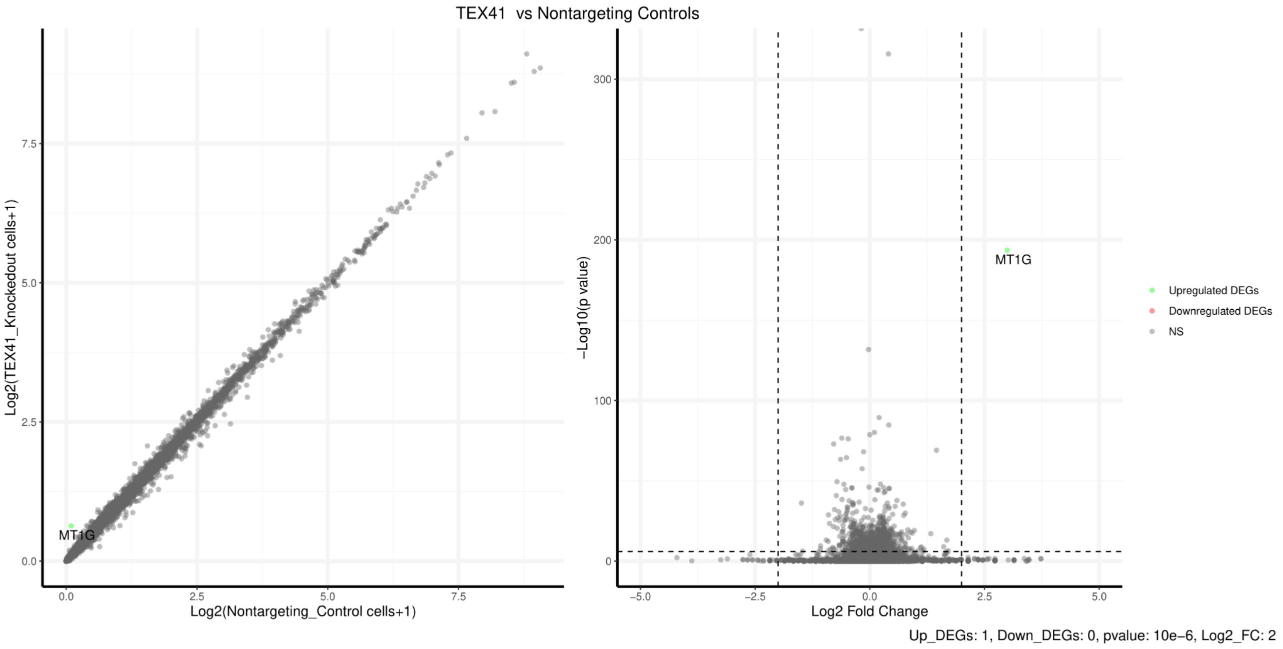

TEX41 vs Nontargeting Controls
Log2(TEX41_Knockedout cells+1)
Log2(Nontargeting_Control cells+1)
MT1G
−Log10(p value)
Log2 Fold Change
Upregulated DEGs
Downregulated DEGs
NS
Up_DEGs: 1, Down_DEGs: 0, pvalue: 10e−6, Log2_FC: 2

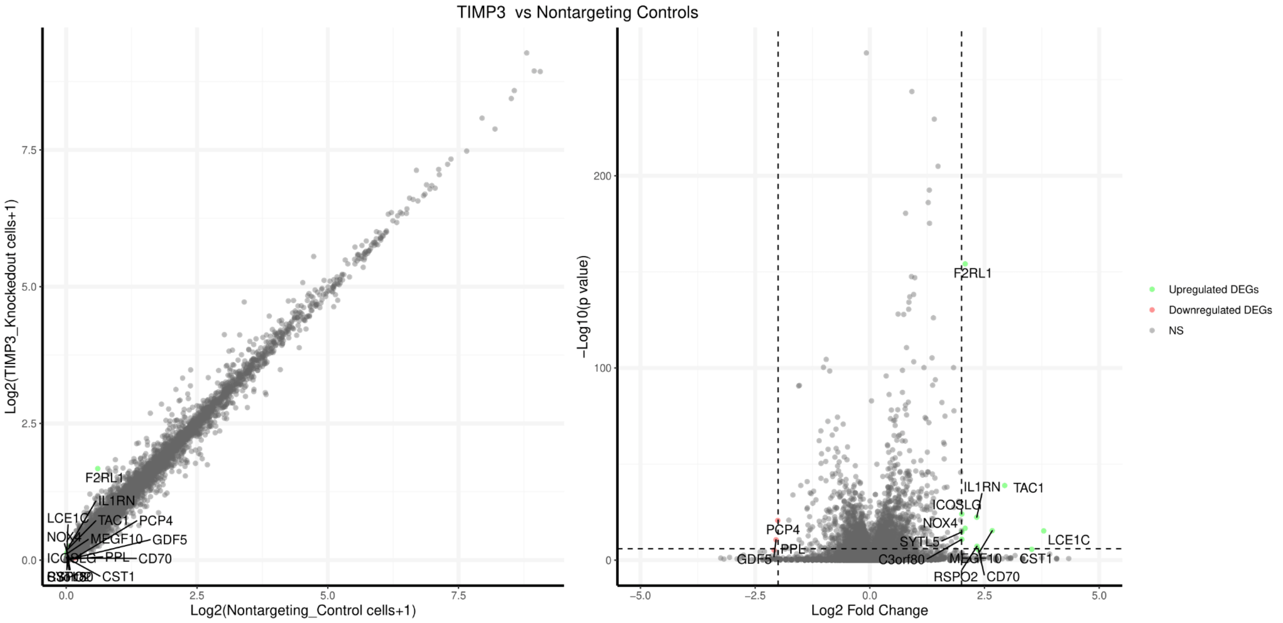

Up_DEGs: 12, Down_DEGs: 3, pvalue: 10e−6, Log2_FC: 2

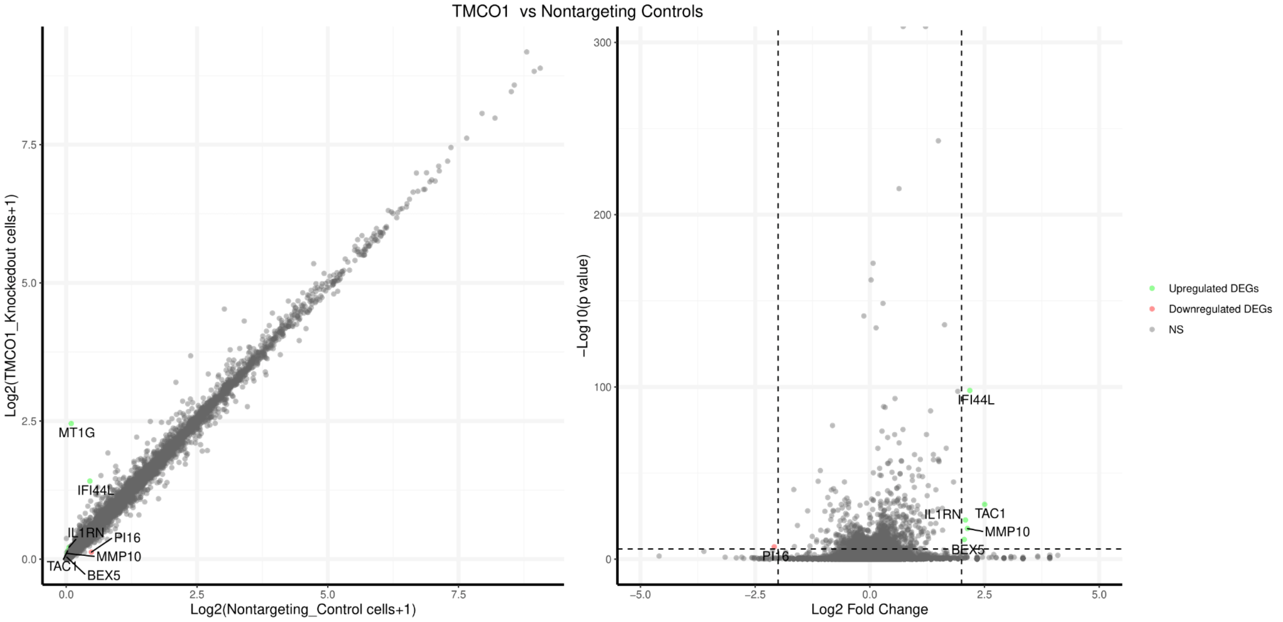

Up_DEGs: 6, Down_DEGs: 1, pvalue: 10e−6, Log2_FC: 2

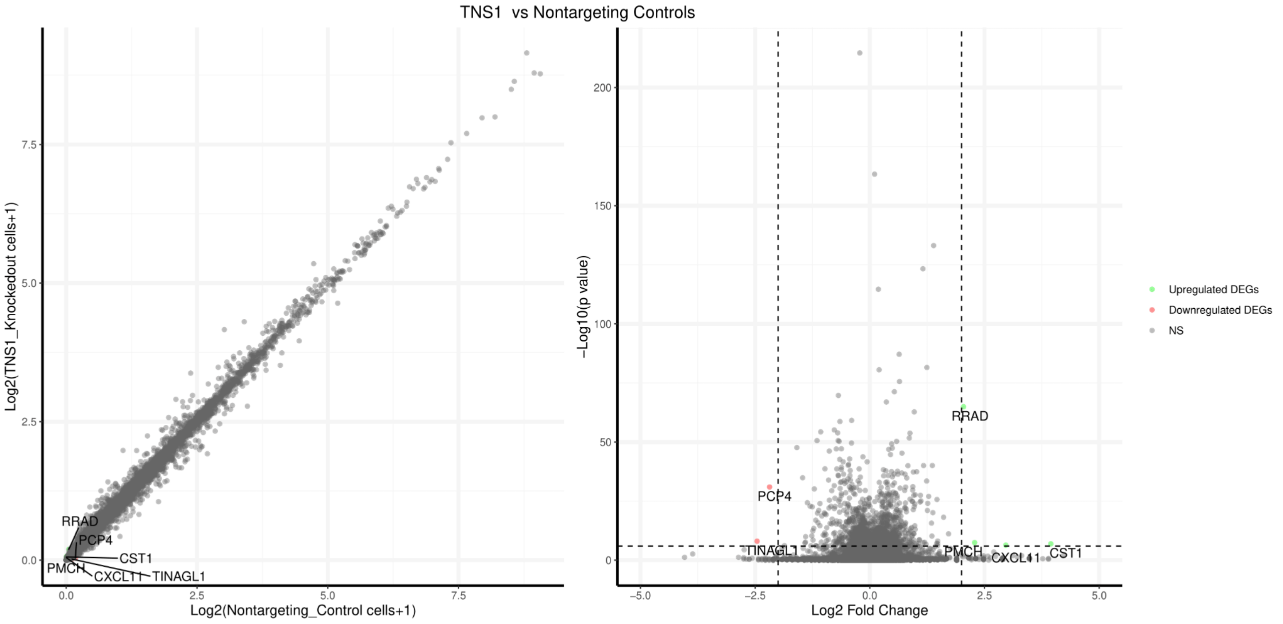

Up_DEGs: 4, Down_DEGs: 2, pvalue: 10e−6, Log2_FC: 2

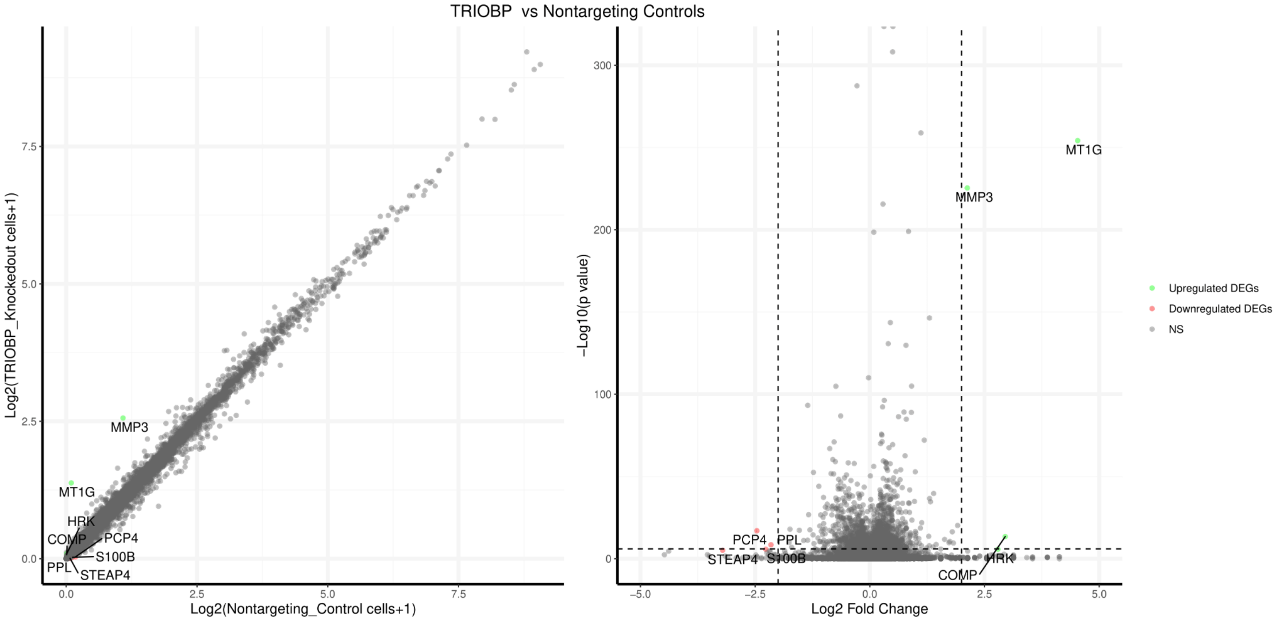

Up_DEGs: 4, Down_DEGs: 4, pvalue: 10e−6, Log2_FC: 2

TXNRD2 vs Nontargeting Controls

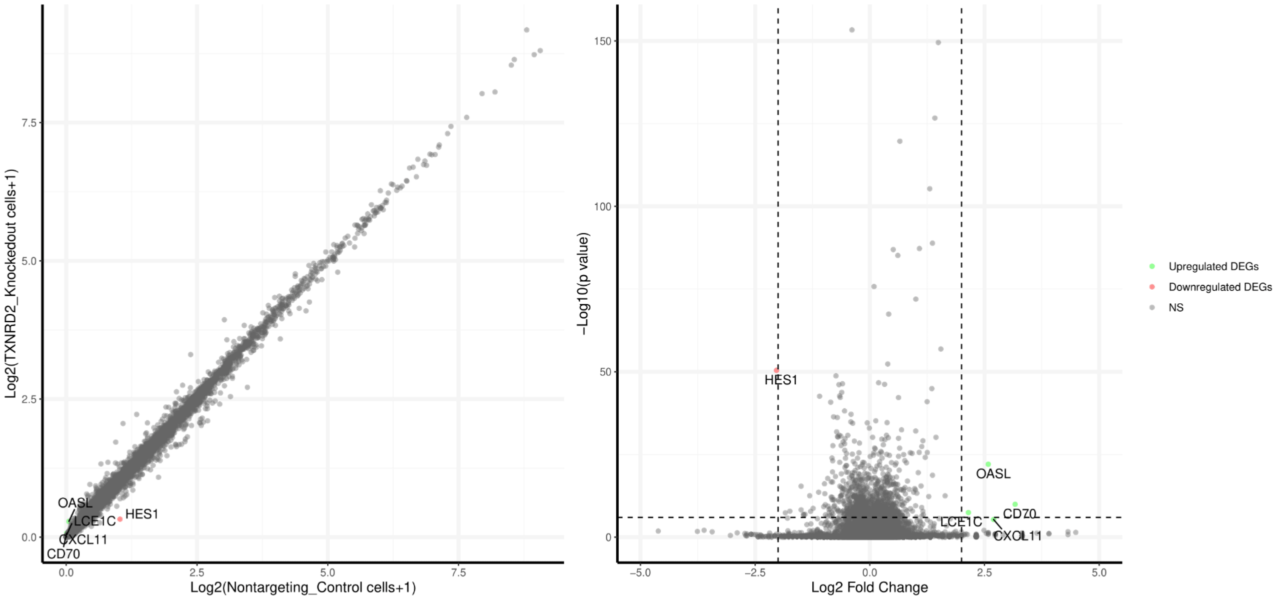

Up_DEGs: 4, Down_DEGs: 1, pvalue: 10e−6, Log2_FC: 2

ZNF280D vs Nontargeting Controls

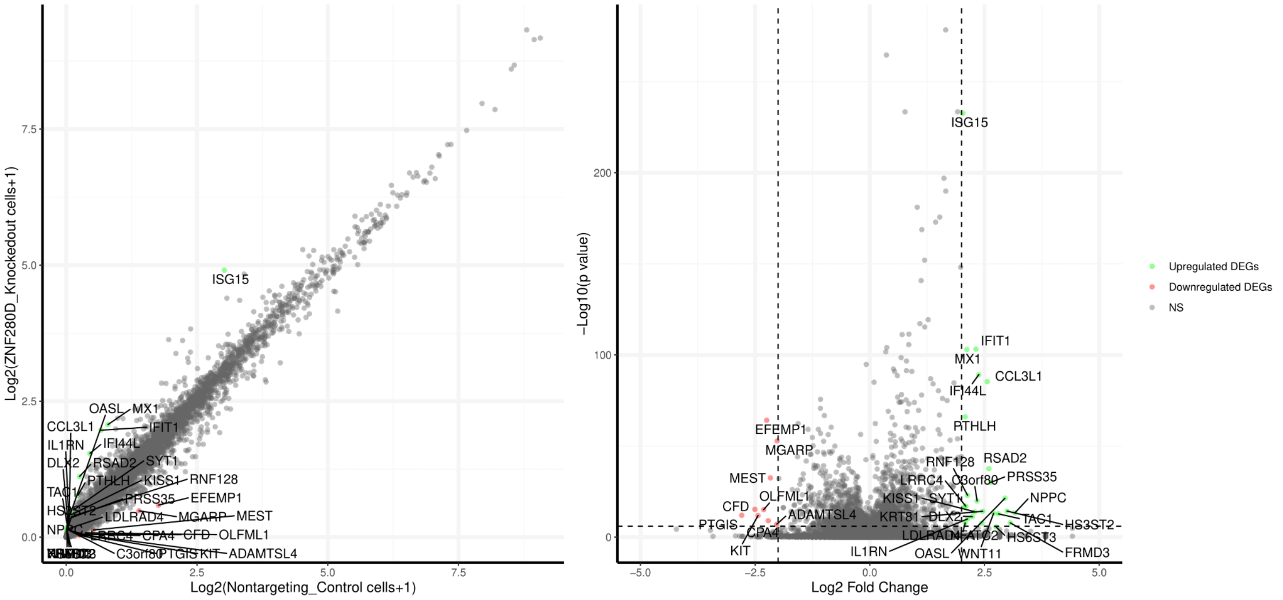

Up_DEGs: 25, Down_DEGs: 9, pvalue: 10e−6, Log2_FC: 2

Supplementary Figure 11:

Differential expressed genes between gene knockout and non-targeting control Beam A Cells. The volcano plots display the Log2 fold change versus -log$_{10}$(P value) for the gene-targeted cells and control non-targeting cells.

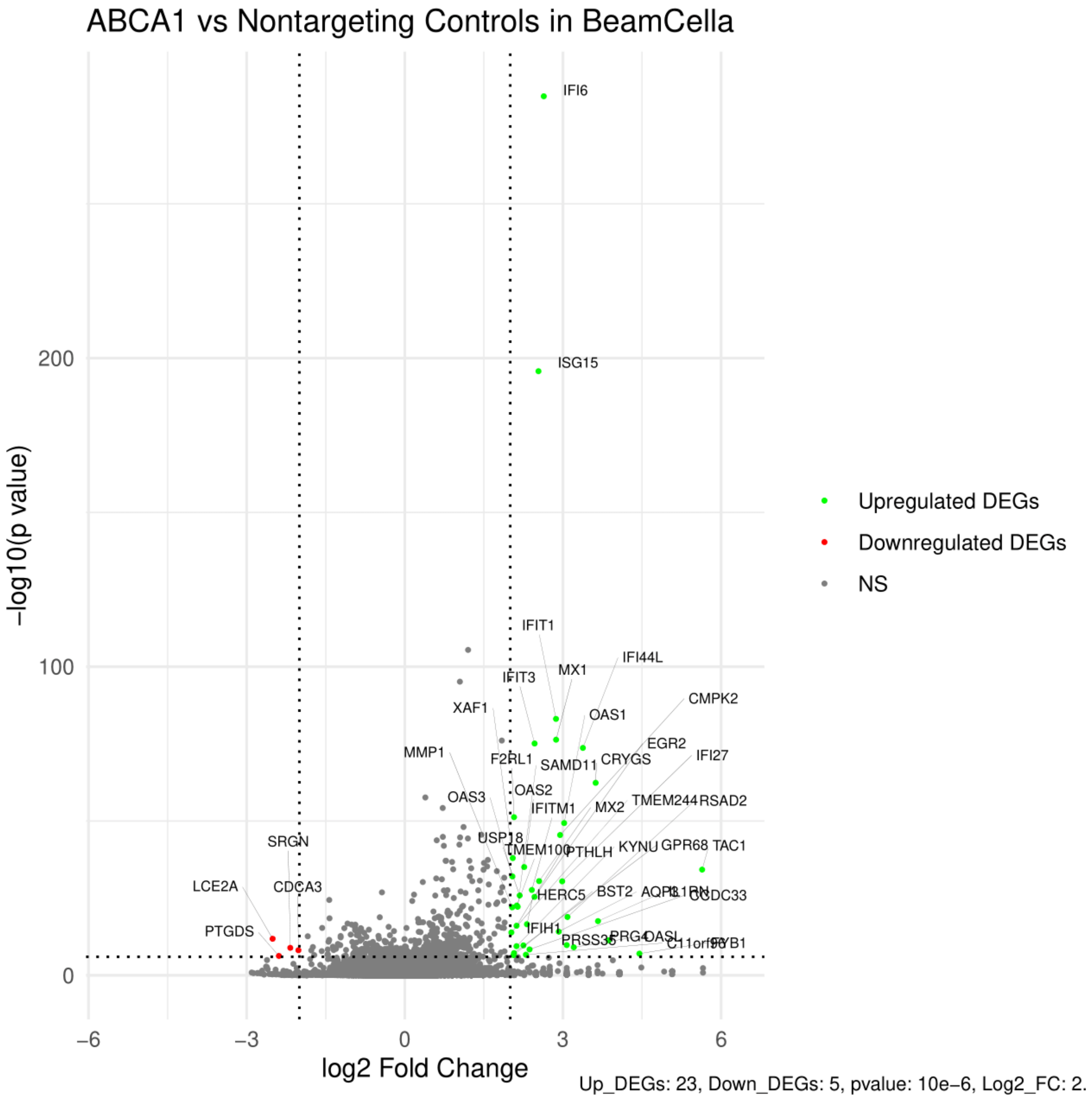

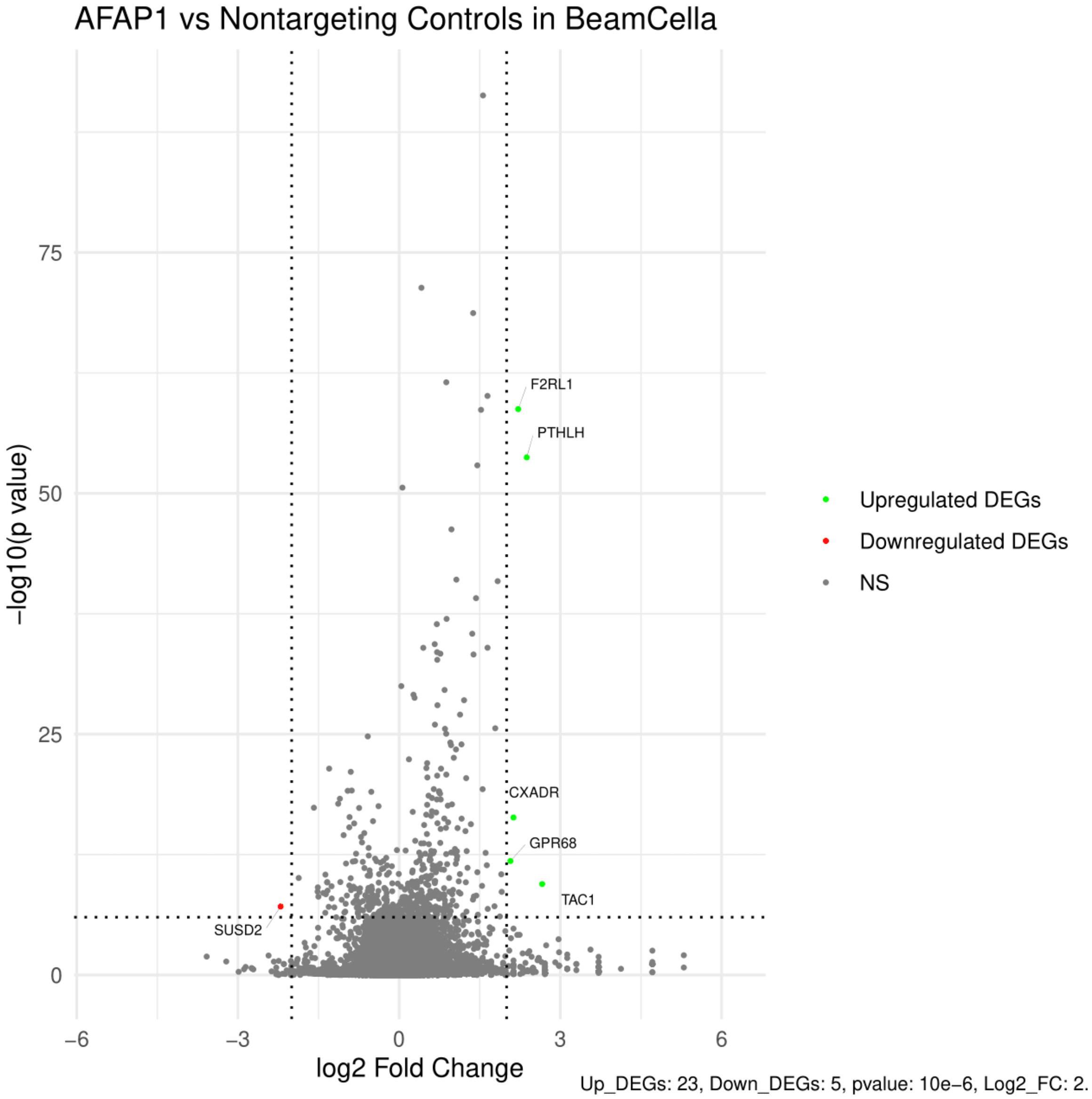

AFAP1 vs Nontargeting Controls in BeamCella
–log10(p value)
75
50
25
0
–6
–3
0
3
6
log2 Fold Change
F2RL1
PTHLH
CXADR
GPR68
TAC1
SUSD2
Upregulated DEGs
Downregulated DEGs
NS
Up_DEGs: 23, Down_DEGs: 5, pvalue: 10e–6, Log2_FC: 2.

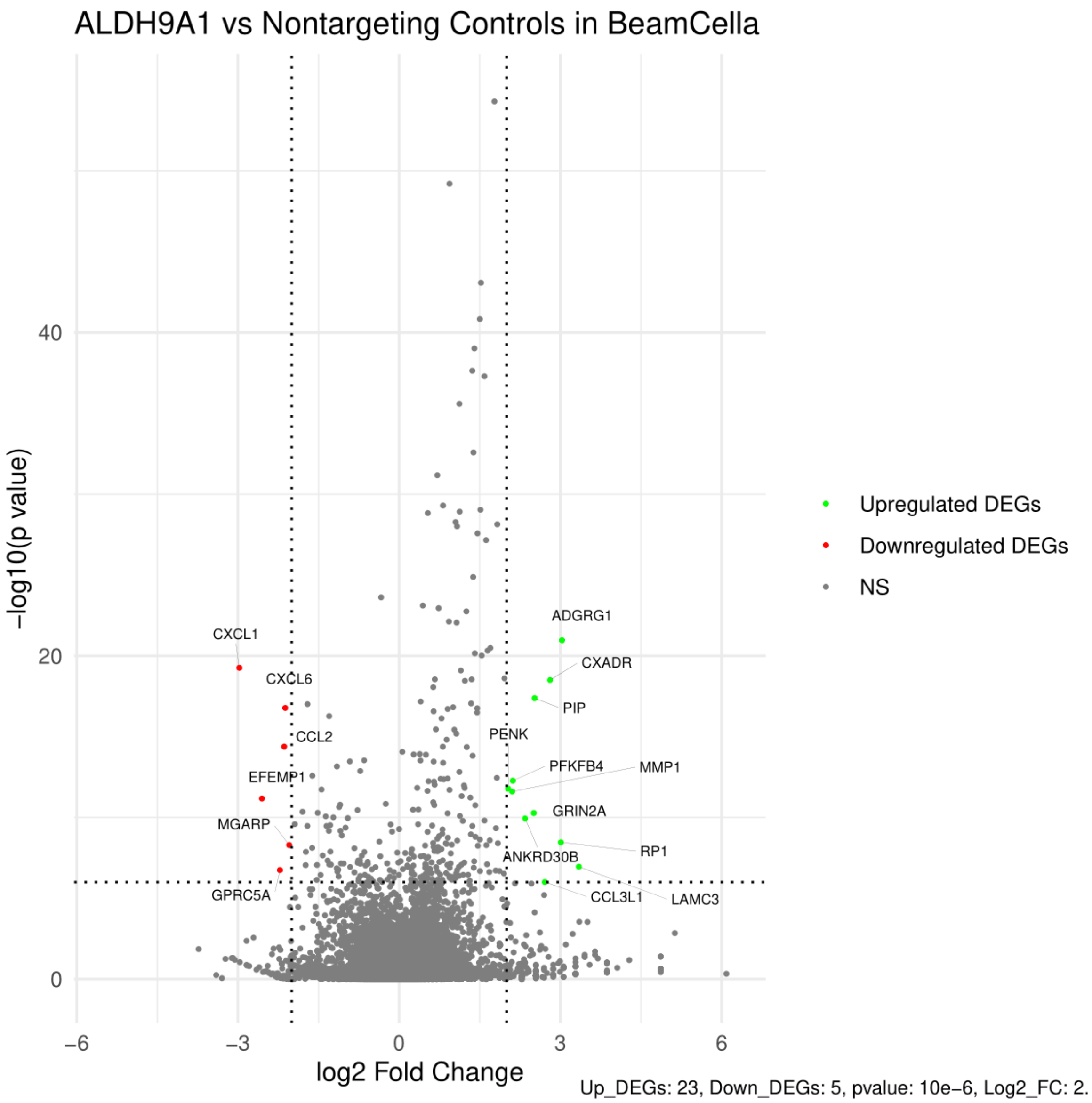

ALDH9A1 vs Nontargeting Controls in BeamCella
-log10(p value)
log2 Fold Change
40
20
0
-6
-3
0
3
6
CXCL1
CXCL6
CCL2
EFEMP1
MGARP
GPRC5A
ADGRG1
CXADR
PIP
PENK
PFKFB4
MMP1
GRIN2A
RP1
ANKRD30B
CCL3L1
LAMC3
Upregulated DEGs
Downregulated DEGs
NS
Up_DEGs: 23, Down_DEGs: 5, pvalue: 10e-6, Log2_FC: 2.

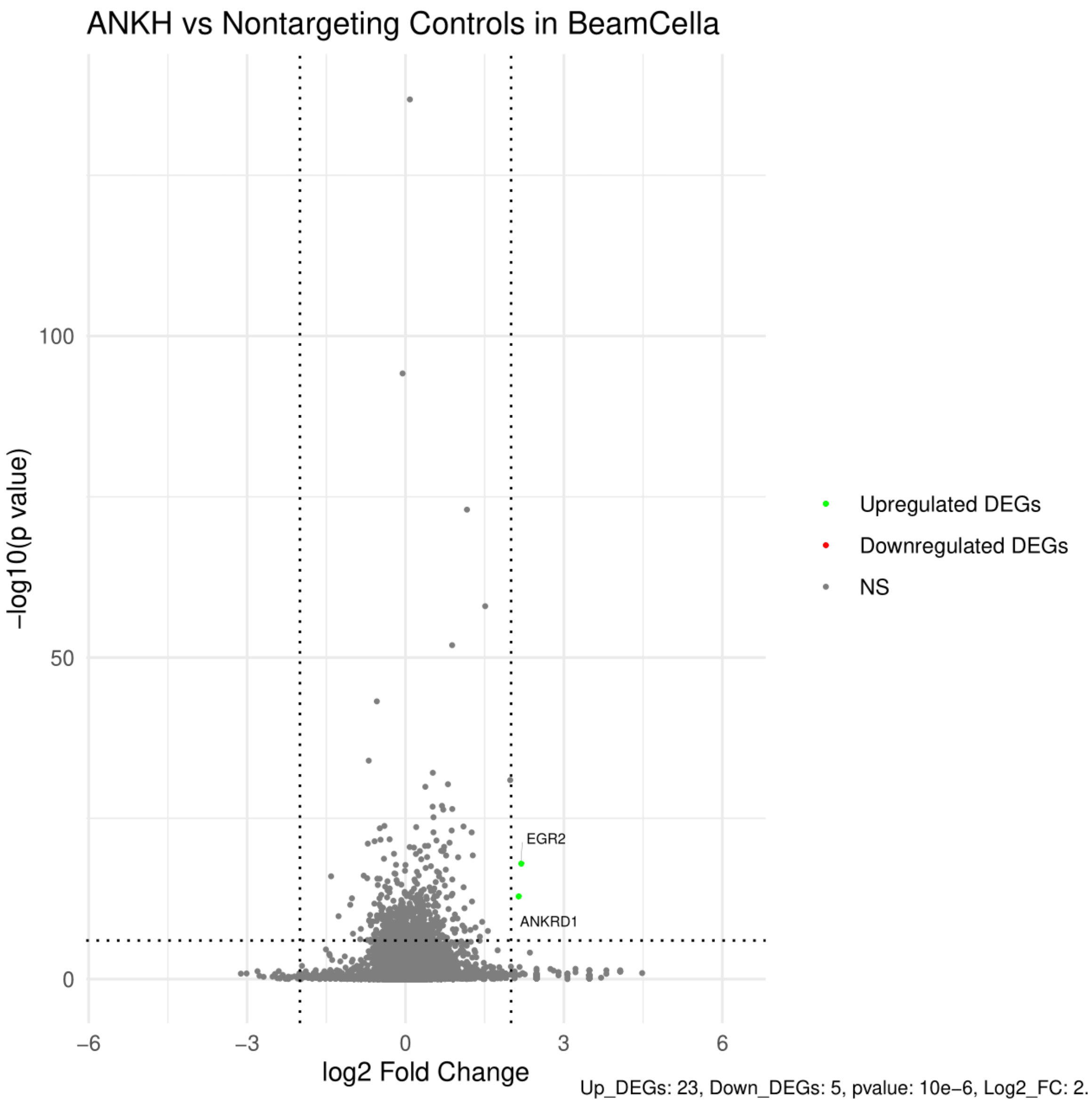
ANKH vs Nontargeting Controls in BeamCella
−log10(p value)
100
50
0
EGR2
ANKRD1
−6
−3
0
3
6
log2 Fold Change
Upregulated DEGs
Downregulated DEGs
NS
Up_DEGs: 23, Down_DEGs: 5, pvalue: 10e−6, Log2_FC: 2.

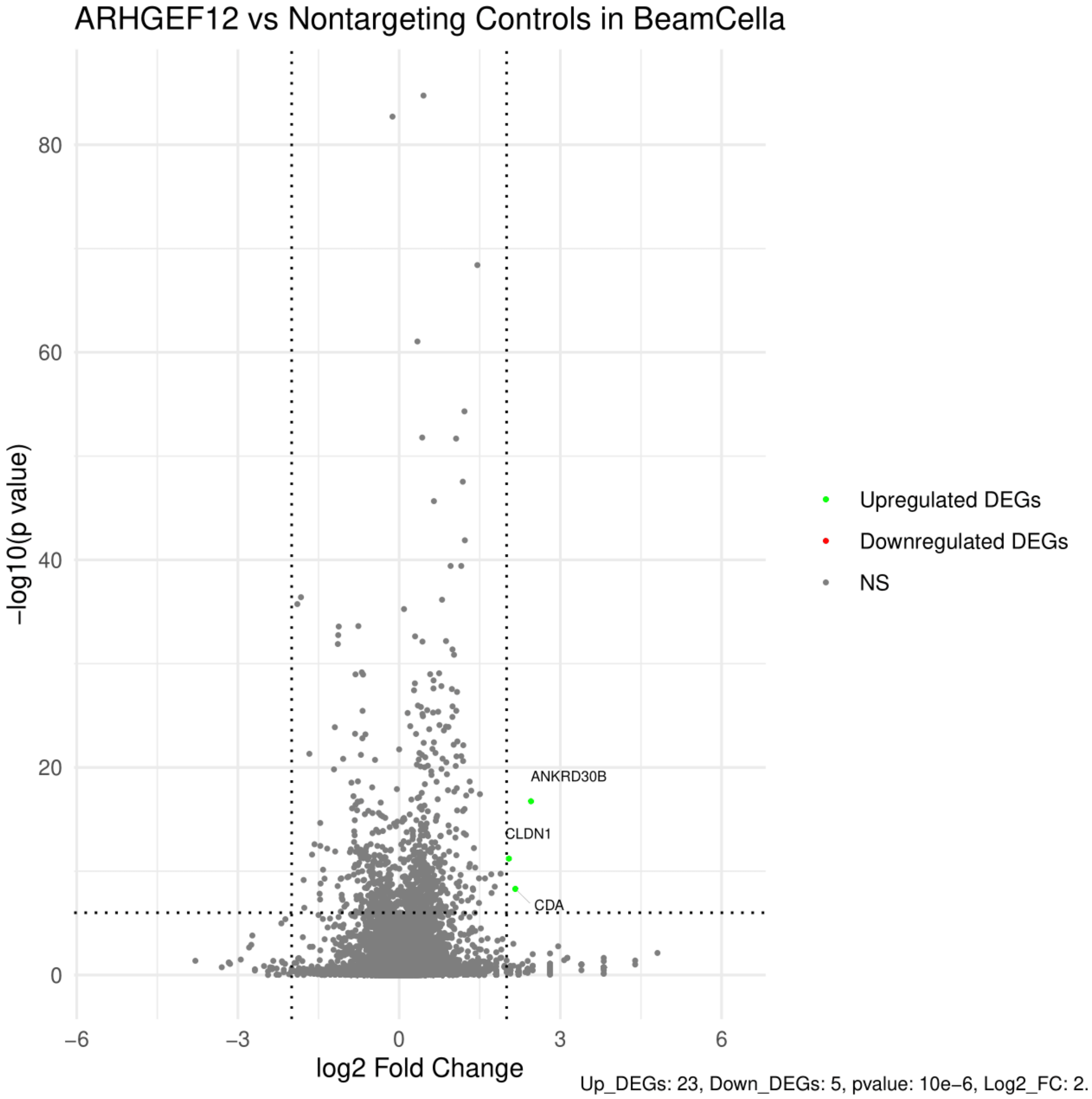

ARHGEF12 vs Nontargeting Controls in BeamCella
−log10(p value)
log2 Fold Change
ANKRD30B
CLDN1
CDA
Upregulated DEGs
Downregulated DEGs
NS
Up_DEGs: 23, Down_DEGs: 5, pvalue: 10e−6, Log2_FC: 2.

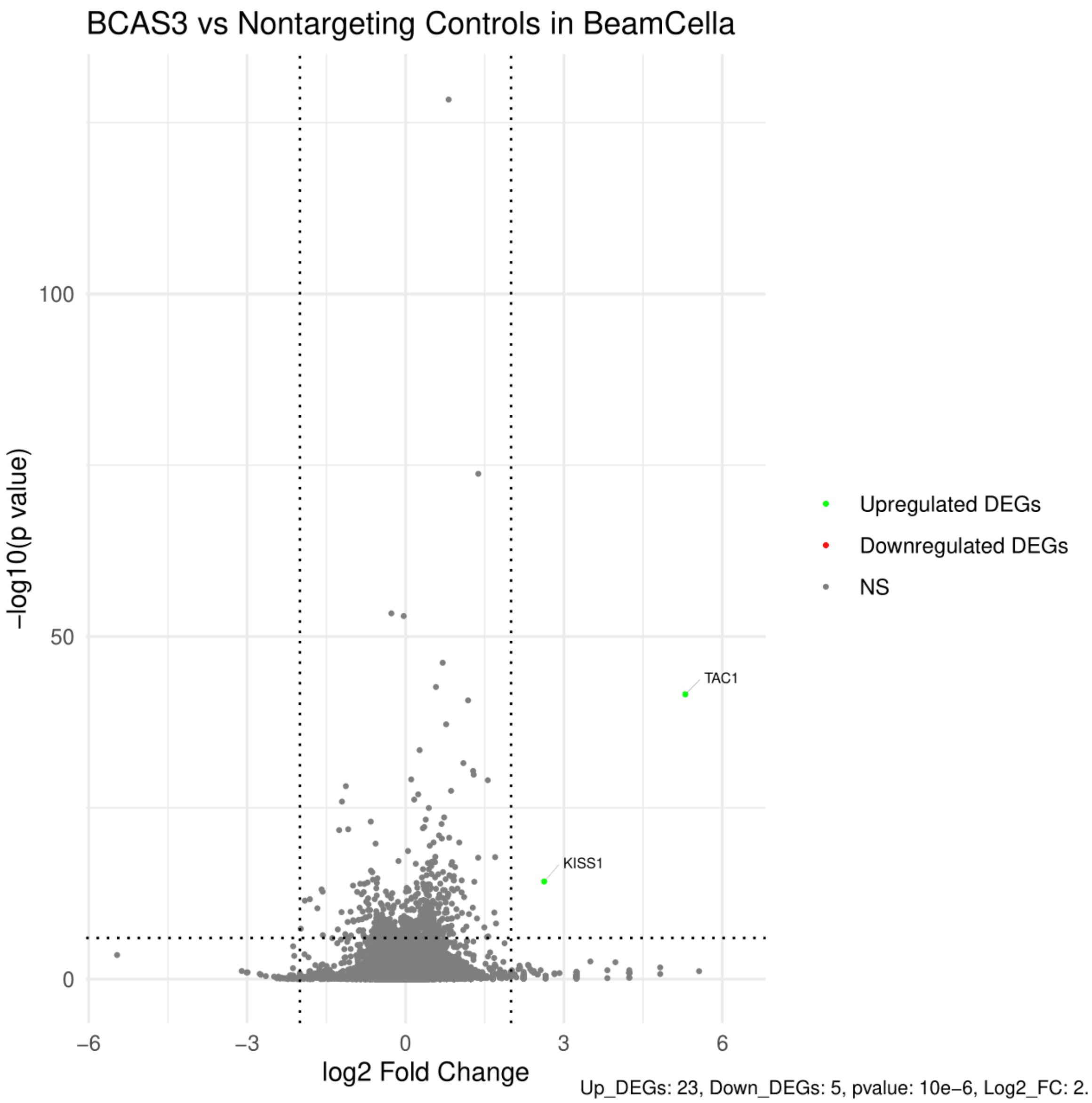
BCAS3 vs Nontargeting Controls in BeamCella
−log10(p value)
100
50
0
−6
−3
0
3
6
log2 Fold Change
TAC1
KISS1
Upregulated DEGs
Downregulated DEGs
NS
Up_DEGs: 23, Down_DEGs: 5, pvalue: 10e−6, Log2_FC: 2.

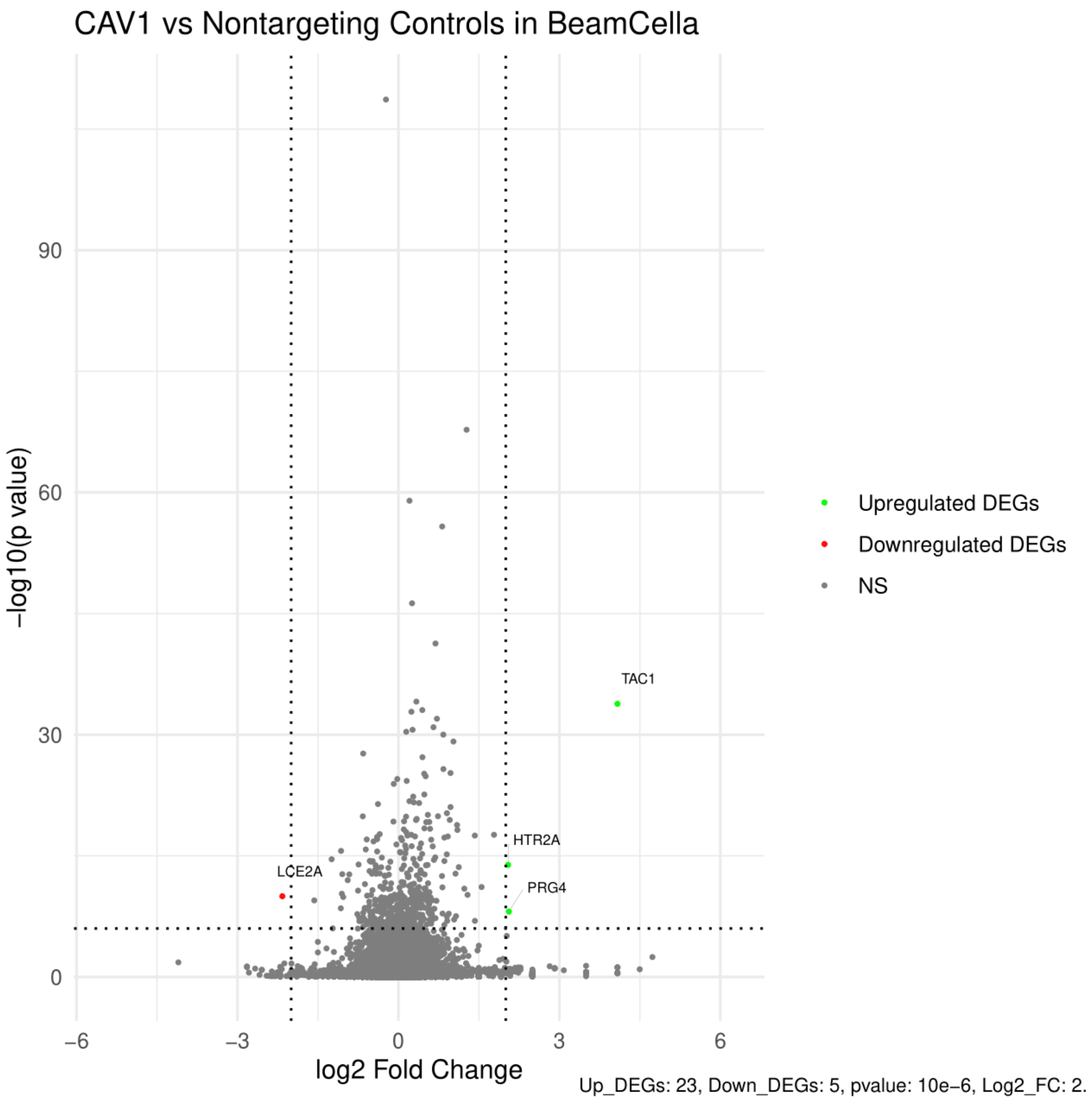

CAV1 vs Nontargeting Controls in BeamCella
−log10(p value)
90
60
30
0
−6
−3
0
3
6
log2 Fold Change
TAC1
HTR2A
LCE2A
PRG4
Upregulated DEGs
Downregulated DEGs
NS
Up_DEGs: 23, Down_DEGs: 5, pvalue: 10e−6, Log2_FC: 2.

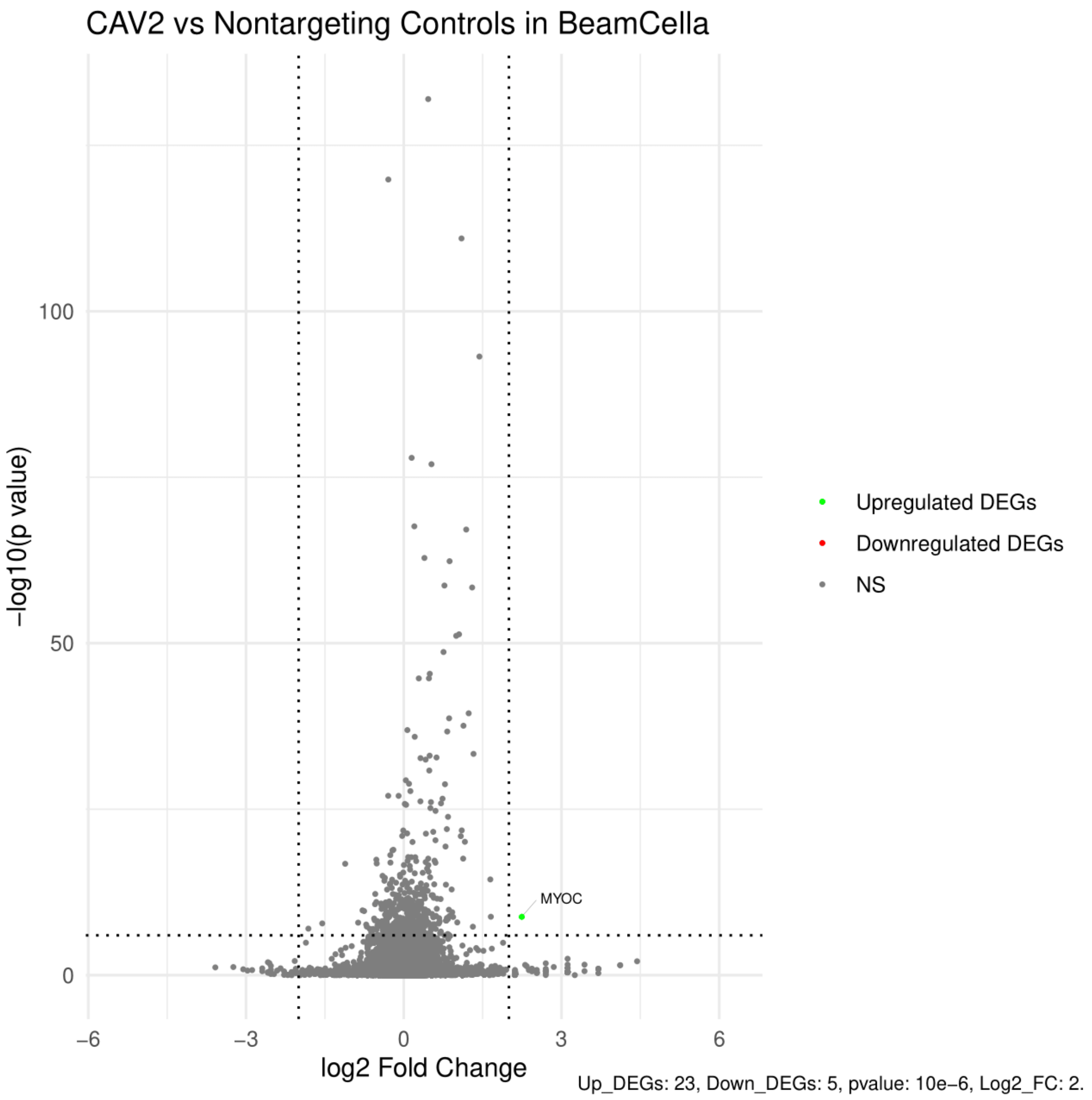

CAV2 vs Nontargeting Controls in BeamCella
−log10(p value)
100
50
0
MYOC
−6
−3
0
3
6
log2 Fold Change
Upregulated DEGs
Downregulated DEGs
NS
Up_DEGs: 23, Down_DEGs: 5, pvalue: 10e−6, Log2_FC: 2.

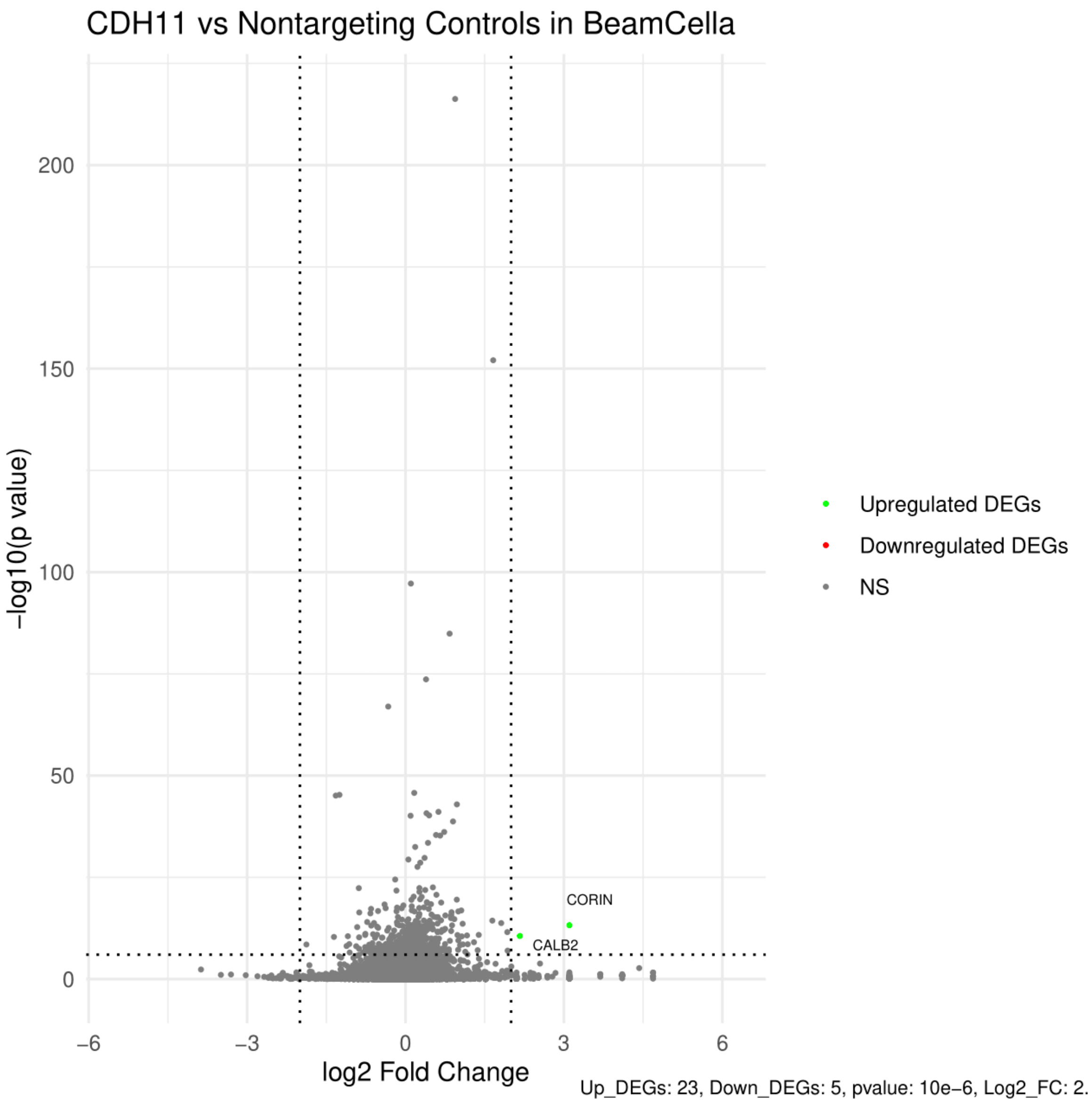
CDH11 vs Nontargeting Controls in BeamCella
−log10(p value)
200
150
100
50
0
−6
−3
0
3
6
log2 Fold Change
CORIN
CALB2
Upregulated DEGs
Downregulated DEGs
NS
Up_DEGs: 23, Down_DEGs: 5, pvalue: 10e−6, Log2_FC: 2.

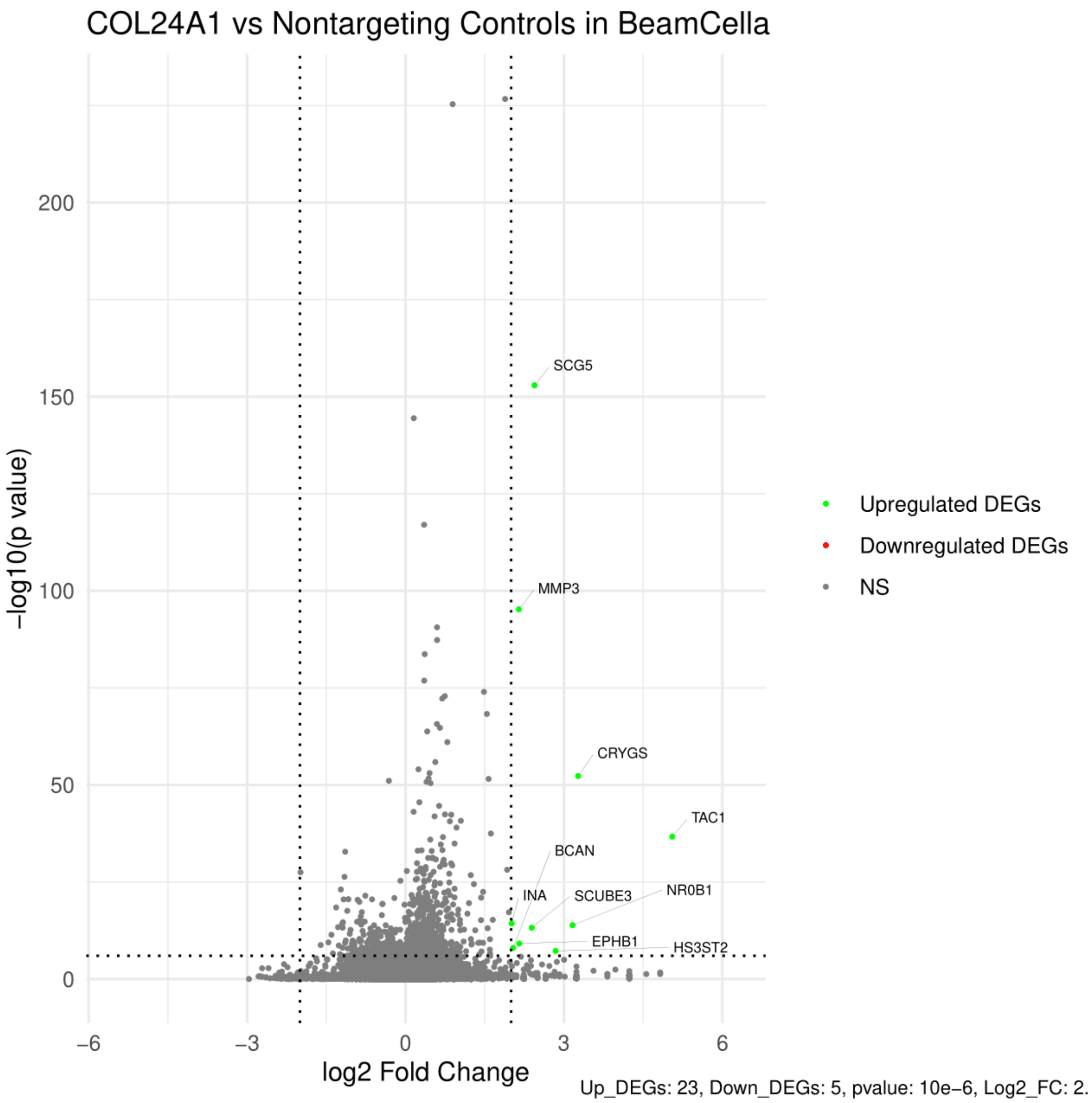
COL24A1 vs Nontargeting Controls in BeamCella
−log10(p value)
log2 Fold Change
SCG5
MMP3
CRYGS
TAC1
BCAN
INA
SCUBE3
NR0B1
EPHB1
HS3ST2
Upregulated DEGs
Downregulated DEGs
NS
Up_DEGs: 23, Down_DEGs: 5, pvalue: 10e−6, Log2_FC: 2.

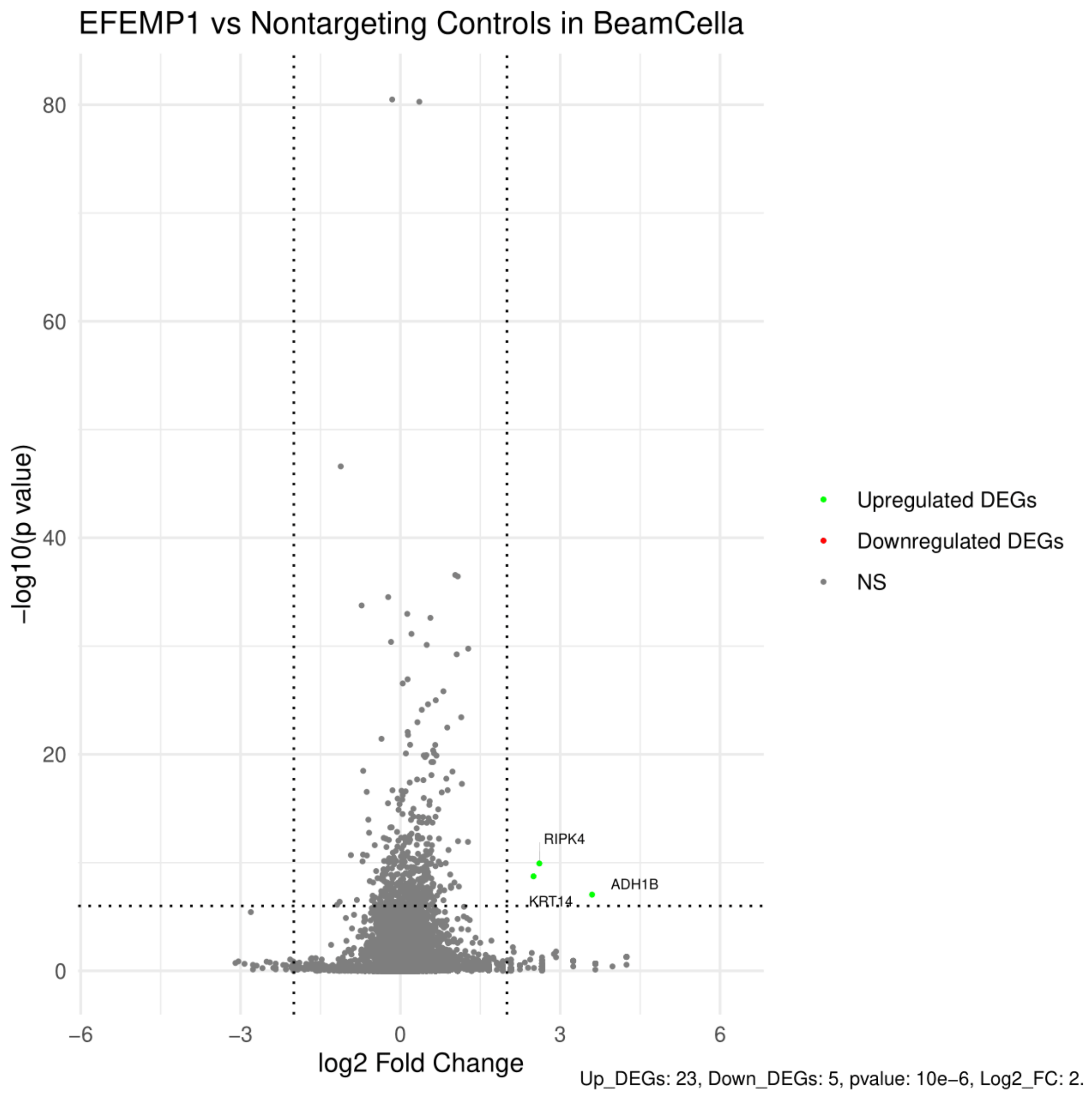
EFEMP1 vs Nontargeting Controls in BeamCella
−log10(p value)
log2 Fold Change
RIPK4
ADH1B
KRT14
Upregulated DEGs
Downregulated DEGs
NS
Up_DEGs: 23, Down_DEGs: 5, pvalue: 10e−6, Log2_FC: 2.

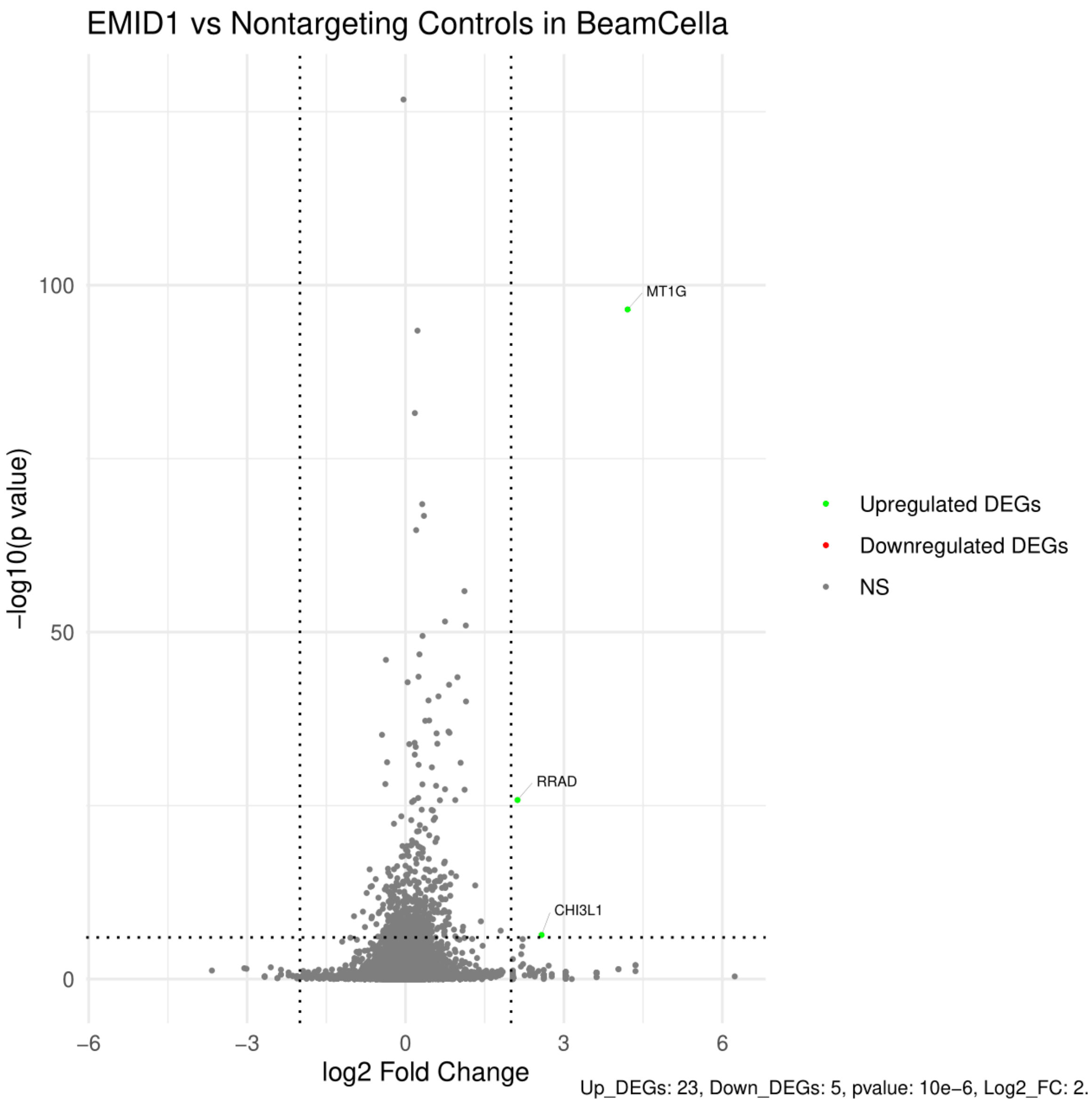
EMID1 vs Nontargeting Controls in BeamCella
−log10(p value)
100
50
0
MT1G
RRAD
CHI3L1
−6
−3
0
3
6
log2 Fold Change
Upregulated DEGs
Downregulated DEGs
NS
Up_DEGs: 23, Down_DEGs: 5, pvalue: 10e−6, Log2_FC: 2.

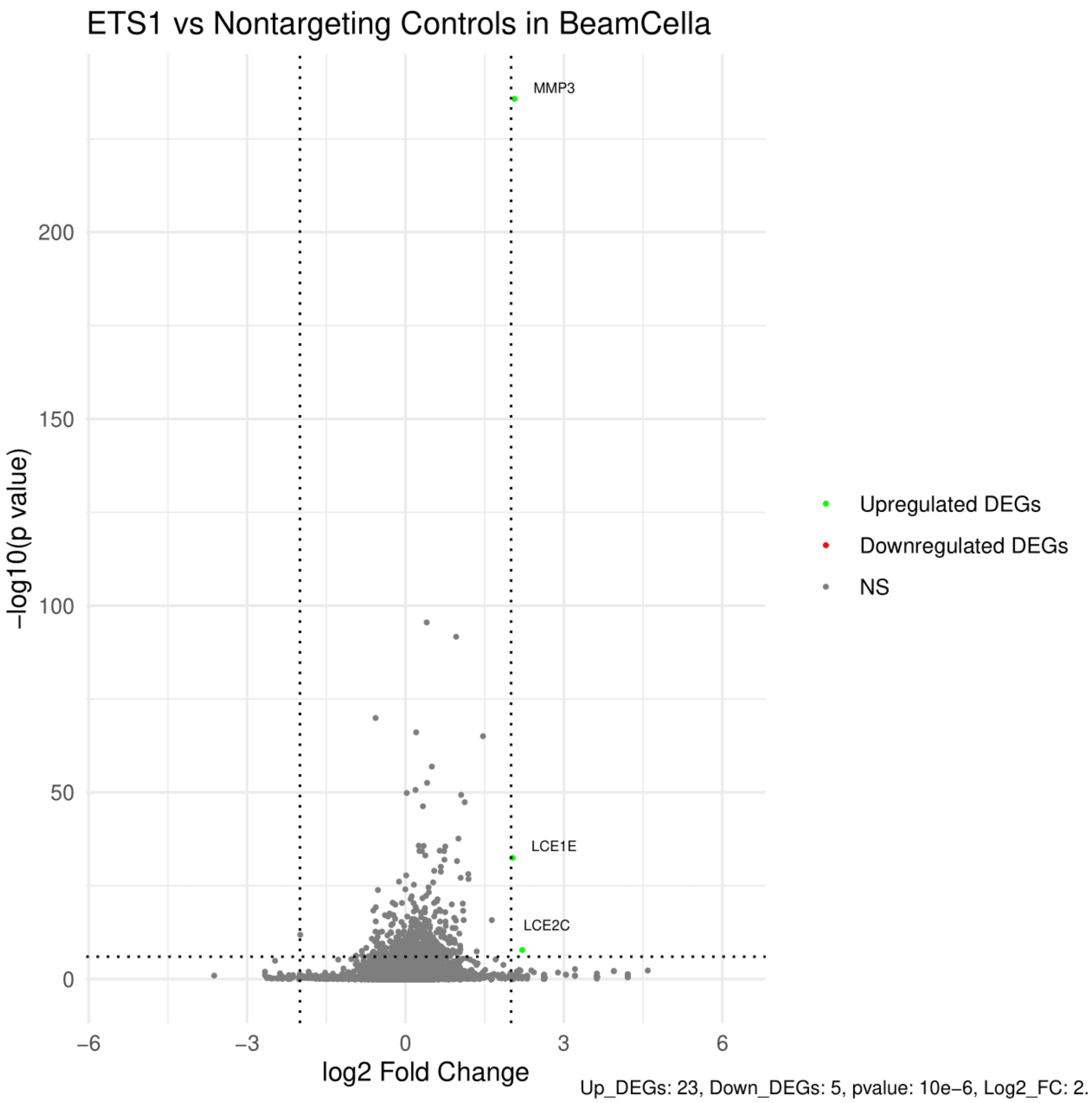
ETS1 vs Nontargeting Controls in BeamCella
MMP3
LCE1E
LCE2C
200
150
100
50
0
−log10(p value)
−6
−3
0
3
6
log2 Fold Change
Upregulated DEGs
Downregulated DEGs
NS
Up_DEGs: 23, Down_DEGs: 5, pvalue: 10e−6, Log2_FC: 2.

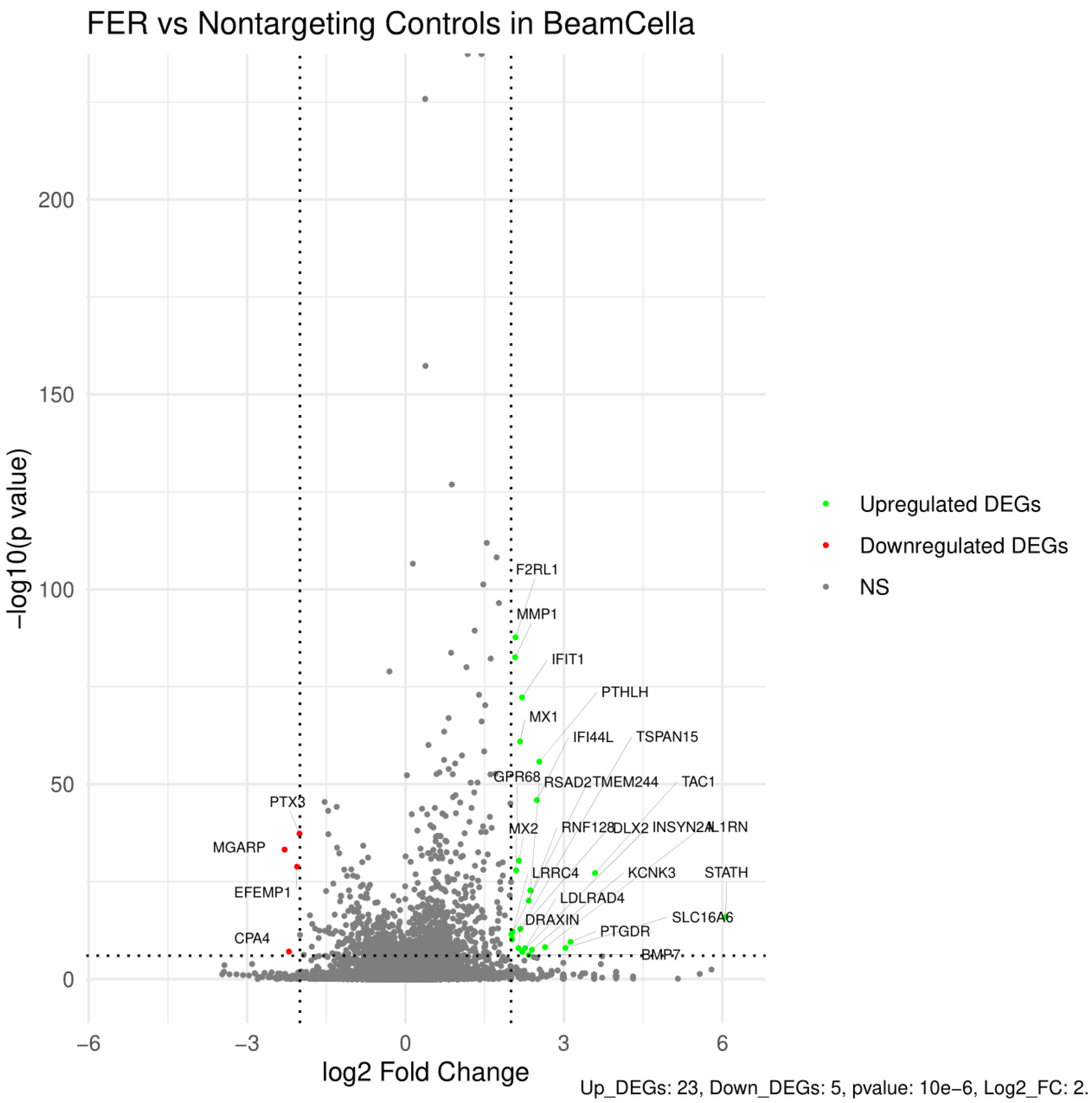
FER vs Nontargeting Controls in BeamCella
-log10(p value)
log2 Fold Change
F2RL1
MMP1
IFIT1
PTHLH
MX1
IFI44L
TSPAN15
GPR68
RSAD2
TMEM244
TAC1
PTX3
MGARP
EFEMP1
CPA4
MX2
RNF128
DLX2
INSYN2A
IL1RN
LRRC4
KCNK3
STATH
LDLRAD4
DRAXIN
SLC16A6
PTGDR
BMP7
Upregulated DEGs
Downregulated DEGs
NS
Up_DEGs: 23, Down_DEGs: 5, pvalue: 10e−6, Log2_FC: 2.

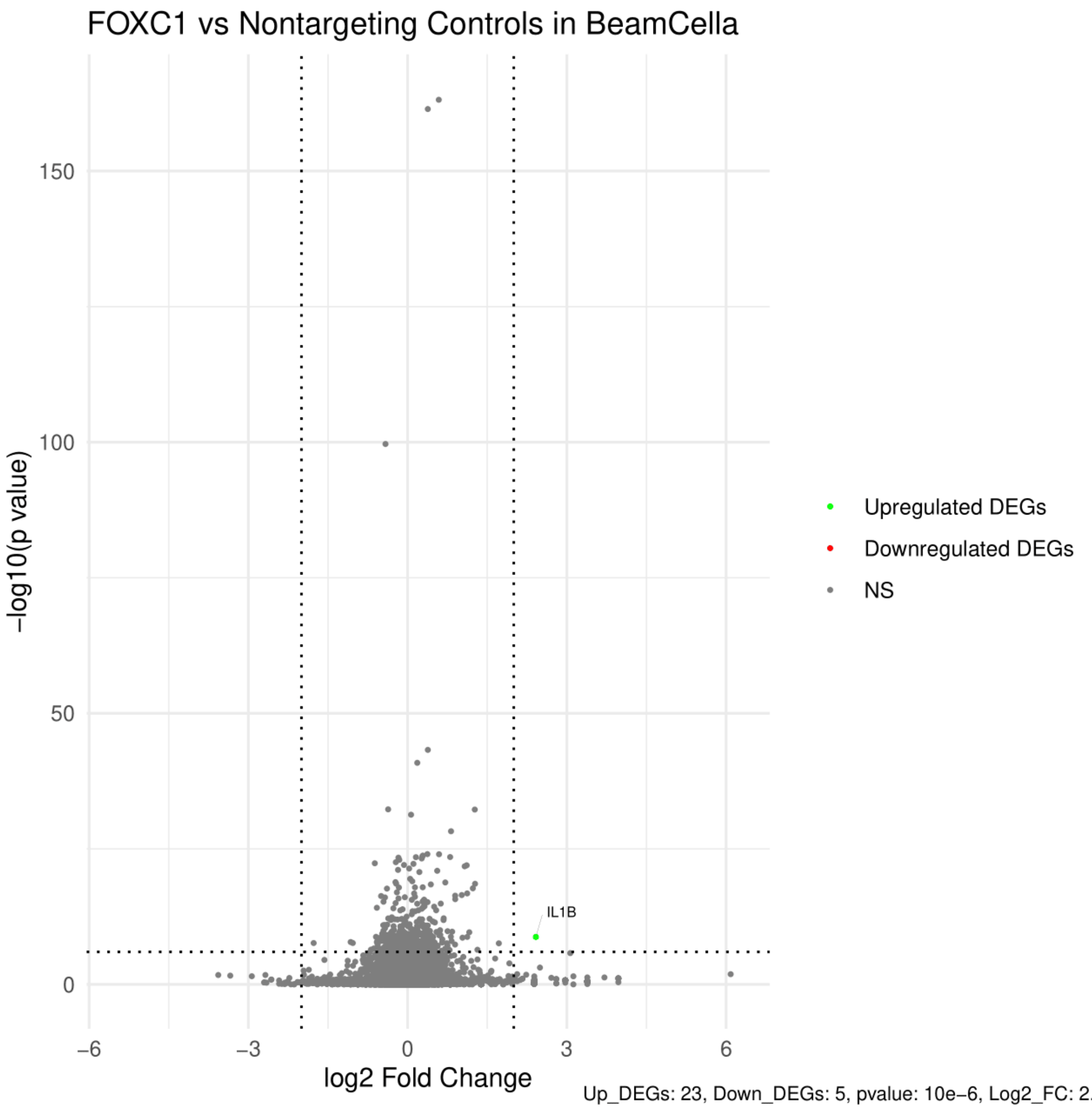

Up_DEGs: 23, Down_DEGs: 5, pvalue: 10e−6, Log2_FC: 2.

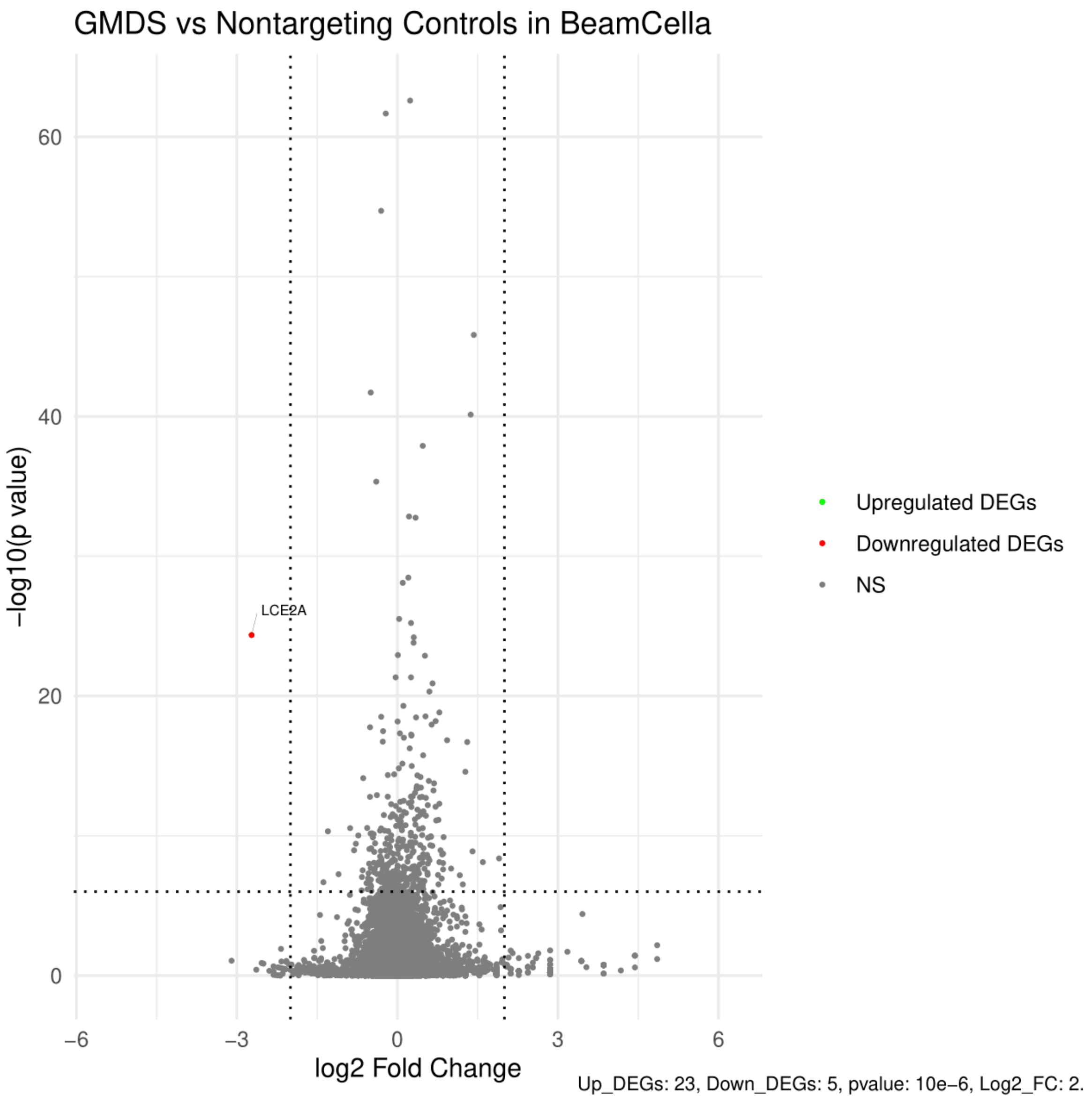
GMDS vs Nontargeting Controls in BeamCella
−log10(p value)
60
40
20
0
−6
−3
0
3
6
log2 Fold Change
LCE2A
Upregulated DEGs
Downregulated DEGs
NS
Up_DEGs: 23, Down_DEGs: 5, pvalue: 10e−6, Log2_FC: 2.

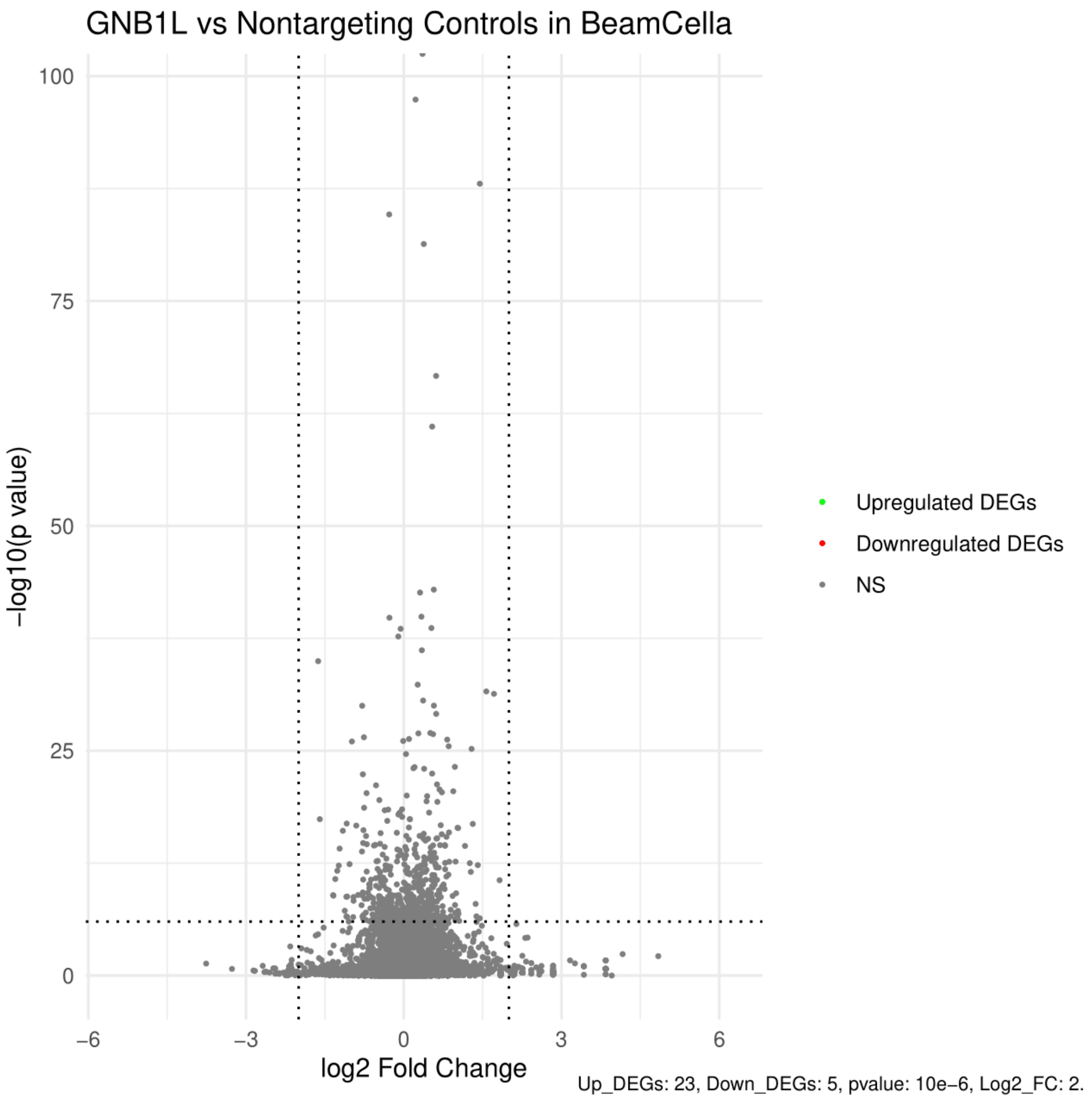
GNB1L vs Nontargeting Controls in BeamCella
100
75
50
25
0
−log10(p value)
−6
−3
0
3
6
log2 Fold Change
Upregulated DEGs
Downregulated DEGs
NS
Up_DEGs: 23, Down_DEGs: 5, pvalue: 10e−6, Log2_FC: 2.

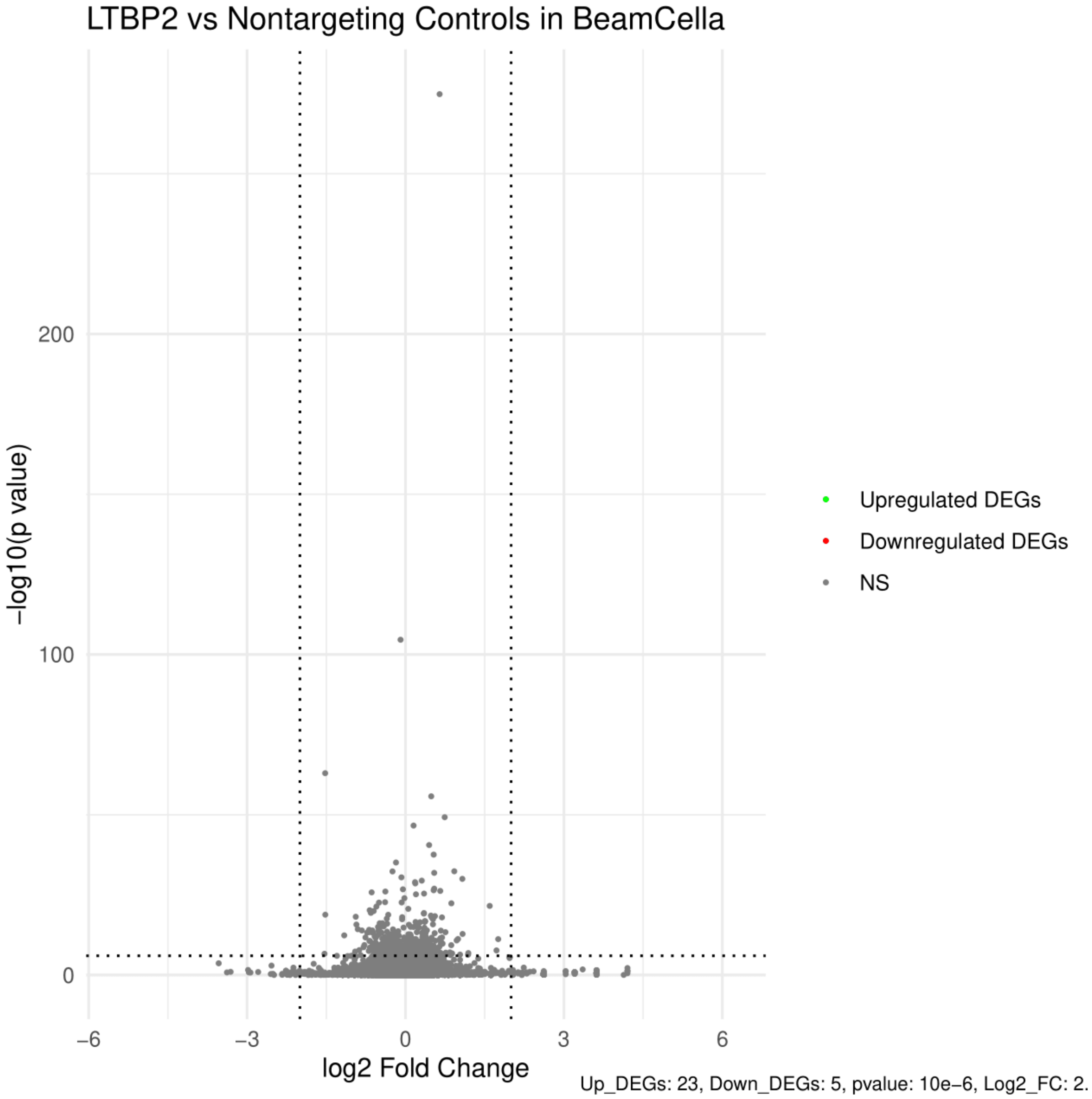
LTBP2 vs Nontargeting Controls in BeamCella
200
100
0
−log10(p value)
−6
−3
0
3
6
log2 Fold Change
Upregulated DEGs
Downregulated DEGs
NS
Up_DEGs: 23, Down_DEGs: 5, pvalue: 10e−6, Log2_FC: 2.

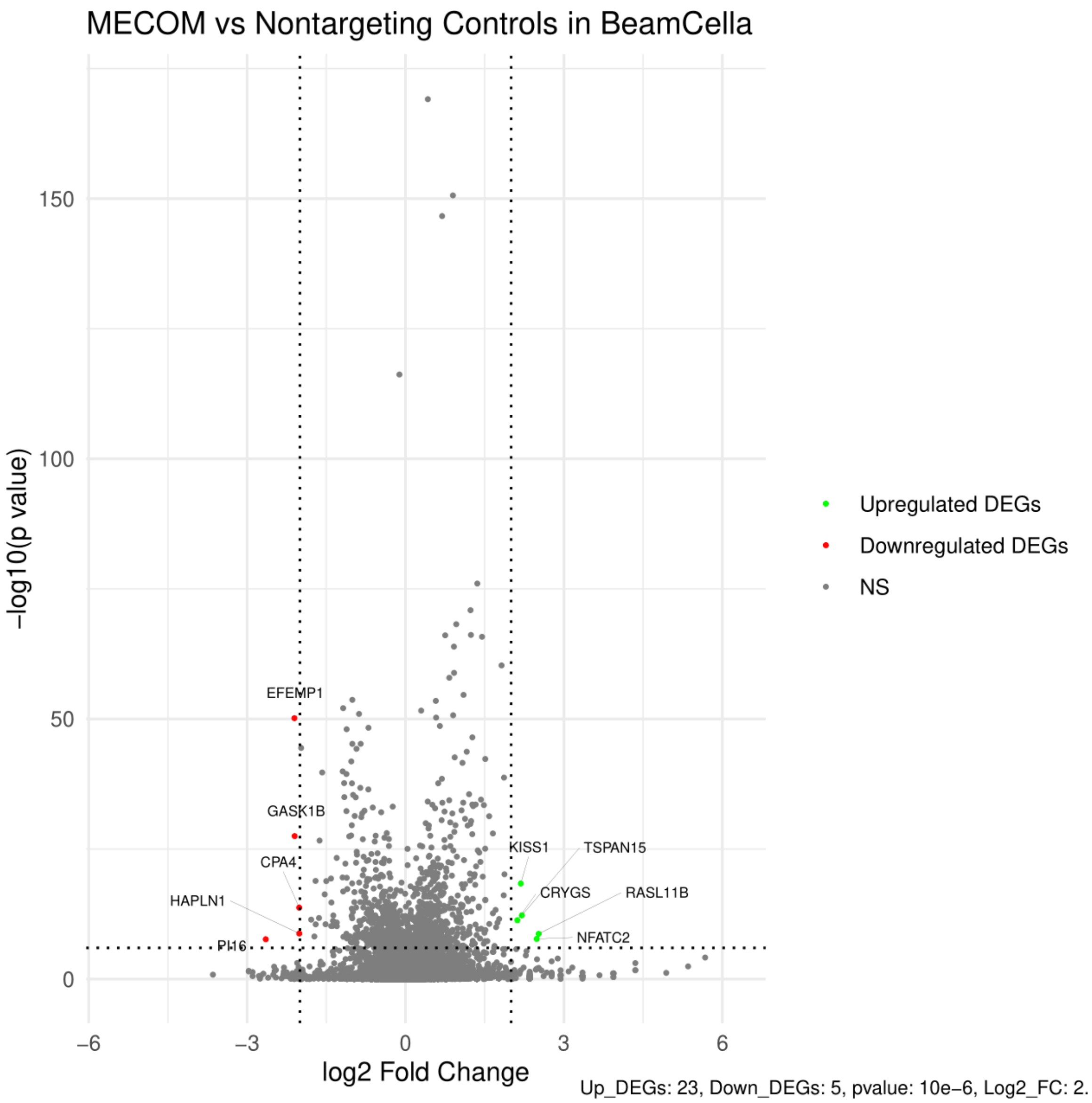

MECOM vs Nontargeting Controls in BeamCella
-log10(p value)
150
100
50
0
EFEMP1
GASK1B
CPA4
HAPLN1
PI16
KISS1
TSPAN15
CRYGS
RASL11B
NFATC2
-6
-3
0
3
6
log2 Fold Change
Upregulated DEGs
Downregulated DEGs
NS
Up_DEGs: 23, Down_DEGs: 5, pvalue: 10e-6, Log2_FC: 2.

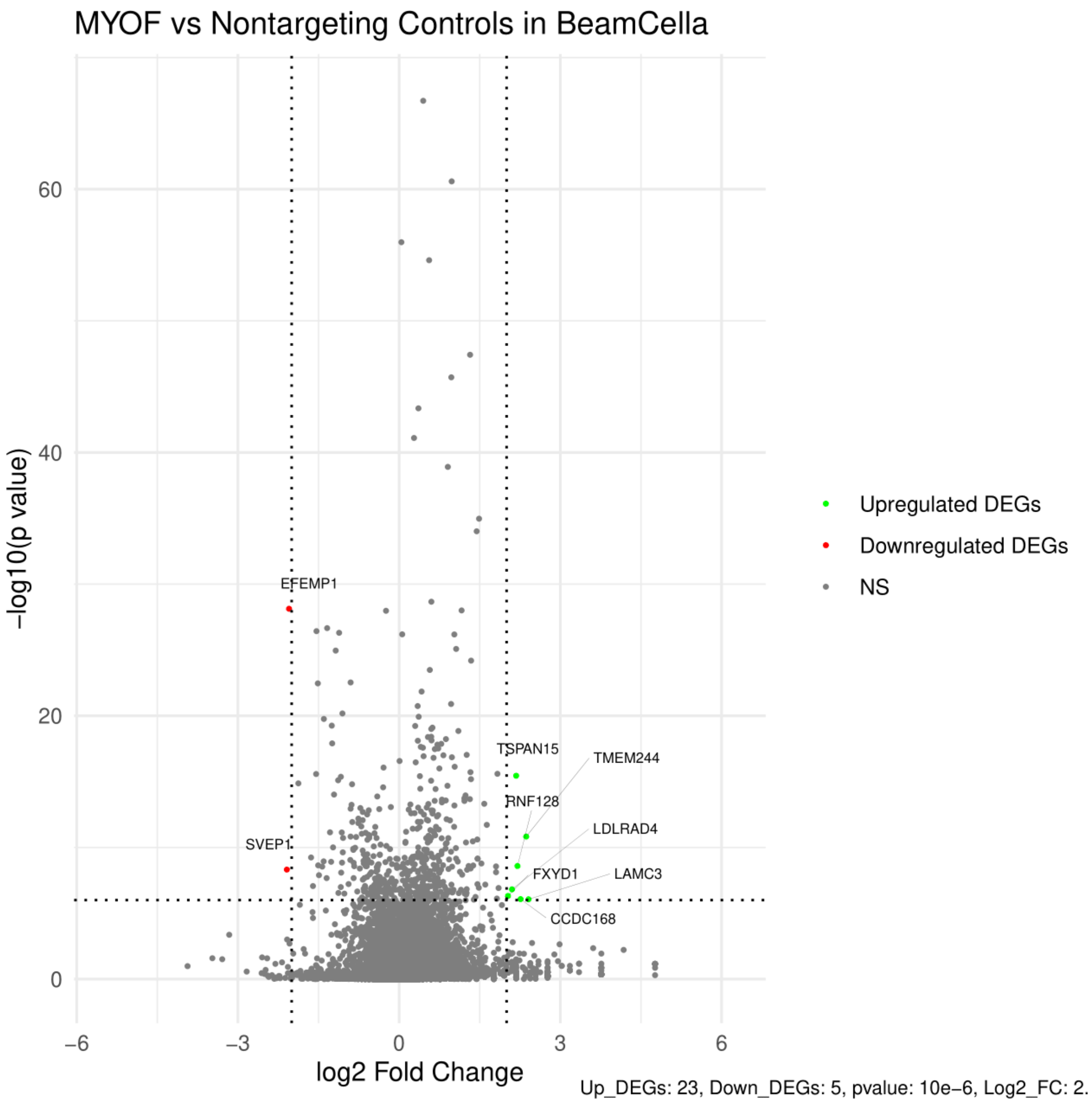
MYOF vs Nontargeting Controls in BeamCella
−log10(p value)
60
40
20
0
−6
−3
0
3
6
log2 Fold Change
EFEMP1
SVEP1
TSPAN15
TMEM244
RNF128
LDLRAD4
FXYD1
LAMC3
CCDC168
Upregulated DEGs
Downregulated DEGs
NS
Up_DEGs: 23, Down_DEGs: 5, pvalue: 10e−6, Log2_FC: 2.

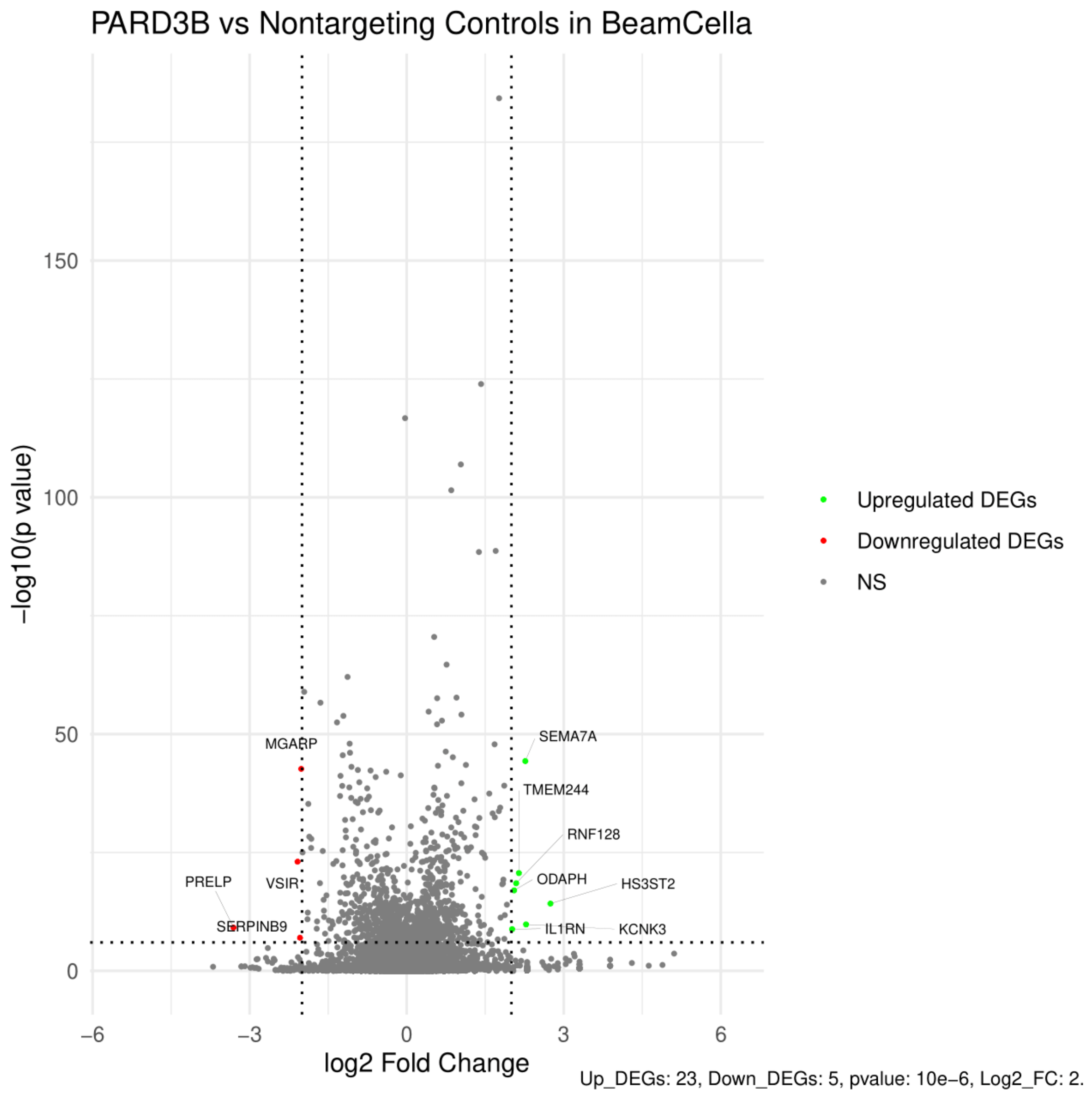

PARD3B vs Nontargeting Controls in BeamCella
−log10(p value)
log2 Fold Change
150
100
50
0
−6
−3
0
3
6
MGARP
SEMA7A
TMEM244
RNF128
ODAPH
HS3ST2
PRELP
VSIR
SERPINB9
IL1RN
KCNK3
Upregulated DEGs
Downregulated DEGs
NS
Up_DEGs: 23, Down_DEGs: 5, pvalue: 10e−6, Log2_FC: 2.

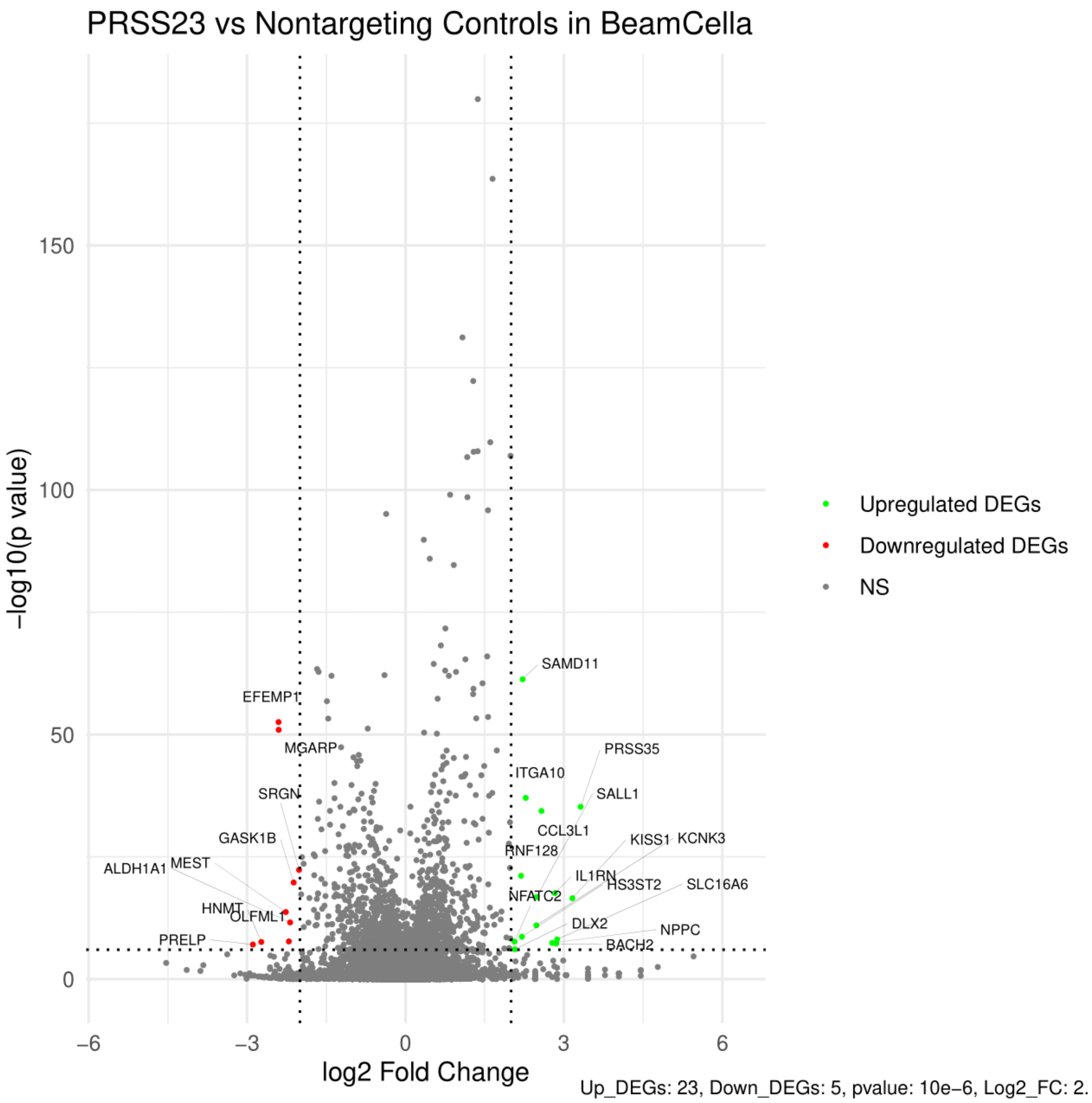

PRSS23 vs Nontargeting Controls in BeamCella
−log10(p value)
log2 Fold Change
Upregulated DEGs
Downregulated DEGs
NS
SAMD11
EFEMP1
MGARP
PRSS35
ITGA10
SRGN
SALL1
CCL3L1
GASK1B
KISS1
KCNK3
RNF128
ALDH1A1
MEST
IL1RN
HS3ST2
SLC16A6
NFATC2
HNMT
OLFML1
DLX2
NPPC
PRELP
BACH2
Up_DEGs: 23, Down_DEGs: 5, pvalue: 10e−6, Log2_FC: 2.

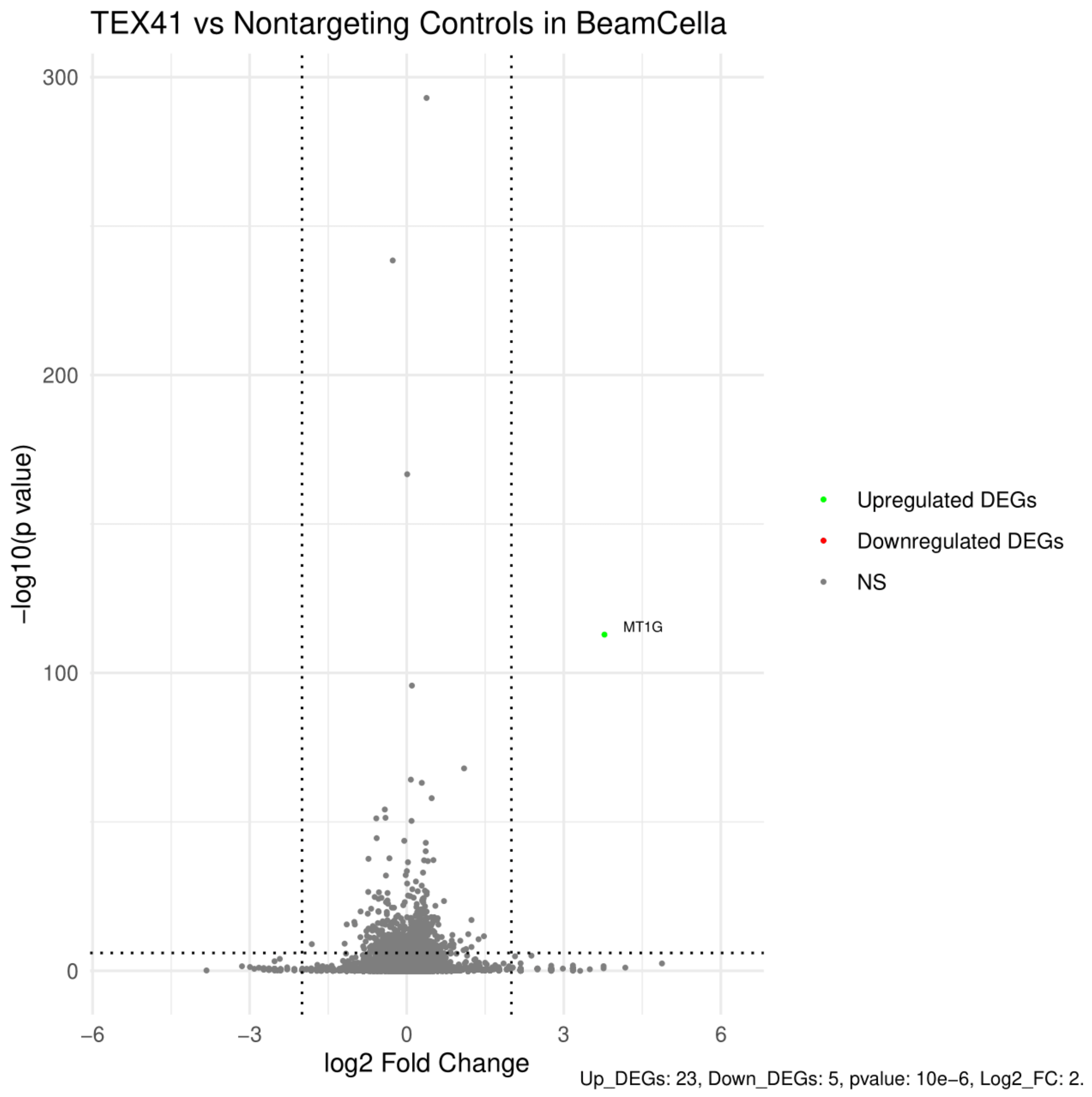

TEX41 vs Nontargeting Controls in BeamCella
300
200
100
0
-log10(p value)
MT1G
-6
-3
0
3
6
log2 Fold Change
Upregulated DEGs
Downregulated DEGs
NS
Up_DEGs: 23, Down_DEGs: 5, pvalue: 10e-6, Log2_FC: 2.

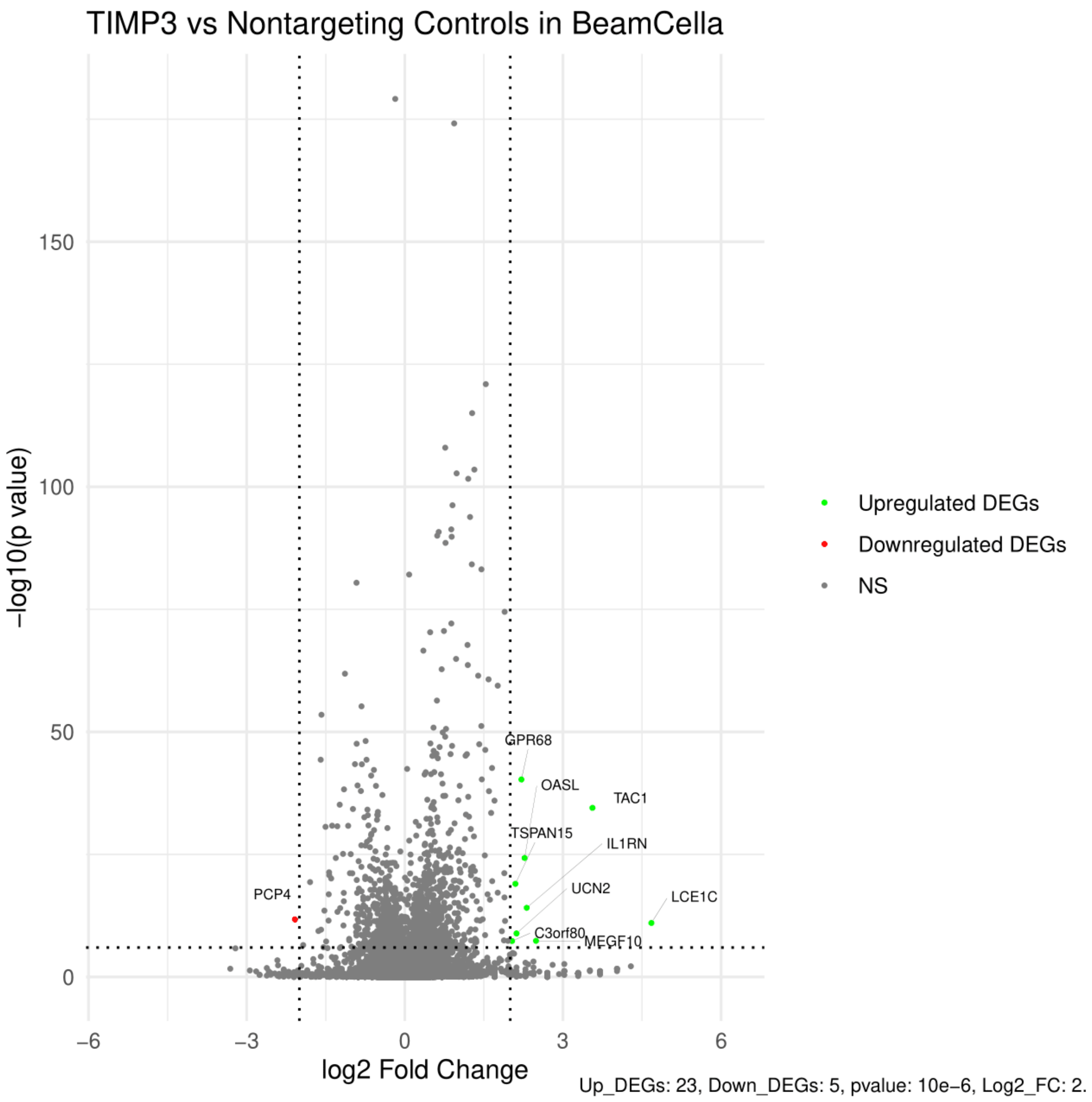
TIMP3 vs Nontargeting Controls in BeamCella
−log10(p value)
150
100
50
0
−6
−3
0
3
6
log2 Fold Change
GPR68
OASL
TAC1
TSPAN15
IL1RN
UCN2
LCE1C
PCP4
C3orf80
MEGF10
Upregulated DEGs
Downregulated DEGs
NS
Up_DEGs: 23, Down_DEGs: 5, pvalue: 10e−6, Log2_FC: 2.

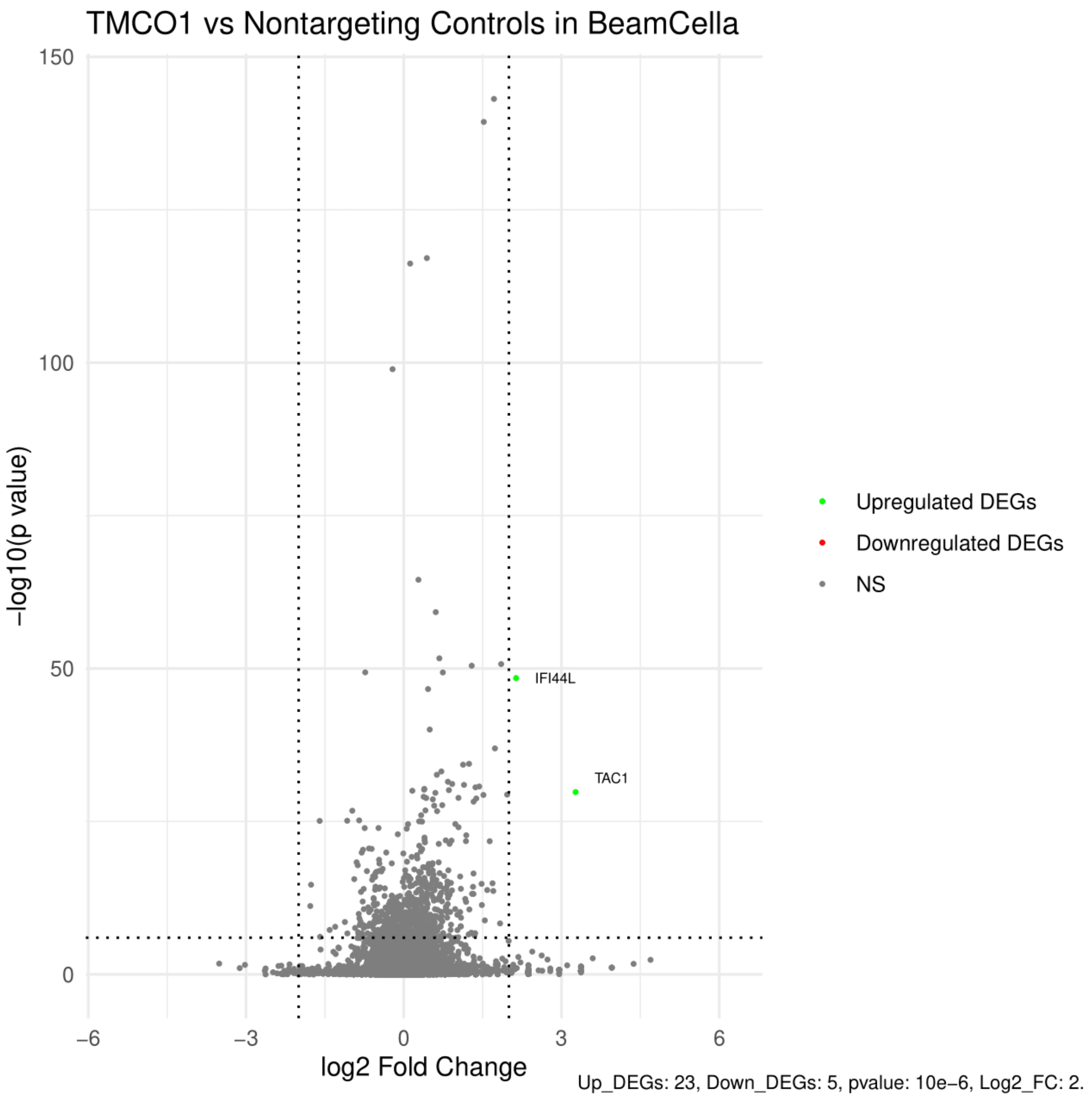
TMCO1 vs Nontargeting Controls in BeamCella
150
100
50
0
−log10(p value)
−6
−3
0
3
6
log2 Fold Change
IFI44L
TAC1
Upregulated DEGs
Downregulated DEGs
NS
Up_DEGs: 23, Down_DEGs: 5, pvalue: 10e−6, Log2_FC: 2.

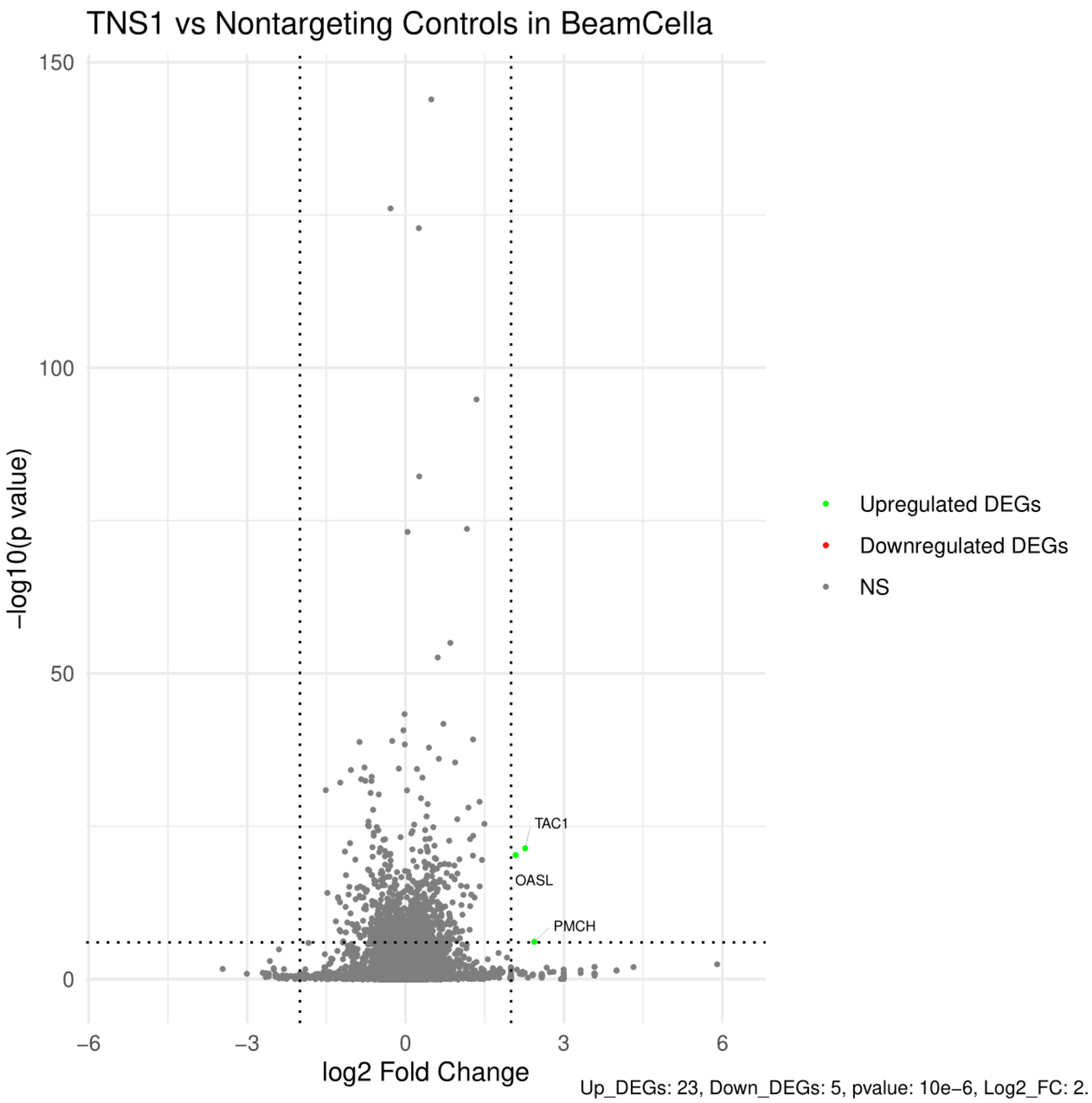
TNS1 vs Nontargeting Controls in BeamCella
150
100
50
0
−log10(p value)
TAC1
OASL
PMCH
−6
−3
0
3
6
log2 Fold Change
Upregulated DEGs
Downregulated DEGs
NS
Up_DEGs: 23, Down_DEGs: 5, pvalue: 10e−6, Log2_FC: 2.

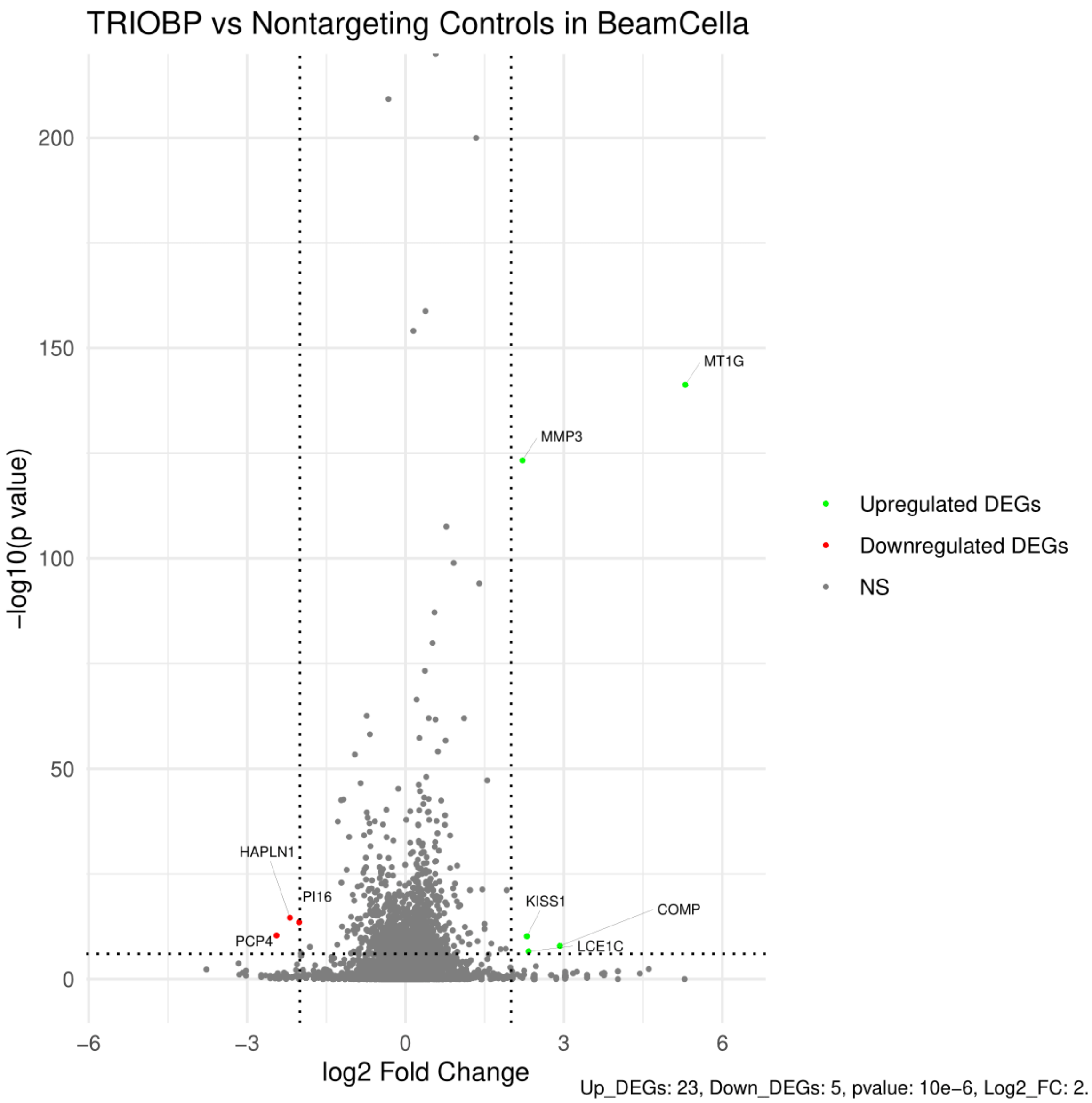
TRIOBP vs Nontargeting Controls in BeamCella
−log10(p value)
log2 Fold Change
200
150
100
50
0
−6
−3
0
3
6
MT1G
MMP3
HAPLN1
PI16
PCP4
KISS1
COMP
LCE1C
Upregulated DEGs
Downregulated DEGs
NS
Up_DEGs: 23, Down_DEGs: 5, pvalue: 10e−6, Log2_FC: 2.

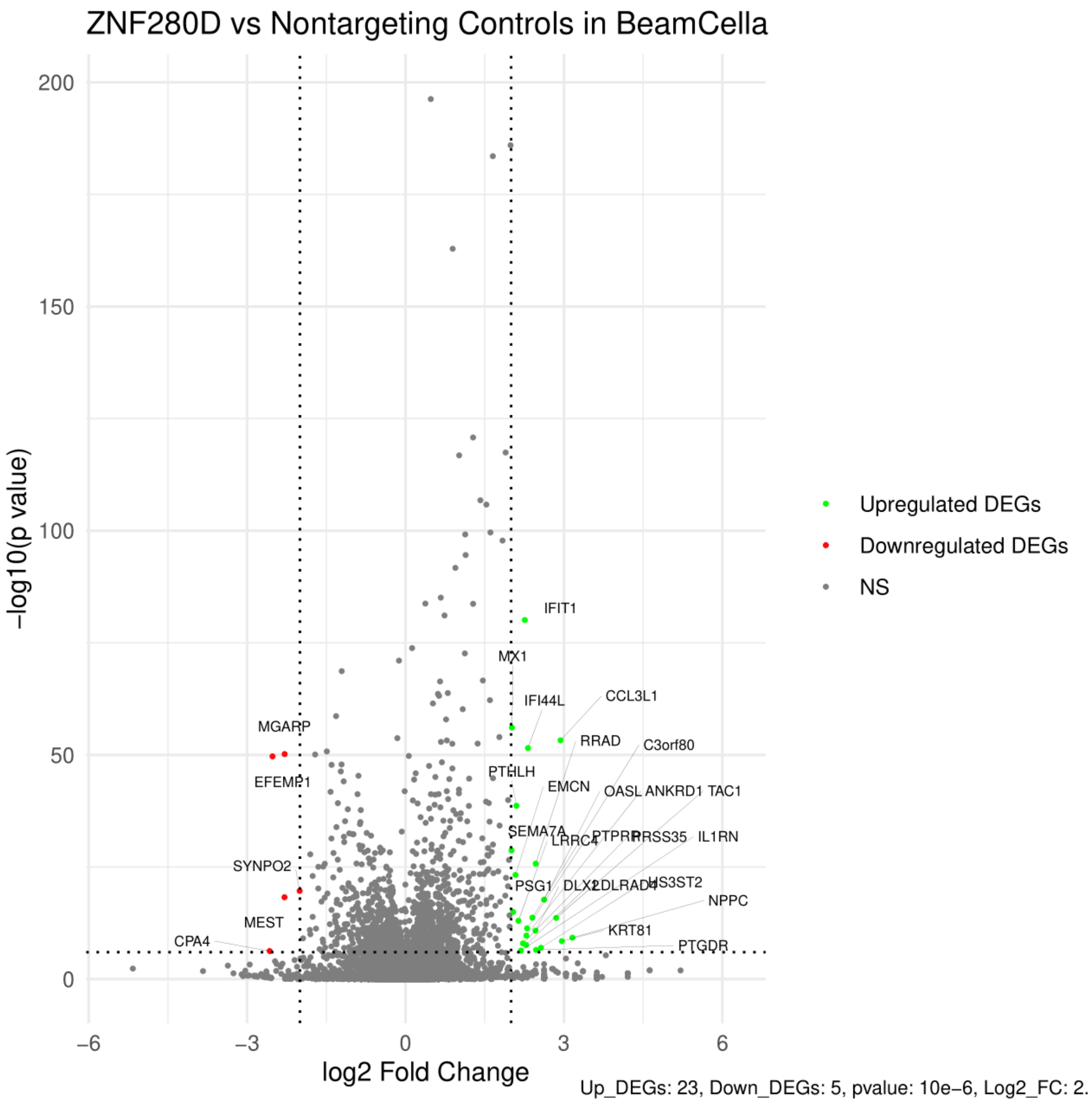
ZNF280D vs Nontargeting Controls in BeamCella
200
150
100
50
0
−log10(p value)
−6
−3
0
3
6
log2 Fold Change
IFIT1
MX1
IFI44L
CCL3L1
MGARP
RRAD
C3orf80
EFEMP1
PTHLH
EMCN
OASL
ANKRD1
TAC1
SEMA7A
LRRC4
PTPRR
PRSS35
IL1RN
SYNPO2
PSG1
DLX2
LDLRAD4
HS3ST2
NPPC
MEST
KRT81
CPA4
PTGDR
Upregulated DEGs
Downregulated DEGs
NS
Up_DEGs: 23, Down_DEGs: 5, pvalue: 10e−6, Log2_FC: 2.

Supplementary Figure 12:

Differential expressed genes between gene knockout and non-targeting control Fibroblast-like Cells. The volcano plots display the Log2 fold change versus -log_{10}(P value) for the gene-targeting cells and control non-targeting cells.

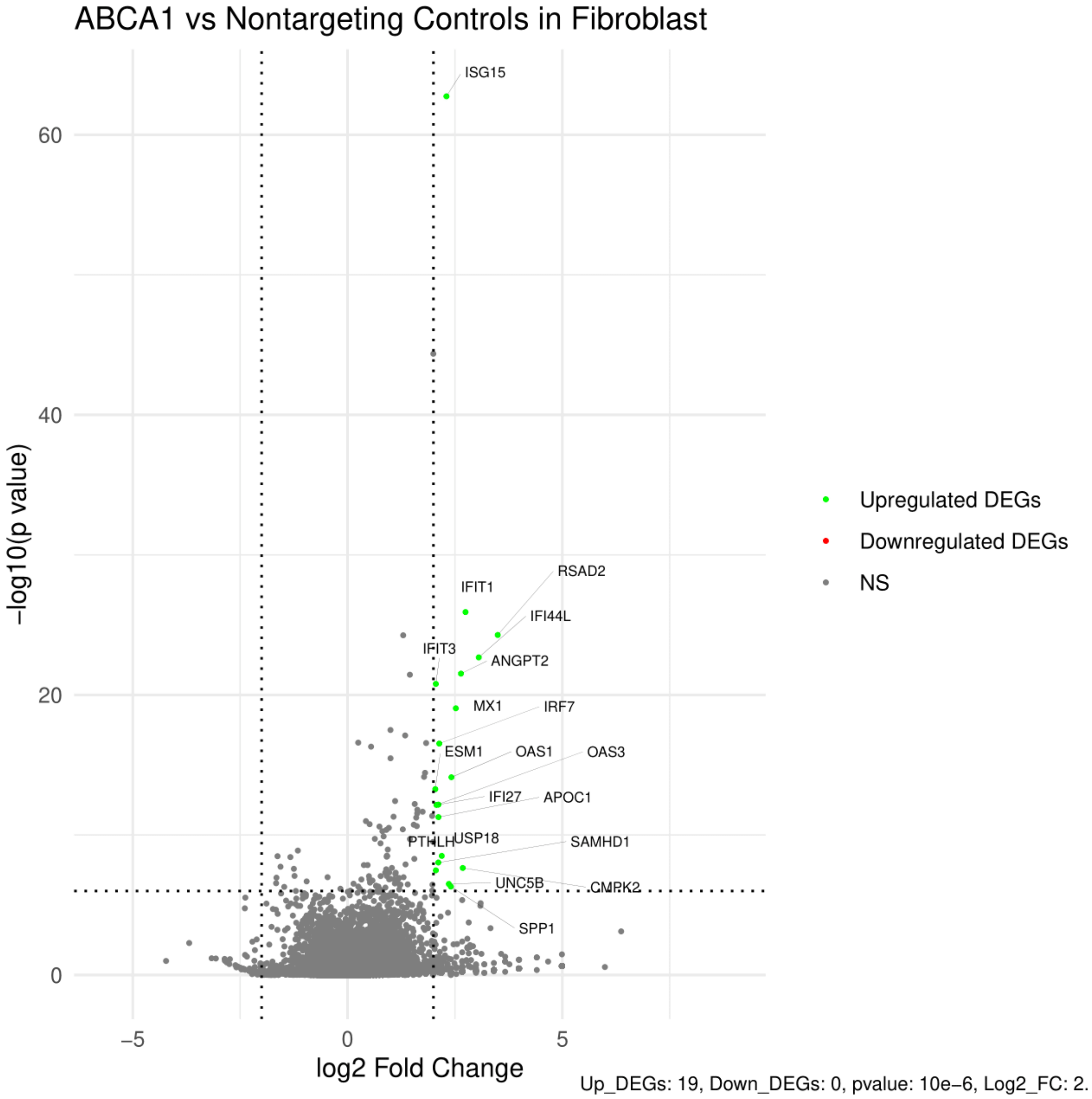
ABCA1 vs Nontargeting Controls in Fibroblast
−log10(p value)
log2 Fold Change
ISG15
IFIT1
RSAD2
IFI44L
IFIT3
ANGPT2
MX1
IRF7
ESM1
OAS1
OAS3
IFI27
APOC1
PTHLH
USP18
SAMHD1
UNC5B
CMPK2
SPP1
Upregulated DEGs
Downregulated DEGs
NS
Up_DEGs: 19, Down_DEGs: 0, pvalue: 10e−6, Log2_FC: 2.

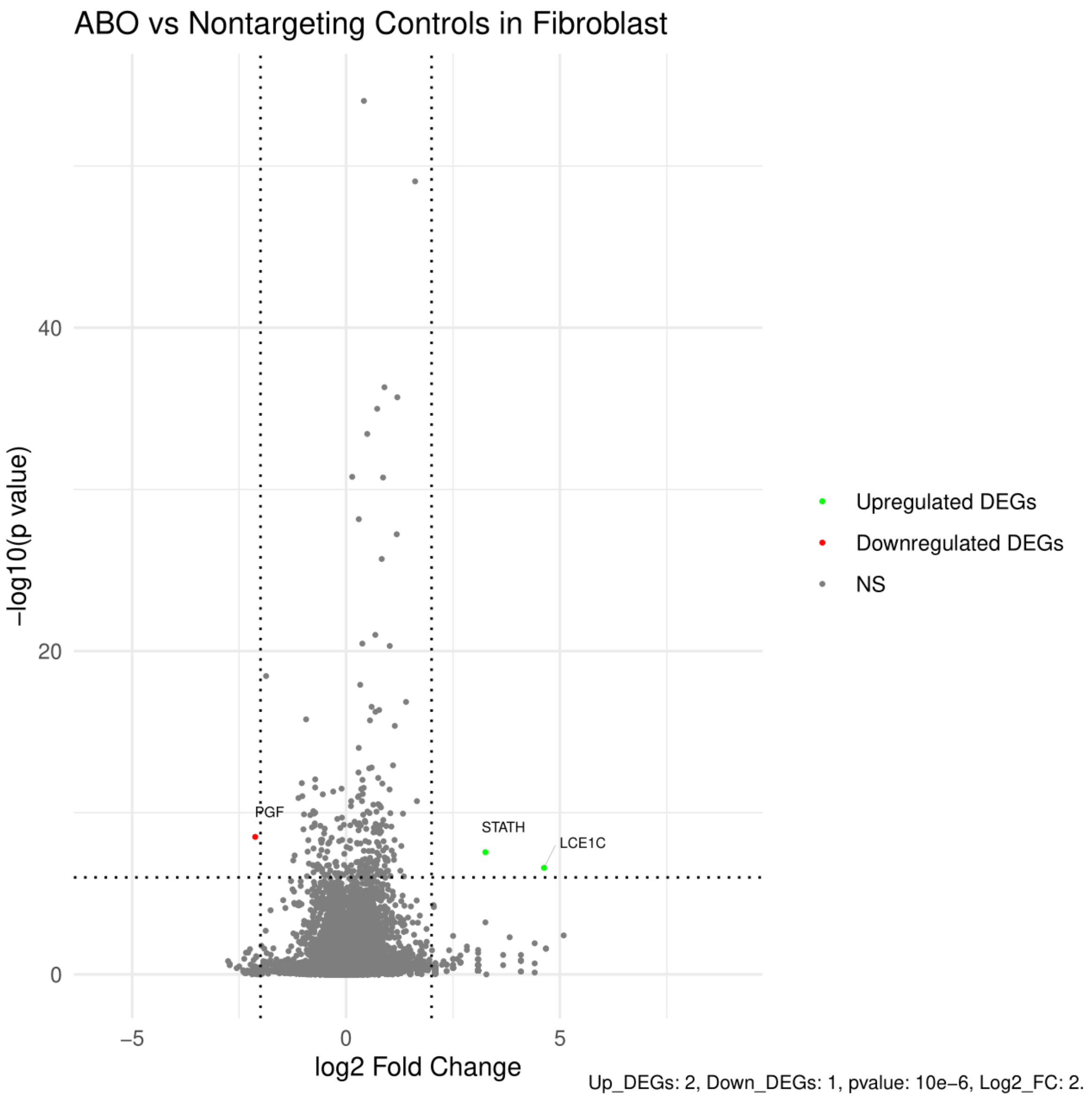
ABO vs Nontargeting Controls in Fibroblast
−log10(p value)
40
20
0
−5
0
5
log2 Fold Change
PGF
STATH
LCE1C
Upregulated DEGs
Downregulated DEGs
NS
Up_DEGs: 2, Down_DEGs: 1, pvalue: 10e−6, Log2_FC: 2.

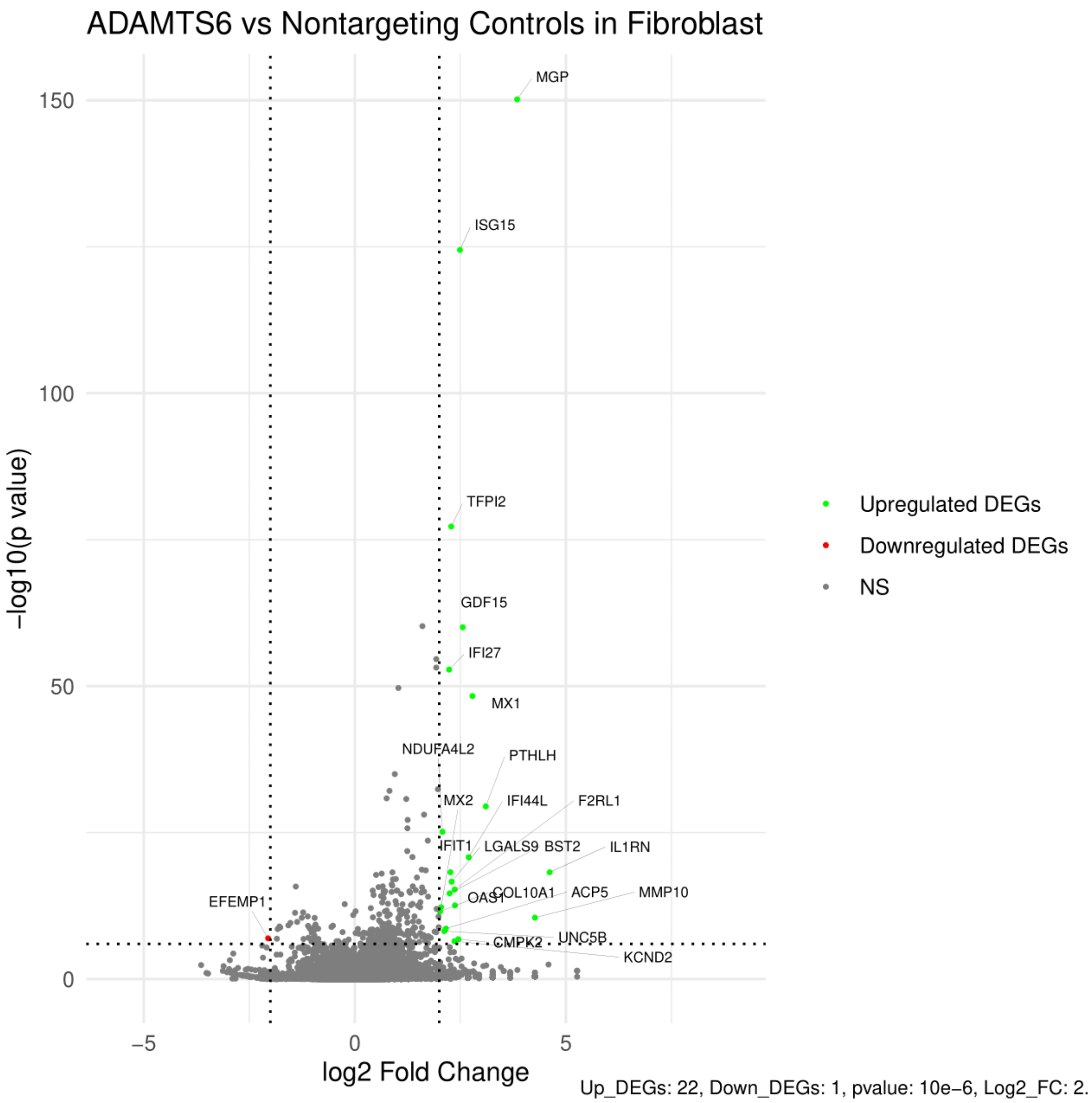

ADAMTS6 vs Nontargeting Controls in Fibroblast
−log10(p value)
log2 Fold Change
150
100
50
0
−5
0
5
MGP
ISG15
TFPI2
GDF15
IFI27
MX1
NDUFA4L2
PTHLH
MX2
IFI44L
F2RL1
IFIT1
LGALS9
BST2
IL1RN
EFEMP1
OAS1
COL10A1
ACP5
MMP10
CMPK2
UNC5B
KCND2
Upregulated DEGs
Downregulated DEGs
NS
Up_DEGs: 22, Down_DEGs: 1, pvalue: 10e−6, Log2_FC: 2.

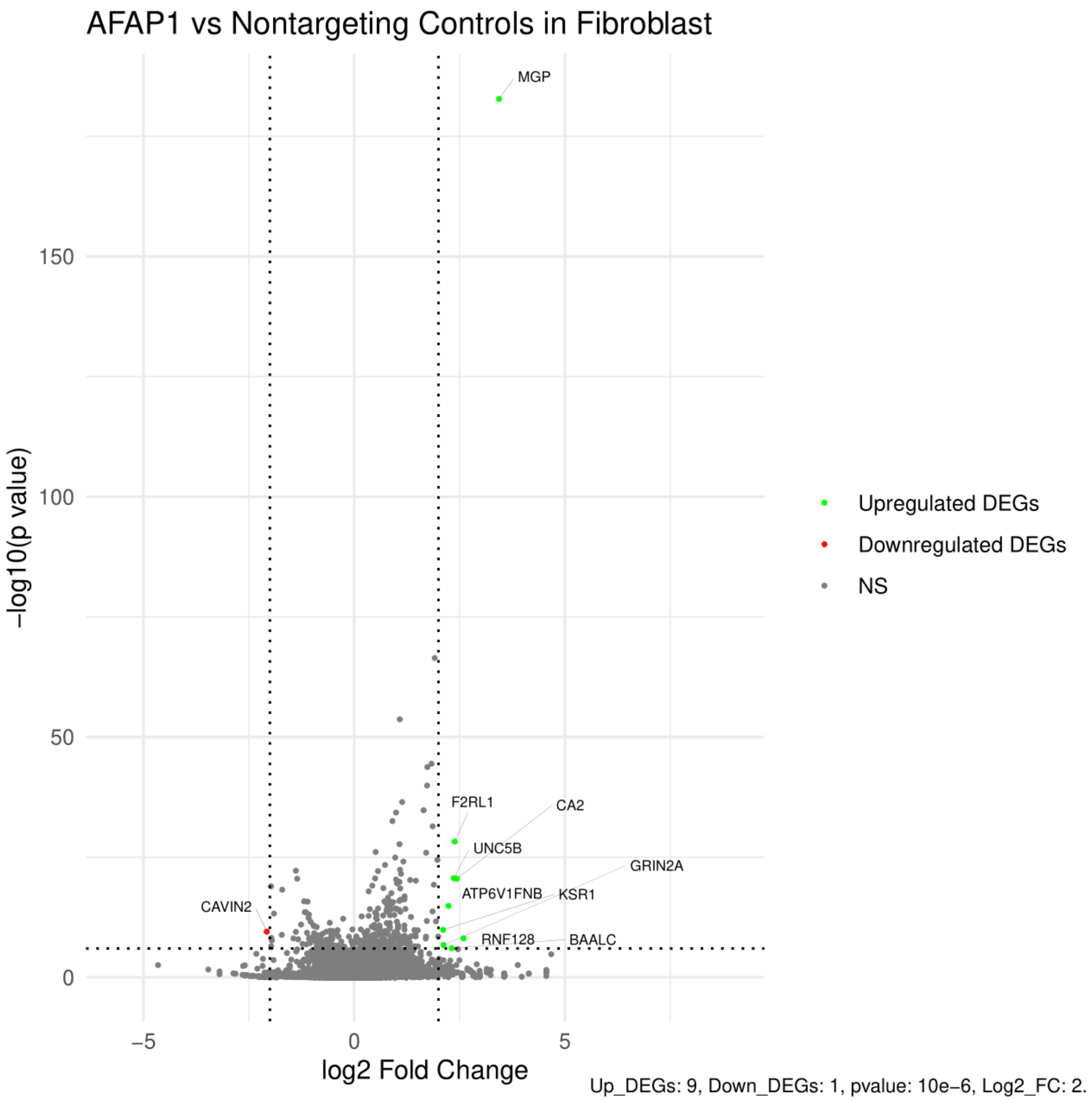
AFAP1 vs Nontargeting Controls in Fibroblast
MGP
F2RL1
CA2
UNC5B
GRIN2A
ATP6V1FNB
KSR1
CAVIN2
RNF128
BAALC
150
100
50
0
−log10(p value)
−5
0
5
log2 Fold Change
Upregulated DEGs
Downregulated DEGs
NS
Up_DEGs: 9, Down_DEGs: 1, pvalue: 10e−6, Log2_FC: 2.

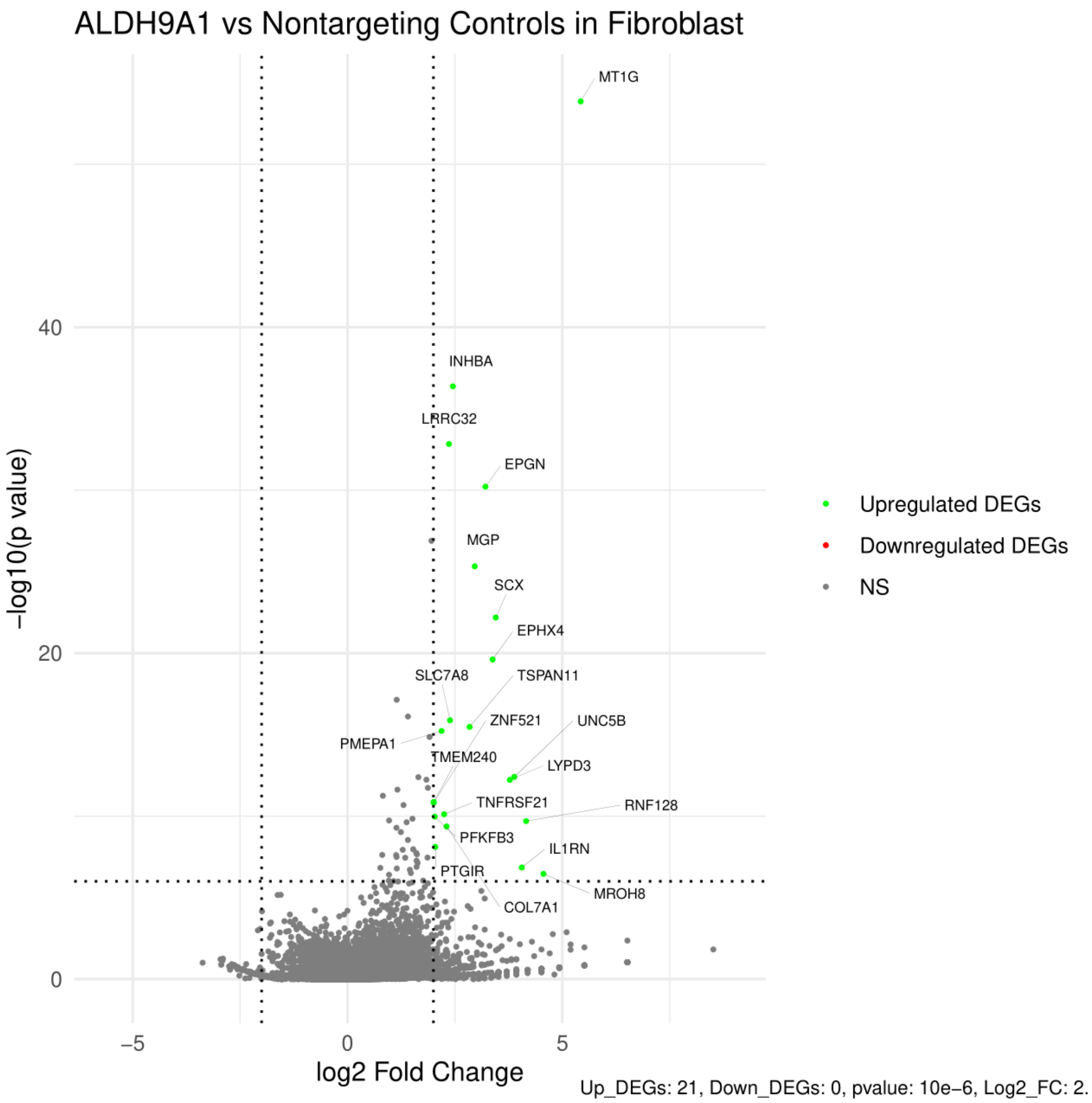
ALDH9A1 vs Nontargeting Controls in Fibroblast
−log10(p value)
log2 Fold Change
40
20
0
−5
0
5
MT1G
INHBA
LRRC32
EPGN
MGP
SCX
EPHX4
SLC7A8
TSPAN11
ZNF521
UNC5B
PMEPA1
TMEM240
LYPD3
TNFRSF21
RNF128
PFKFB3
IL1RN
PTGIR
MROH8
COL7A1
Upregulated DEGs
Downregulated DEGs
NS
Up_DEGs: 21, Down_DEGs: 0, pvalue: 10e−6, Log2_FC: 2.

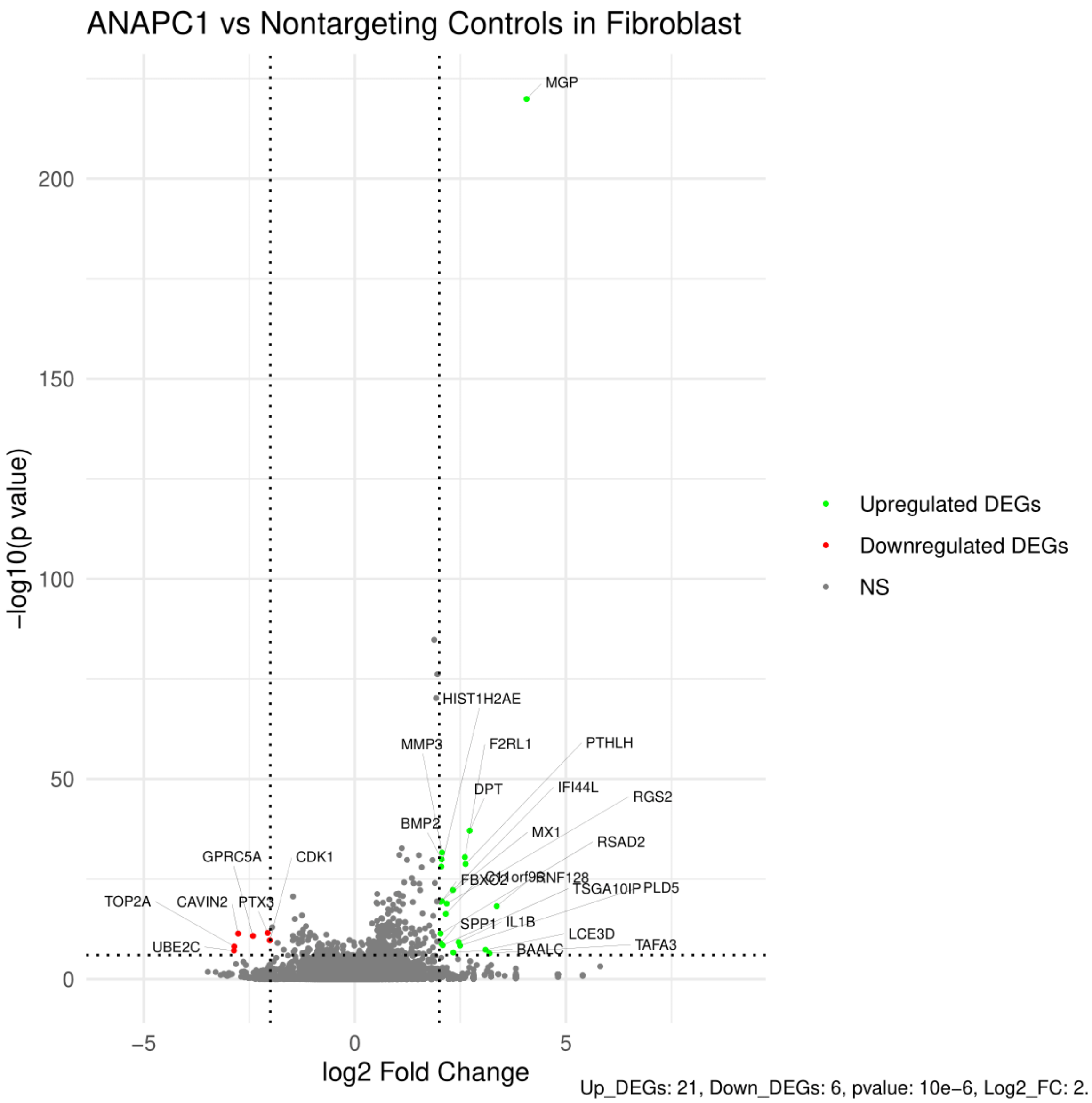
ANAPC1 vs Nontargeting Controls in Fibroblast
-log10(p value)
log2 Fold Change
Upregulated DEGs
Downregulated DEGs
NS
MGP
HIST1H2AE
MMP3
F2RL1
PTHLH
DPT
IFI44L
RGS2
BMP2
MX1
RSAD2
GPRC5A
CDK1
FBXO2
C11orf96
RNF128
TSGA10IP
PLD5
TOP2A
CAVIN2
PTX3
SPP1
IL1B
LCE3D
UBE2C
BAALC
TAFA3
Up_DEGs: 21, Down_DEGs: 6, pvalue: 10e-6, Log2_FC: 2.

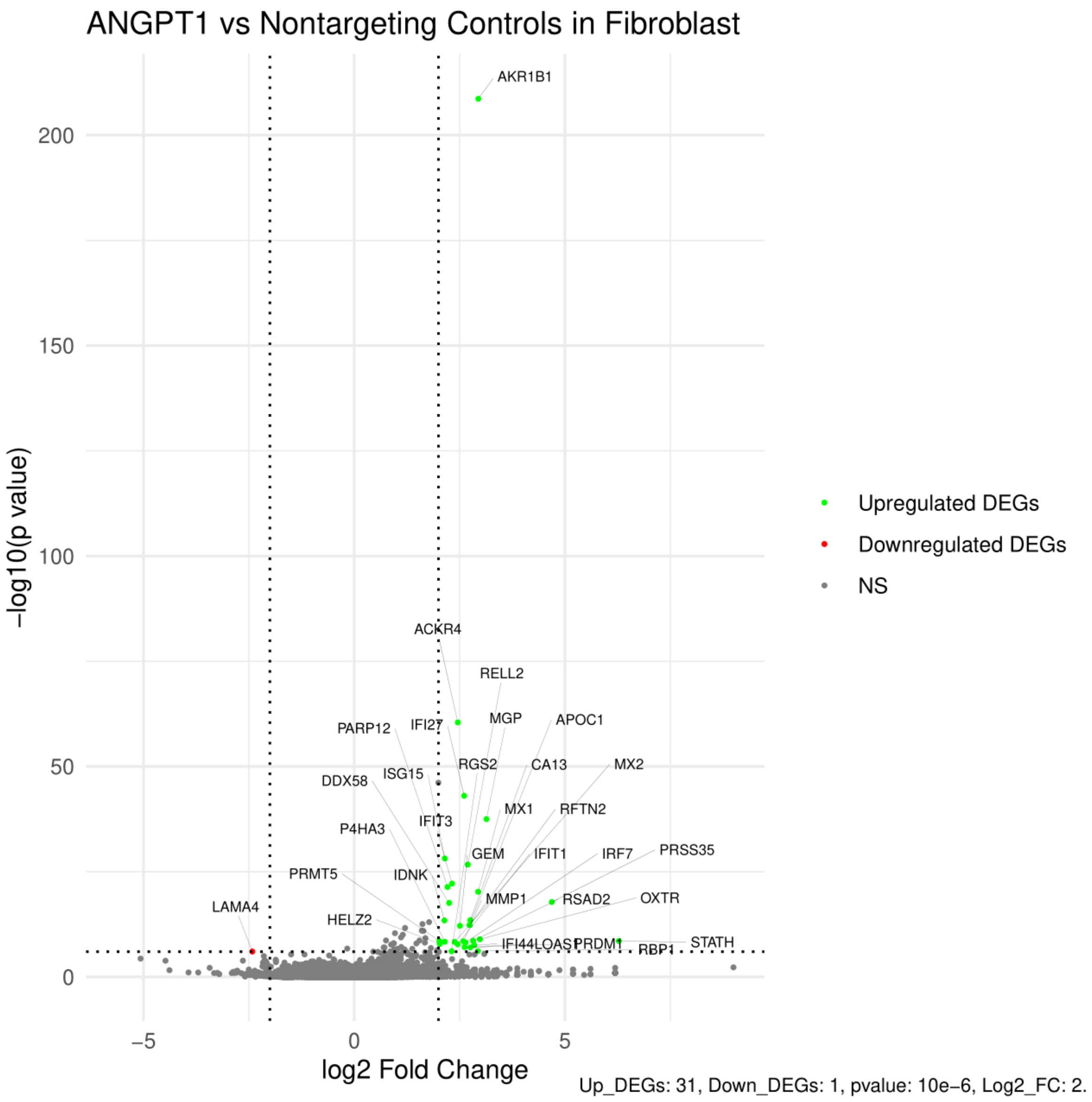
ANGPT1 vs Nontargeting Controls in Fibroblast
AKR1B1
ACKR4
RELL2
PARP12
IFI27
MGP
APOC1
DDX58
ISG15
RGS2
CA13
MX2
P4HA3
IFIT3
MX1
RFTN2
GEM
IFIT1
IRF7
PRSS35
PRMT5
IDNK
LAMA4
MMP1
RSAD2
OXTR
HELZ2
IFI44LOASPRDM1
RBP1
STATH
200
150
100
50
0
−log10(p value)
−5
0
5
log2 Fold Change
Upregulated DEGs
Downregulated DEGs
NS
Up_DEGs: 31, Down_DEGs: 1, pvalue: 10e−6, Log2_FC: 2.

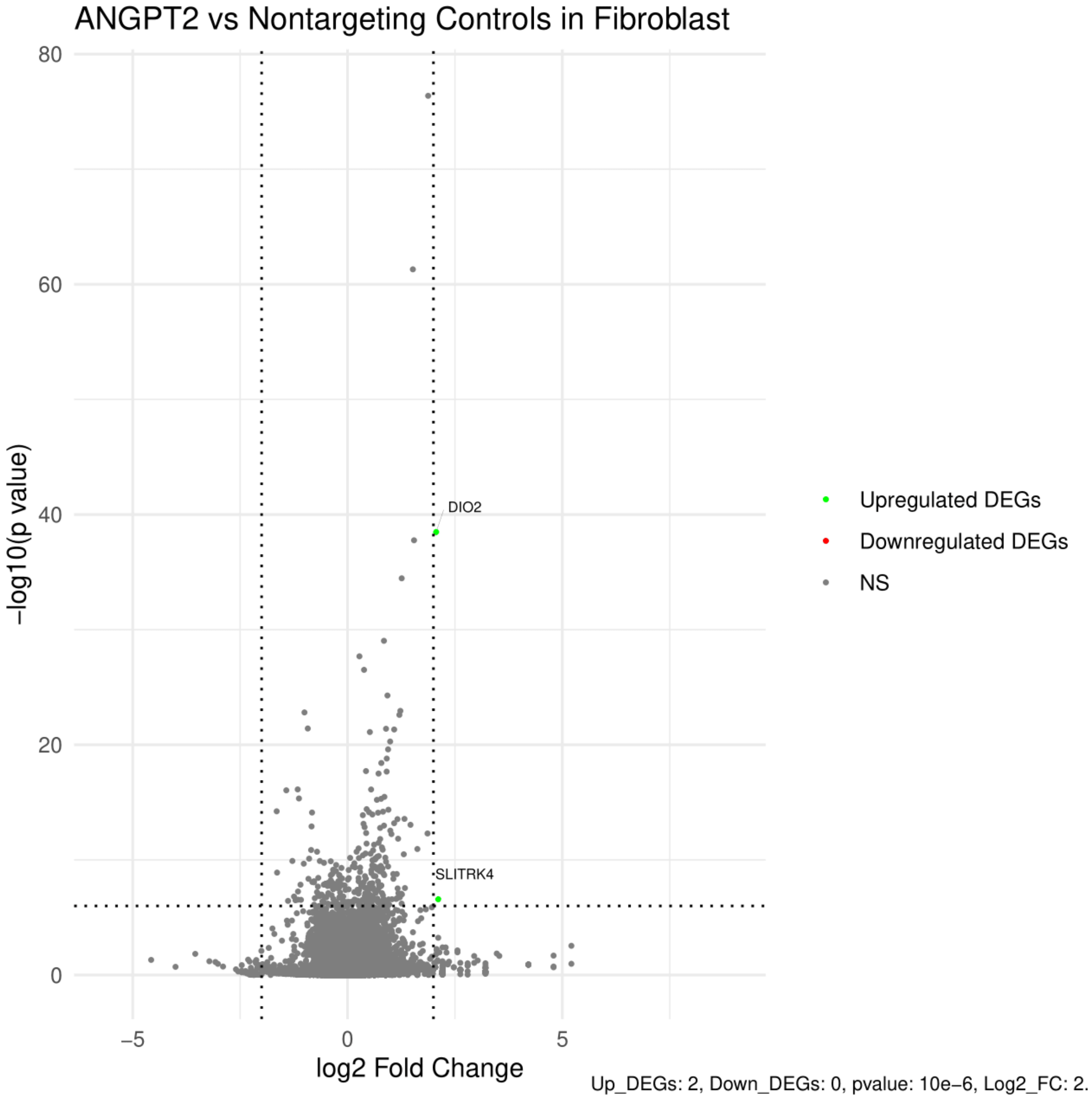
ANGPT2 vs Nontargeting Controls in Fibroblast
80
60
40
20
0
−log10(p value)
DIO2
SLITRK4
−5
0
5
log2 Fold Change
Upregulated DEGs
Downregulated DEGs
NS
Up_DEGs: 2, Down_DEGs: 0, pvalue: 10e−6, Log2_FC: 2.

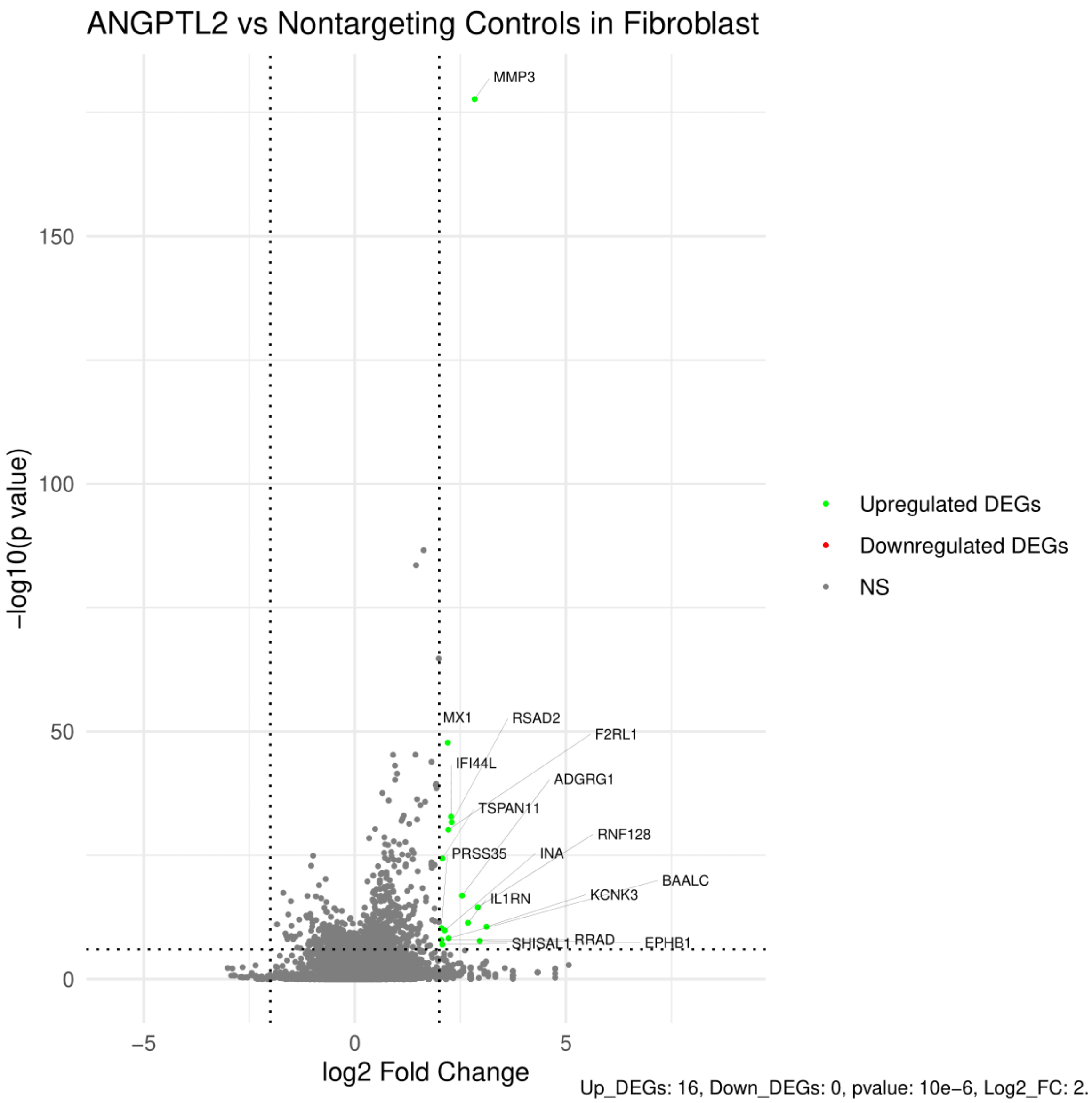
ANGPTL2 vs Nontargeting Controls in Fibroblast
MMP3
MX1
RSAD2
F2RL1
IFI44L
ADGRG1
TSPAN11
RNF128
PRSS35
INA
BAALC
IL1RN
KCNK3
SHISAL1
RRAD
EPHB1
150
100
50
0
−log10(p value)
−5
0
5
log2 Fold Change
Upregulated DEGs
Downregulated DEGs
NS
Up_DEGs: 16, Down_DEGs: 0, pvalue: 10e−6, Log2_FC: 2.

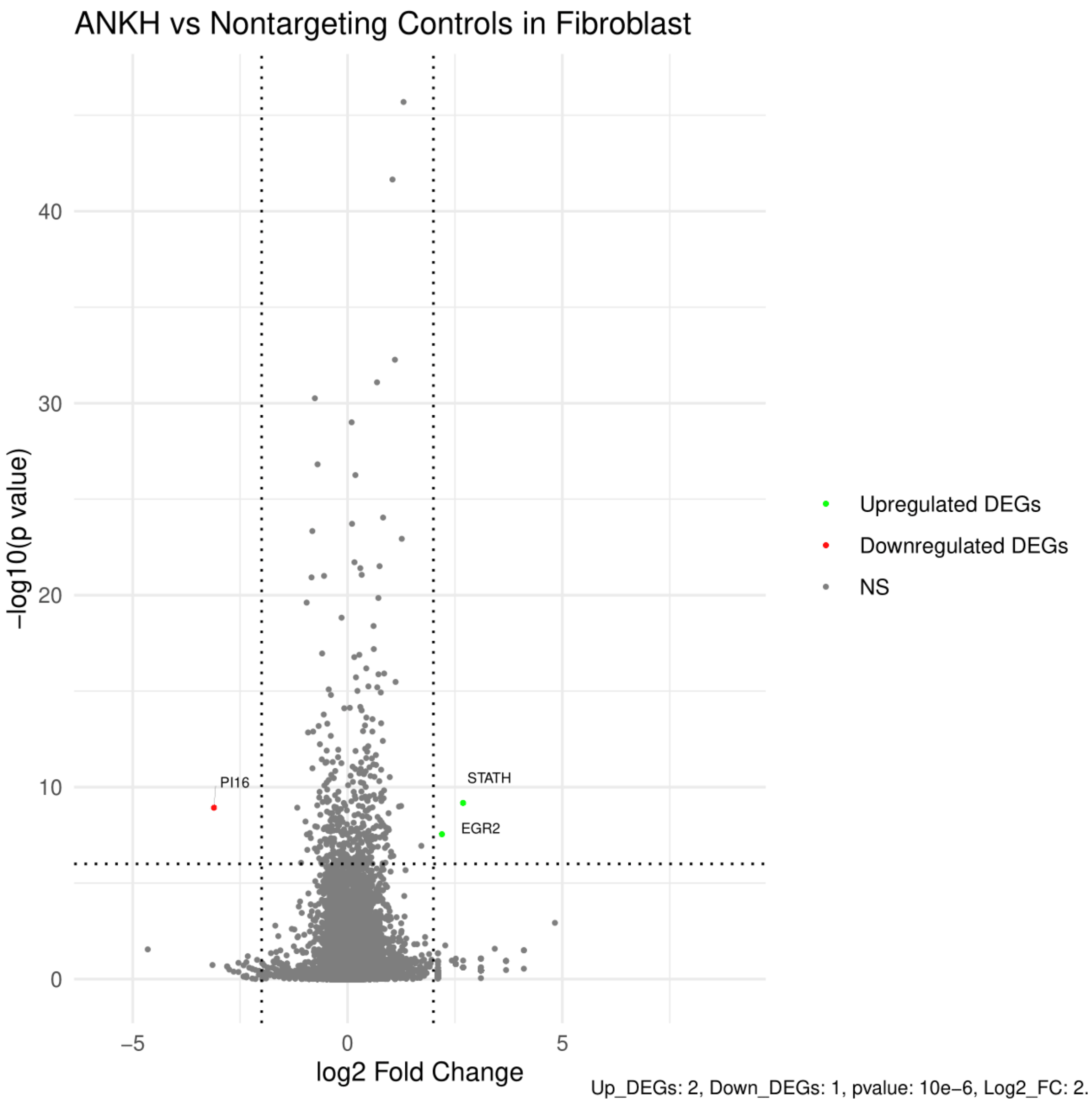
ANKH vs Nontargeting Controls in Fibroblast
−log10(p value)
40
30
20
10
0
−5
0
5
log2 Fold Change
PI16
STATH
EGR2
Upregulated DEGs
Downregulated DEGs
NS
Up_DEGs: 2, Down_DEGs: 1, pvalue: 10e−6, Log2_FC: 2.

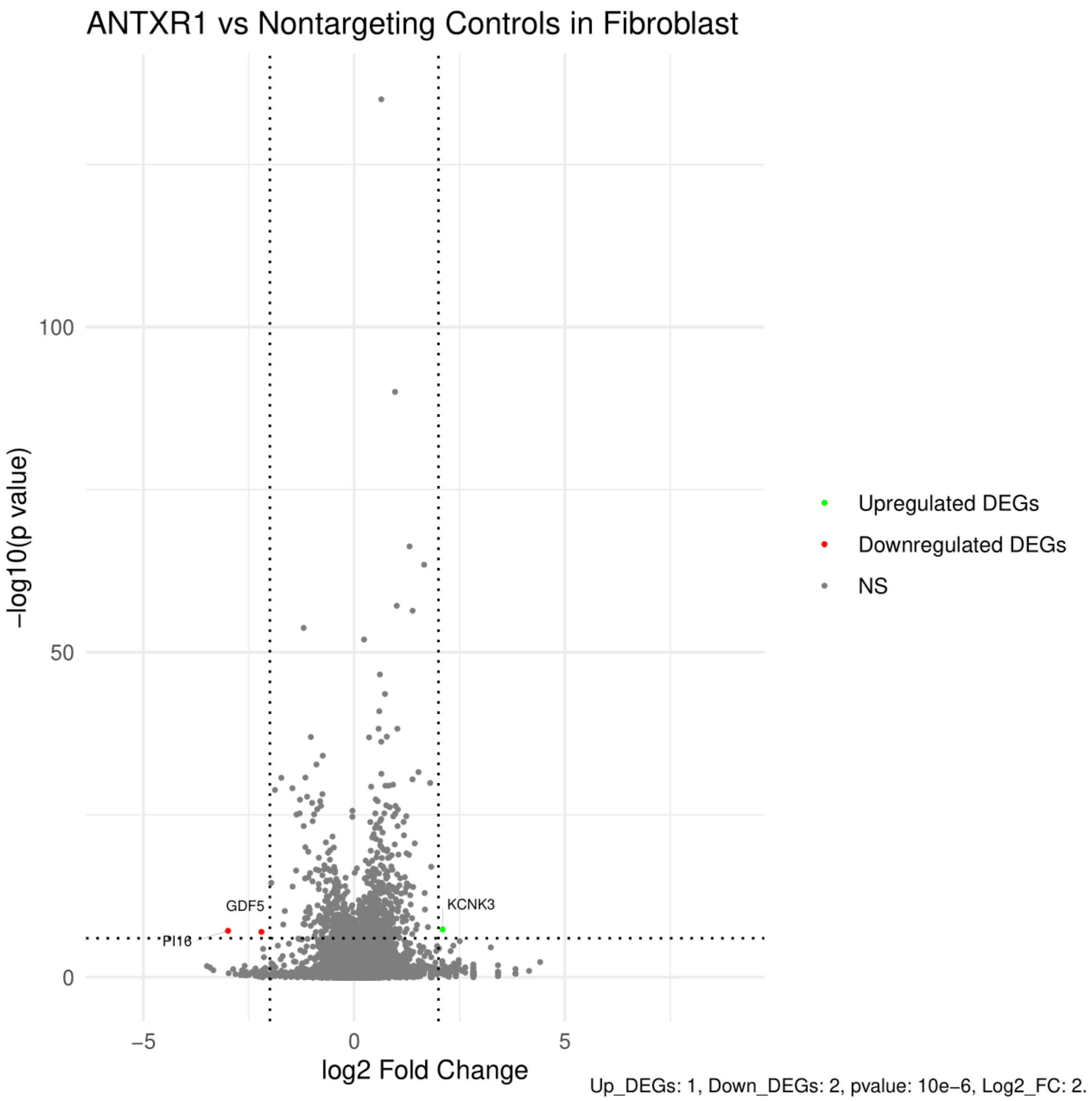

ANTXR1 vs Nontargeting Controls in Fibroblast
100
50
0
−log10(p value)
−5
0
5
log2 Fold Change
GDF5
PI16
KCNK3
Upregulated DEGs
Downregulated DEGs
NS
Up_DEGs: 1, Down_DEGs: 2, pvalue: 10e−6, Log2_FC: 2.

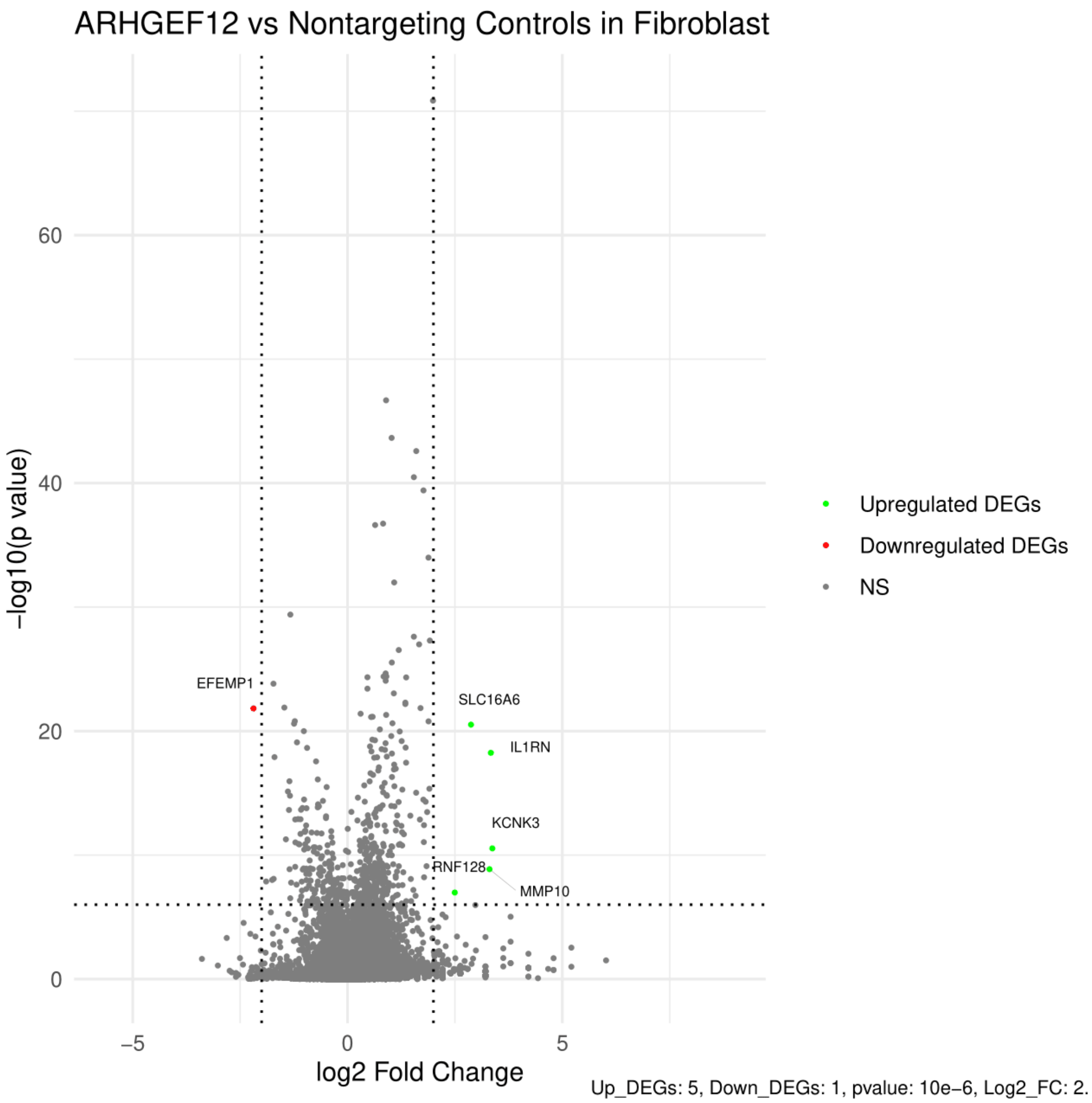
ARHGEF12 vs Nontargeting Controls in Fibroblast
−log10(p value)
60
40
20
0
−5
0
5
log2 Fold Change
EFEMP1
SLC16A6
IL1RN
KCNK3
RNF128
MMP10
Upregulated DEGs
Downregulated DEGs
NS
Up_DEGs: 5, Down_DEGs: 1, pvalue: 10e−6, Log2_FC: 2.

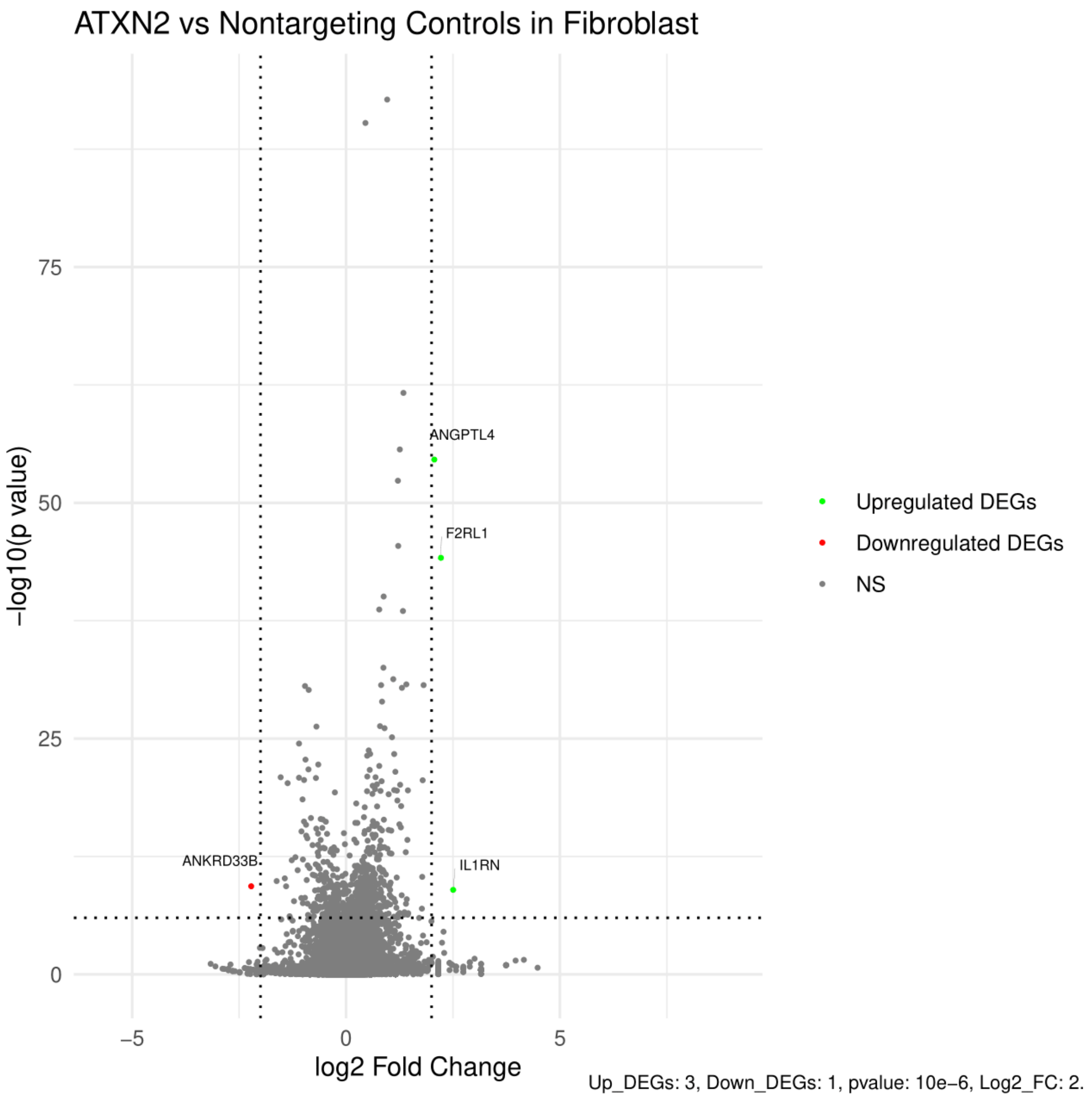

ATXN2 vs Nontargeting Controls in Fibroblast
ANGPTL4
F2RL1
ANKRD33B
IL1RN
75
50
25
0
−log10(p value)
−5
0
5
log2 Fold Change
Upregulated DEGs
Downregulated DEGs
NS
Up_DEGs: 3, Down_DEGs: 1, pvalue: 10e−6, Log2_FC: 2.

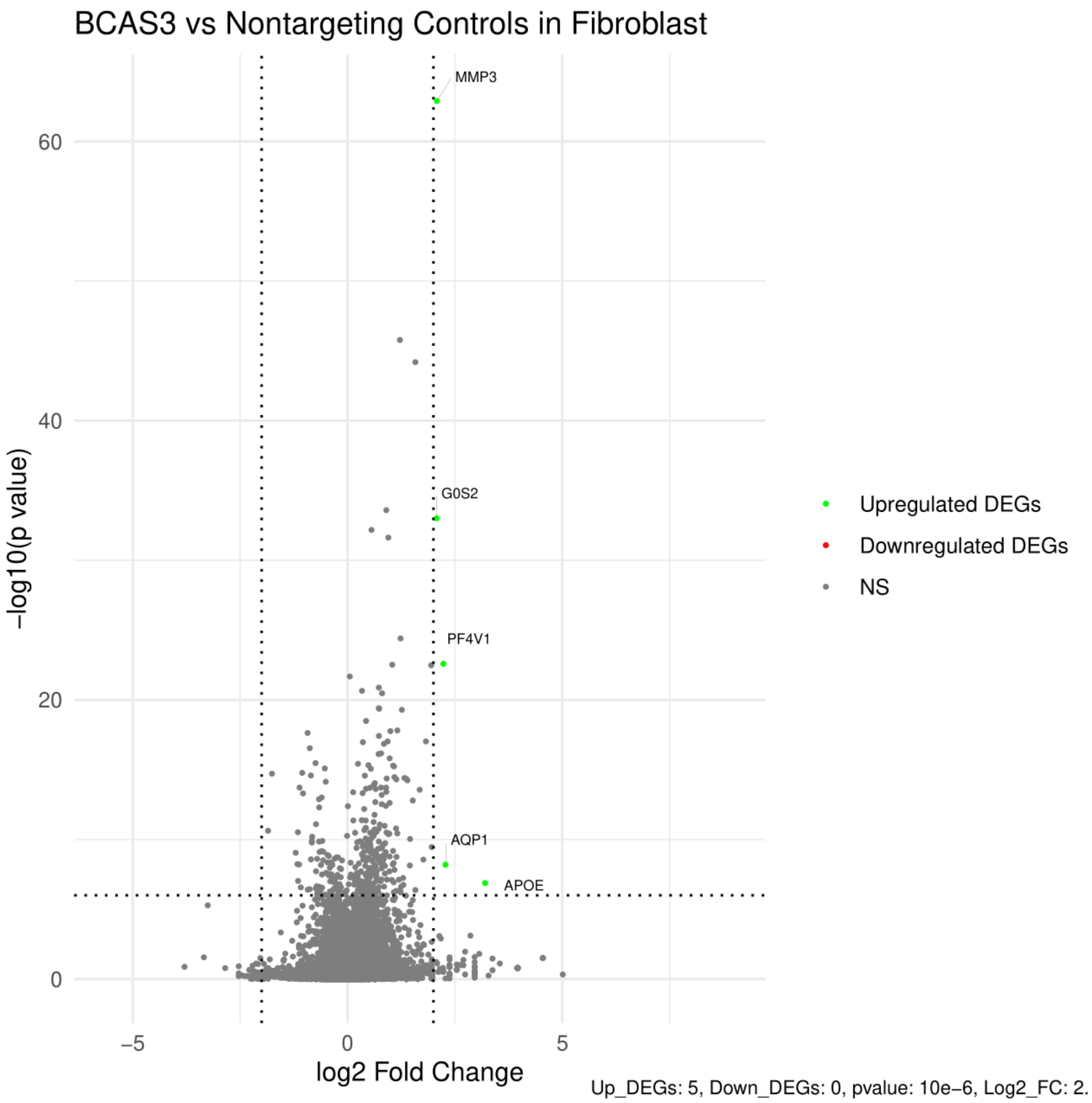
BCAS3 vs Nontargeting Controls in Fibroblast
MMP3
G0S2
PF4V1
AQP1
APOE
60
40
20
0
−log10(p value)
−5
0
5
log2 Fold Change
Upregulated DEGs
Downregulated DEGs
NS
Up_DEGs: 5, Down_DEGs: 0, pvalue: 10e−6, Log2_FC: 2.

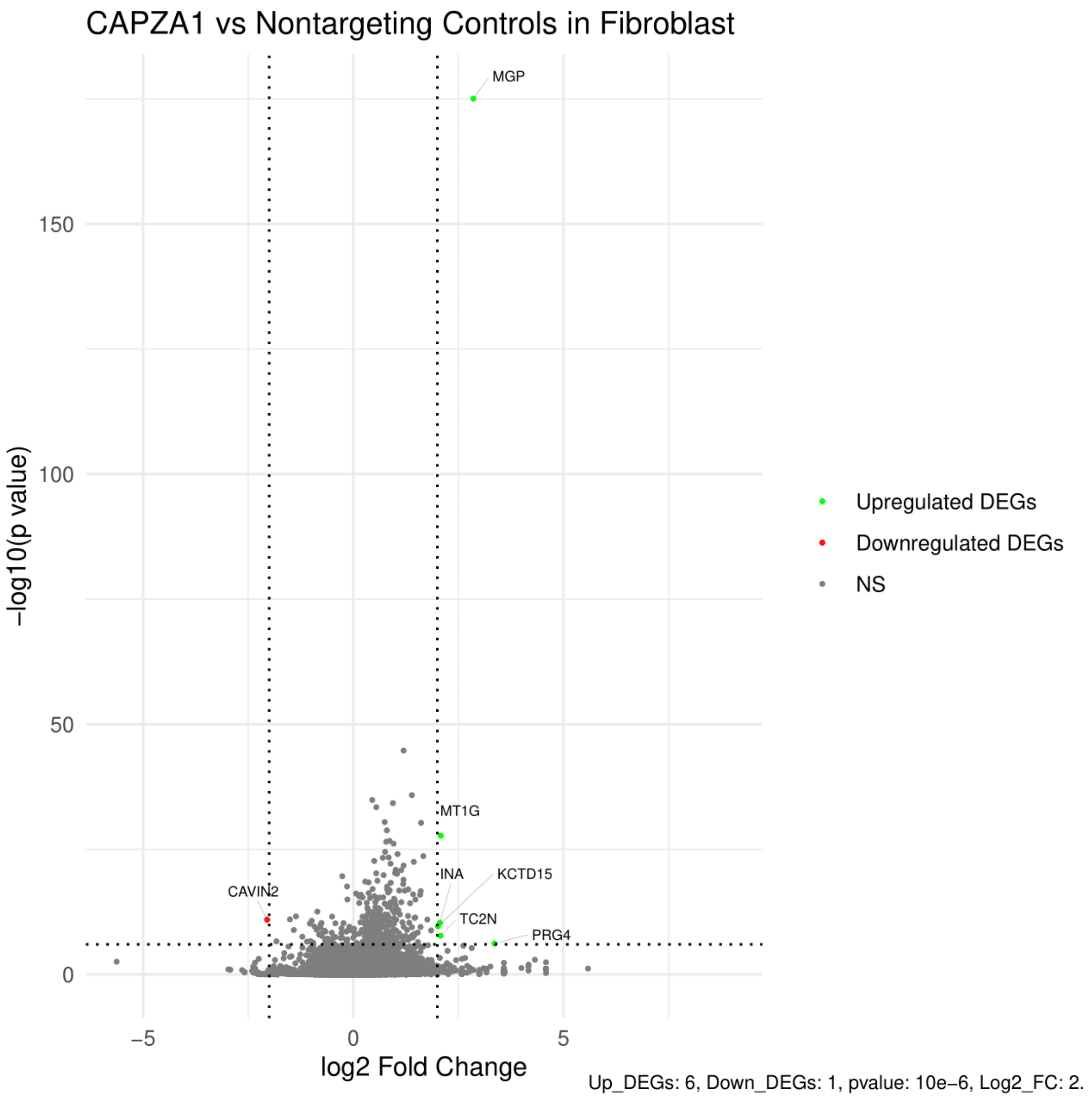

CAPZA1 vs Nontargeting Controls in Fibroblast
MGP
MT1G
INA
KCTD15
CAVIN2
TC2N
PRG4
150
100
50
0
−log10(p value)
−5
0
5
log2 Fold Change
Upregulated DEGs
Downregulated DEGs
NS
Up_DEGs: 6, Down_DEGs: 1, pvalue: 10e−6, Log2_FC: 2.

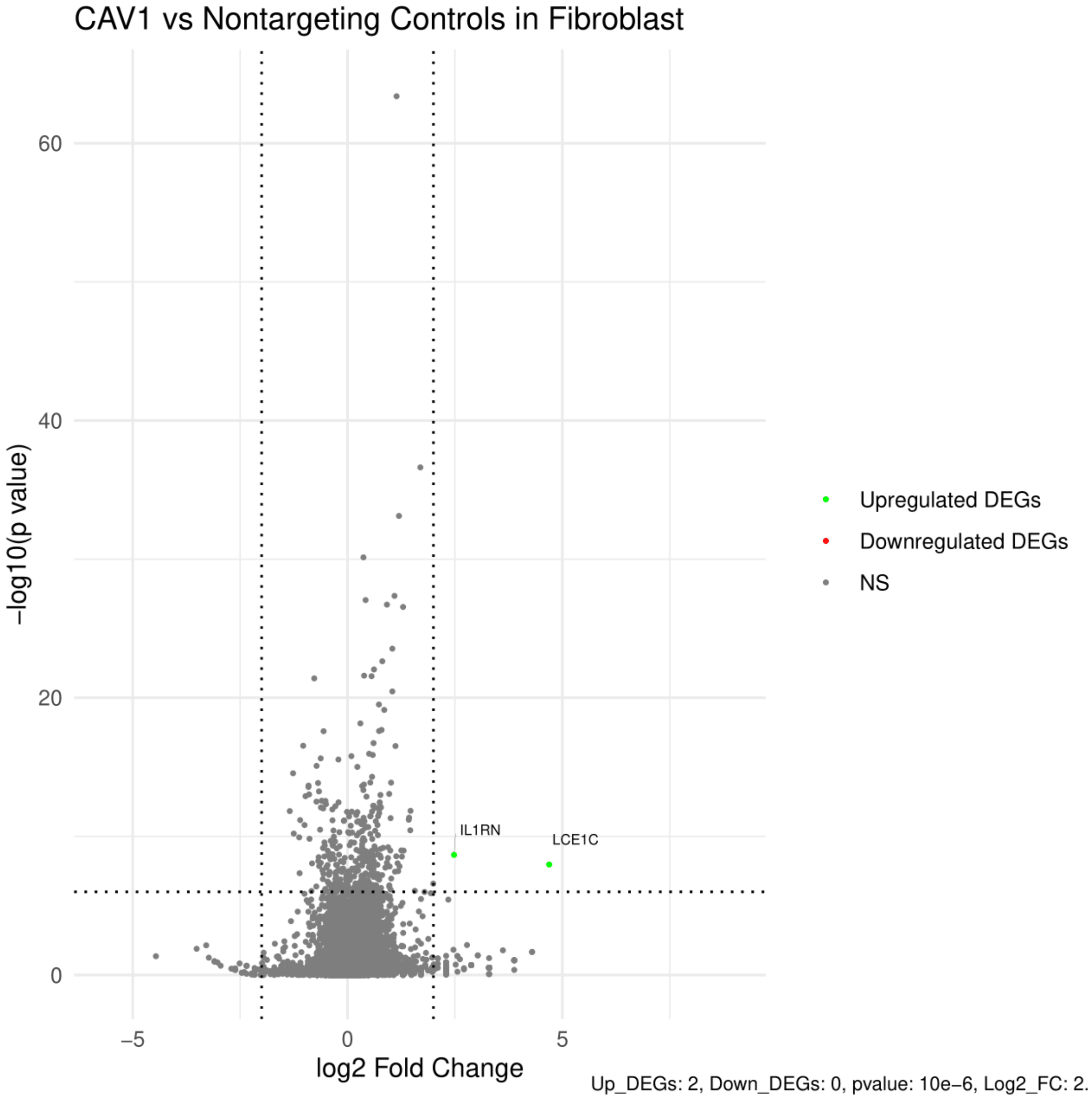
CAV1 vs Nontargeting Controls in Fibroblast
60
40
20
0
−log10(p value)
IL1RN
LCE1C
−5
0
5
log2 Fold Change
Upregulated DEGs
Downregulated DEGs
NS
Up_DEGs: 2, Down_DEGs: 0, pvalue: 10e−6, Log2_FC: 2.

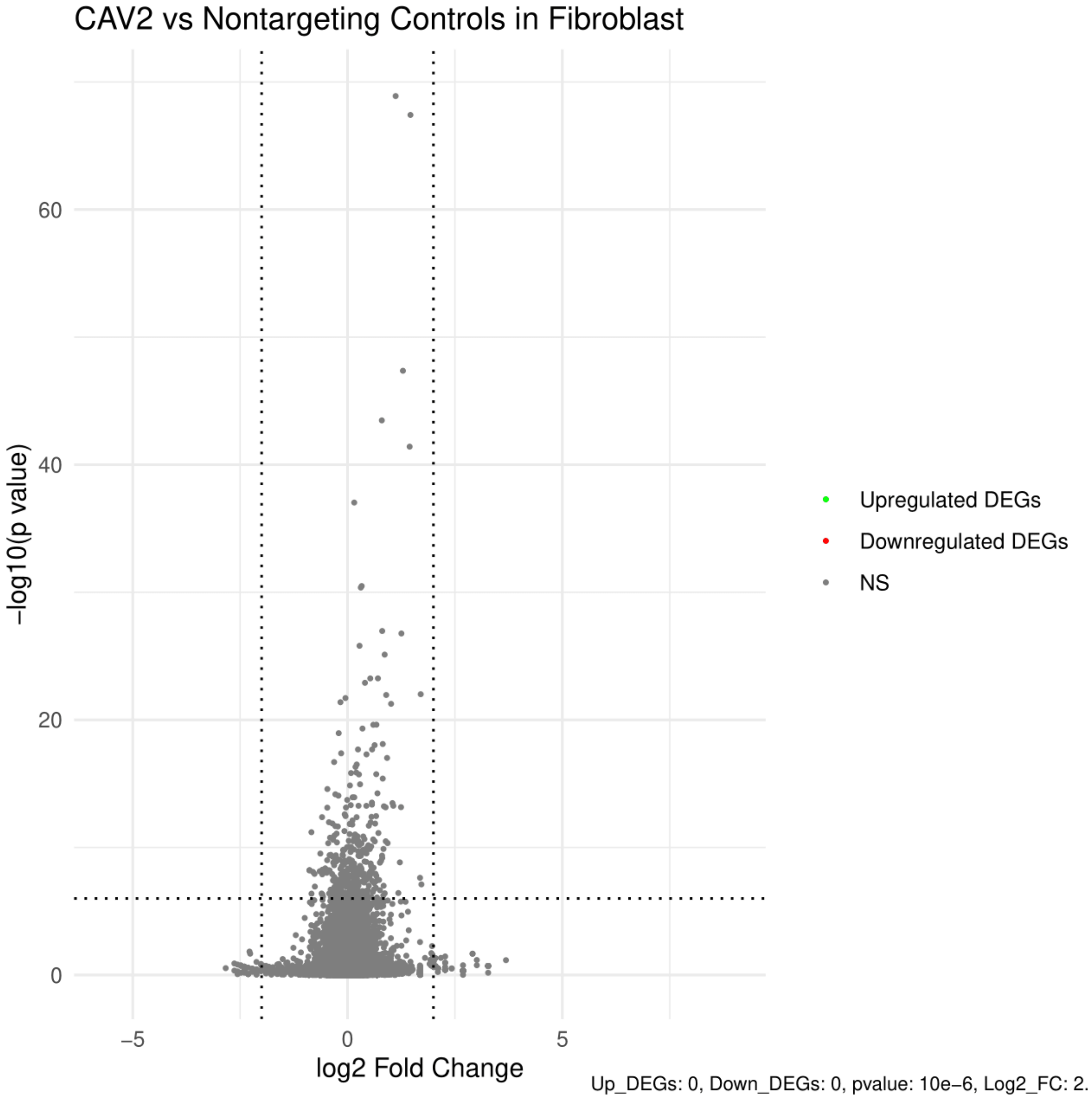
CAV2 vs Nontargeting Controls in Fibroblast
60
40
20
0
−log10(p value)
−5
0
5
log2 Fold Change
Upregulated DEGs
Downregulated DEGs
NS
Up_DEGs: 0, Down_DEGs: 0, pvalue: 10e−6, Log2_FC: 2.

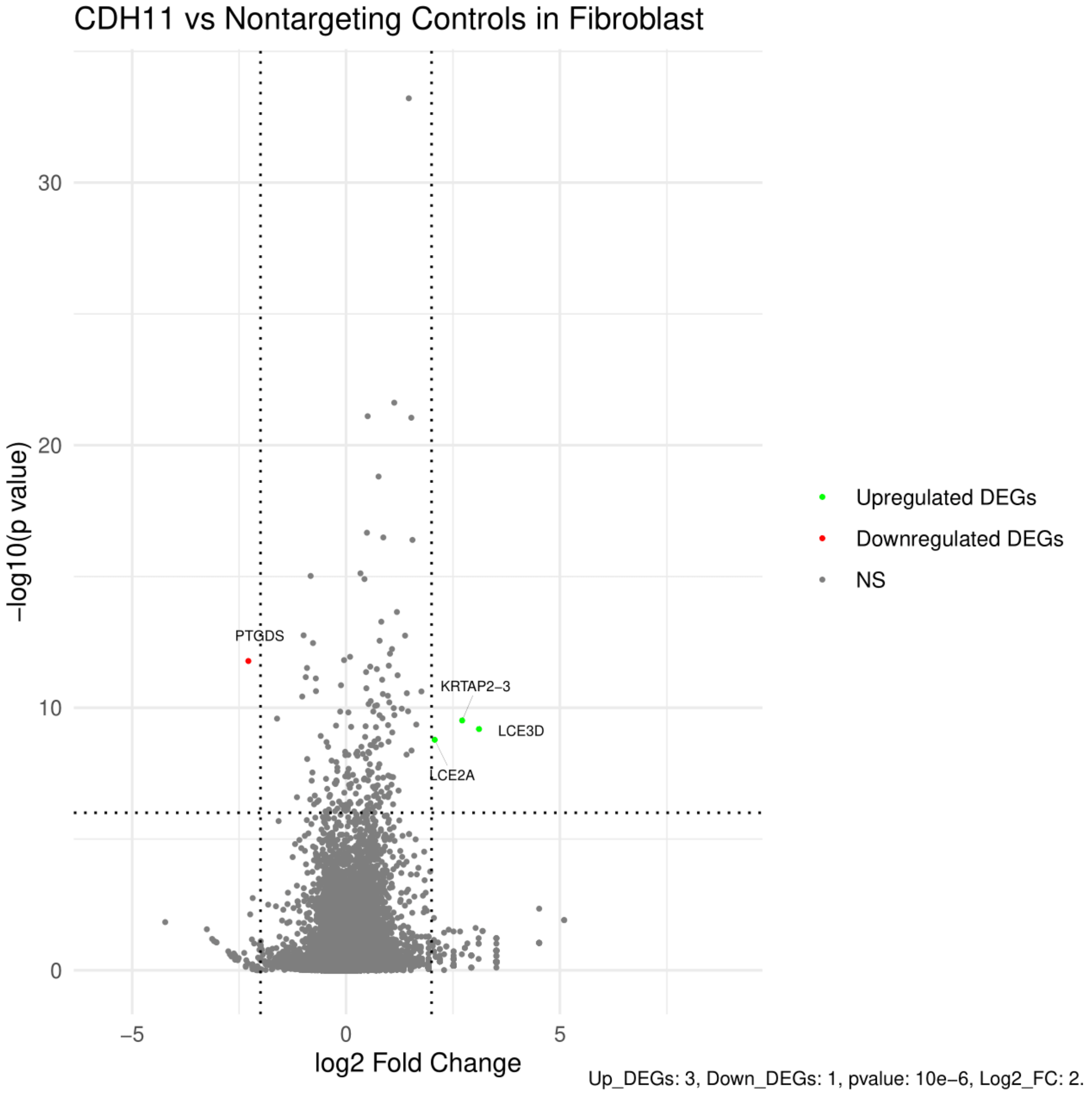
CDH11 vs Nontargeting Controls in Fibroblast
30
20
10
0
−log10(p value)
−5
0
5
log2 Fold Change
PTGDS
KRTAP2−3
LCE3D
LCE2A
Upregulated DEGs
Downregulated DEGs
NS
Up_DEGs: 3, Down_DEGs: 1, pvalue: 10e−6, Log2_FC: 2.

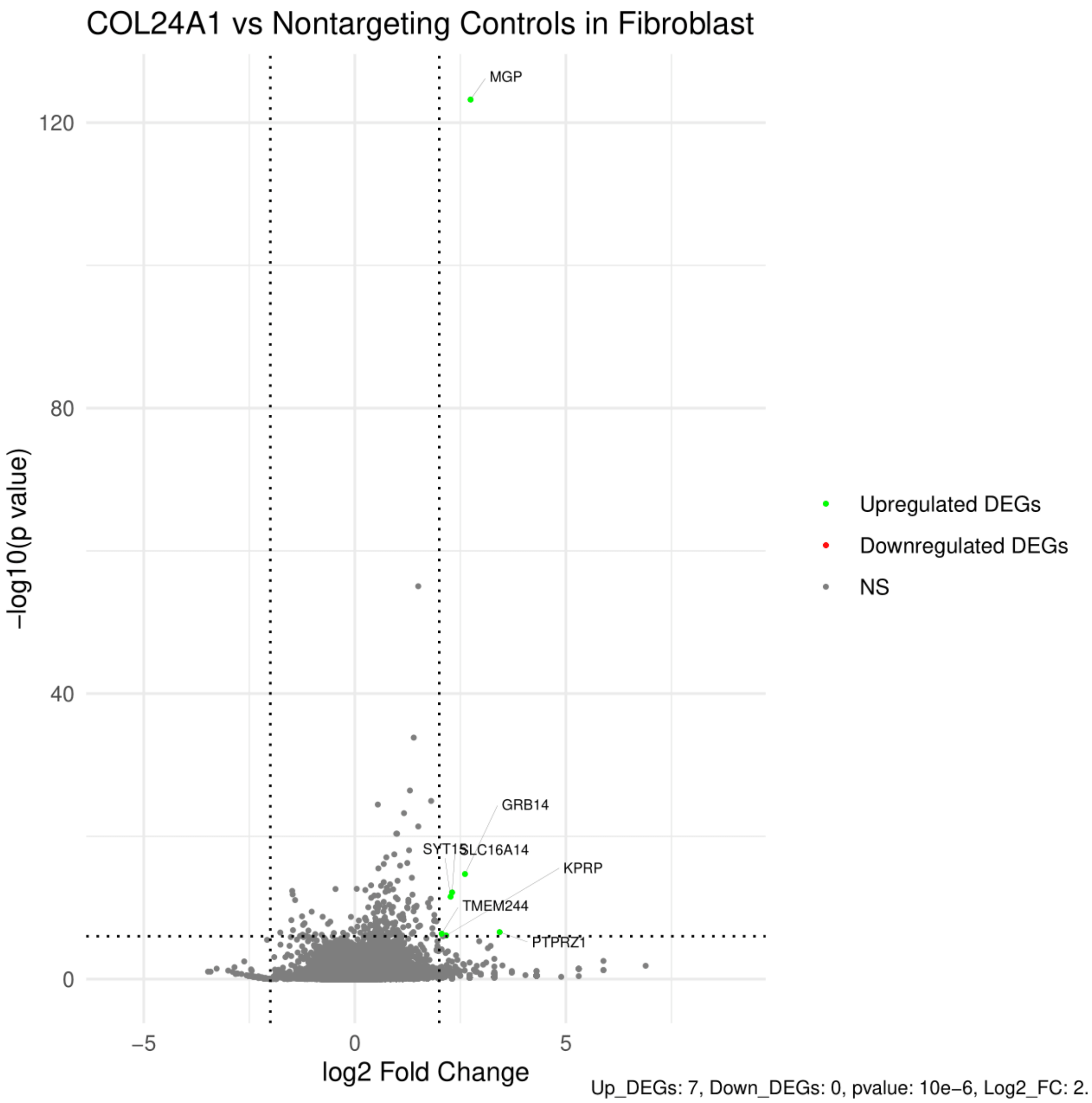

COL24A1 vs Nontargeting Controls in Fibroblast
MGP
GRB14
SYT15
SLC16A14
KPRP
TMEM244
PTPRZ1
120
80
40
0
−log10(p value)
−5
0
5
log2 Fold Change
Upregulated DEGs
Downregulated DEGs
NS
Up_DEGs: 7, Down_DEGs: 0, pvalue: 10e−6, Log2_FC: 2.

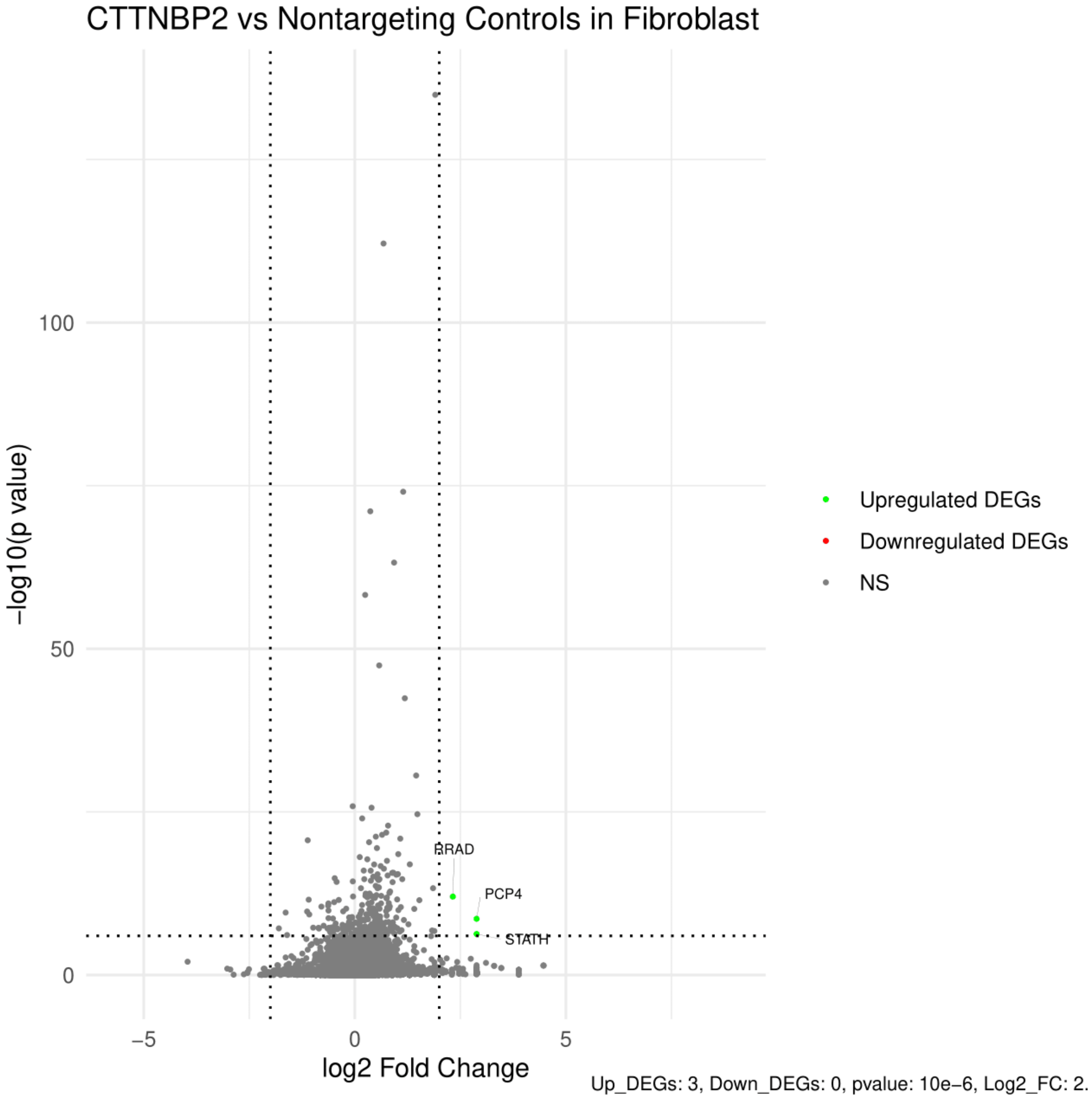
CTTNBP2 vs Nontargeting Controls in Fibroblast
100
50
0
−log10(p value)
−5
0
5
log2 Fold Change
RRAD
PCP4
STATH
Upregulated DEGs
Downregulated DEGs
NS
Up_DEGs: 3, Down_DEGs: 0, pvalue: 10e−6, Log2_FC: 2.

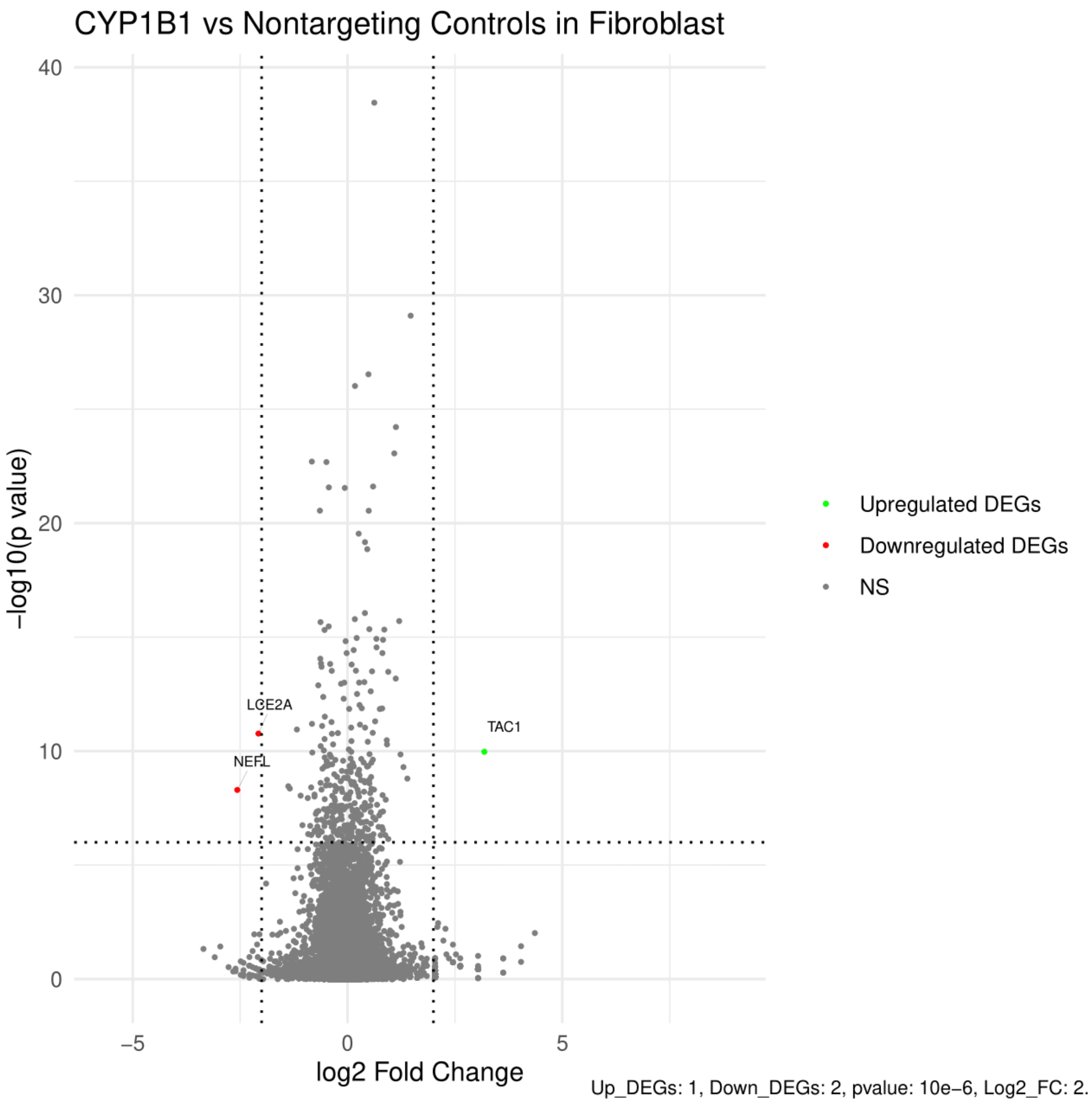
CYP1B1 vs Nontargeting Controls in Fibroblast
40
30
20
10
0
−log10(p value)
LCE2A
NEFL
TAC1
−5
0
5
log2 Fold Change
Upregulated DEGs
Downregulated DEGs
NS
Up_DEGs: 1, Down_DEGs: 2, pvalue: 10e−6, Log2_FC: 2.

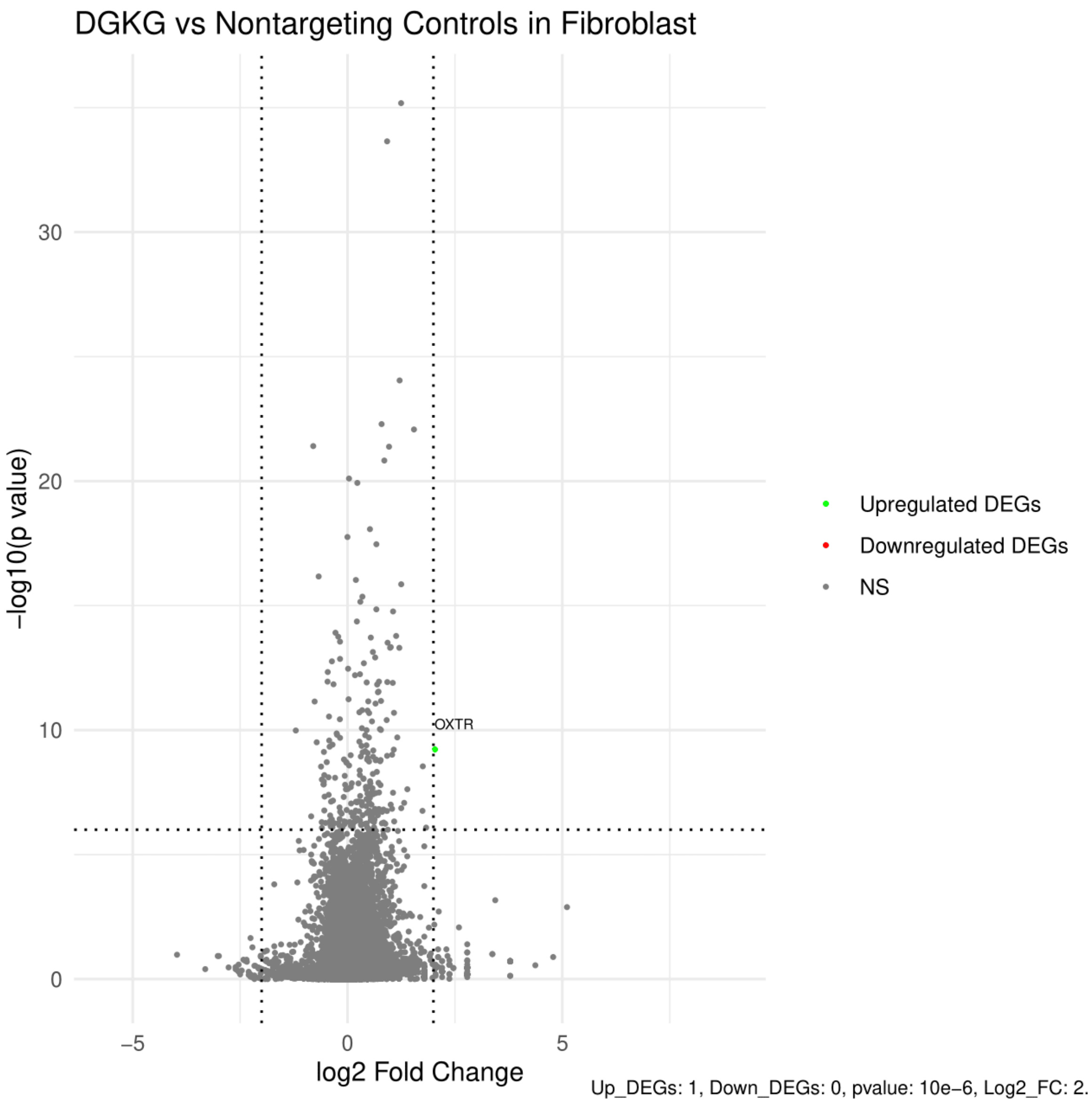
DGKG vs Nontargeting Controls in Fibroblast
30
20
10
0
−log10(p value)
OXTR
−5
0
5
log2 Fold Change
Upregulated DEGs
Downregulated DEGs
NS
Up_DEGs: 1, Down_DEGs: 0, pvalue: 10e−6, Log2_FC: 2.

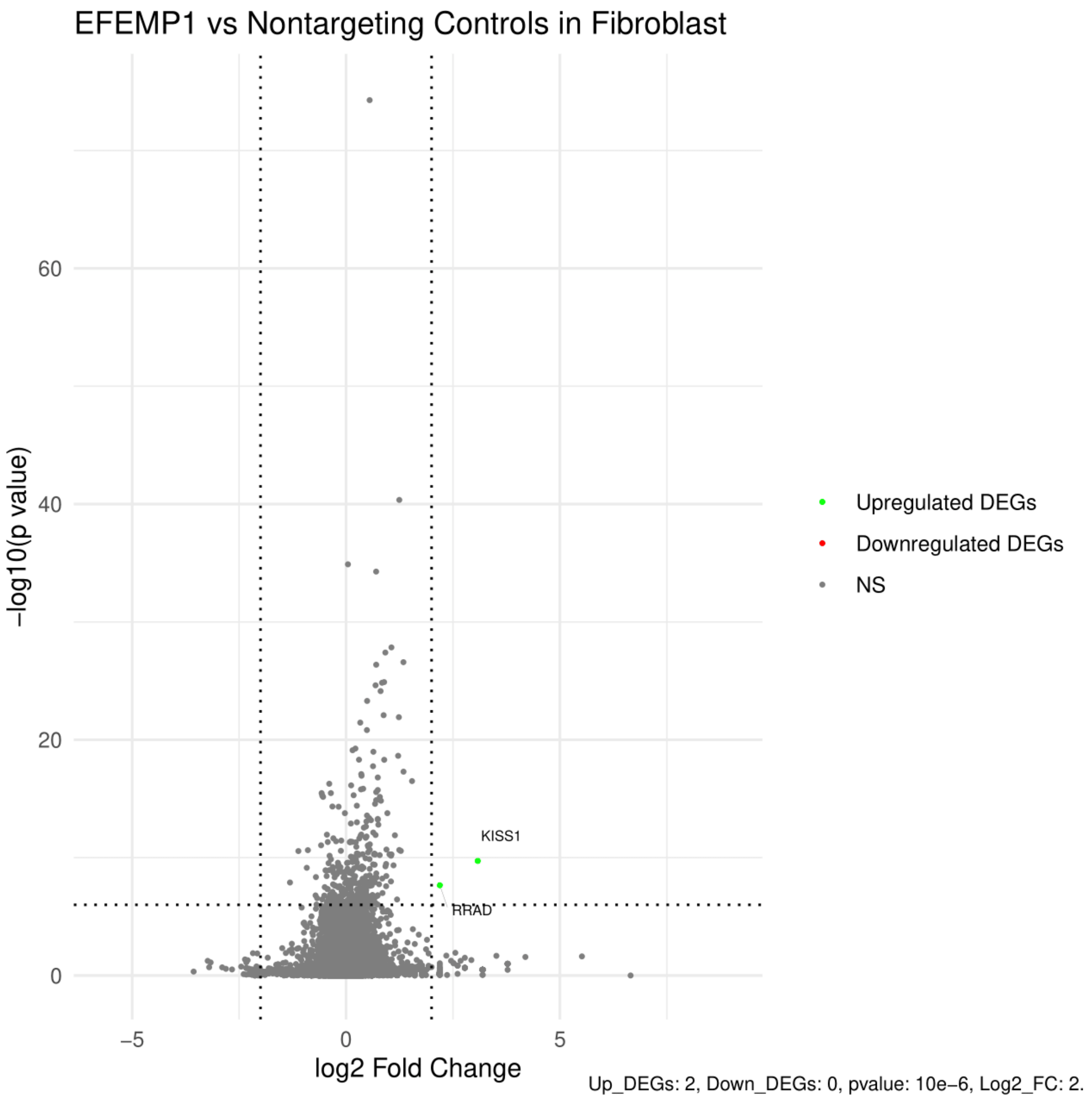
EFEMP1 vs Nontargeting Controls in Fibroblast
−log10(p value)
60
40
20
0
KISS1
RRAD
−5
0
5
log2 Fold Change
Upregulated DEGs
Downregulated DEGs
NS
Up_DEGs: 2, Down_DEGs: 0, pvalue: 10e−6, Log2_FC: 2.

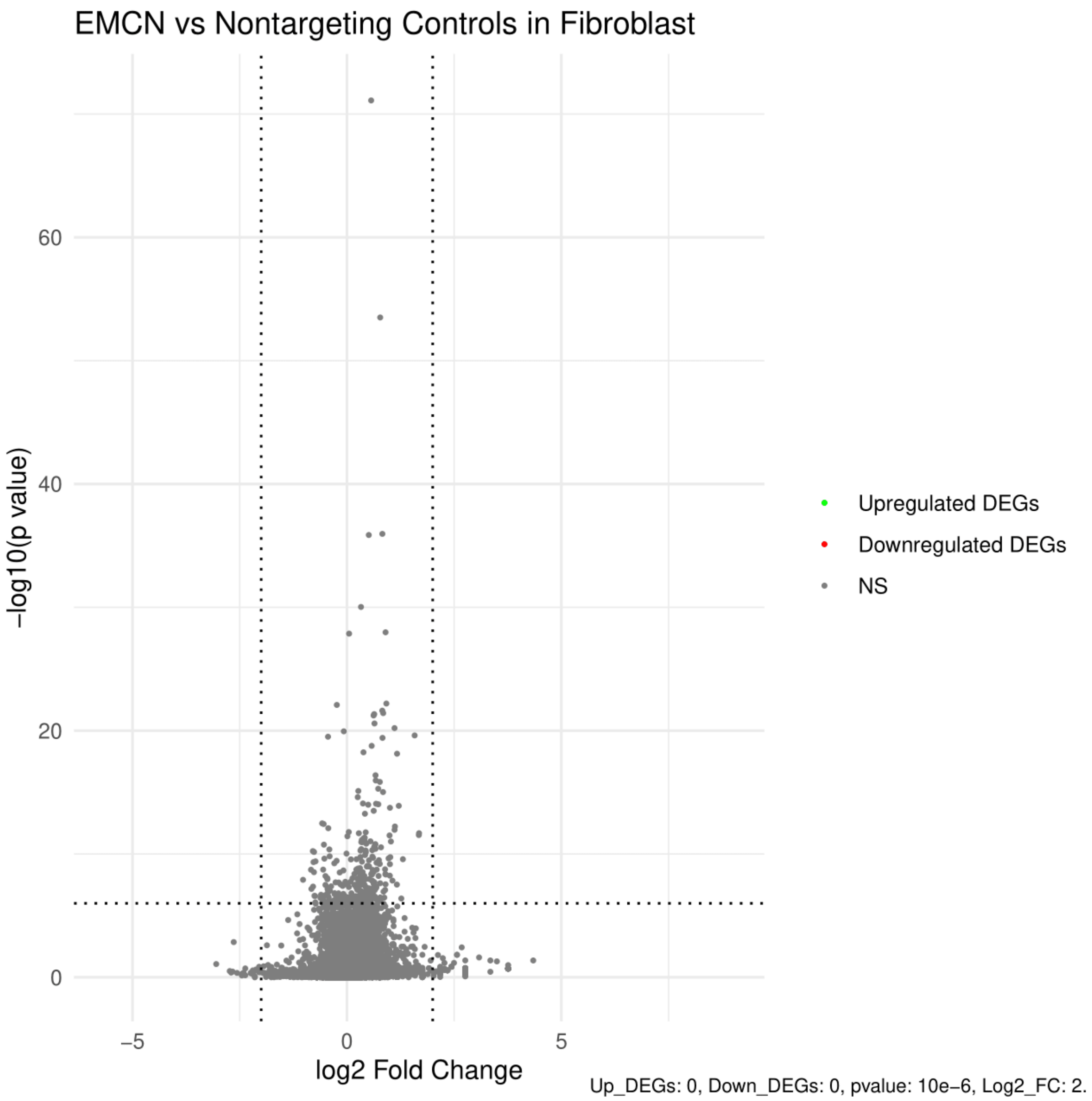
EMCN vs Nontargeting Controls in Fibroblast
60
40
20
0
−log10(p value)
−5
0
5
log2 Fold Change
Upregulated DEGs
Downregulated DEGs
NS
Up_DEGs: 0, Down_DEGs: 0, pvalue: 10e−6, Log2_FC: 2.

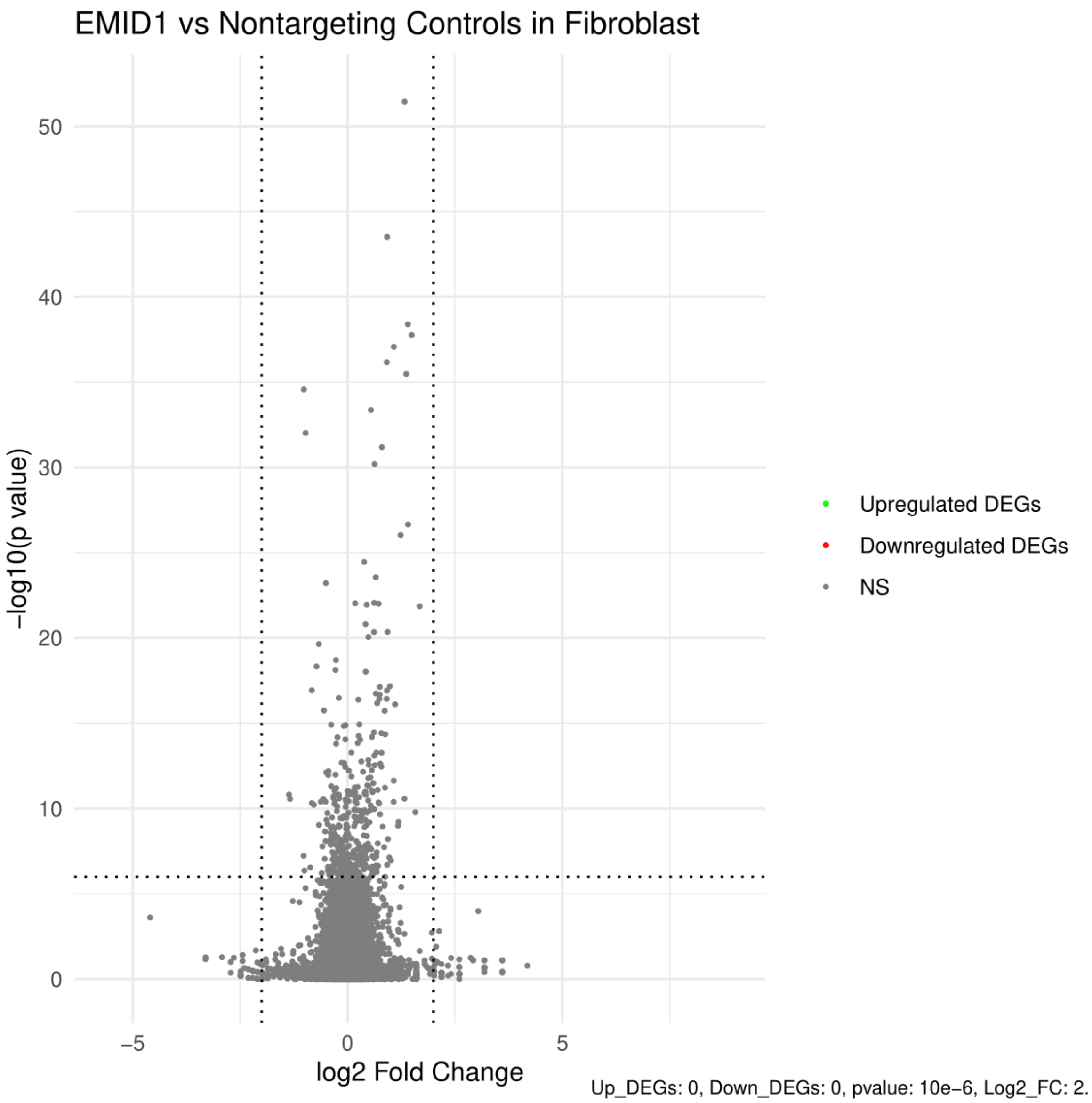
EMID1 vs Nontargeting Controls in Fibroblast
50
40
30
20
10
0
−log10(p value)
−5
0
5
log2 Fold Change
Upregulated DEGs
Downregulated DEGs
NS
Up_DEGs: 0, Down_DEGs: 0, pvalue: 10e−6, Log2_FC: 2.

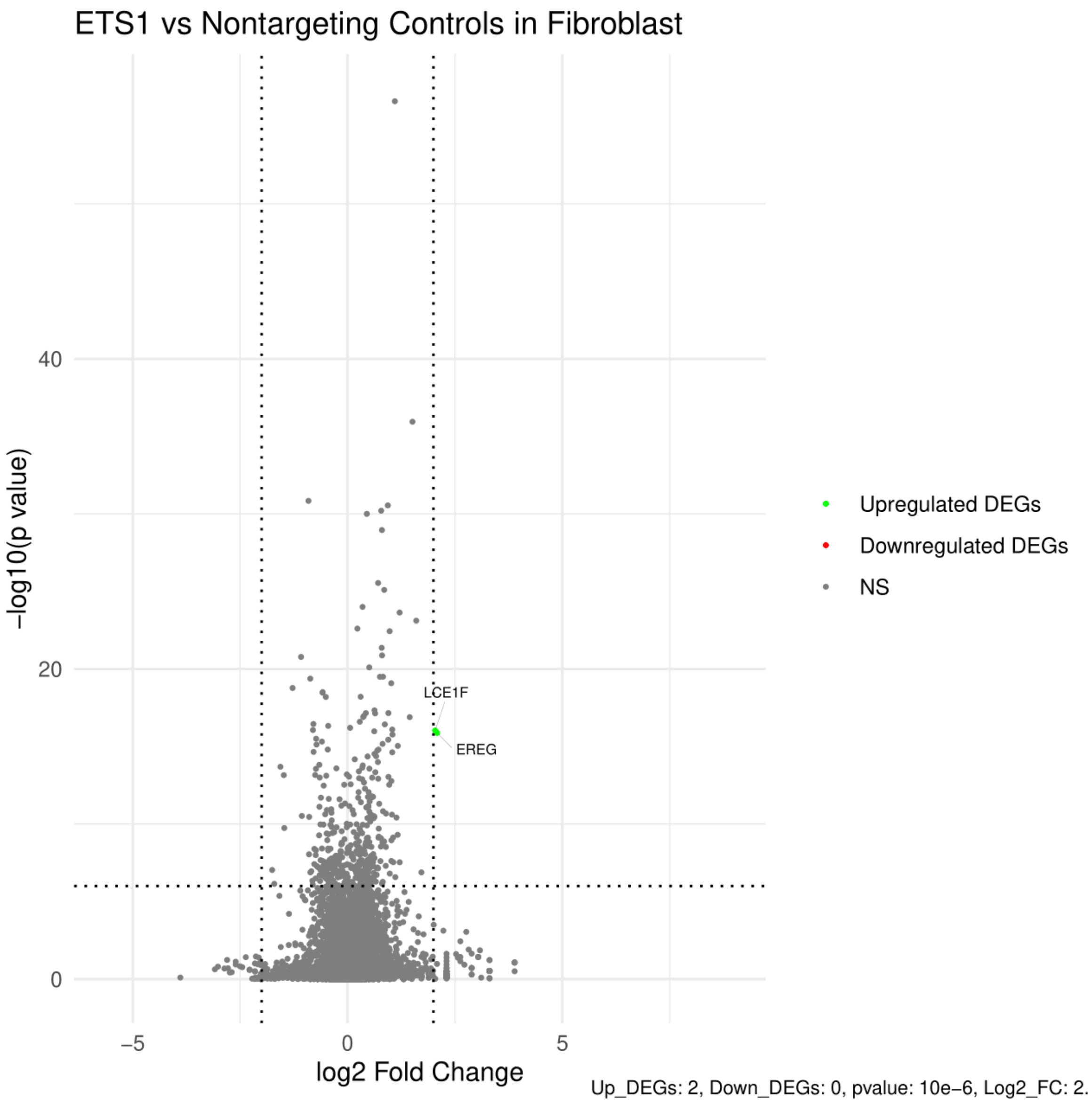

ETS1 vs Nontargeting Controls in Fibroblast
−log10(p value)
40
20
0
LCE1F
EREG
−5
0
5
log2 Fold Change
Upregulated DEGs
Downregulated DEGs
NS
Up_DEGs: 2, Down_DEGs: 0, pvalue: 10e−6, Log2_FC: 2.

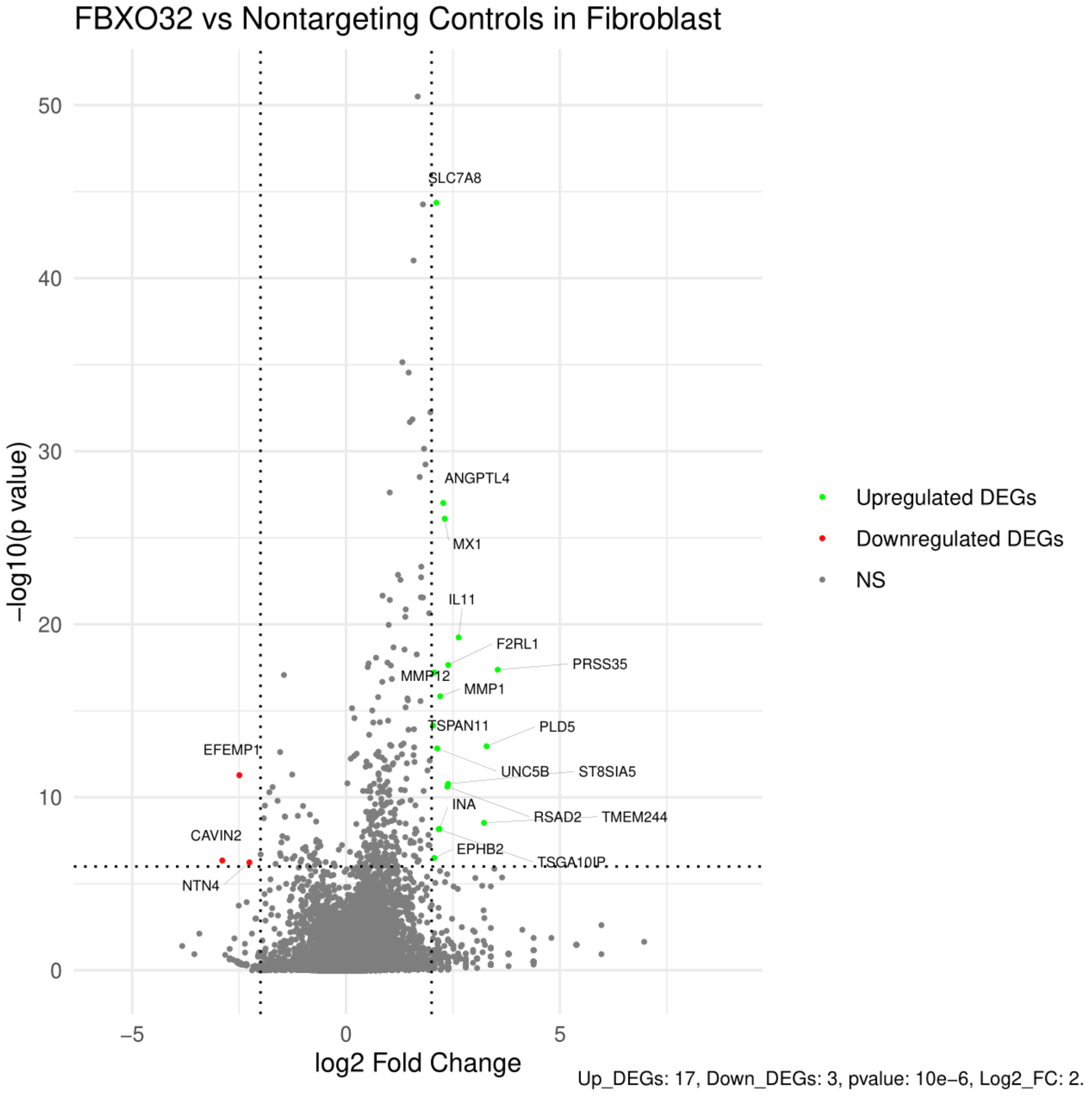
FBXO32 vs Nontargeting Controls in Fibroblast
−log10(p value)
log2 Fold Change
50
40
30
20
10
0
−5
0
5
SLC7A8
ANGPTL4
MX1
IL11
F2RL1
PRSS35
MMP12
MMP1
TSPAN11
PLD5
EFEMP1
UNC5B
ST8SIA5
INA
RSAD2
TMEM244
CAVIN2
EPHB2
TSGA10IP
NTN4
Upregulated DEGs
Downregulated DEGs
NS
Up_DEGs: 17, Down_DEGs: 3, pvalue: 10e−6, Log2_FC: 2.

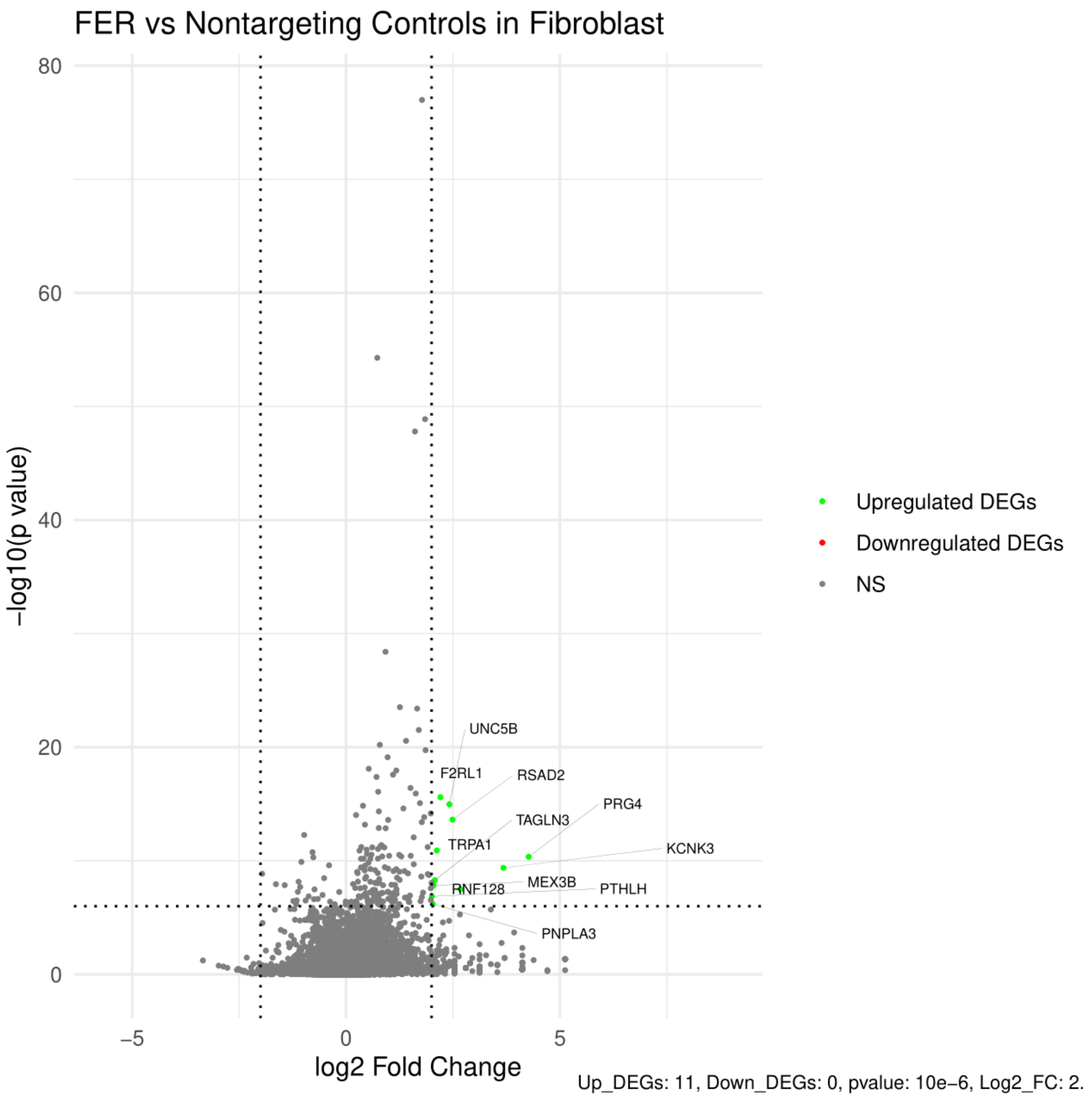

FER vs Nontargeting Controls in Fibroblast
80
60
40
20
0
–log10(p value)
–5
0
5
log2 Fold Change
UNC5B
F2RL1
RSAD2
PRG4
TAGLN3
TRPA1
KCNK3
MEX3B
RNF128
PTHLH
PNPLA3
Upregulated DEGs
Downregulated DEGs
NS
Up_DEGs: 11, Down_DEGs: 0, pvalue: 10e−6, Log2_FC: 2.

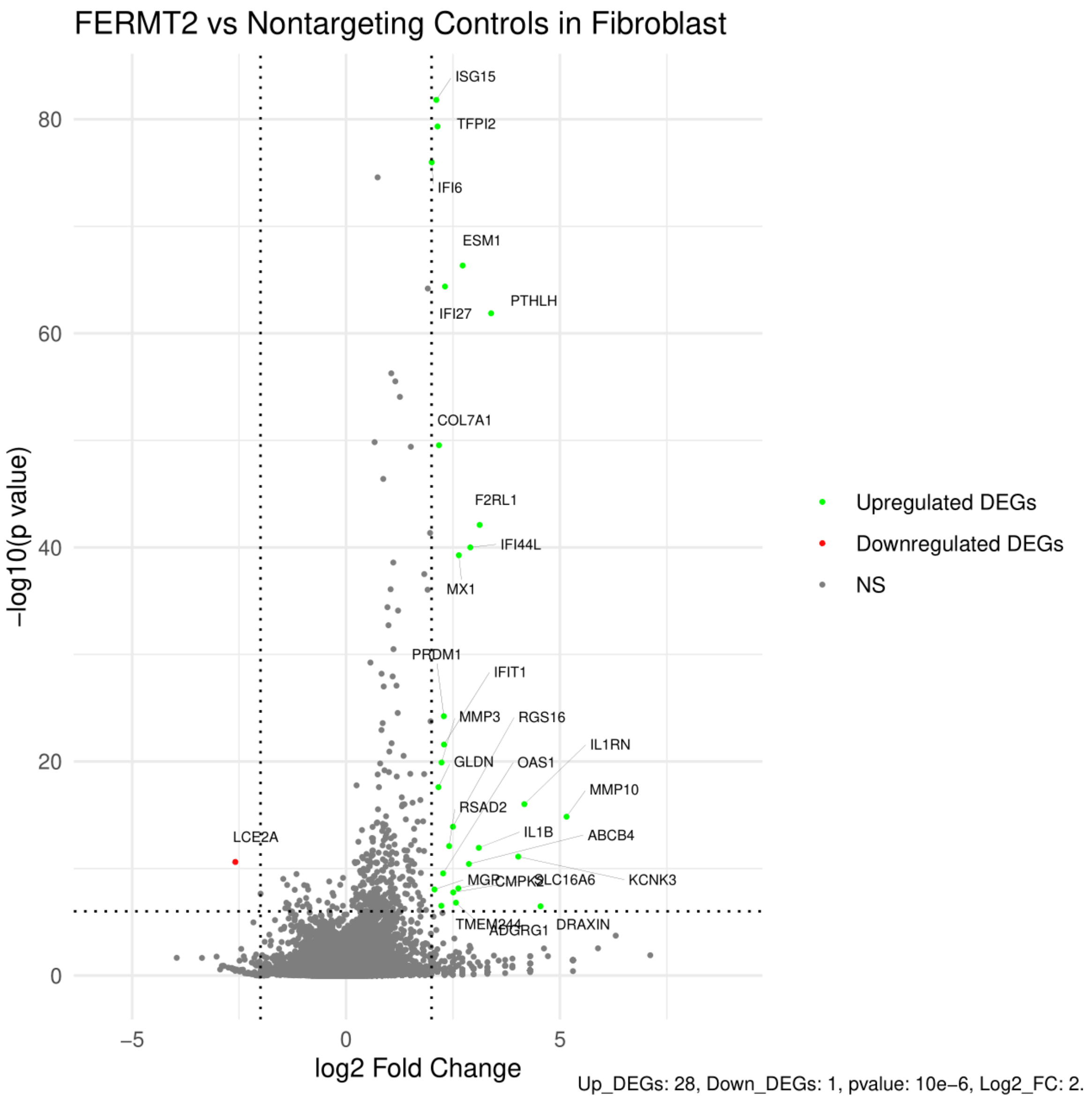

FERMT2 vs Nontargeting Controls in Fibroblast
−log10(p value)
log2 Fold Change
Upregulated DEGs
Downregulated DEGs
NS
ISG15
TFPI2
IFI6
ESM1
IFI27
PTHLH
COL7A1
F2RL1
IFI44L
MX1
PRDM1
IFIT1
MMP3
RGS16
IL1RN
GLDN
OAS1
MMP10
RSAD2
IL1B
ABCB4
LCE2A
MGP
CMPK2
SLC16A6
KCNK3
TMEM244
ADGRG1
DRAXIN
Up_DEGs: 28, Down_DEGs: 1, pvalue: 10e−6, Log2_FC: 2.

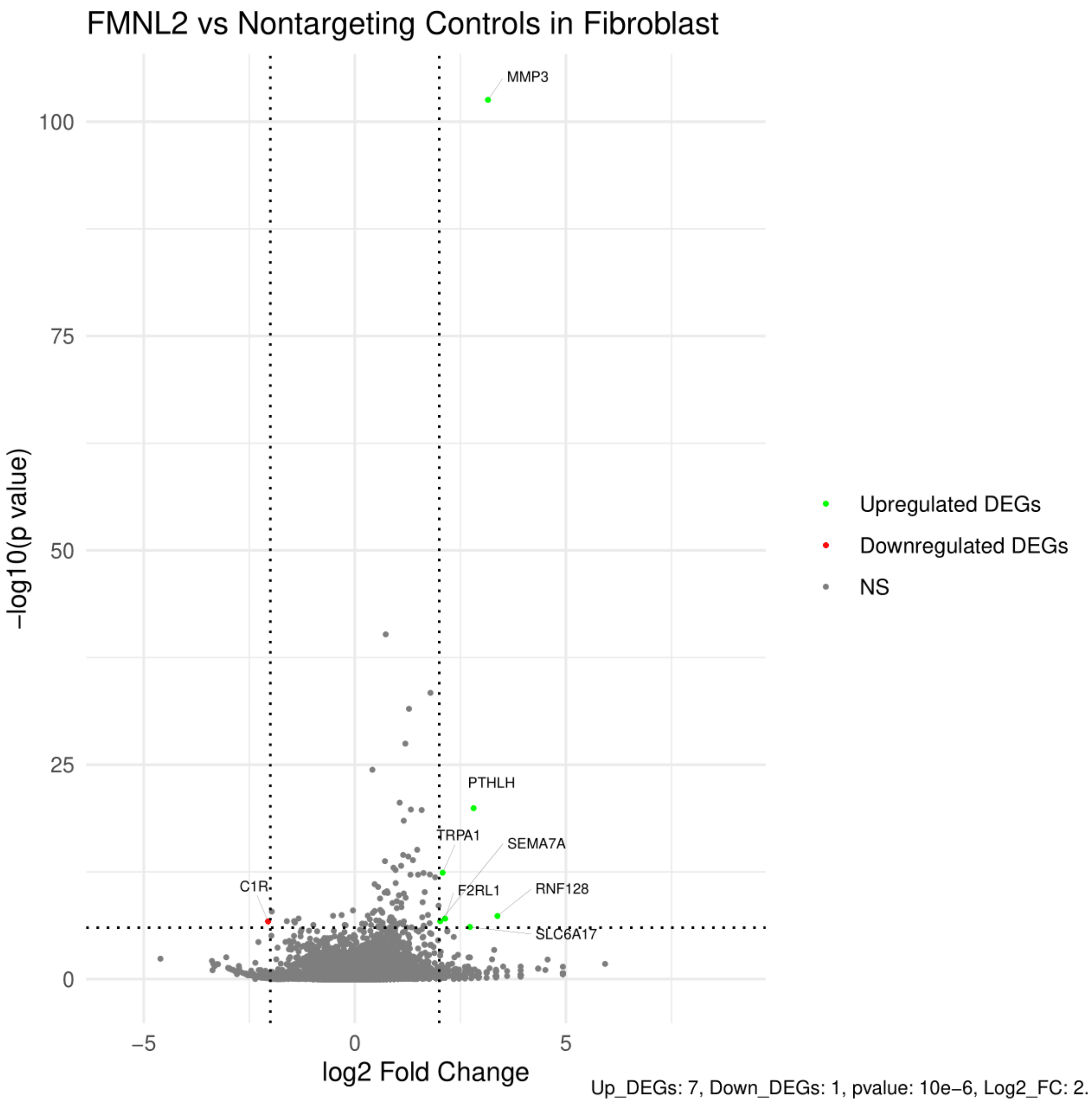
FMNL2 vs Nontargeting Controls in Fibroblast
MMP3
PTHLH
TRPA1
SEMA7A
C1R
F2RL1
RNF128
SLC6A17
100
75
50
25
0
−log10(p value)
−5
0
5
log2 Fold Change
Upregulated DEGs
Downregulated DEGs
NS
Up_DEGs: 7, Down_DEGs: 1, pvalue: 10e−6, Log2_FC: 2.

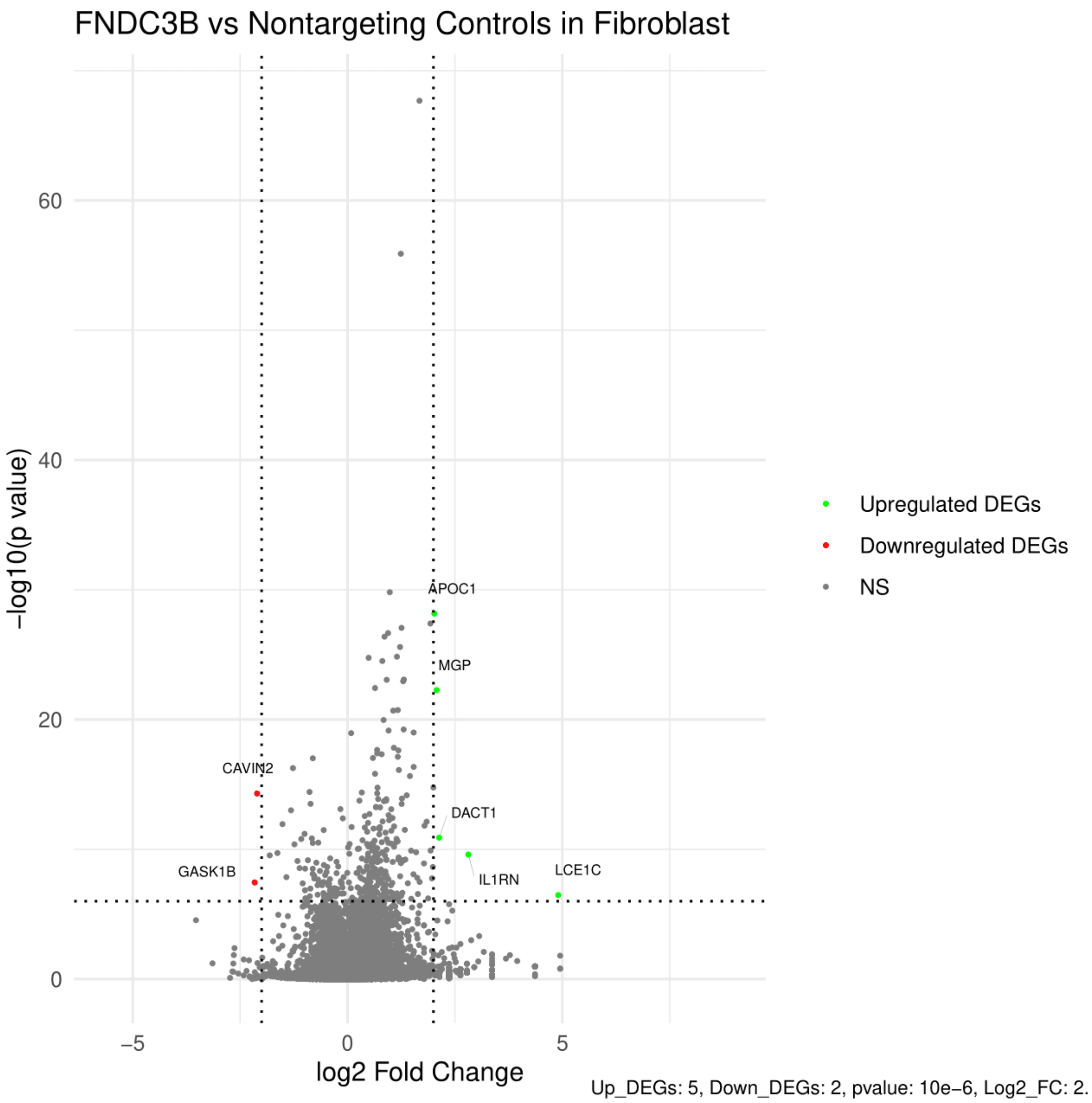

FNDC3B vs Nontargeting Controls in Fibroblast
-log10(p value)
60
40
20
0
-5
0
5
log2 Fold Change
APOC1
MGP
CAVIN2
DACT1
GASK1B
IL1RN
LCE1C
Upregulated DEGs
Downregulated DEGs
NS
Up_DEGs: 5, Down_DEGs: 2, pvalue: 10e-6, Log2_FC: 2.

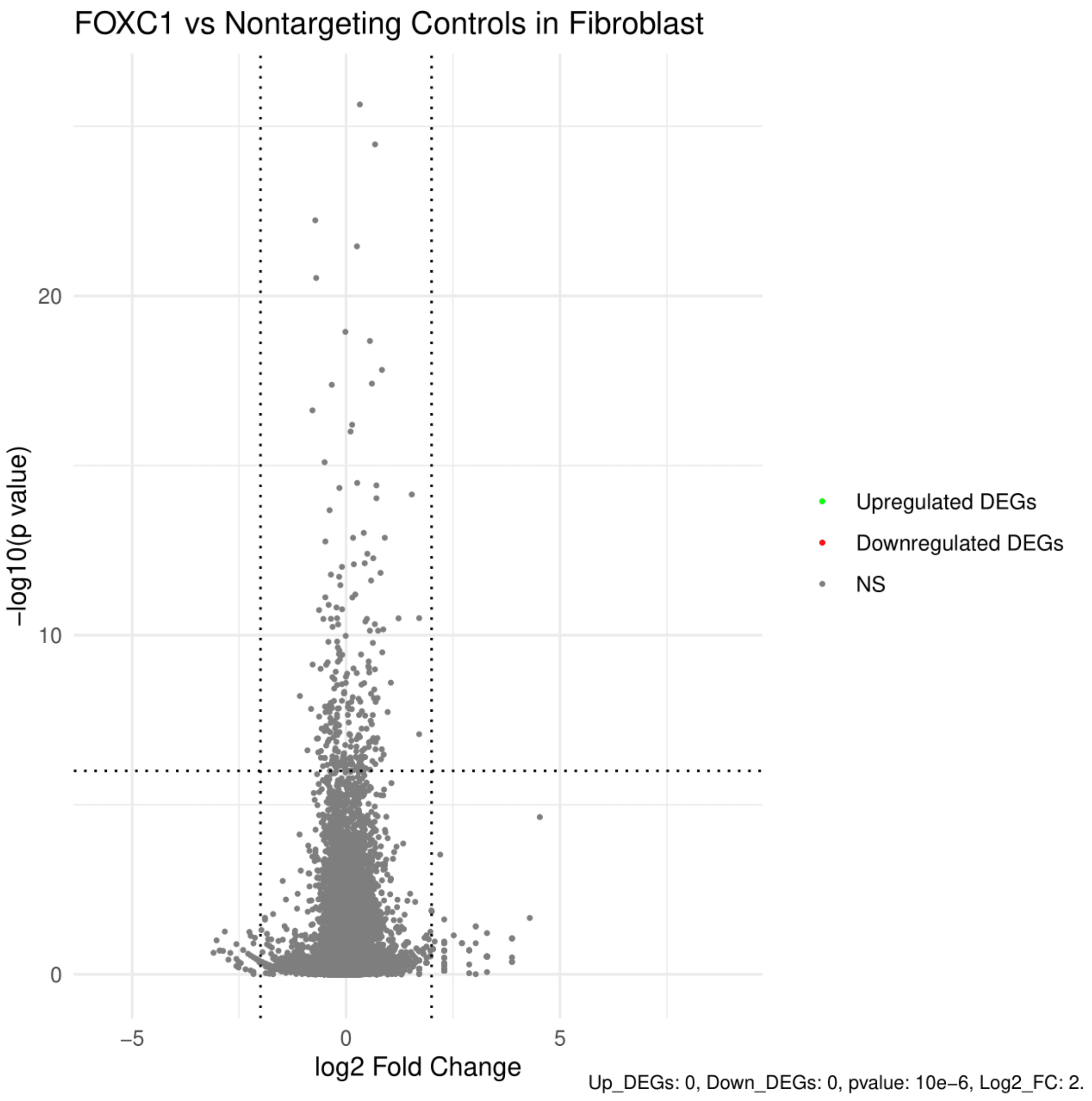
FOXC1 vs Nontargeting Controls in Fibroblast
−log10(p value)
20
10
0
−5
0
5
log2 Fold Change
Upregulated DEGs
Downregulated DEGs
NS
Up_DEGs: 0, Down_DEGs: 0, pvalue: 10e−6, Log2_FC: 2.

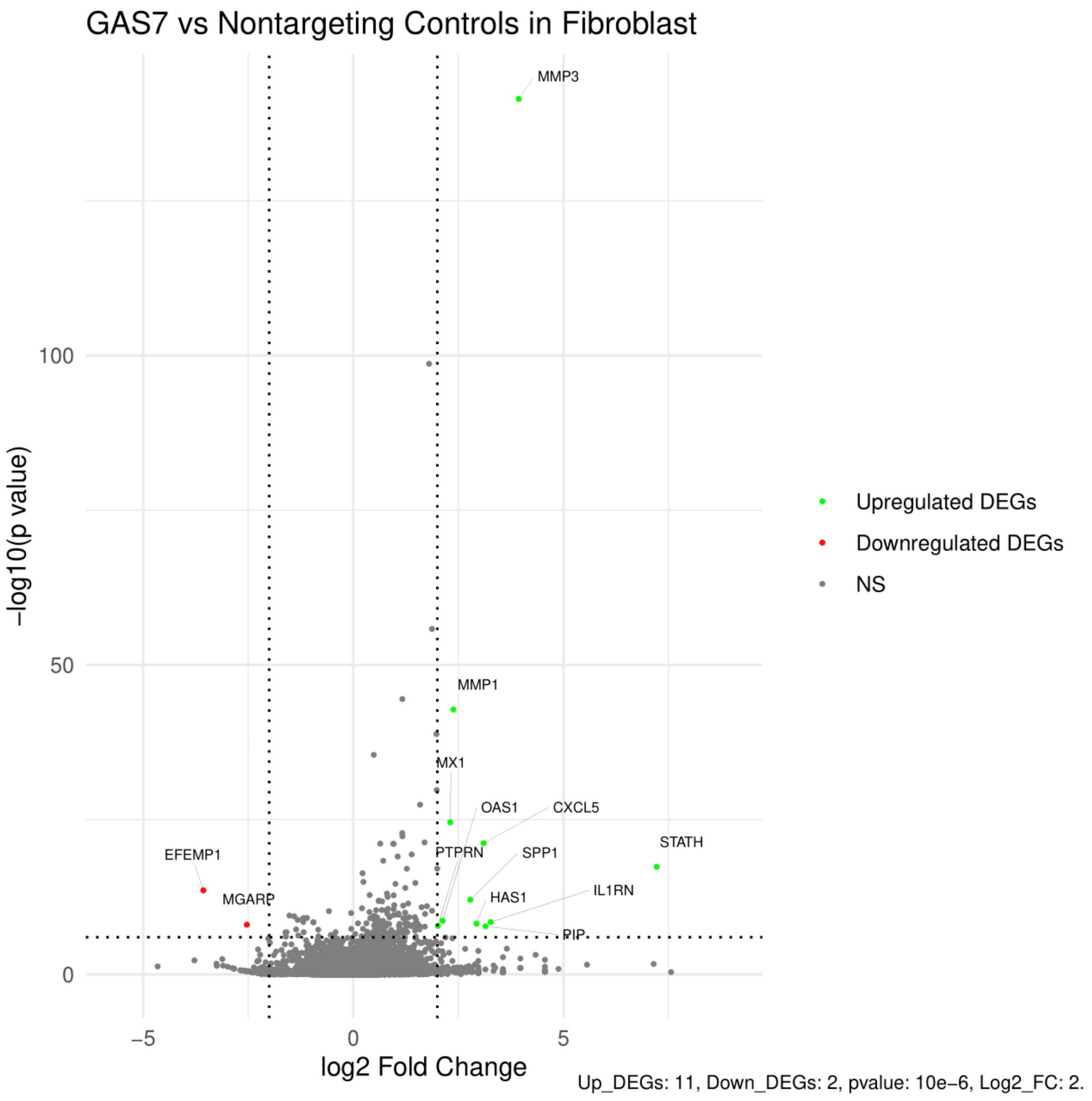
GAS7 vs Nontargeting Controls in Fibroblast
MMP3
MMP1
MX1
OAS1
CXCL5
STATH
EFEMP1
PTPRN
SPP1
MGARP
HAS1
IL1RN
PIP
100
50
0
−5
0
5
−log10(p value)
log2 Fold Change
Upregulated DEGs
Downregulated DEGs
NS
Up_DEGs: 11, Down_DEGs: 2, pvalue: 10e−6, Log2_FC: 2.

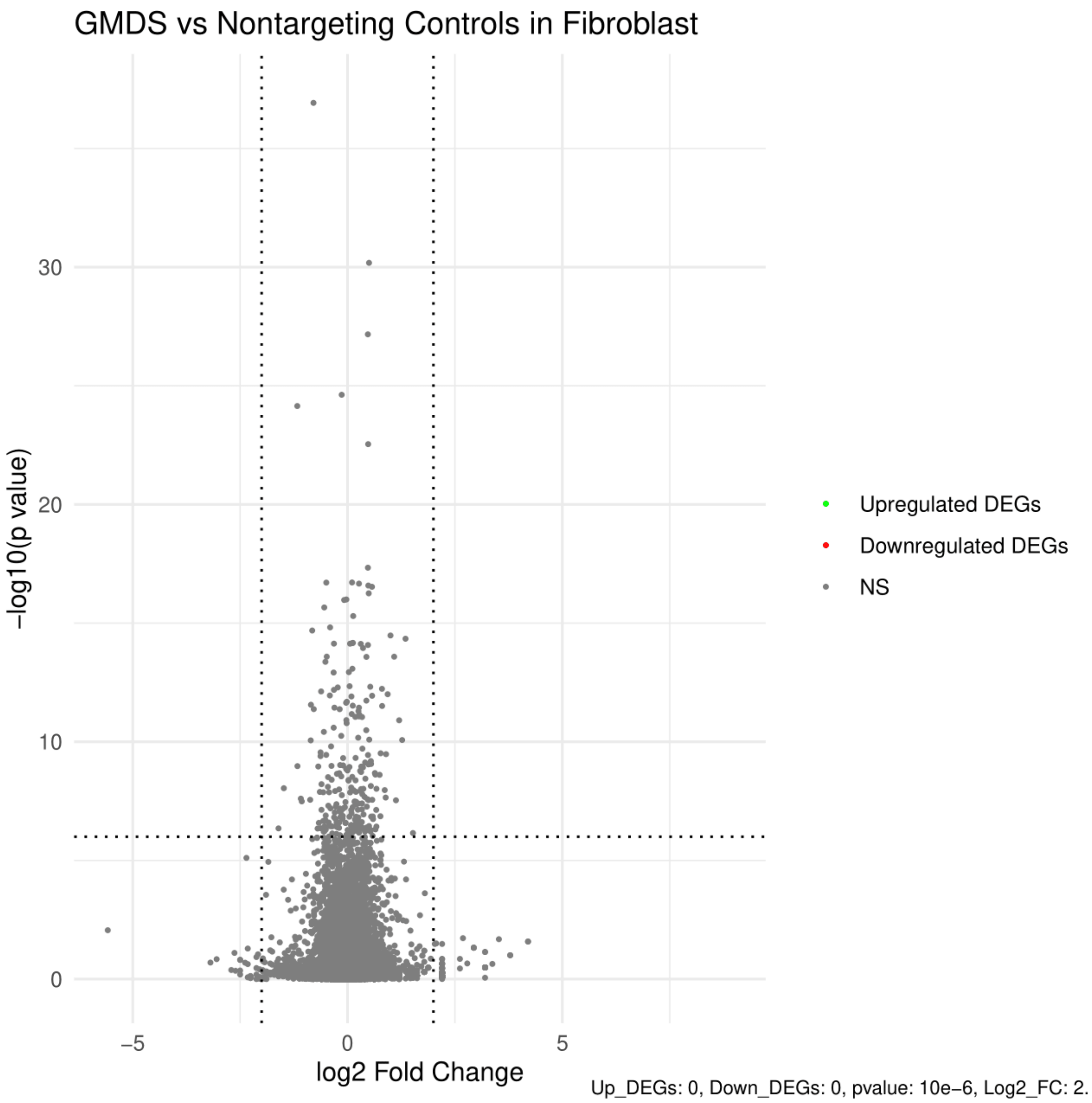
GMDS vs Nontargeting Controls in Fibroblast
30
20
10
0
−log10(p value)
−5
0
5
log2 Fold Change
Upregulated DEGs
Downregulated DEGs
NS
Up_DEGs: 0, Down_DEGs: 0, pvalue: 10e−6, Log2_FC: 2.

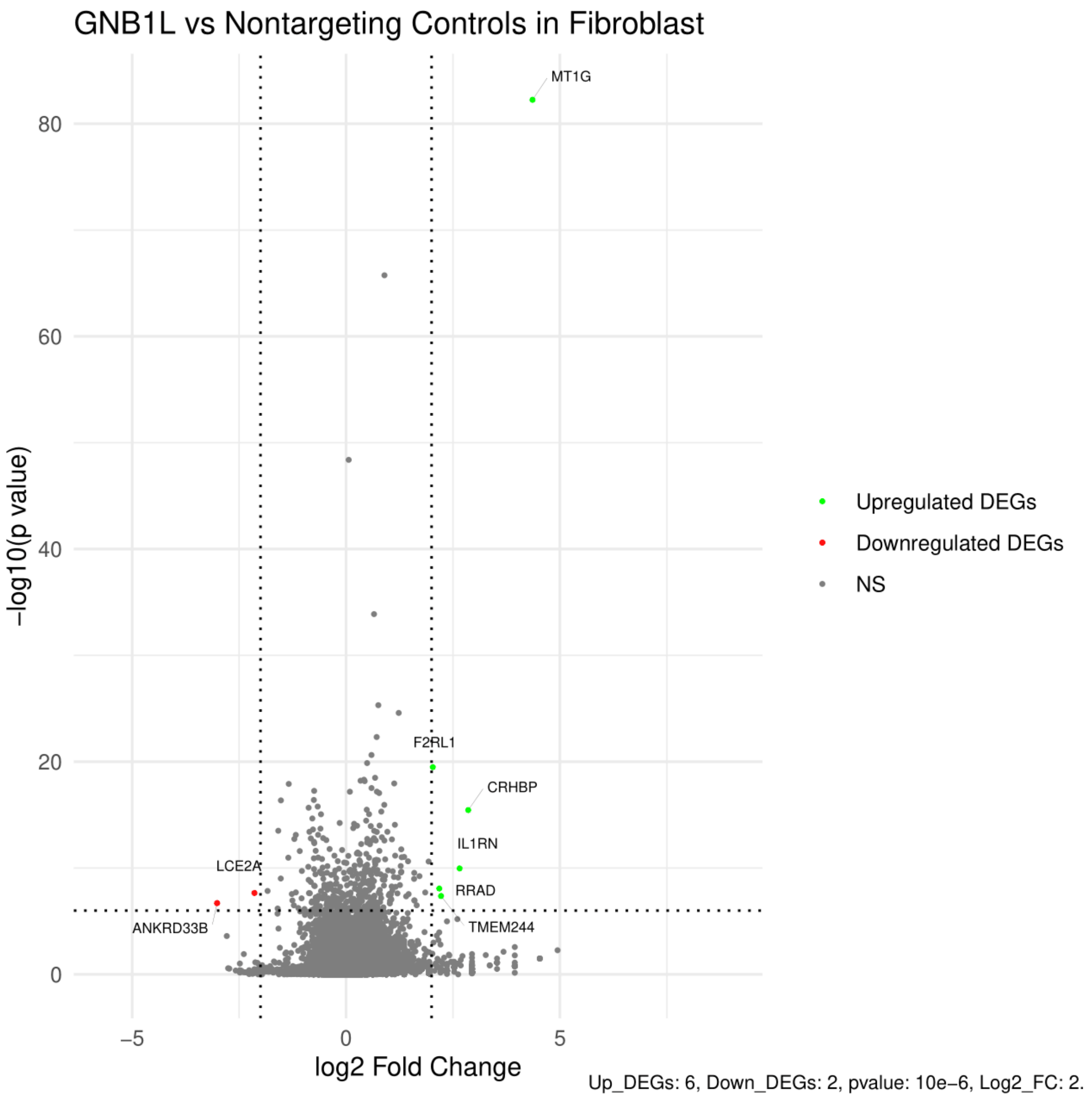

GNB1L vs Nontargeting Controls in Fibroblast
−log10(p value)
80
60
40
20
0
−5
0
5
log2 Fold Change
MT1G
F2RL1
CRHBP
IL1RN
LCE2A
RRAD
ANKRD33B
TMEM244
Upregulated DEGs
Downregulated DEGs
NS
Up_DEGs: 6, Down_DEGs: 2, pvalue: 10e−6, Log2_FC: 2.

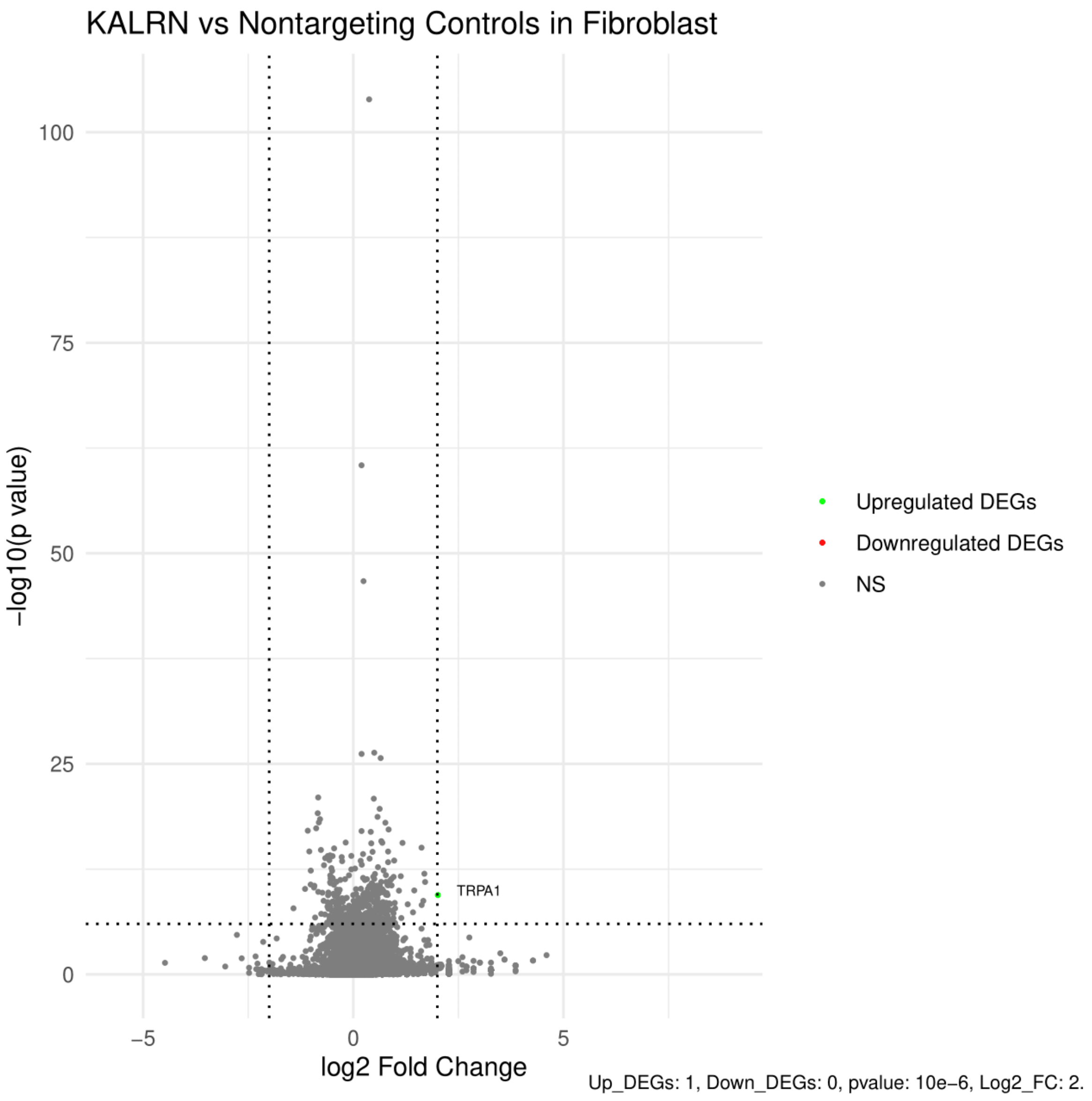

KALRN vs Nontargeting Controls in Fibroblast
100
75
50
25
0
−log10(p value)
TRPA1
−5
0
5
log2 Fold Change
Upregulated DEGs
Downregulated DEGs
NS
Up_DEGs: 1, Down_DEGs: 0, pvalue: 10e−6, Log2_FC: 2.

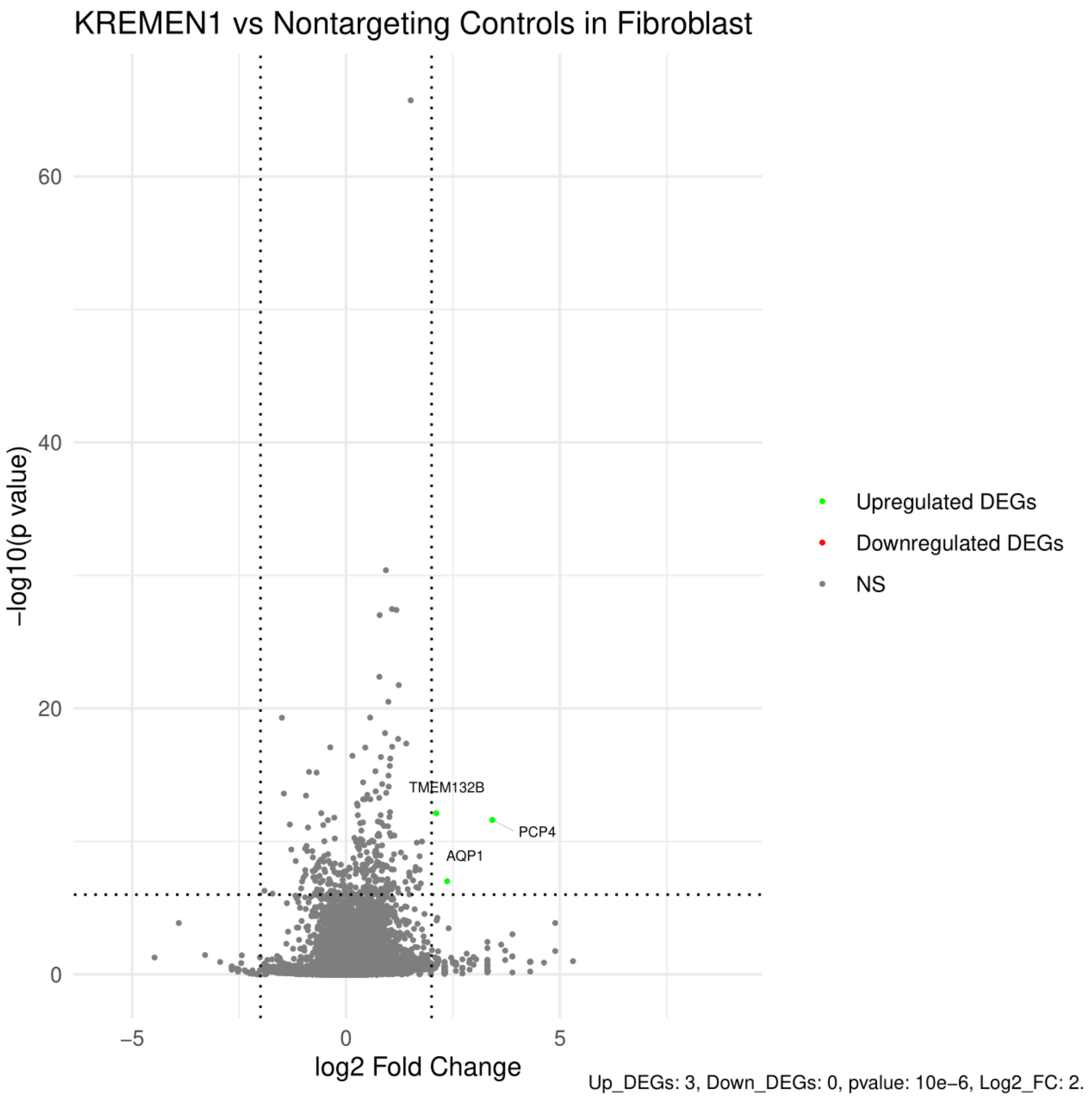

KREMEN1 vs Nontargeting Controls in Fibroblast
60
40
20
0
−log10(p value)
−5
0
5
log2 Fold Change
TMEM132B
PCP4
AQP1
Upregulated DEGs
Downregulated DEGs
NS
Up_DEGs: 3, Down_DEGs: 0, pvalue: 10e−6, Log2_FC: 2.

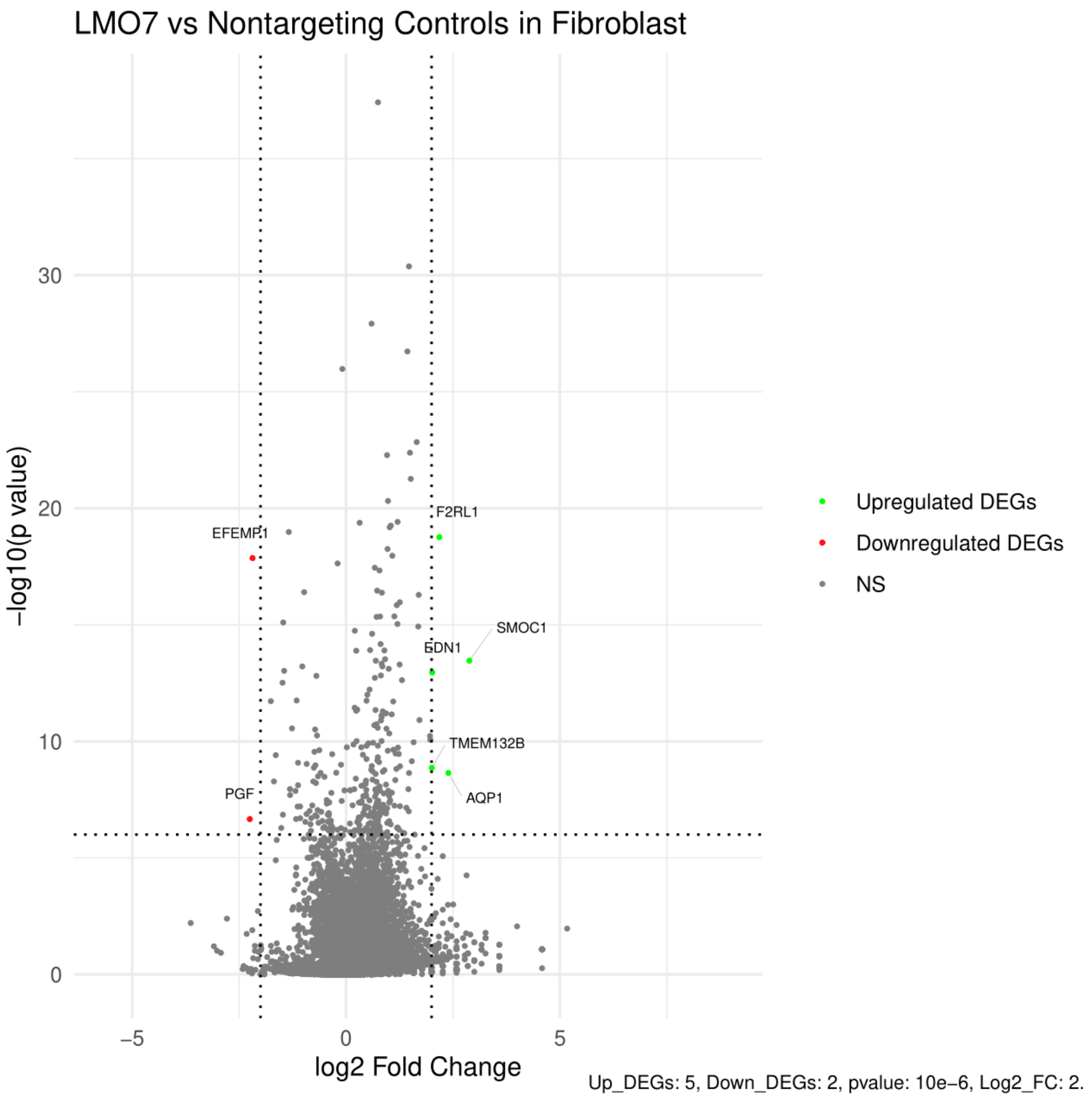

LMO7 vs Nontargeting Controls in Fibroblast
−log10(p value)
30
20
10
0
−5
0
5
log2 Fold Change
EFEMP1
PGF
F2RL1
SMOC1
EDN1
TMEM132B
AQP1
Upregulated DEGs
Downregulated DEGs
NS
Up_DEGs: 5, Down_DEGs: 2, pvalue: 10e−6, Log2_FC: 2.

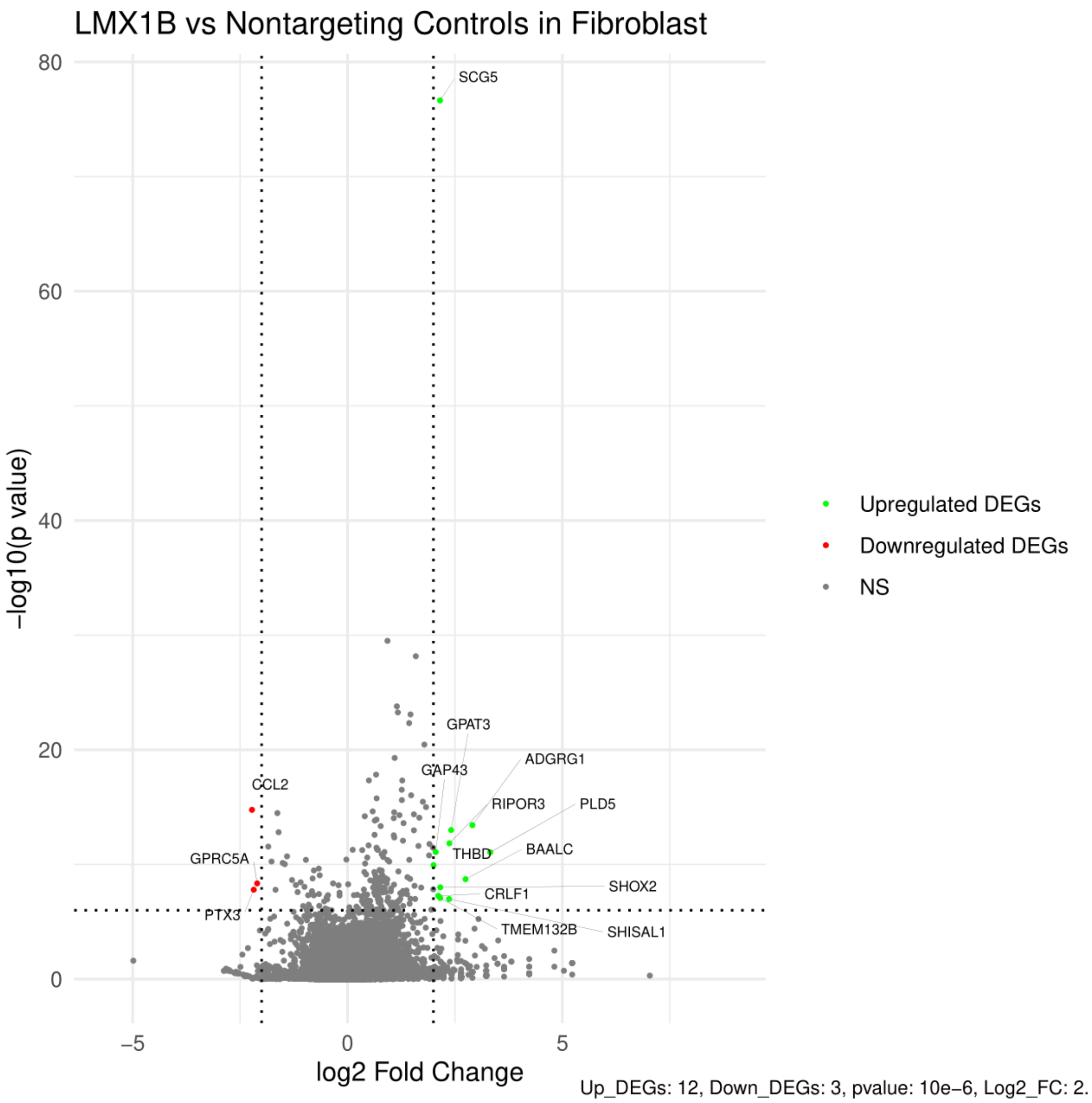
LMX1B vs Nontargeting Controls in Fibroblast
80
60
40
20
0
−log10(p value)
−5
0
5
log2 Fold Change
SCG5
GPAT3
ADGRG1
GAP43
CCL2
RIPOR3
PLD5
THBD
BAALC
GPRC5A
SHOX2
CRLF1
PTX3
TMEM132B
SHISAL1
Upregulated DEGs
Downregulated DEGs
NS
Up_DEGs: 12, Down_DEGs: 3, pvalue: 10e−6, Log2_FC: 2.

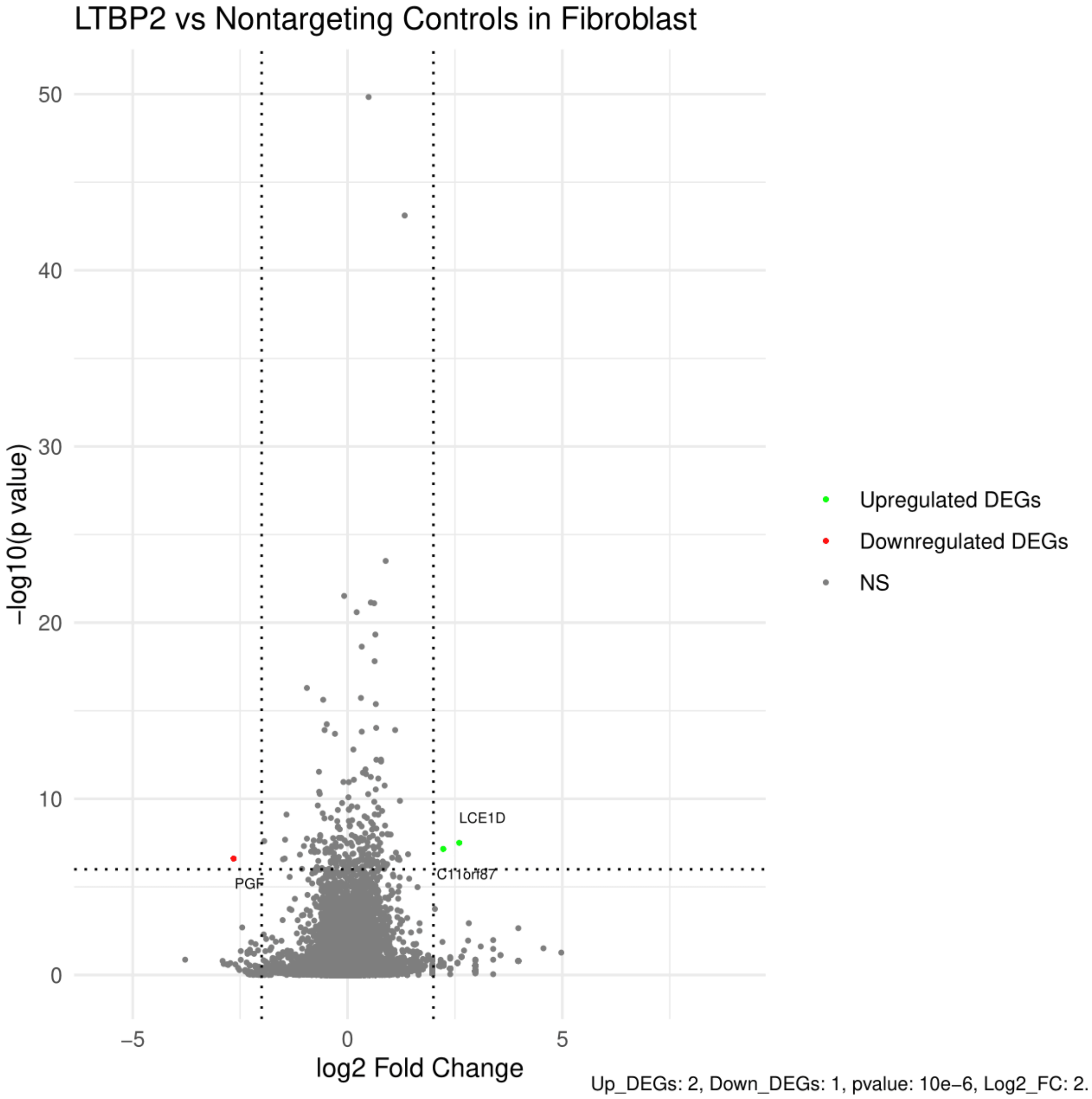
LTBP2 vs Nontargeting Controls in Fibroblast
−log10(p value)
log2 Fold Change
LCE1D
C11orf87
PGF
Upregulated DEGs
Downregulated DEGs
NS
Up_DEGs: 2, Down_DEGs: 1, pvalue: 10e−6, Log2_FC: 2.

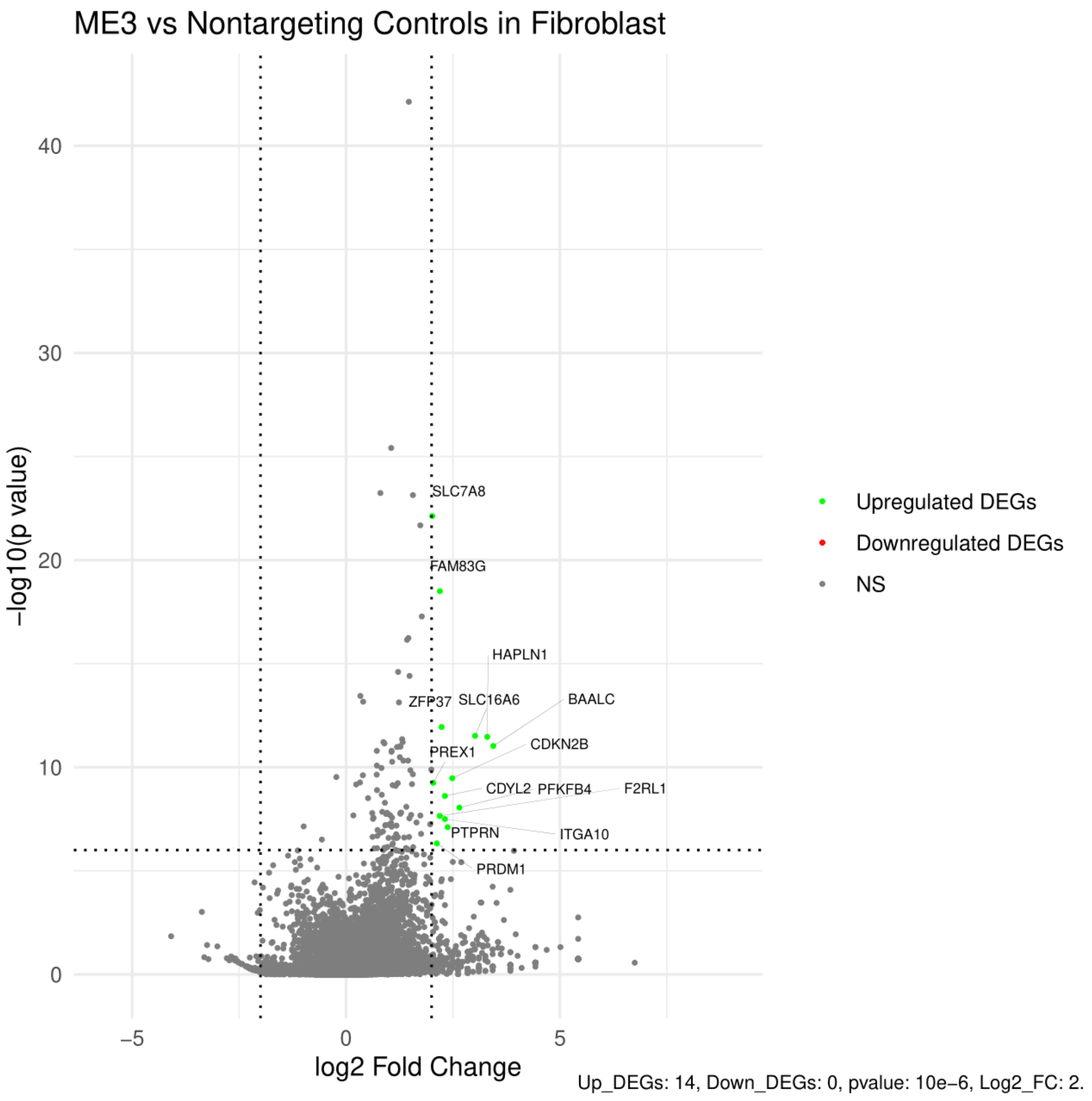
ME3 vs Nontargeting Controls in Fibroblast
−log10(p value)
log2 Fold Change
SLC7A8
FAM83G
HAPLN1
ZFP37
SLC16A6
BAALC
CDKN2B
PREX1
CDYL2
PFKFB4
F2RL1
PTPRN
ITGA10
PRDM1
Upregulated DEGs
Downregulated DEGs
NS
Up_DEGs: 14, Down_DEGs: 0, pvalue: 10e−6, Log2_FC: 2.

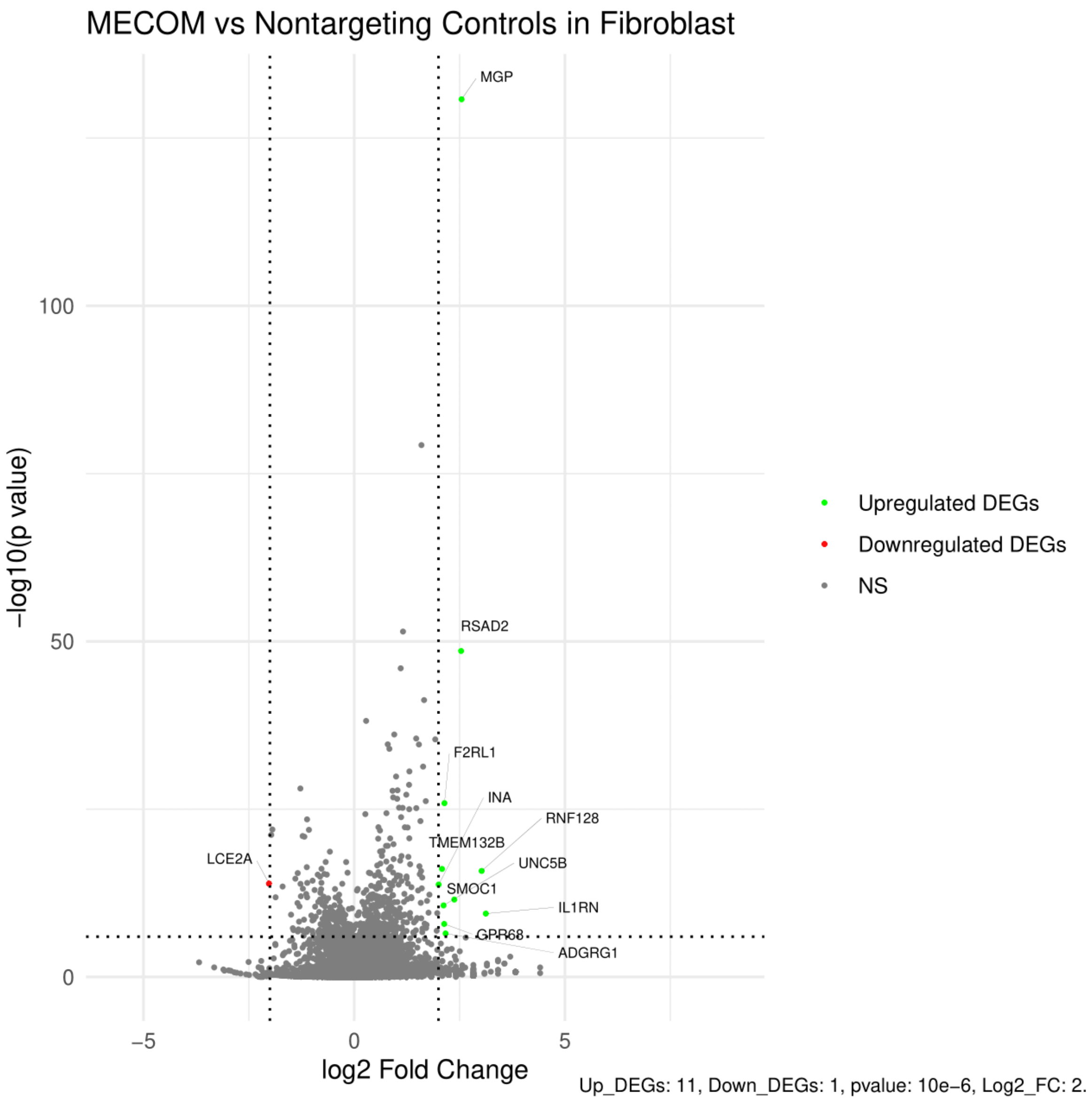
MECOM vs Nontargeting Controls in Fibroblast
MGP
RSAD2
F2RL1
INA
RNF128
TMEM132B
UNC5B
LCE2A
SMOC1
IL1RN
GPR68
ADGRG1
100
50
0
–log10(p value)
–5
0
5
log2 Fold Change
Upregulated DEGs
Downregulated DEGs
NS
Up_DEGs: 11, Down_DEGs: 1, pvalue: 10e–6, Log2_FC: 2.

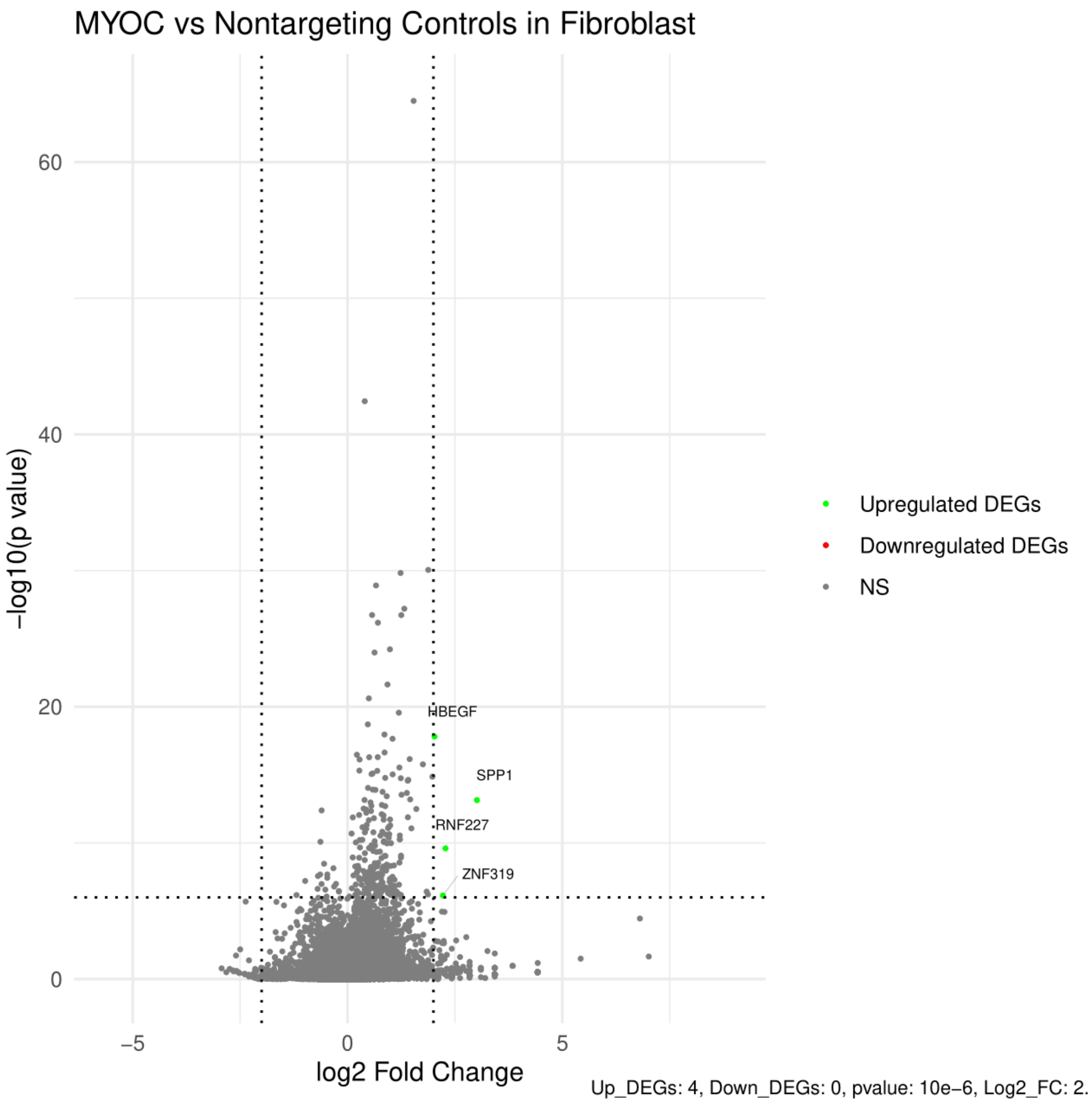

MYOC vs Nontargeting Controls in Fibroblast
60
40
20
0
−log10(p value)
HBEGF
SPP1
RNF227
ZNF319
−5
0
5
log2 Fold Change
Upregulated DEGs
Downregulated DEGs
NS
Up_DEGs: 4, Down_DEGs: 0, pvalue: 10e−6, Log2_FC: 2.

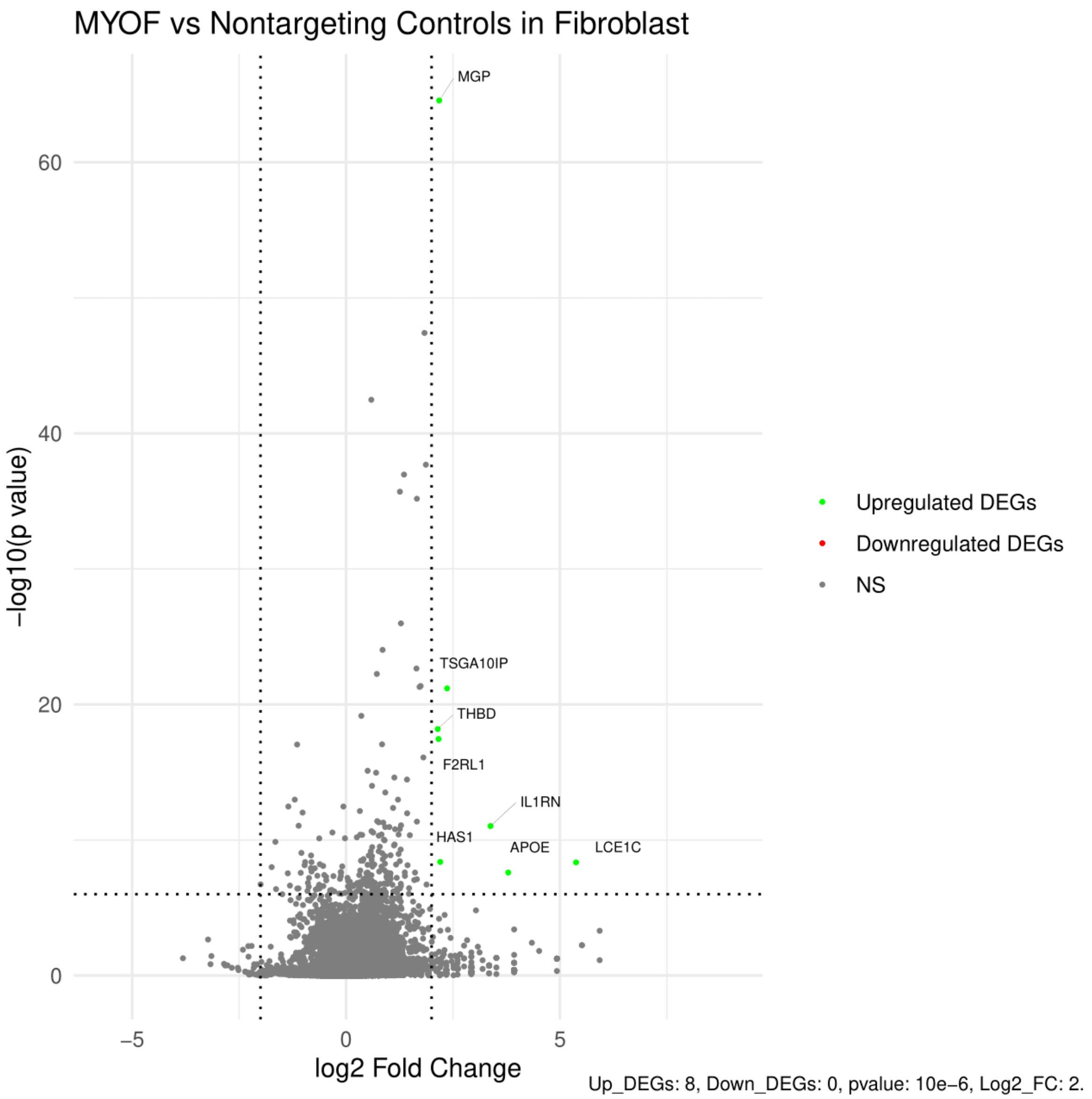
MYOF vs Nontargeting Controls in Fibroblast
MGP
TSGA10IP
THBD
F2RL1
IL1RN
HAS1
APOE
LCE1C
60
40
20
0
-log10(p value)
-5
0
5
log2 Fold Change
Upregulated DEGs
Downregulated DEGs
NS
Up_DEGs: 8, Down_DEGs: 0, pvalue: 10e-6, Log2_FC: 2.

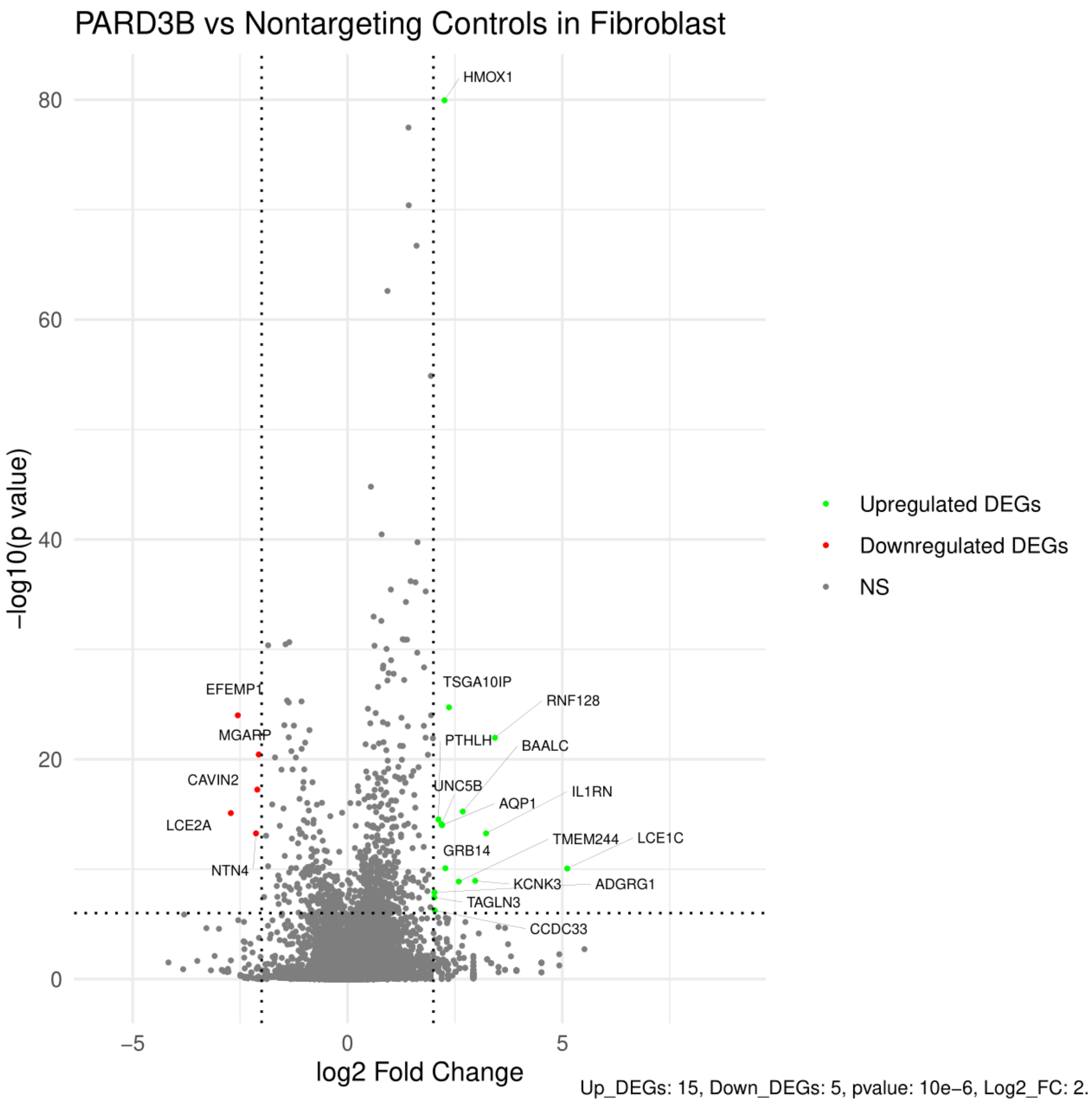

PARD3B vs Nontargeting Controls in Fibroblast
−log10(p value)
log2 Fold Change
80
60
40
20
0
−5
0
5
HMOX1
TSGA10IP
RNF128
PTHLH
BAALC
UNC5B
AQP1
IL1RN
TMEM244
LCE1C
GRB14
KCNK3
ADGRG1
TAGLN3
CCDC33
EFEMP1
MGARP
CAVIN2
LCE2A
NTN4
Upregulated DEGs
Downregulated DEGs
NS
Up_DEGs: 15, Down_DEGs: 5, pvalue: 10e−6, Log2_FC: 2.

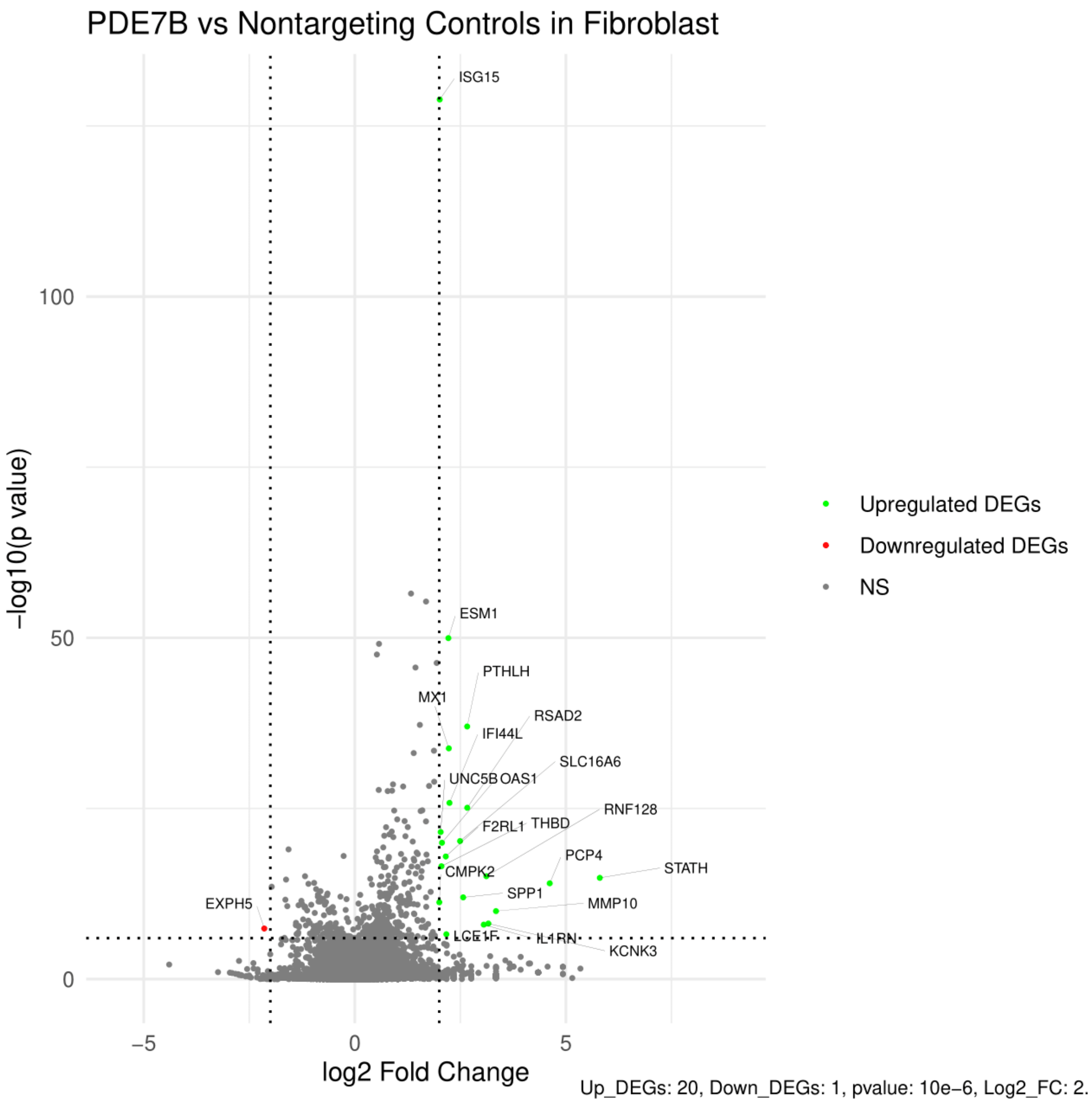
PDE7B vs Nontargeting Controls in Fibroblast
ISG15
ESM1
PTHLH
MX1
RSAD2
IFI44L
SLC16A6
UNC5B
OAS1
RNF128
F2RL1
THBD
PCP4
CMPK2
STATH
SPP1
EXPH5
MMP10
LCE1F
IL1RN
KCNK3
100
50
0
−log10(p value)
−5
0
5
log2 Fold Change
Upregulated DEGs
Downregulated DEGs
NS
Up_DEGs: 20, Down_DEGs: 1, pvalue: 10e−6, Log2_FC: 2.

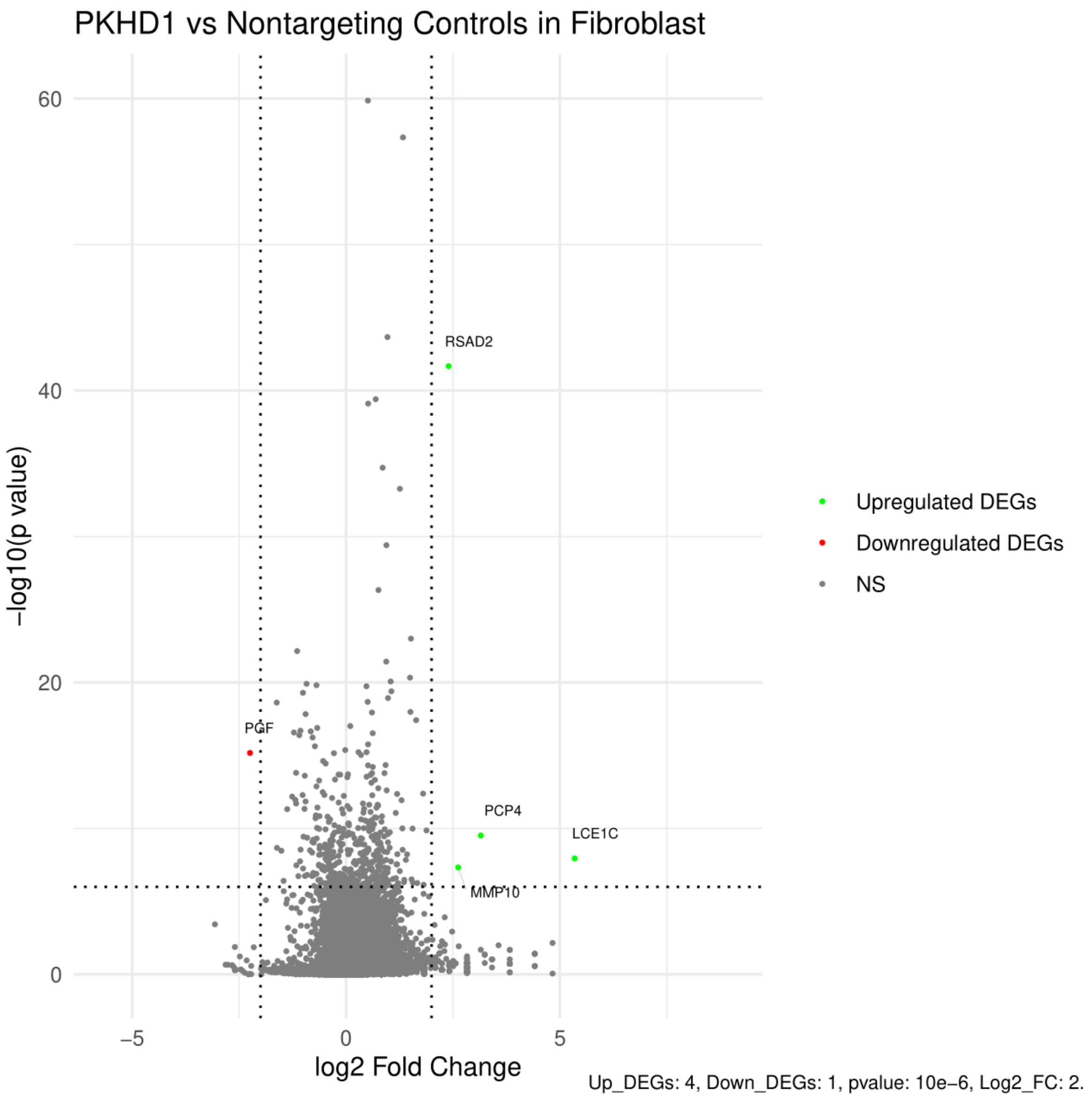
PKHD1 vs Nontargeting Controls in Fibroblast
60
40
20
0
−log10(p value)
RSAD2
PGF
PCP4
LCE1C
MMP10
−5
0
5
log2 Fold Change
Upregulated DEGs
Downregulated DEGs
NS
Up_DEGs: 4, Down_DEGs: 1, pvalue: 10e−6, Log2_FC: 2.

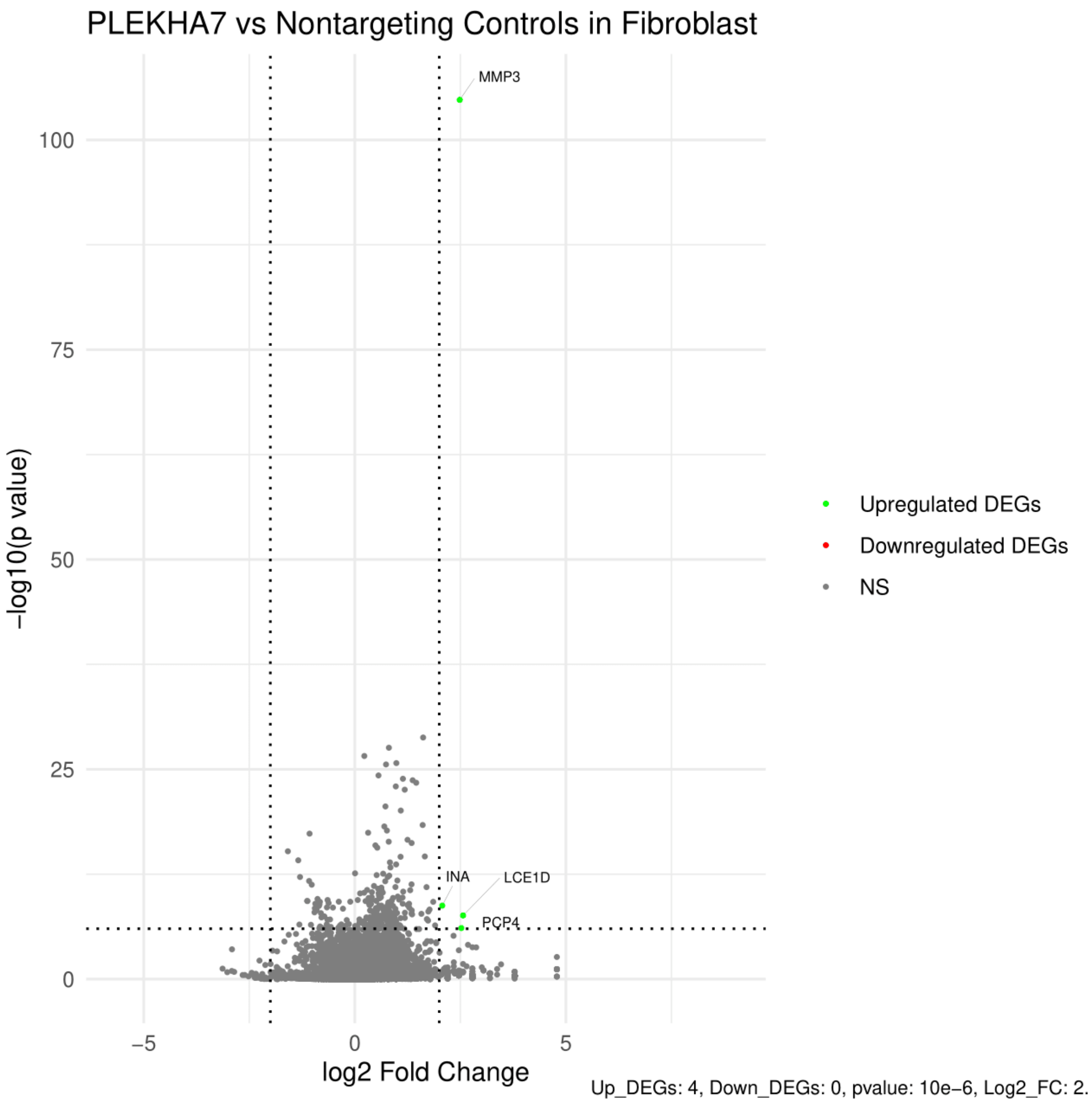
PLEKHA7 vs Nontargeting Controls in Fibroblast
MMP3
INA
LCE1D
PCP4
100
75
50
25
0
−5
0
5
−log10(p value)
log2 Fold Change
Upregulated DEGs
Downregulated DEGs
NS
Up_DEGs: 4, Down_DEGs: 0, pvalue: 10e−6, Log2_FC: 2.

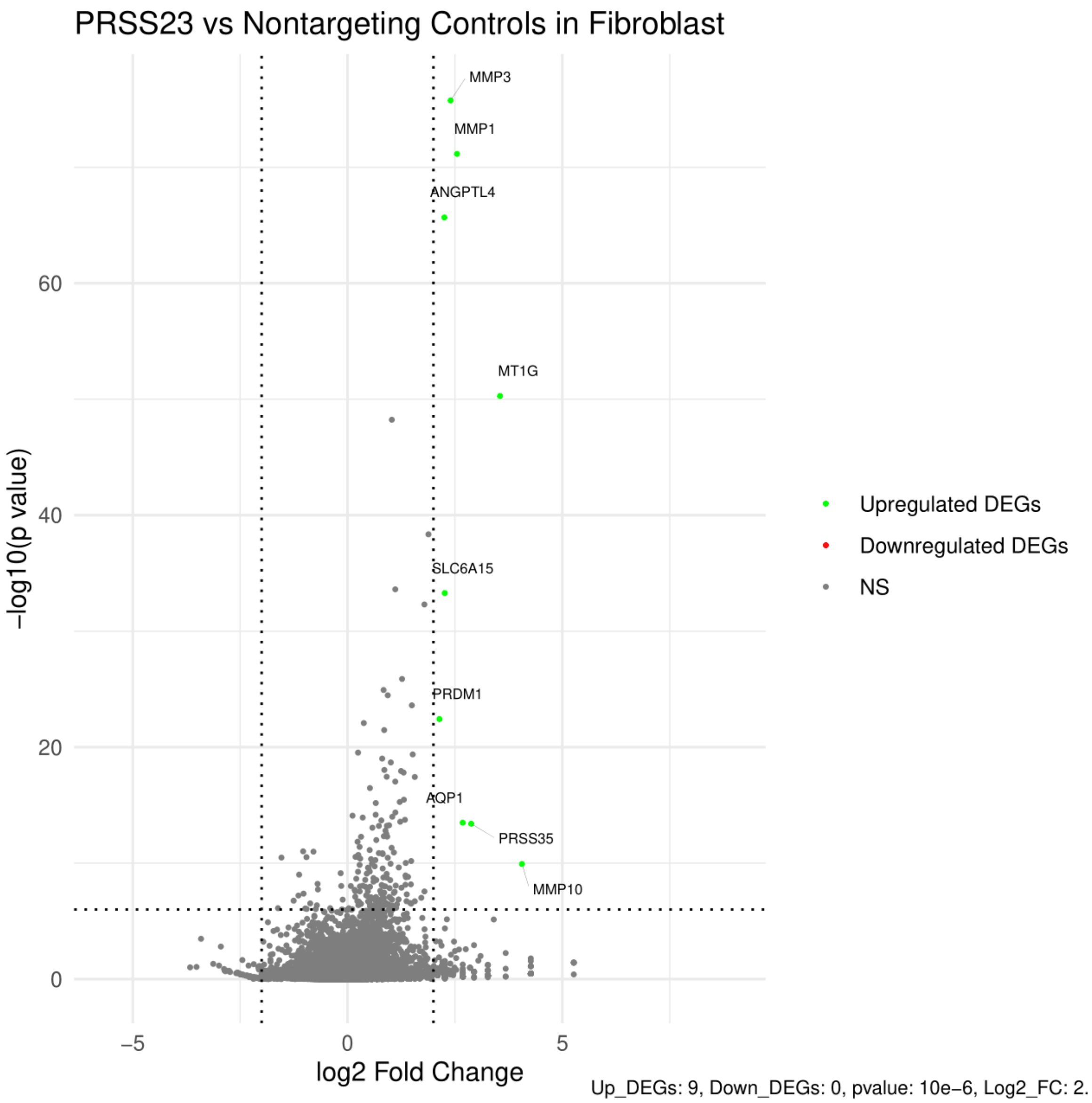
PRSS23 vs Nontargeting Controls in Fibroblast
MMP3
MMP1
ANGPTL4
MT1G
SLC6A15
PRDM1
AQP1
PRSS35
MMP10
−log10(p value)
60
40
20
0
−5
0
5
log2 Fold Change
Upregulated DEGs
Downregulated DEGs
NS
Up_DEGs: 9, Down_DEGs: 0, pvalue: 10e−6, Log2_FC: 2.

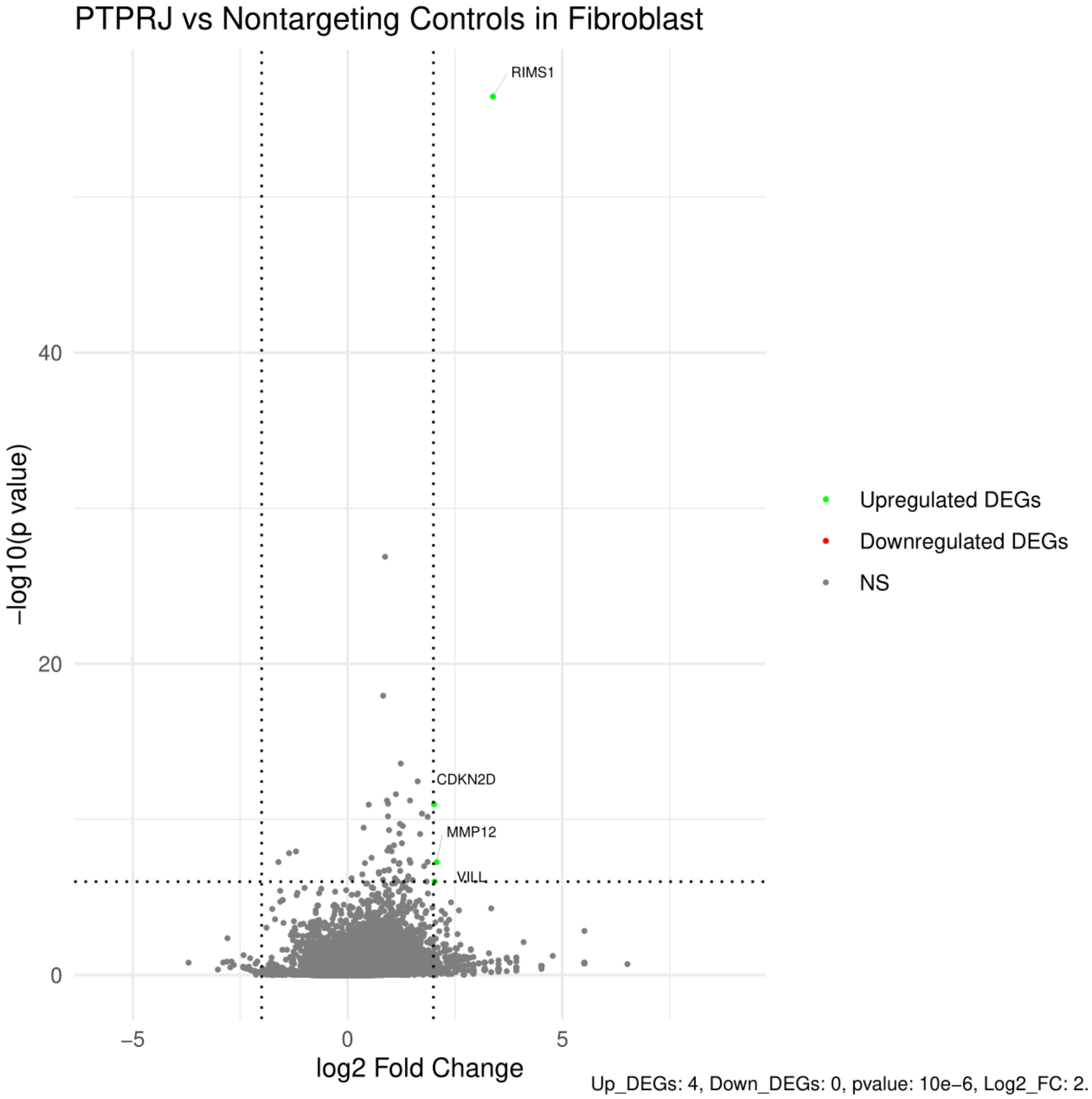
PTPRJ vs Nontargeting Controls in Fibroblast
RIMS1
CDKN2D
MMP12
VILL
40
20
0
−log10(p value)
−5
0
5
log2 Fold Change
Upregulated DEGs
Downregulated DEGs
NS
Up_DEGs: 4, Down_DEGs: 0, pvalue: 10e−6, Log2_FC: 2.

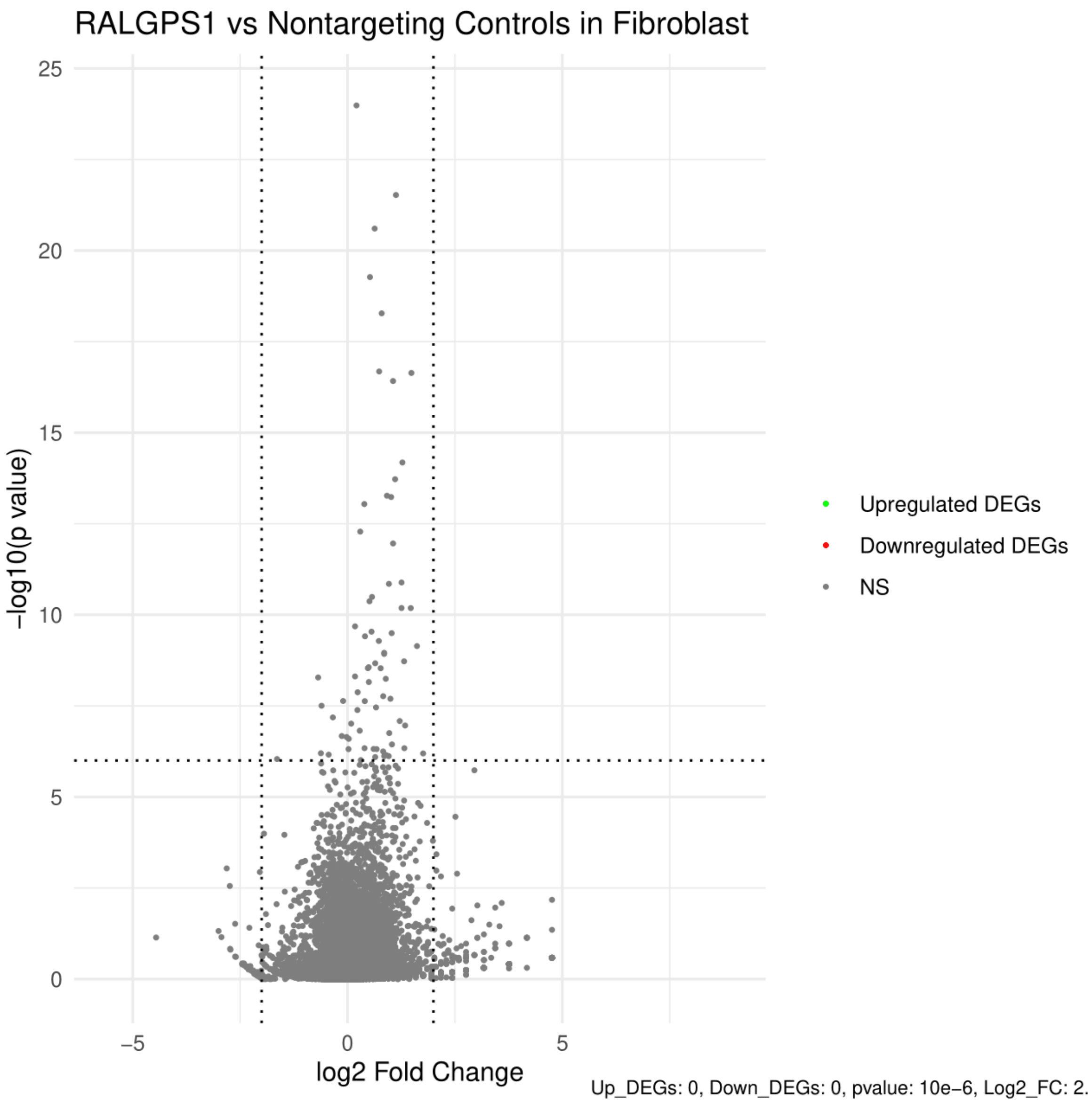
RALGPS1 vs Nontargeting Controls in Fibroblast
25
20
15
10
5
0
−log10(p value)
−5
0
5
log2 Fold Change
Upregulated DEGs
Downregulated DEGs
NS
Up_DEGs: 0, Down_DEGs: 0, pvalue: 10e−6, Log2_FC: 2.

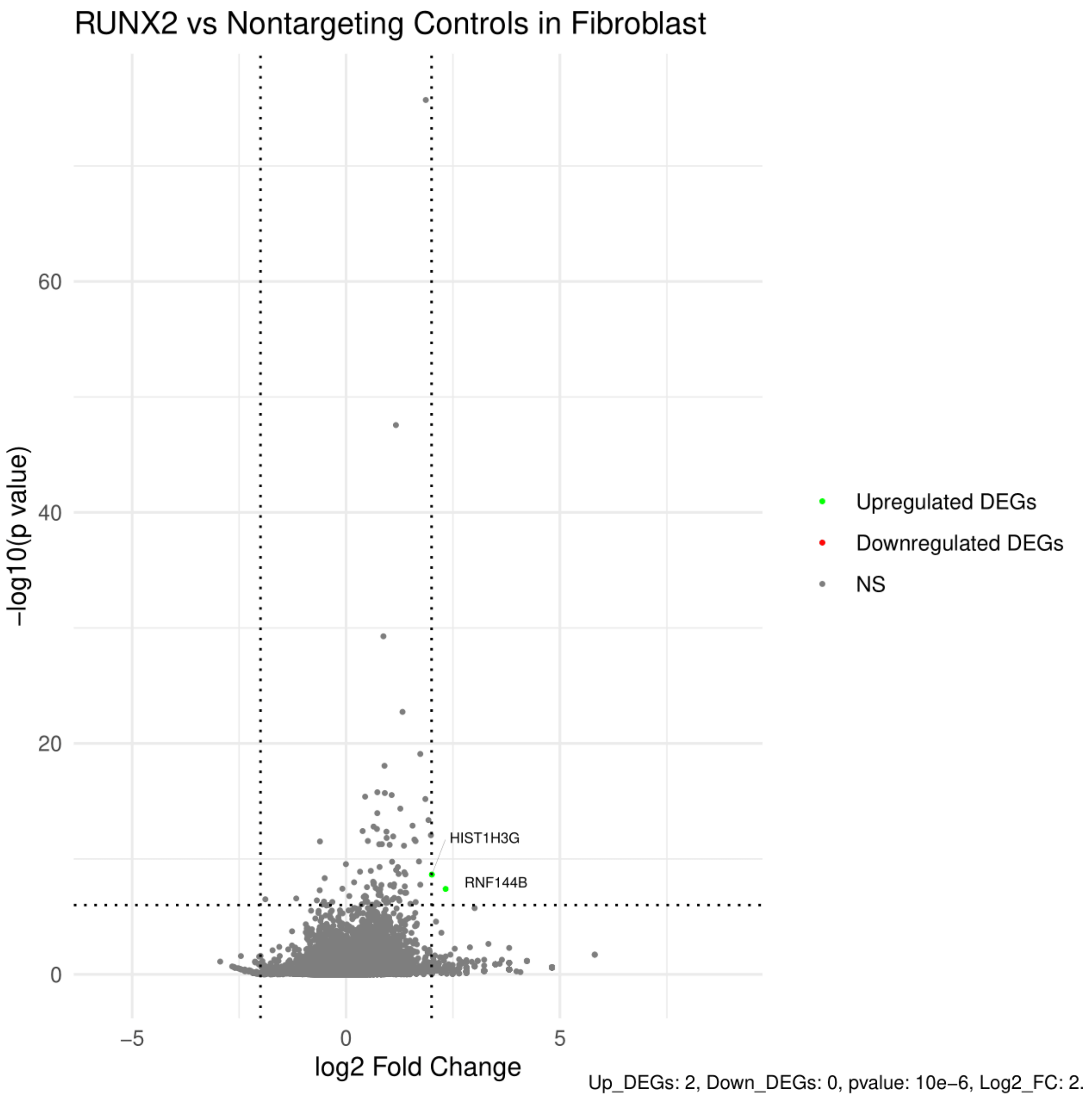

RUNX2 vs Nontargeting Controls in Fibroblast
60
40
20
0
−log10(p value)
−5
0
5
log2 Fold Change
HIST1H3G
RNF144B
Upregulated DEGs
Downregulated DEGs
NS
Up_DEGs: 2, Down_DEGs: 0, pvalue: 10e−6, Log2_FC: 2.

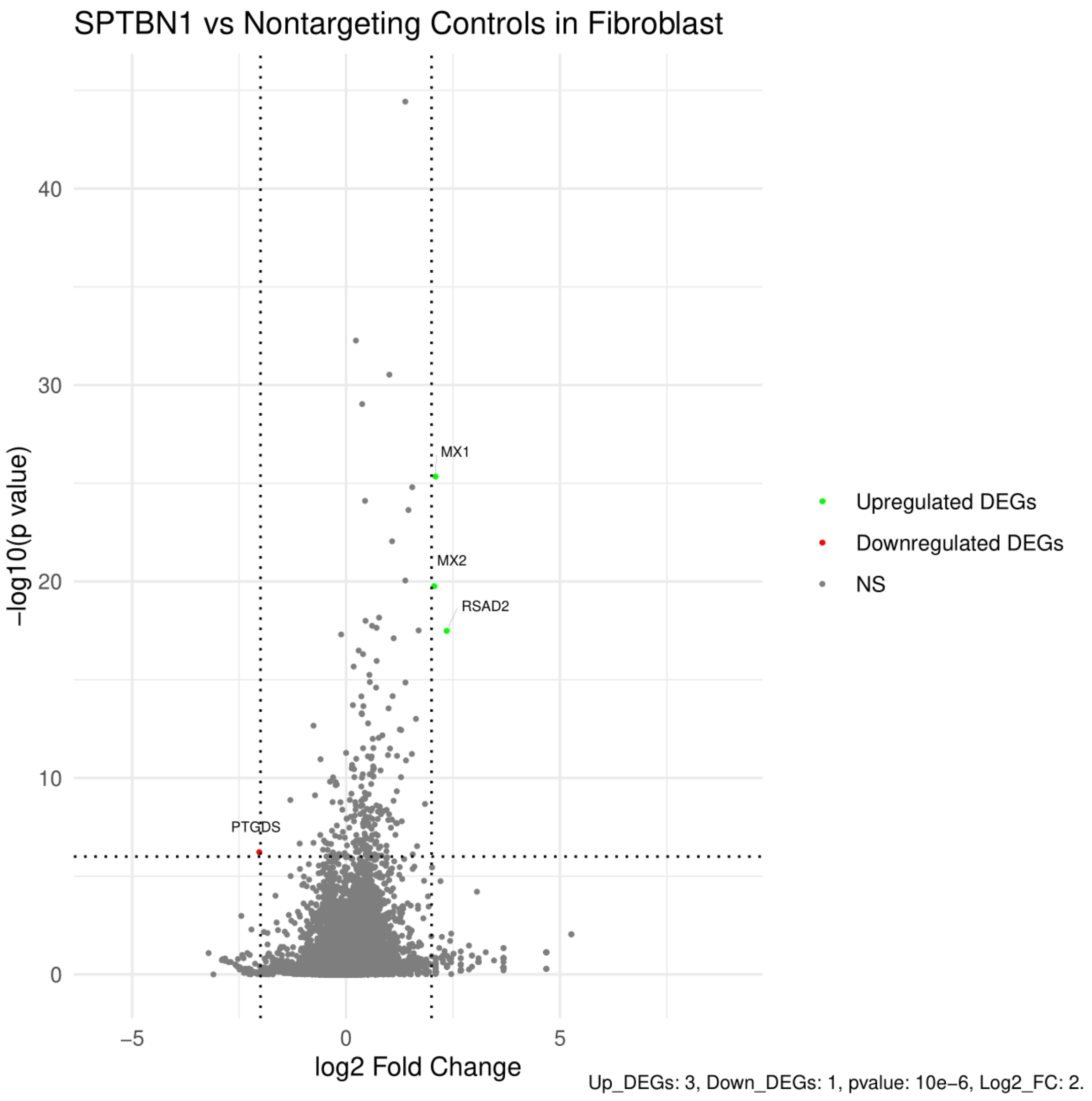
SPTBN1 vs Nontargeting Controls in Fibroblast
40
30
20
10
0
−log10(p value)
MX1
MX2
RSAD2
PTGDS
−5
0
5
log2 Fold Change
Upregulated DEGs
Downregulated DEGs
NS
Up_DEGs: 3, Down_DEGs: 1, pvalue: 10e−6, Log2_FC: 2.

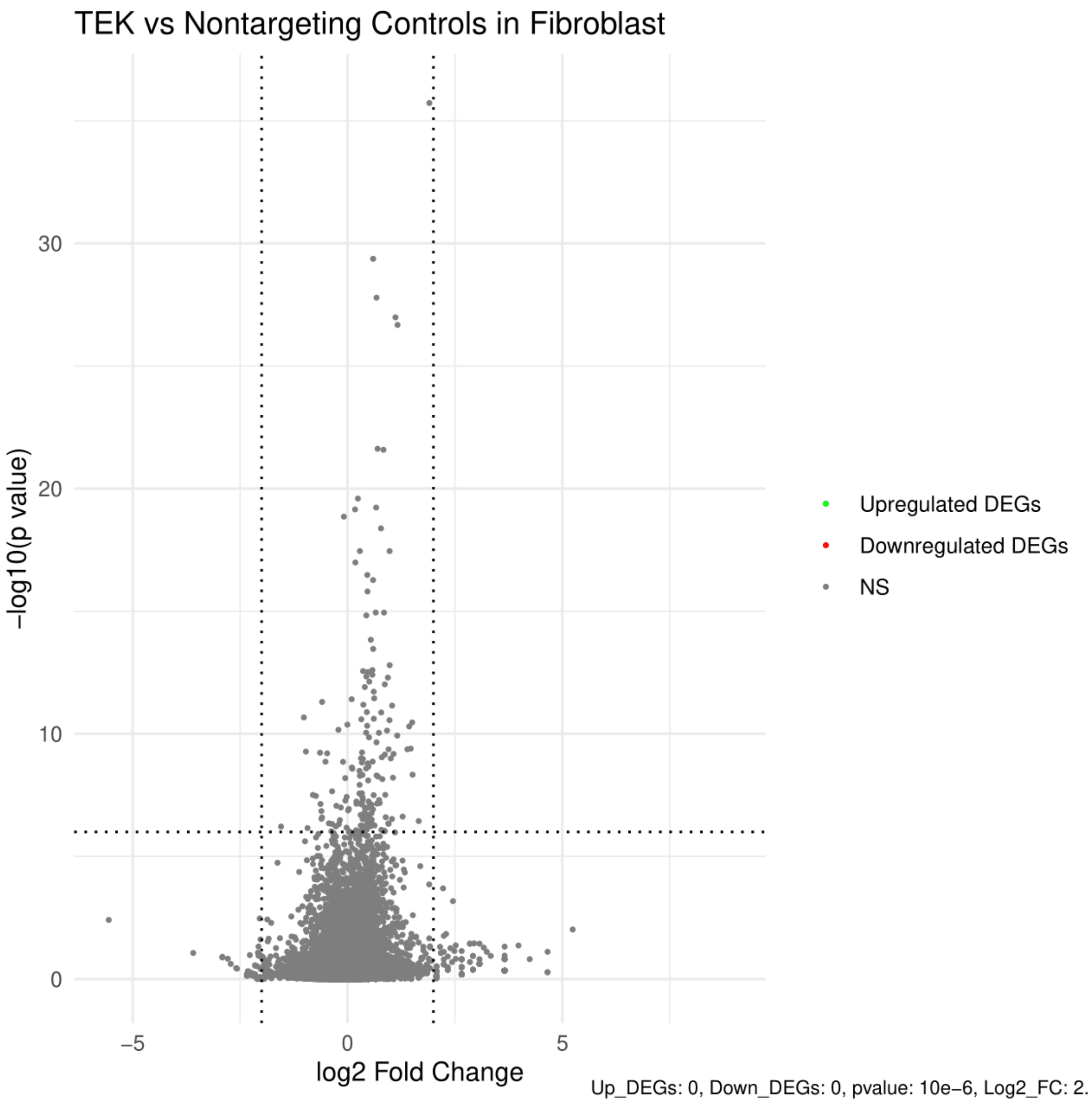
TEK vs Nontargeting Controls in Fibroblast
30
20
10
0
−log10(p value)
−5
0
5
log2 Fold Change
Upregulated DEGs
Downregulated DEGs
NS
Up_DEGs: 0, Down_DEGs: 0, pvalue: 10e−6, Log2_FC: 2.

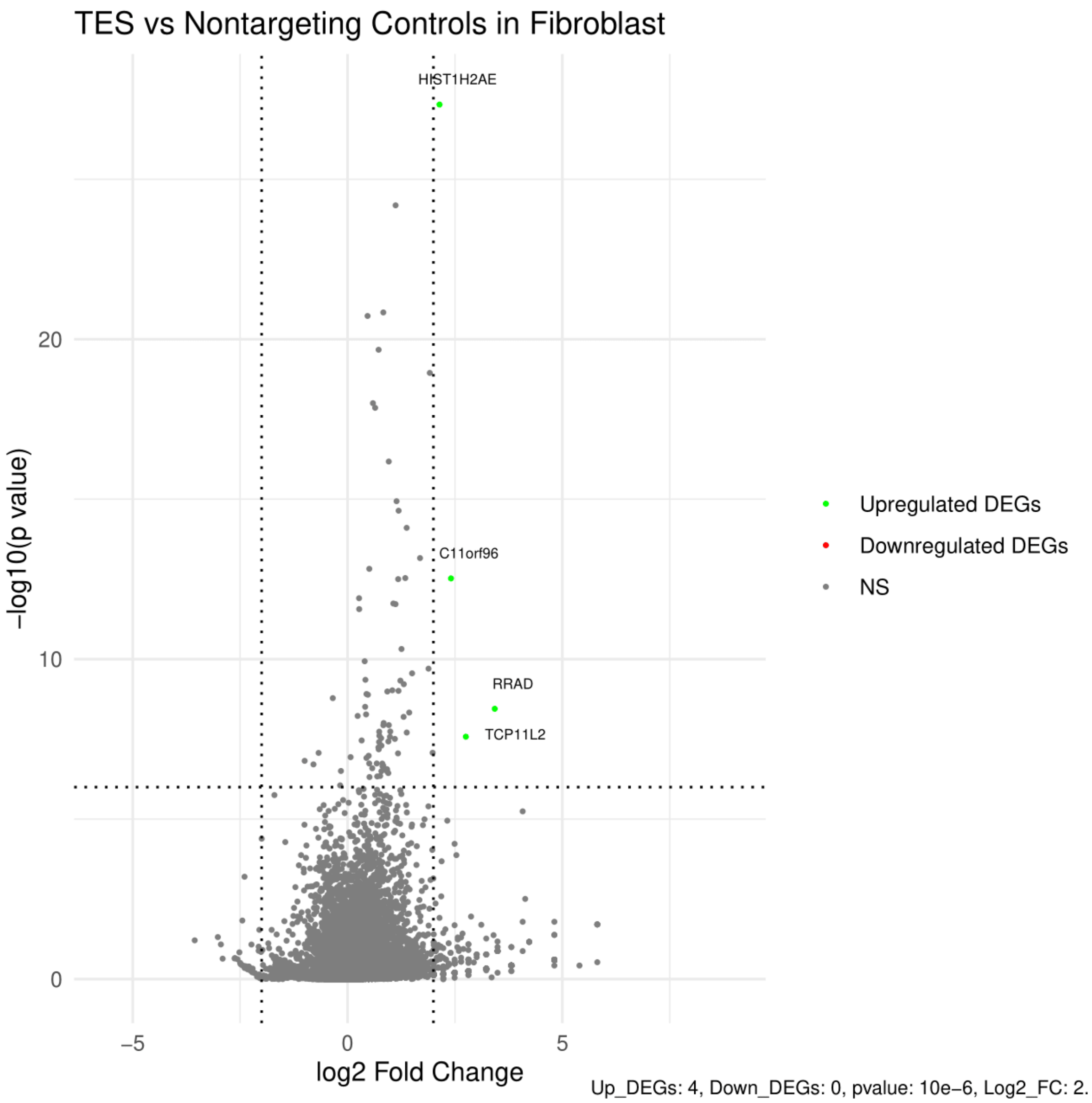
TES vs Nontargeting Controls in Fibroblast
HIST1H2AE
C11orf96
RRAD
TCP11L2
20
10
0
−log10(p value)
−5
0
5
log2 Fold Change
Upregulated DEGs
Downregulated DEGs
NS
Up_DEGs: 4, Down_DEGs: 0, pvalue: 10e−6, Log2_FC: 2.

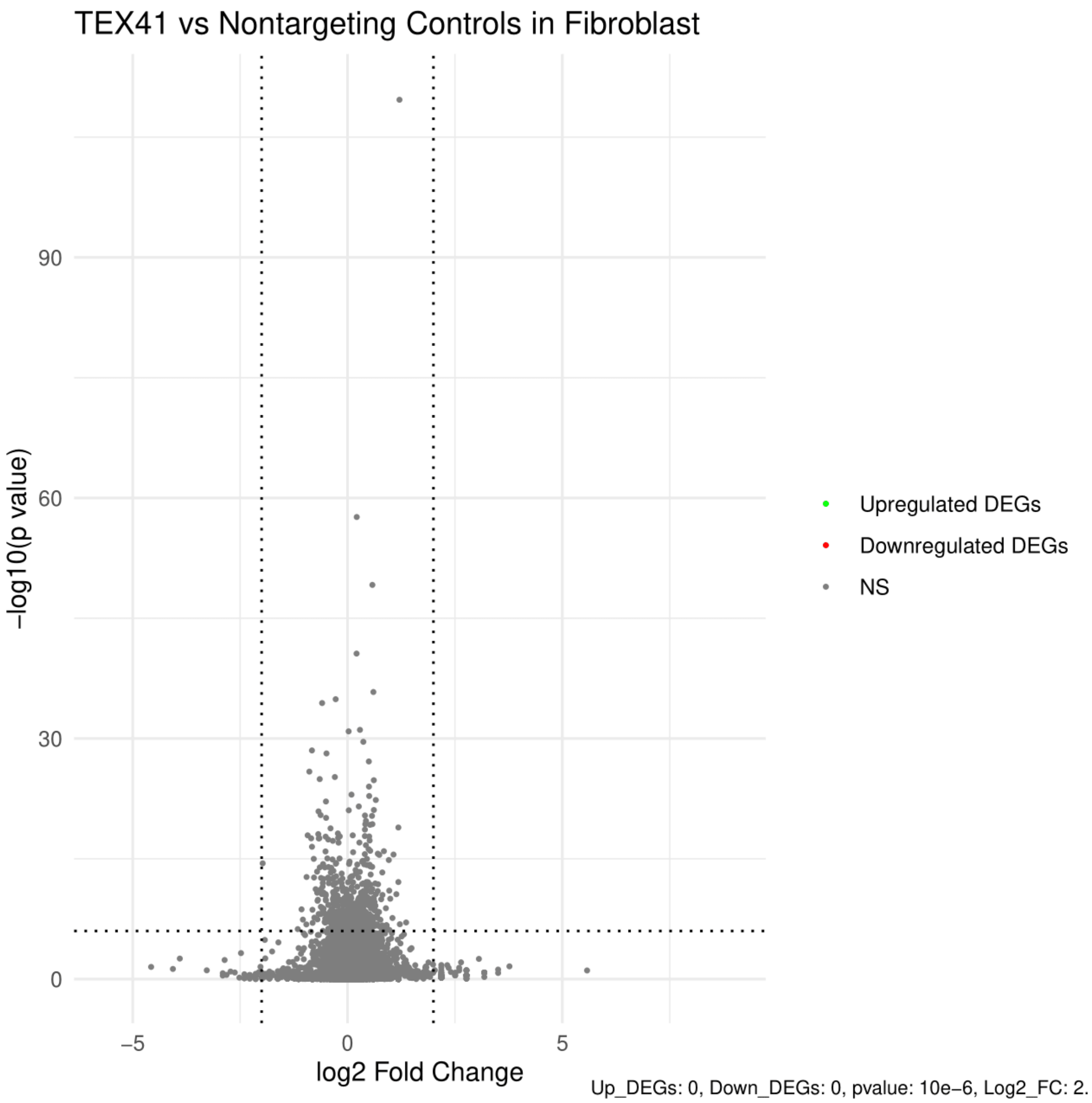
TEX41 vs Nontargeting Controls in Fibroblast
90
60
30
0
−log10(p value)
−5
0
5
log2 Fold Change
Upregulated DEGs
Downregulated DEGs
NS
Up_DEGs: 0, Down_DEGs: 0, pvalue: 10e−6, Log2_FC: 2.

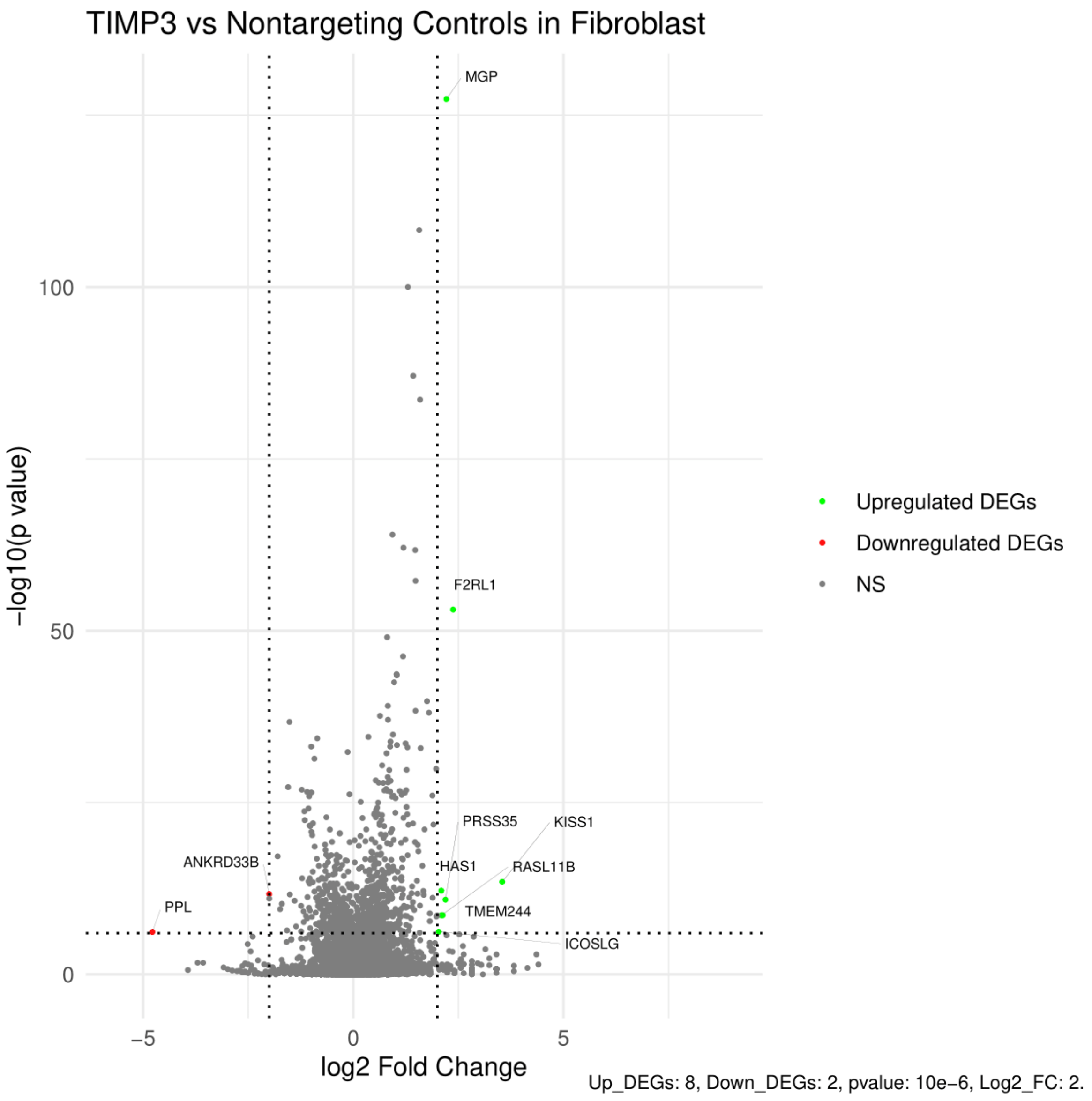
TIMP3 vs Nontargeting Controls in Fibroblast
MGP
F2RL1
PRSS35
KISS1
ANKRD33B
HAS1
RASL11B
PPL
TMEM244
ICOSLG
100
50
0
−log10(p value)
−5
0
5
log2 Fold Change
Upregulated DEGs
Downregulated DEGs
NS
Up_DEGs: 8, Down_DEGs: 2, pvalue: 10e−6, Log2_FC: 2.

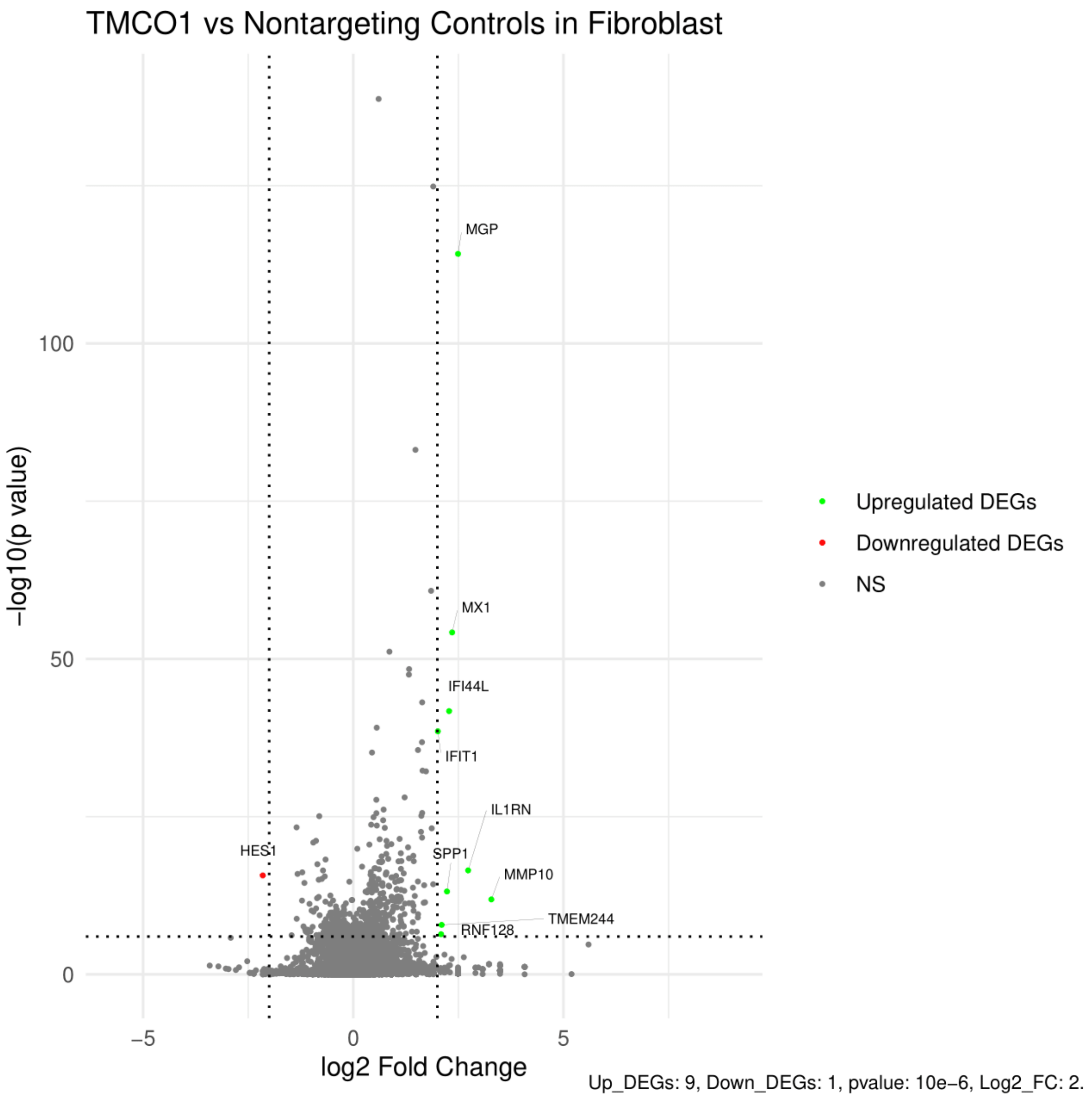
TMCO1 vs Nontargeting Controls in Fibroblast
MGP
MX1
IFI44L
IFIT1
IL1RN
HES1
SPP1
MMP10
TMEM244
RNF128
100
50
0
−5
0
5
−log10(p value)
log2 Fold Change
Upregulated DEGs
Downregulated DEGs
NS
Up_DEGs: 9, Down_DEGs: 1, pvalue: 10e−6, Log2_FC: 2.

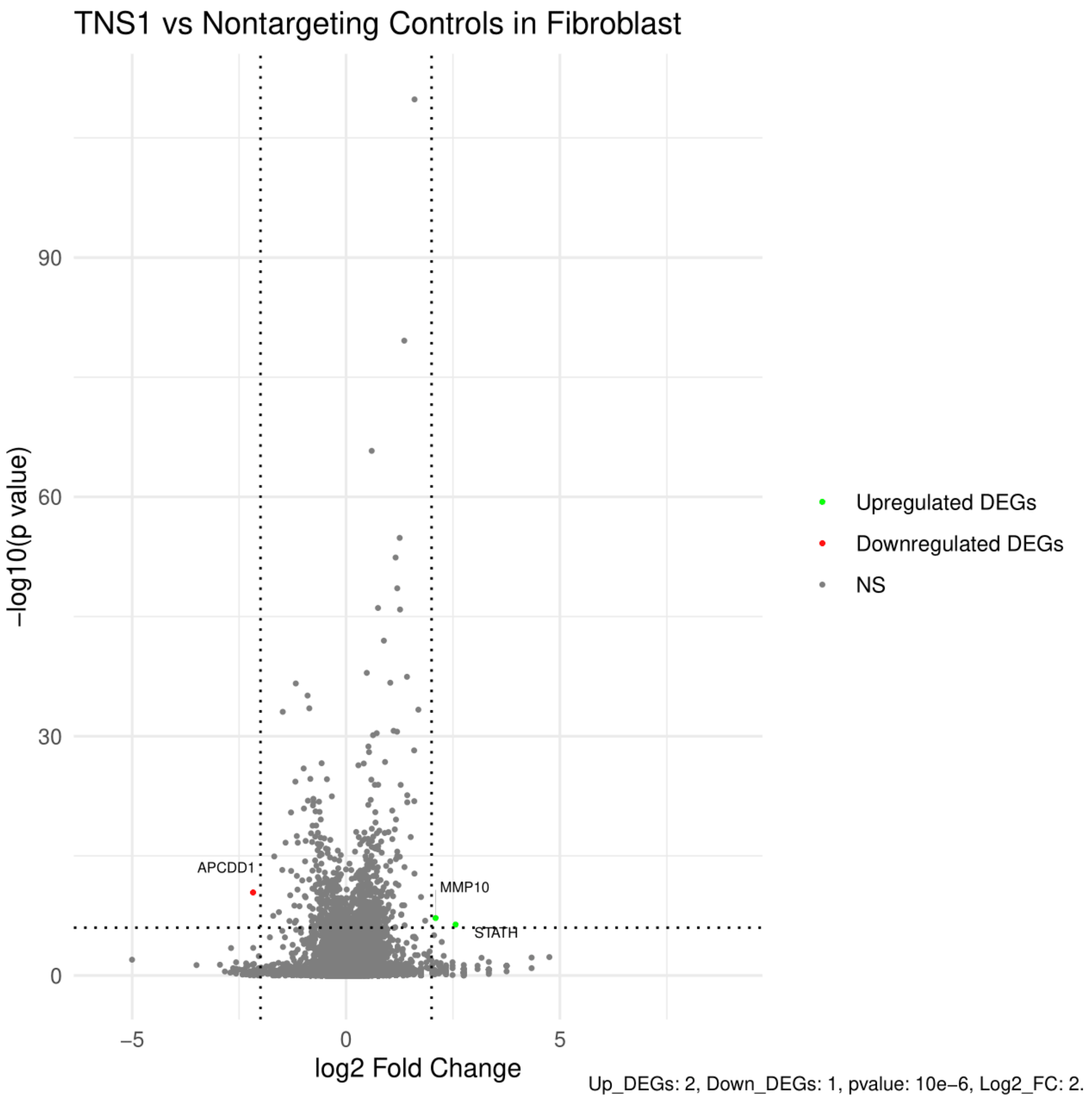
TNS1 vs Nontargeting Controls in Fibroblast
−log10(p value)
90
60
30
0
−5
0
5
log2 Fold Change
APCDD1
MMP10
STATH
Upregulated DEGs
Downregulated DEGs
NS
Up_DEGs: 2, Down_DEGs: 1, pvalue: 10e−6, Log2_FC: 2.

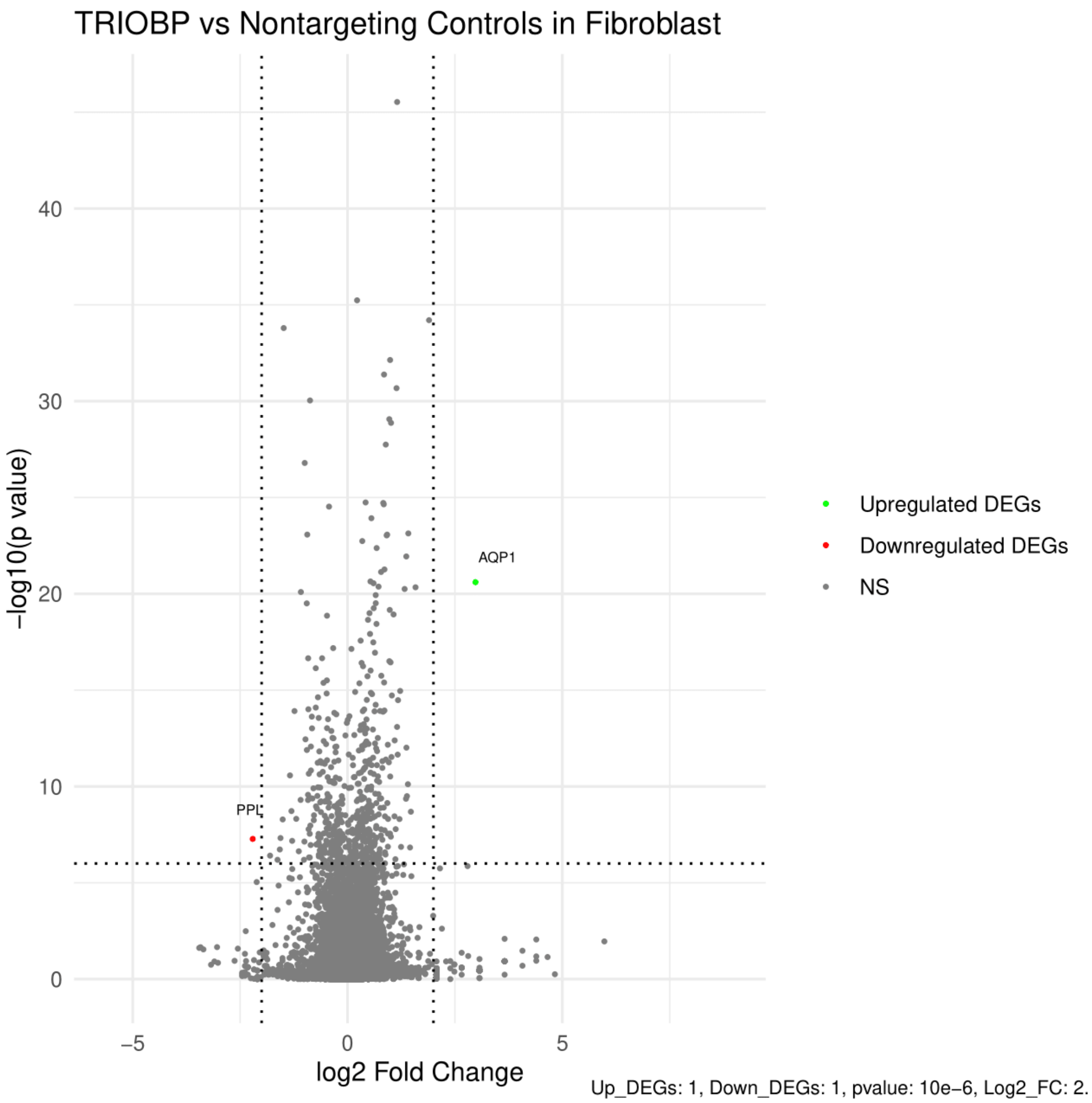
TRIOBP vs Nontargeting Controls in Fibroblast
40
30
20
10
0
−log10(p value)
AQP1
PPL
−5
0
5
log2 Fold Change
Upregulated DEGs
Downregulated DEGs
NS
Up_DEGs: 1, Down_DEGs: 1, pvalue: 10e−6, Log2_FC: 2.

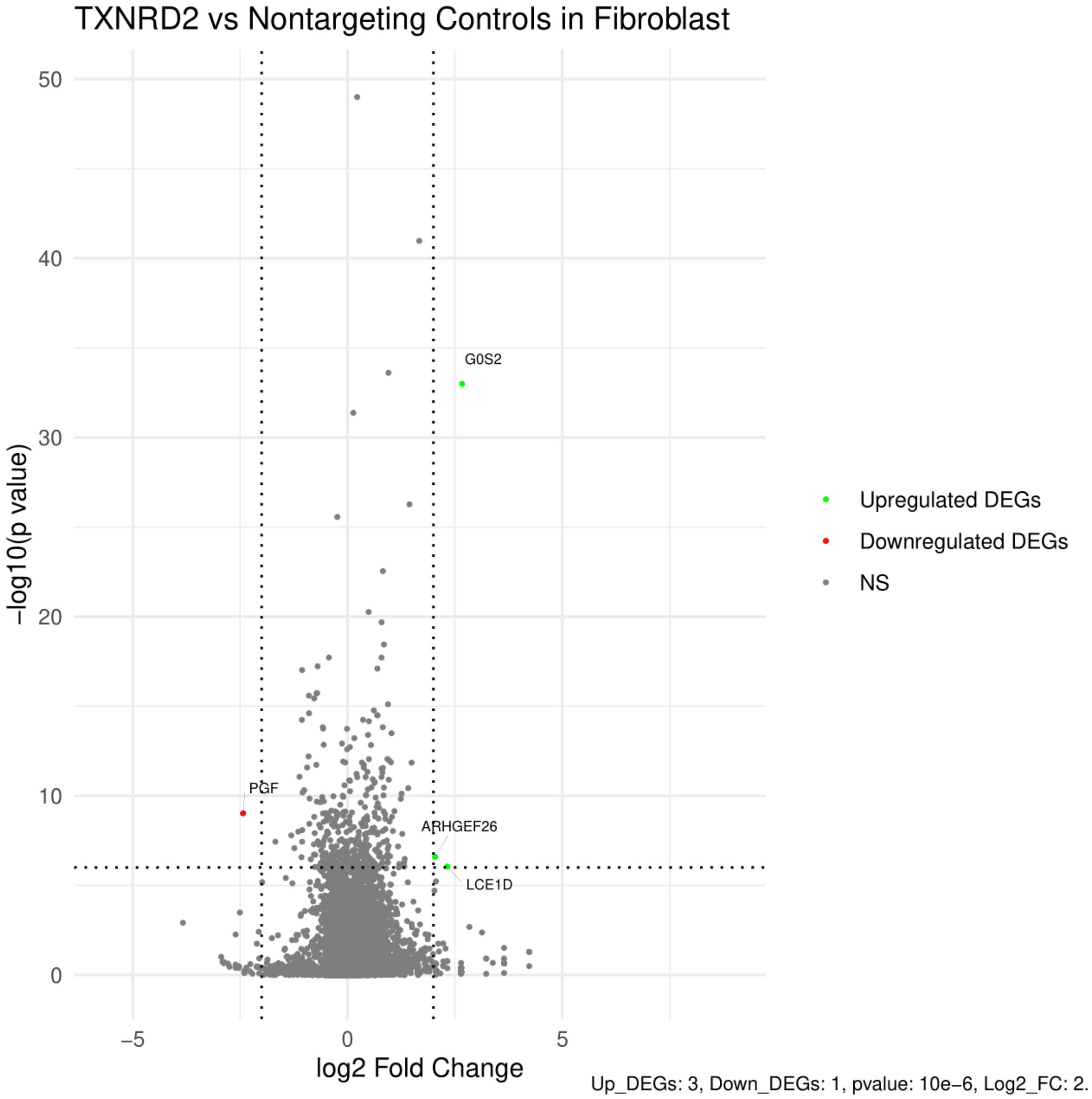
TXNRD2 vs Nontargeting Controls in Fibroblast
50
40
30
20
10
0
−log10(p value)
−5
0
5
log2 Fold Change
G0S2
PGF
ARHGEF26
LCE1D
Upregulated DEGs
Downregulated DEGs
NS
Up_DEGs: 3, Down_DEGs: 1, pvalue: 10e−6, Log2_FC: 2.

ZNF280D vs Nontargeting Controls in Fibroblast

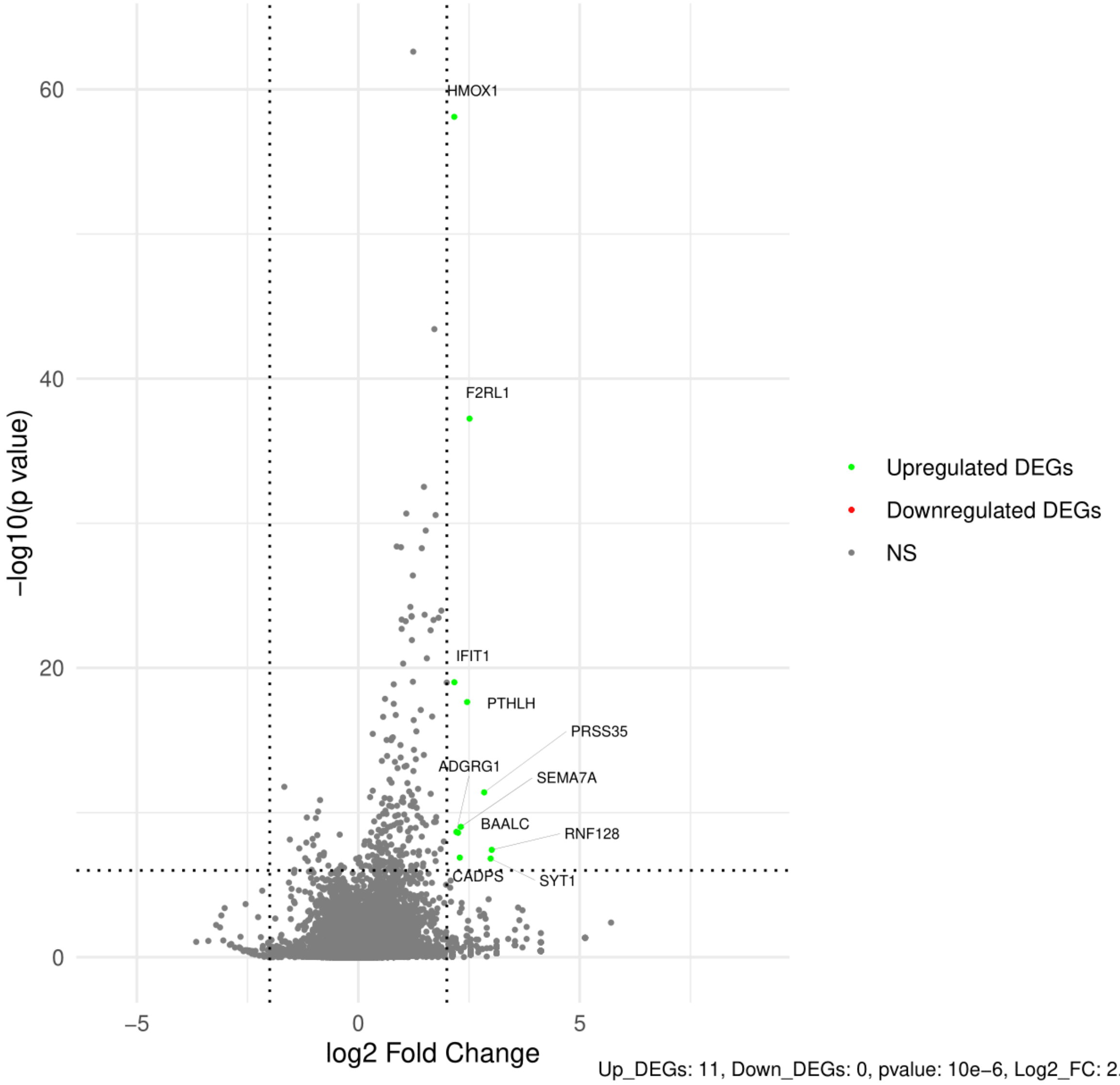

Up_DEGs: 11, Down_DEGs: 0, pvalue: 10e−6, Log2_FC: 2.
